# Decarboxylative Nickel- and Photoredox-Catalyzed Aminocarbonylation of (Hetero)Aryl Bromides

**DOI:** 10.1021/acs.orglett.3c02389

**Published:** 2023-10-05

**Authors:** Valeriia Hutskalova, Farhan Bou Hamdan, Christof Sparr

**Affiliations:** † Department of Chemistry, University of Basel, St. Johanns-Ring 19, 4056 Basel, Switzerland; ‡ Syngenta Crop Protection AG, Crop Protection Research, Schaffhauserstrasse 101, CH-4332 Stein, Switzerland

## Abstract

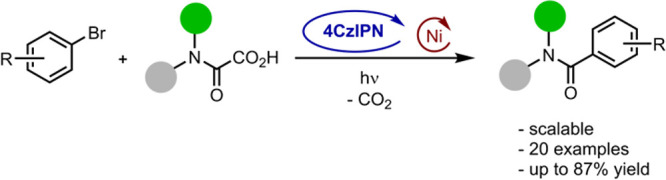

An efficient methodology for the photoredox- and nickel-catalyzed aminocarbonylation of (hetero)aryl bromides was developed. The utilization of readily available oxamic acids, the application of a broadly used organic photoredox catalyst (4CzIPN), and mild reaction conditions make this transformation an appealing alternative to classical amidation procedures. The generation of carbamoyl radicals was supported by trapping reactions with a hydrogen atom transfer catalyst in the presence of D_2_O, yielding the deuterated formamide. The generality of this deuteration protocol was confirmed for various oxamic acids.

The amide bond is an exceptionally important structural motif not only as the backbone of peptides but also as the linkage of numerous small bioactive pharmaceuticals and agrochemicals. Owing to the high abundance of amides in the final bioactive products^1,2^ or synthetic intermediates, amidation reactions belong to the most frequently performed transformations.^3^ Therefore, significant efforts have been devoted to the development of new amidation protocols over the last years.^4−6^ The currently available synthetic approaches to amides can be grouped into three general strategies based on the bond disconnections for each specific retrosynthetic consideration (Scheme 1A). Direct coupling of carboxylic acids and amines (route a) clearly represents the most common route to amides. However, without preactivation, the formation of unreactive carboxylate-ammonium salts makes this approach suitable only for a very limited range of substrates that tolerate harsh reaction conditions. The generation of more reactive carboxylic acid derivatives by the application of stoichiometric amounts of an activating or coupling reagent routinely allows for milder reaction conditions. However, poor atom economy, high cost, and certain safety concerns about commonly utilized coupling agents^7^ along with a significant amount of generated waste limit this synthetic approach and negatively impact its suitability for industrial applications. Transition-metal-catalyzed aminocarbonylation (route b),^8,9^ in turn, represents a three-component methodology that transforms readily available aryl halides to amides. However, the use of toxic CO gas, expensive palladium catalysts, and ligands hampers the application of this reaction in industry on a large scale. Route c, on the other hand, relies on the initial formation of the desired N—C(=O) bond, followed by the later creation of a C—C bond with the required substituent. This strategy is realized by the generation of a carbamoyl radical as a reactive intermediate. The corresponding *N-*hydroxyphthalimide esters^10,11^ and 4-carbamoyl-1,4-dihydropyridines^12^ thereby proved to be useful carbamoyl radical precursors (Scheme 1B). However, in both cases, poor atom economy and waste arising from the decarboxylative auxiliary are noteworthy disadvantages compared with substrates that decarboxylate without byproducts (Scheme 1C). Recently, the Maiti group reported a silyl-radical-mediated halogen abstraction as a tool to access carbamoyl radicals starting from carbamoyl chlorides (Scheme 1D).^13^ In contrast, the readily available and expediently utilized oxamic acids represent an attractive alternative as carbamoyl radical precursors with only CO_2_ contributing to the waste stream.^14^ Interestingly, Li and co-workers disclosed a single example of an amide preparation by a decarboxylative nickel-catalyzed cross-coupling (Scheme 1C).^15^ However, this example required a high photocatalyst loading (20 mol %), and the yield was compromised. Moreover, the Fu group reported a decarboxylative coupling of potassium oxalate monoamides with aryl halides with precious iridium and palladium catalysts.^16^ Considering the need for new efficient amidation methodologies, we hence aimed to explore the scope of decarboxylative nickel- and organophotoredox-catalyzed aminocarbonylation of aryl halides (Scheme 1E).

**Scheme 1:**
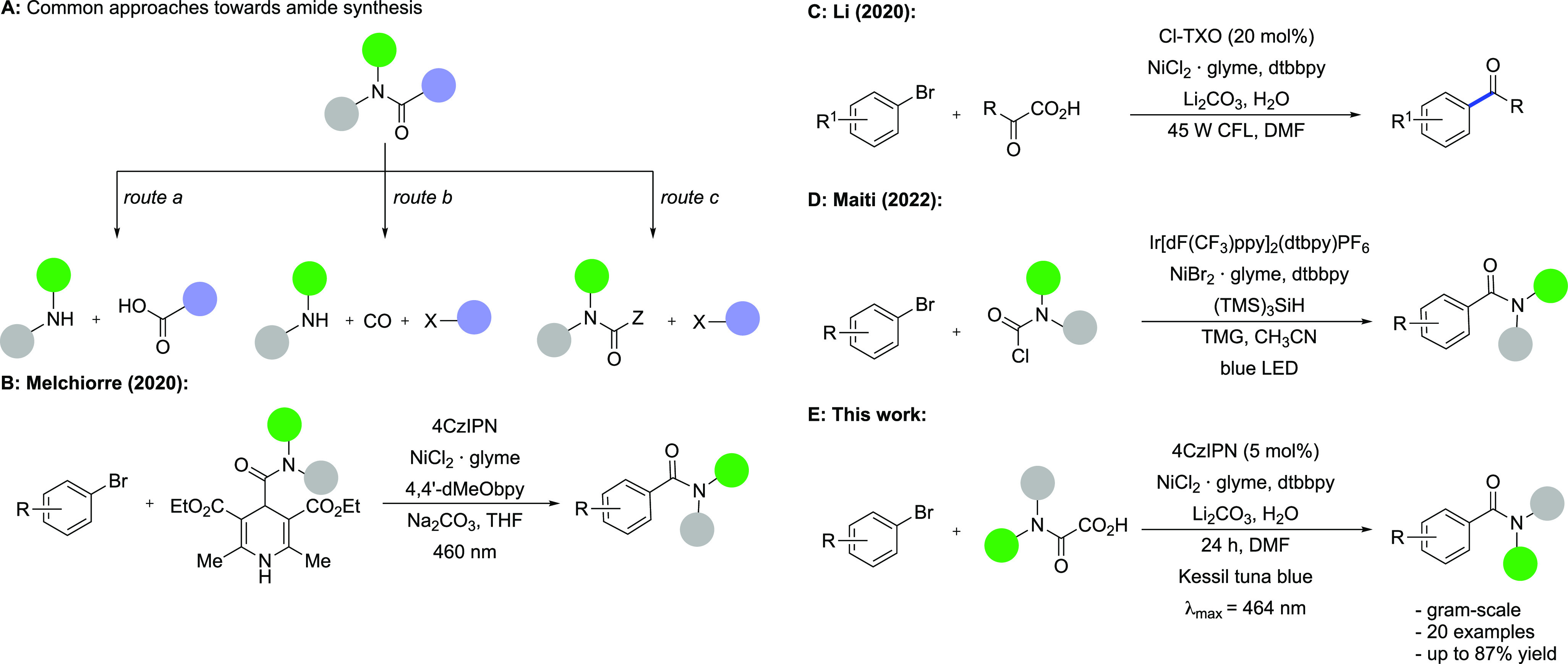
Classical Synthetic Routes and Photoredox-Catalytic Approaches toward Amides

We initiated our studies by an expeditious substrate preparation by an operationally simple two-step sequence involving the acylation of *N*-methylaniline with methyl 2-chloro-2-oxoacetate followed by hydrolysis of the obtained methyl ester. With oxamic acid **1a** in hand, we embarked on the optimization of the reaction conditions. For a comparison with the reported example,^15^ we started our optimizations utilizing 2-chloro-thioxanthen-9-one (Cl-TXO) as photocatalyst. However, in our hands neither the change of the light source nor the diversification of the nickel salt or solvent allowed a yield of more than 30% to be achieved (details are provided in the Supporting Information). Notably, lowering the nickel loading to 5 mol % led to a decreased conversion and yield, while diverse bipyridine ligands showed insignificant differences in the reaction outcome (Table S2). Variations of the base also did not result in any noteworthy change in the reaction performance (Table 1, entries 1–4). Furthermore, the application of phthalimide^17^ as an additive was also ineffective for the improvement of the yield (Table 1, entry 5). We next proceeded with the evaluation of diversified photocatalysts. While acridinium salts proved to be unsuitable for this transformation (Table S1), transition-metal-based photocatalyst ((Ir(dF(CF_3_)ppy)_2_(dtbpy))PF_6_ delivered the desired product in 30% yield (Table 1, entry 7). In contrast, representatives of the cyanoarene-based organic donor–acceptor photocatalysts showed promising results. In particular, full conversion of the 4-bromobenzonitrile was achieved with 1,2,3,5-tetrakis(carbazol-9-yl)-4,6-dicyanobenzene (4CzIPN) and pentacarbazolylbenzonitrile (5CzBN) (Table 1, entries 9, 14). On the other hand, two other members of this class of photocatalysts led to either low yield or no reaction (Table 1, entries 13, 15). These results were rationalized by the insufficient ground state reduction potential of 3CzClIPN (*E*_1/2_(PC/PC^•–^) = −1.16 V)^18^ for nickel reductions (*E*_1/2_(Ni^II^/Ni^0^) = −1.20 V)^15^ and the poor oxidizing properties of 3DPA2FBN (*E*_1/2_(PC*/PC^•–^) = +0.92 V) that negatively affect the decarboxylation of oxamic acid. The photocatalyst loading also appeared as a decisive factor, since an increase of the loading to more than 3% caused a notable drop in yield (Table 1, entries 10–12), while the application of 1 mol % of 4CzIPN was insufficient to achieve full conversion (Table 1, entry 8). Further extensive reaction condition optimization revealed DMF and NiCl_2_·glyme as the ideal solvent and nickel precursor (Table 1, entry 21). After establishing these suitable reaction conditions, we next set out to assess the substrate scope by varying the natures of both the oxamic acid and the coupling partner. To our delight, oxamic acids bearing halides (−Cl, −F) and electron-donating groups (−Me, −OMe) all yielded products (**2b**, **2c**, **2d**, **2j**) with high isolated yields in the range of 75–87% (Scheme 2). Moreover, both *N*-alkyl-*N*-aryl and *N*,*N*-dialkyl derivatives were identified as suitable substrates tolerating different alkyl groups, such as benzyl (**2h**) and sterically hindered adamantyl (**2g**) or isopropyl residues (**2k**). Remarkably, *N*,*N*-diaryl oxamic acid (**1e**) was also successfully subjected to the reaction, giving the corresponding product with high yield (**2e**). However, the methodology was unsuitable for 2-oxo-2-(phenylamino)acetic acid (**1m**), presumably due to the lower stability of the generated *N*-arylcarbamoyl radical and its tendency for decarbonylation.^19,20^ Interestingly, no formation of the desired product was observed for the intramolecular version of the transformation (Scheme 2). Next, the generality of the reaction was explored for different aryl bromides. Substrates containing electron-withdrawing substituents (−CF_3_, −CN, and −CO_2_Me) yielded the desired products, albeit with low yields (**3a**, **3b**, and **3c**), while aryl bromides possessing an electron-donating group (−OMe) did not participate in the desired transformation. This observation could be due to a slow oxidative addition step of electron-rich aryl halides which can cause aggregation of low-valent nickel complexes.^21^ Sterically hindered aryl bromides also represent a limitation of the methodology. Several electron-deficient heteroaryl bromides such as 3-bromoquinoline and 2-bromopyrazine gave rise to products with moderate or low yields (**3e**, **3g**). We next investigated the efficiency of the developed aminocarbonylation procedure of (hetero)aryl bromides by demonstrating that it is suitably performed on a gram scale. The product **2j** (Scheme 2) was thereby prepared with an isolated yield of 84% on a 5 mmol scale with no need to change the reaction parameters. A potential mechanism of this dual catalytic transformation involves the excitation of the photocatalyst 4CzIPN (PC) by irradiation with visible light.^15^ The obtained PC* next oxidizes the salt formed of the oxamic acid, leading to the corresponding radical that rapidly undergoes decarboxylation to form the carbamoyl radical (Scheme 3). The resulting reduced species of the photocatalyst then participates in the generation of L_*n*_Ni^0^ by a L_*n*_Ni^I^X reduction. The reduced nickel complex results in an oxidative addition of the aryl bromide, followed by the interception of the carbamoyl radical. Finally, a reductive elimination yields the desired amide product and regenerates L_*n*_Ni^I^X. Formation of trace amounts of *N*-methyl-*N*-phenylformamide (<5% yield) as a side-product during the preparation of **2a** serves as an indication of the generation of carbamoyl radicals according to this mechanism. To further support the involvement of the carbamoyl radicals, we explored the preparation of deuterated formamides by synergistic thiol and photoredox catalysis utilizing D_2_O as an inexpensive deuterium source. Recently, the Li group developed an approach to C1-deuterated aldehydes relying on the photoredox-catalyzed decarboxylation of α-oxo carboxylic acids.^22^ In contrast, the preparation of deuterated formamides starting from readily available oxamic acids remained unknown. We therefore initiated our investigation using a reaction system similar to the one developed for the aminocarbonylation of aryl bromides using 4CzIPN as a photocatalyst, Li_2_CO_3_ as a base, and DMF as a reaction medium (Table 2). Notably, performing the reaction with 2,4,6-trimethylthiophenol as a hydrogen atom transfer catalyst gave a 43% yield with high D incorporation (90%) (Table 2, entry 4). Reducing the amount of D_2_O led to both decreased yield and D incorporation (Table 2, entry 3), while doubling the quantity of D_2_O caused a significant drop in yield with only minor improvement in D incorporation (Table 2, entry 5). Furthermore, a comparison of the performances of Ir-based photocatalyst (Table 2, entries 1 and 2) with the organic one (4CzIPN) revealed that the latter is as efficient, validating that the use of precious metals is not needed for this transformation. Moreover, control experiments demonstrated that the presence of both photocatalyst and HAT catalyst is essential for the desired deuteration reaction (Table 2, entries 8, 10). Finally, we applied the deuteration protocol to four different oxamic acids that yielded desired products **4a**–**4d** with moderate yields and high D incorporation (90–92%) (Scheme 4). Therefore, this reaction can be used not only to evaluate the formation of carbamoyl radicals but also as an approach to deuterated formamides under mild metal-free conditions.

**Table 1:**
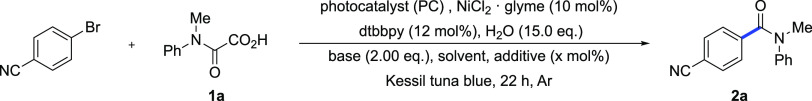
Optimization of the Reaction Conditions

Entry^a^	PC	PC (mol %)	Base	Solvent	Concentration	Modifications	Conversion^b^	Yield^c^ (Isolated yield)
1	Cl-TXO	20	Li_2_CO_3_	DMF	33 mM		39%	25%
2	Cl-TXO	20	Na_2_CO_3_	DMF	33 mM		49%	22%
3	Cl-TXO	20	K_2_CO_3_	DMF	33 mM		58%	28%
4	Cl-TXO	20	Cs_2_CO_3_	DMF	33 mM		49%	23%
5	Cl-TXO	20	Li_2_CO_3_	DMF	33 mM	Phthalimide^d^	56%	27%
6	di-*t*Bu-Mes-Acr^+^BF_4_^–^	5	Li_2_CO_3_	DMF	33 mM	SynLED	0%	0%
7	(Ir(dF(CF_3_)ppy)_2_(dtbpy))PF_6_	8	Li_2_CO_3_	DMF	33 mM		76%	31%
8	4CzIPN	1	Li_2_CO_3_	DMF	33 mM		78%	53%
9	4CzIPN	3	Li_2_CO_3_	DMF	33 mM		>95%	63% (51%)
10	4CzIPN	5	Li_2_CO_3_	DMF	33 mM		>95%	45%
11	4CzIPN	8	Li_2_CO_3_	DMF	33 mM		>95%	47%
12	4CzIPN	15	Li_2_CO_3_	DMF	33 mM		>95%	40%
13	3CzClIPN	3	Li_2_CO_3_	DMF	33 mM		0%	0%
14	5CzBN	3	Li_2_CO_3_	DMF	33 mM		>95%	63%
15	3DPA2FBN	3	Li_2_CO_3_	DMF	33 mM		44%	23%
16	4CzIPN	3	Li_2_CO_3_	DMF	33 mM	No H_2_O	>95%	45%
17	4CzIPN	3	Li_2_CO_3_	THF	33 mM		0%	0%
18	4CzIPN	3	Li_2_CO_3_	DMC	33 mM		0%	0%
19	4CzIPN	3	Li_2_CO_3_	CH_3_CN	33 mM		0%	0%
20	4CzIPN	3	Li_2_CO_3_	DMF	20 mM		>95%	36%
**21**	**4CzIPN**	**3**	**Li_2_CO_3_**	**DMF**	**50 mM**		**>95%**	**64%**

a Reaction conditions: 4-Bromobenzonitrile (18.2 mg, 100 μmol), **1a** (35.8 mg, 200 μmol), base (200 μmol), NiCl_2_·glyme (2.20 mg, 10.0 μmol), 4,4′-dtbbpy (4,4-di-*tert*-butyl-2,2-dipyridyl) (3.22 mg, 12.0 μmol), argon, 22 h, Kessil tuna blue as a light source (λ_max_ = 464 nm).

b Conversion based on ^1^H NMR analysis of the crude reaction mixture.

c Yields determined by ^1^H NMR analysis of the crude mixture using 1,2,4,5-tetramethylbenzene as an internal standard.

d 0.25 equiv.

**Scheme 2:**
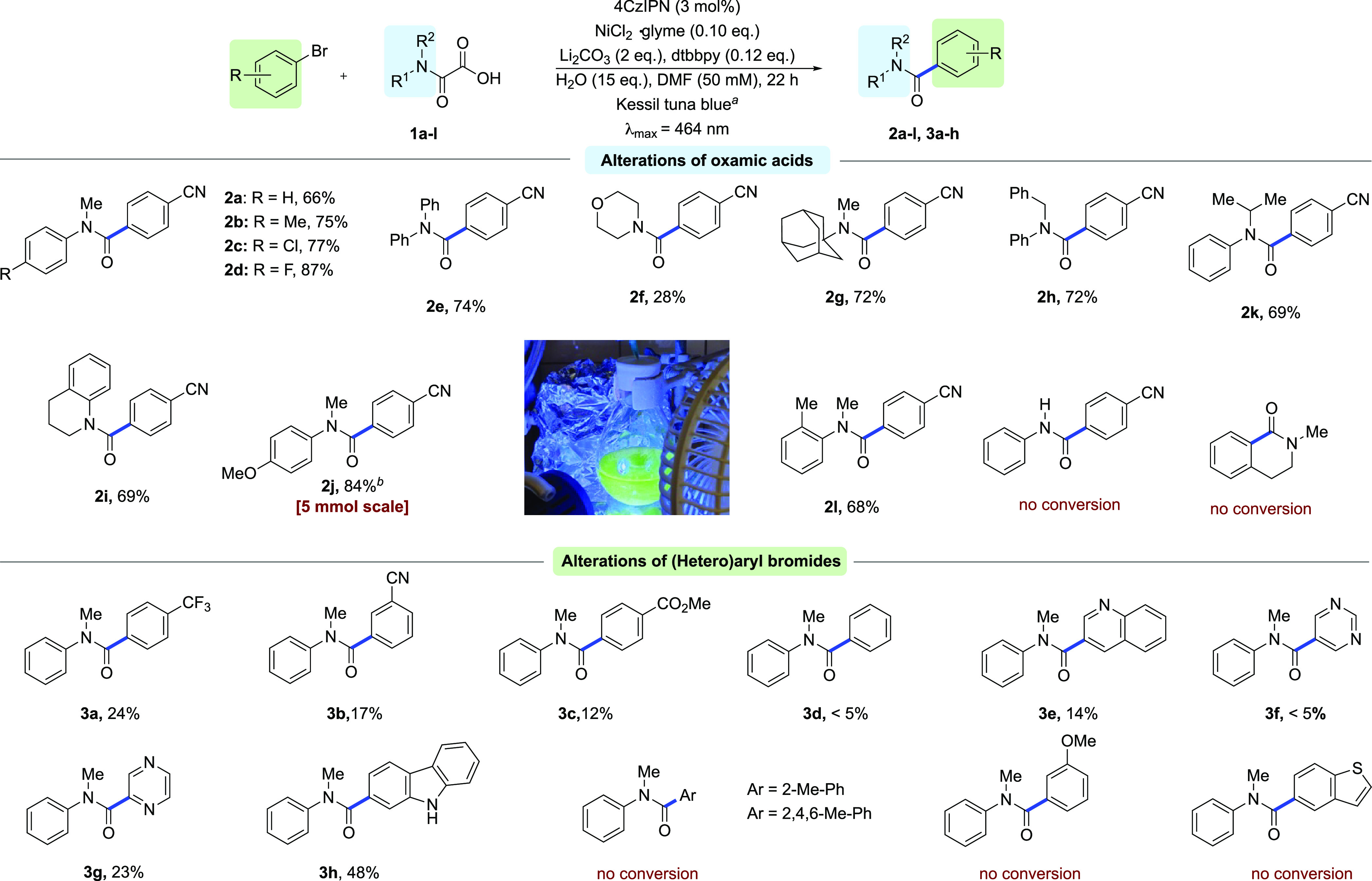
Substrate Scope Reaction conditions: ArBr (200 μmol, 1.00 equiv), **1a**–**l** (400 μmol, 2.00 equiv), Li_2_CO_3_ (29.6 mg, 400 μmol, 2.00 equiv), 4CzIPN (4.73 mg, 6.00 μmol, 0.03 equiv), NiCl_2_·glyme (4.39 mg, 20.0 μmol, 0.10 equiv), 4,4′-dtbbpy (6.44 mg, 24.0 μmol, 0.12 equiv), DMF (4.0 mL), H_2_O (54.0 mg, 3.00 mmol, 15.0 equiv), argon, 22 h, Kessil tuna blue (λ_max_ = 464 nm), isolated yields are reported. 4-Bromobenzonitrile (910 mg, 5.00 mmol, 1.00 equiv), **1j** (2.09 g, 10.0 mmol, 2.0 equiv), 22 h.

**Scheme 3:**
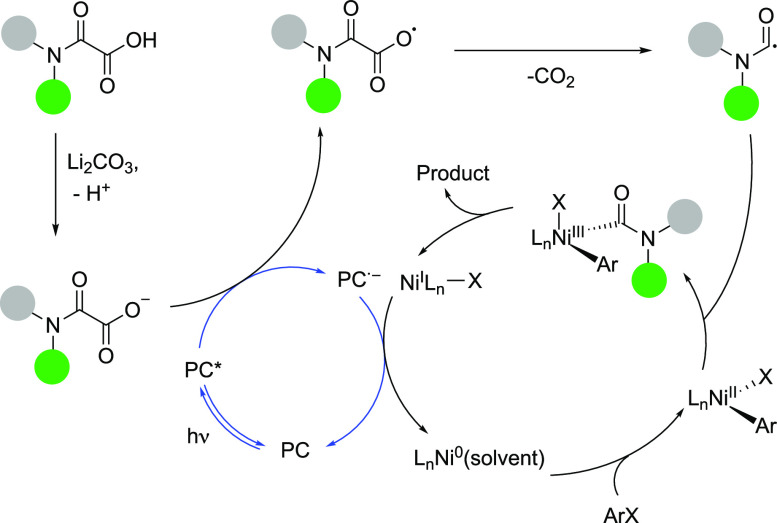
Potential Mechanism^15^

**Table 2:**
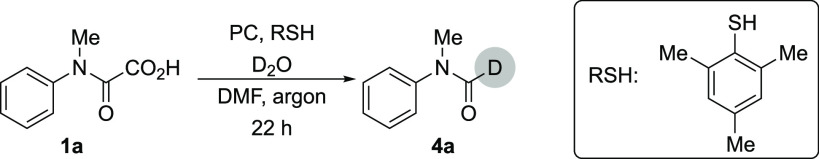
Deuterated Formamide Synthesis: Optimization^a^

Entry^b^	PC	Thiol (mol %)	D_2_O (equiv)	Yield^c^ (D incorporation)
1	[Ir]	10	28	37% (73%)
2	[Ir]	10	56	40% (87%)
3	4CzIPN	10	28	22% (43%)
**4**	**4CzIPN**	**10**	**56**	**43% (90%)**
5	4CzIPN	10	112	11% (92%)
6	4CzIPN	30	56	35% (87%)
7	4CzIPN	60	56	32% (86%)
8	–	10	56	0%
9	4CzIPN	10	–	44% (0%)
10	4CzIPN	–	56	0%

a [Ir] = Ir(dF(CF_3_)ppy)_2_(dtbpy))PF_6_.

b Reaction conditions: **1a** (17.9 mg, 100 μmol), 4CzIPN (2.37 mg, 3.00 μmol), Li_2_CO_3_ (7.39 mg, 100 μmol), DMF (2.0 mL), argon, 22 h, Kessil tuna blue (λ_max_ = 464 nm).

c Yields were determined by the ^1^H NMR analysis of the crude mixture using 1,2,4,5-tetramethylbenzene as an internal standard.

**Scheme 4:**
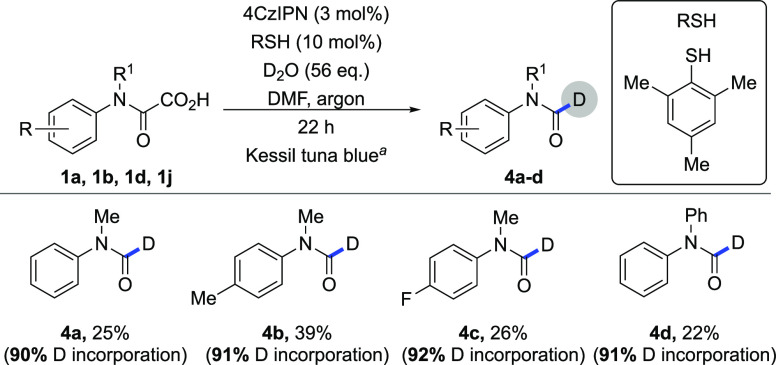
Preparation of Deuterated Formamides Reaction conditions: **1** (300 μmol), Li_2_CO_3_ (22.2 mg, 300 μmol), 2,4,6-trimethylthiophenol (4.57 mg, 3.00 μmol), D_2_O (336 μg, 16.8 mmol), DMF (6.0 mL), argon, 22 h, Kessil tuna blue (λ_max_ = 464 nm).

In conclusion, an efficient methodology toward amide synthesis relying on the photoredox- and nickel-catalyzed cross-coupling of readily available oxamic acids with aryl bromides was developed. Mild reaction conditions and the application of a broadly used organic photocatalyst (4CzIPN) make this transformation suitable as an alternative to precious metal-catalyzed aminocarbonylations. The scope and limitations were investigated by varying the oxamic acids and (hetero)aryl bromides. The procedure was successfully performed on a gram scale, and the trapping of the carbamoyl radical with a HAT catalyst in the presence of D_2_O supports the generation of carbamoyl radicals. Furthermore, a methodology that provides access to deuterated formamides was applied to different oxamic acids, yielding the desired products with a high level of D incorporation.

## Data Availability

The data underlying this study are available in the published article and its Supporting Information.

## Supporting Information Available

Experimental details and characterization data (PDF)
