# Supplementary material for: Decarboxylative Nickel- and Photoredox-Catalyzed Aminocarbonylation of (Hetero)Aryl Bromides
Source: Org Lett. 2023 Oct 5;26(14):2768–72. doi: 10.1021/acs.orglett.3c02389 (PMC11020166; doi:10.1021/acs.orglett.3c02389)
Supplement: Supplementary file 1 — ol3c02389_si_001.pdf [file ol3c02389_si_001.pdf]

**Supporting Information**

# Decarboxylative Nickel- and Photoredox-Catalyzed Aminocarbonylation of (Hetero)Aryl Bromides

Valeriia Hutskalova, Farhan Bou Hamdan,* and Christof Sparr*

V.H. and C.S.: Department of Chemistry, University of Basel

F.B.H.: Syngenta Crop Protection AG, Crop Protection Research, Stein, Switzerland

F.B.H.: E-mail: farhan.bou_hamdan@syngenta.com

C.S.: E-mail: christof.sparr@unibas.ch

## Table of Contents

1. General Information ....................1
2. Oxamic Acids Synthesis ....................2
3. Optimization Studies ....................10
4. Substrate Scope....................13
5. Synthesis of Deuterated Formamides: Optimization Studies....................21
6. NMR Data....................23
7. References ....................79

## 1. General Information

All reaction solvents and reagents were obtained from commercial suppliers and used without further purification unless stated otherwise. Solvents for extractions and chromatography were technical grade. Syringes were used to transfer air and moisture sensitive liquids and solutions. Analytical thin layer chromatography (Merck silica gel 60 F254 plates) was utilized for monitoring reactions and visualized by UV light (254 nm and 350 nm). Flash Chromatography was performed with SiliCycle silica gel 60 (230-400 Mesh) or otherwise stated stationary columns. Concentration *in vacuo* was performed by rotary evaporation to ~10 mbar at 40°C and drying at ~ $10^{-2}$ mbar at room temperature.

$^{1}$H-NMR spectra were recorded on Bruker DPX 400 MHz or Bruker DRX 500 MHz spectrometers at 298 K in the indicated deuterated solvent supplied by *Cambridge Isotope Laboratories*. Chemical shifts ($\delta$) are quoted in parts per million (ppm) and referenced to the residual solvent peak ($\delta$ = 7.26 ppm for $CDCl_3$ and 2.50 ppm for $(CD_3)_2SO$, 0.00 ppm TMS). The multiplicities are reported in Hz as: s = singlet, br = broad singlet, d = doublet, t = triplet, q = quartet, m = multiplet, dm = doublet of multiplets, and ddm = doublet of doublet of multiplets. $^{13}$C- and 2D-NMR spectra were recorded with $^{1}$H-decoupling on Bruker DRX 500 MHz spectrometers at 298 K in the indicated deuterated solvent supplied by *Cambridge Isotope Laboratories*. Chemical shifts ($\delta$) are quoted in parts per million (ppm) and referenced to the residual solvent peak ($\delta$ = 77.16 ppm for $CDCl_3$ and 39.52 ppm for $(CD_3)_2SO$).

Melting points were measured on a Büchi B-565 melting point apparatus and are uncorrected. IR spectroscopy was measured on an ATR Varian Scimitar 800 FT-IR spectrometer and reported in cm$^{-1}$. The intensities of the bands are reported as: w = weak, m = medium, s = strong. High-resolution mass spectrometry (HR-ESI) was recorded by Dr. Michael Pfeffer at the University of Basel on a *Bruker MaXis 4G QTOF* ESI mass spectrometer. The reactions were performed in a custom-built photoreactor reflecting and directing the light towards the samples leading to homogenous light exposure. The cooling was achieved utilizing a fan. The temperature inside the reactor was in the range of 30 and 32°C (monitored by an internal thermometer). Screw-top clear glass vials (4 or 7 mL) were used. The scale-up synthesis of **2j** was performed with a Kessil tuna blue LED lamp (λmax = 464 nm, 10 cm distance from the flask, 40 W, 100% intensity) and with cooling by a fan.

# 2. Oxamic Acids Synthesis

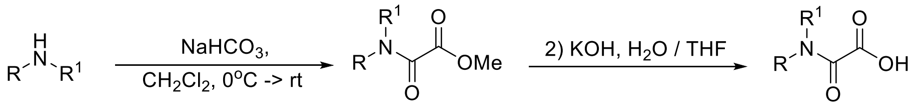


**General procedure A for the synthesis of methyl 2-oxoacetates S1-Sn (step 1)** (according to a literature procedure):[1] A mixture of the corresponding amine (1.00 eq.) and $NaHCO_3$ (1.50 eq.) in dry $CH_2Cl_2$ (1.0 $molL^{-1}$) was cooled to 0°C under argon. A solution of methyl 2-chloro-2-oxoacetate (1.30 eq.) in dry $CH_2Cl_2$ (1.0 $molL^{-1}$) was added dropwise to the mixture. The resulting reaction mixture was then allowed to warm up to room temperature and was left stirring for 18 h. After the reaction completion, EtOAc and $H_2O$ was added. The aqueous layer was then extracted with EtOAc twice. The combined organic phase was dried over $Na_2SO_4$ and the solvent was removed. The residue was then purified with silica gel chromatography (from cyclohexane/EtOAc 1:0 to 3:1) yielding the title compound.

**General procedure B for the synthesis of oxamic acids 1a-1n (step 2)** (according to a modified literature procedure):[1] The corresponding methyl 2-oxoacetate (1.00 eq.) was dissolved in THF (0.13 $molL^{-1}$), then an aqueous solution of KOH (1.50 eq., 2.0 $molL^{-1}$) was added dropwise. The resulting reaction mixture was stirred for 18 h and then diluted with EtOAc and $H_2O$. After phase separation, the **aqueous** phase was acidified with HCl (1.0 $molL^{-1}$) to pH=1 and extracted with EtOAc (×3). The combined organic phase was dried over $Na_2SO_4$, and the solvent was removed yielding the desired oxamic acid.

### Methyl 2-(methyl(phenyl)amino)-2-oxoacetate S1a:

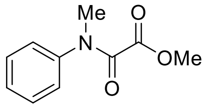


Prepared according to the general procedure **A** using *N*-methylaniline (3.23 g, 30.0 mmol), methyl 2-chloro-2-oxoacetate (4.78g, 39.0 mmol), and $NaHCO_3$ (3.78 g, 45.0 mmol) to give the title compound as colorless oil (5.60 g, 29.0 mmol, 97%). NMR corresponds to literature reports:[2] $^{1}$H NMR (500 MHz, $CDCl_3$) δ 7.42 – 7.37 (2H, m, 3*H*, C5*H*), 7.37 – 7.32 (1H, m, C4*H*), 7.25 – 7.20 (2H, m, C2*H*, C6*H*), 3.56 (3H, s, OC$H_3$), 3.36 (3H, s, N–C$H_3$); $^{13}$C NMR (126 MHz, $CDCl_3$) δ = 162.9 (*C*$O_2$Me), 161.6 (N(Me)*C*=O), 141.5 (*C1*), 129.6 (*C3*, *C5*), 128.4 (*C4*), 126.2 (*C2*, *C6*), 52.2 (O*C*$H_3$), 36.1 (N–*C*$H_3$).

### 2-(Methyl(phenyl)amino)-2-oxoacetic acid 1a:

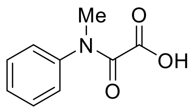


Prepared according to the general procedure **B** using methyl 2-(methyl(phenyl)amino)-2-oxoacetate **S1a** (1.35 g, 7.00 mmol) and KOH (589 mg, 10.5 mmol) to give the title compound as a white solid (1.12 g, 6.25 mmol, 89%). NMR corresponds to literature reports:[1] $^{1}$H NMR (500 MHz, DMSO) δ = 13.88 (C$O_2$*H*), 7.45 (2H, dd, $^{3}J$ 8.4, 7.0 Hz, C3*H*, C5*H*), 7.39 – 7.35 (1H, m, C4*H*), 7.36 – 7.30 (2H, m, C2*H*, C6*H*), 3.24 (3H, s, N–C$H_3$); $^{13}$C NMR (126 MHz, DMSO) δ = 164.7 (*C*$O_2$H), 163.2 (N(Me)*C*=O), 141.9 (*C1*), 129.9 (*C3*, *C5*), 128.4 (*C4*), 126.7 (*C2*, *C6*), 35.7 (N–*C*$H_3$).

### Methyl 2-(methyl(p-tolyl)amino)-2-oxoacetate S1b:

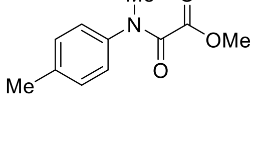


Prepared according to the general procedure **A** using *N*,4-dimethylaniline (1.82 g, 15.0 mmol), methyl 2-chloro-2-oxoacetate (2.39 g, 19.5 mmol), and $NaHCO_3$ (1.89 g, 22.5 mmol) to give the title

compound as a white solid (2.98 g, 14.4 mmol, 96%): $^1$H NMR (500 MHz, $CDCl_3$): δ = 7.20 – 7.15 (2H, m, C3*H*, C5*H*), 7.12 – 7.08 (2H, m, C2*H*, C6*H*), 3.58 (3H, s, OC$H_3$), 3.33 (3H, s, N–C$H_3$), 2.36 (3H, s, C4–C$H_3$); $^{13}$C NMR (126 MHz, $CDCl_3$) δ = 163.1 (*C*$O_2$Me) , 161.7 (N(Me)*C*=O), 138.8 (*C1*), 138.4 (*C4*), 130.2 (*C3, C5*), 126.1 (*C2*, *C6*), 52.2 (O*C*$H_3$), 36.2 (N–*C*$H_3$), 21.1 (C4–*C*$H_3$).

### 2-(Methyl(*p*-tolyl)amino)-2-oxoacetic acid 1b:

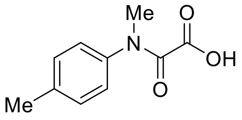


Prepared according to the general procedure **B** using methyl 2-(methyl(p-tolyl)amino)-2-oxoacetate **S1b** (1.45 g, 7.00 mmol) and KOH (589 mg, 10.5 mmol) to give the title compound as a white solid (1.14 g, 5.90 mmol, 84%). NMR corresponds to literature reports:[1] $^1$H NMR (500 MHz, DMSO) δ = 13.78 ($CO_2$*H*), 7.26 – 7.22 (2H, m, C3*H*, C5*H*), 7.22 – 7.18 (2H, m, C2*H*, C6*H*), 3.21 (3H, s, N–C$H_3$), 2.31 (3H, s, C4–C$H_3$); $^{13}$C NMR (126 MHz, DMSO) δ = 164.8 (*C*$O_2$H), 163.2 (N(Me)*C*=O), 139.3 (*C1*), 137.9 (*C4*), 130.3 (*C3*, *C5*), 126.7 (*C2*, *C6*), 35.7 (N–*C*$H_3$), 21.0 (C4–*C*$H_3$).

### Methyl 2-((4-chlorophenyl)(methyl)amino)-2-oxoacetate S1c:

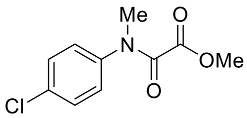


Prepared according to the general procedure **A** using 4-chloro-*N*-methylaniline (2.12 g, 15.0 mmol), methyl 2-chloro-2-oxoacetate (2.39 g, 19.5 mmol), and $NaHCO_3$ (1.89 g, 22.5 mmol) to give the title compound as a white solid (2.86 g, 12.6 mmol, 84%). NMR corresponds to literature reports:[2] $^1$H NMR (500 MHz, $CDCl_3$) δ = 7.37 (2H, d, $^3J$ 8.4 Hz, C3*H*, C5*H*), 7.18 (2H, d, $^3J$ 8.4 Hz, C2*H*, C6*H*), 3.61 (3H, s, OC$H_3$), 3.34 (3H, s, N–C$H_3$); $^{13}$C NMR (126 MHz, $CDCl_3$) δ = 162.7 (*C*$O_2$Me), 161.3 (N(Me)*C*=O), 139.9 (*C1*), 134.3 (*C4*), 129.9 (*C3*, *C5*), 127.6 (*C2*, *C6*), 52.4 (O*C*$H_3$), 36.2 (N–*C*$H_3$).

### 2-((4-Chlorophenyl)(methyl)amino)-2-oxoacetic acid 1c:

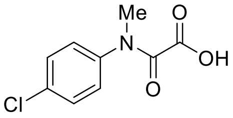


Prepared according to the general procedure **B** using methyl 2-((4-chlorophenyl)(methyl)amino)-2-oxoacetate **S1c** (1.59 g, 7.00 mmol) and KOH (589 mg, 10.5 mmol) to give the title compound as a white solid (1.15 g, 5.38 mmol, 77%). NMR corresponds to literature reports:[1] $^1$H NMR (500 MHz, DMSO) δ = 14.01 ($CO_2$*H*), 7.52 (2H, d, $^3J$ 8.4 Hz, C3*H*, C5*H*), 7.37 (2H, d, $^3J$ 8.3 Hz, C2*H*, C6*H*), 3.23 (3H, s, N–C$H_3$); $^{13}$C NMR (126 MHz, DMSO) δ = 164.6 (*C*$O_2$H), 162.9 (N(Me)*C*=O), 140.8 (*C1*), 132.9 (*C4*), 129.9 (*C3*, *C5*), 128.7 (*C2*, *C6*), 35.6 (N–*C*$H_3$).

### Methyl 2-((4-fluorophenyl)(methyl)amino)-2-oxoacetate S1d:

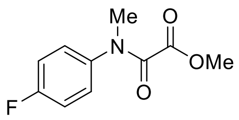


Prepared according to the general procedure **A** using 4-fluoro-*N*-methylaniline (1.88 g, 15.0 mmol), methyl 2-chloro-2-oxoacetate (2.39 g, 19.5 mmol), and $NaHCO_3$ (1.89 g, 22.5 mmol) to give the title compound as a white solid (3.10 g, 14.7 mmol, 98%). NMR corresponds to literature reports:[2] $^1$H NMR (500 MHz, $CDCl_3$) δ = 7.25 – 7.17 (2H, m, C2*H*, C6*H*), 7.13 – 7.02 (2H, m, C3*H*, C5*H*), 3.58 (3H, s, OC$H_3$), 3.33 (3H, s, N–C$H_3$); $^{13}$C NMR (126 MHz, $CDCl_3$) δ = 162.8 (*C*$O_2$Me), 162.1 (d, $^1J_{CF}$ 249.1 Hz, *C4*), 161.5 (N(Me)*C*=O), 137.4 (d, $^4J_{CF}$ 3.2 Hz, *C1*), 128.4 (d, $^3J_{CF}$ 8.8 Hz, *C2*, *C6*), 116.6 (d, $^2J_{CF}$ 22.9 Hz, *C3*, *C5*), 52.3 (O*C*$H_3$), 36.3 (N–*C*$H_3$).

### 2-((4-Fluorophenyl)(methyl)amino)-2-oxoacetic acid 1d:

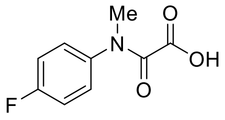

Prepared according to the general procedure **B** using methyl 2-((4-fluorophenyl)(methyl)amino)-2-oxoacetate **S1d** (1.48 g, 7.00 mmol) and KOH (589 mg, 10.5 mmol) to give the title compound as a white solid (1.08 g, 5.48 mmol, 78%). NMR corresponds to literature reports:[1] $^{1}$H NMR (500 MHz, DMSO) δ = 13.92 (1H, s, $CO_2$*H*), 7.46 – 7.34 (2H, m, C2*H*, C6*H*), 7.33 – 7.20 (2H, m, C3*H*, C5*H*), 3.22 (3H, s, N–*C*$H_3$); $^{13}$C NMR (126 MHz, DMSO) δ = 164.7 (*C*$O_2$H), 163.1 (N(Me)*C*=O), 161.70 (d, $^{1}J_{CF}$ 245.3 Hz, *C4*), 138.2 (d, $^{4}J_{CF}$ 2.9 Hz, *C1*), 129.3 (d, $^{3}J_{CF}$ 9.0 Hz, *C2*, *C6*), 116.7 (d, $^{2}J_{CF}$ 22.9 Hz, *C3*, *C5*), 35.8 (N–*C*$H_3$).

### Methyl 2-(diphenylamino)-2-oxoacetate S1e:

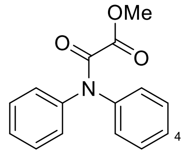

Prepared according to the general procedure **A** using *N*-phenylaniline (2.54 g, 15.0 mmol), methyl 2-chloro-2-oxoacetate (2.39 g, 19.5 mmol), and $NaHCO_3$ (1.89 g, 22.5 mmol) to give the title compound as a white solid (3.66 g 14.3 mmol, 96%): NMR corresponds to literature reports:[2] $^{1}$H NMR (500 MHz, $CDCl_3$) δ = 7.44 – 7.33 (5H, m, C2*H*, C3*H*, C4*H*, C5*H*, C6*H)*, 7.32 – 7.26 (5H, m, C2*H*, C3*H*, C4*H*, C5*H*, C6*H*), 3.61 (3H, s, OC*H*$_3$); $^{13}$C NMR (126 MHz, $CDCl_3$) δ = 162.9 (*C*$O_2$Me), 161.0 (N*C*=O), 140.5, 140.4 (*C1*), 129.7 (*C2*/*C6*), 129.2 (*C3*/*C5*), 128.6 (*C4*), 127.8 (*C2*/*C6*), 127.1 (*C4*), 125.84(C3/C5), 52.4 (O*C*$H_3$).

### 2-(Diphenylamino)-2-oxoacetic acid 1e:

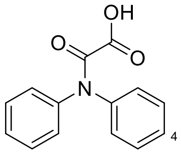

Prepared according to the general procedure **B** using methyl 2-(diphenylamino)-2-oxoacetate **S1e** (1.78 g, 7.00 mmol) and KOH (589 mg, 10.5 mmol) to give the title compound as a white solid (1.06 g, 4.41 mmol, 63%): NMR corresponds to literature reports:[2] $^{1}$H NMR (500 MHz, DMSO) δ = 14.15 (1H, s, $CO_2$*H*), 7.60 – 7.17 (5H, m, C2*H*, C3*H*, C4*H*, C5*H*, C6*H*); $^{13}$C NMR (126 MHz, DMSO) δ = 164.4 (*C*$O_2$H), 162.8 (N*C*=O), 140.98, 140.93 (C1), 130.1, 129.7 (*C3*/*C5*), 128.9, 128.5 (*C2*/*C6*), 127.6, 126.8 (*C4*).

### Methyl 2-morpholino-2-oxoacetate S1f:

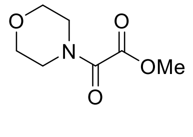

Prepared according to the general procedure **A** using morpholine (958 mg, 11.0 mmol), methyl 2-chloro-2-oxoacetate (1.75 g, 14.3 mmol), and $NaHCO_3$ (1.39 g, 16.5 mmol) to give the title compound as a white solid (1.50 g, 8.66 mmol, 79%). NMR corresponds to literature reports:[3] $^{1}$H NMR (500 MHz, $CDCl_3$) δ = 3.88 (3H, s, OC*H*$_3$), 3.76 – 3.69 (4H, m, OC*H*$_2$), 3.69 – 3.63 (2H, m, NC*H*$_2$), 3.54 – 3.45 (2H, m, NC*H*$_2$); $^{13}$C NMR (126 MHz, $CDCl_3$) δ = 162.7 (*C*$O_2$Me), 159.8 (N*C*=O), 66.7 (O*C*$H_2$), 66.4 (O*C*$H_2$), 52.8 (O*C*$H_3$), 46.5 (N*C*$H_2$), 41.9 (N*C*$H_2$).

### 2-Morpholino-2-oxoacetic acid 1f:

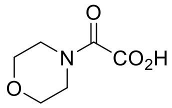

Prepared according to the general procedure **B** using methyl 2-morpholino-2-oxoacetate **S1f** (1.49 g, 8.60 mmol) and KOH (724 mg, 12.9 mmol) to give the title compound as a white solid (874 mg,

5.49 mmol, 64%): $^{1}$H NMR (500 MHz, DMSO) δ = 3.64 – 3.55 (4H, 2 × OC*H*$_2$), 3.51 – 3.44 (2H, m, NC*H*$_2$), 3.41 – 3.34 (2H, m, NC*H*$_2$); $^{13}$C NMR (126 MHz, DMSO) δ = 164.9 (*C*O$_2$H), 161.7, 161.4 (N*C*=O), 66.6 (O*C*H$_2$), 66.2 (O*C*H$_2$), 46.3 (N*C*H$_2$), 41.2 (N*C*H$_2$).

**Methyl 2-(((3s,5s,7s)-adamantan-1-yl)(methyl)amino)-2-oxoacetate S1g:**

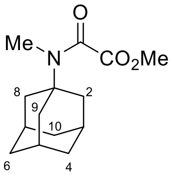


Prepared according to the general procedure **A** using *N*-methyl-1-adamantylamin (397 mg, 2.40 mmol), methyl 2-chloro-2-oxoacetate (382 mg, 3.12 mmol), and NaHCO$_3$ (302 mg, 3.60 mmol) to give the title compound as a white solid (200 mg 796 µmol, 33%, m.p. 107.0 – 107.5°C): R$_f$ 0.56 (cyclohexane/EtOAc 2:1); $\nu_{max}$ (neat): 2969w, 2907m, 2847m, 1733s, 1634s, 1495w, 1452w, 1403m, 1295m, 1248s, 1196m, 1094s, 898w, 827w, 674w cm$^{-1}$; $^{1}$H NMR (500 MHz, CDCl$_3$) δ = 3.84 (3H, s, OC*H*$_3$), 2.84 (3H, s, N–C*H*$_3$), 2.20 (6H, s, C2*H*$_2$, C8*H*$_2$, C9*H*$_2$,), 2.13 (3H, s, C3*H*$_2$, C5*H*$_2$, C7*H*$_2$,), 1.81 – 1.60 (6H, m, C4*H*$_2$, C6*H*$_2$, C10*H*$_2$,); $^{13}$C NMR (126 MHz, CDCl$_3$) δ = 163.8 (*C*O$_2$Me), 162.8 (N*C*=O), 58.8 (*C1*), 52.3 (O*C*H$_3$), 39.0 (*C2*, *C8*, *C9*), 36.3 (*C4*, *C6*, *C10*), 31.6 (N–*C*H$_3$), 29.9 (*C3*, *C5*, *C7*); HMRS (ESI) m/z: [M+H$^+$] Calcd. for $C_{14}H_{22}NO_3$ 252.1594; Found 252.1593.

**2-(((3s,5s,7s)-Adamantan-1-yl)(methyl)amino)-2-oxoacetic acid 1g:**

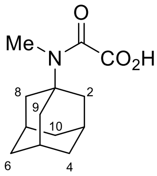


Prepared according to the general procedure **B** using methyl 2-(((3s,5s,7s)-adamantan-1-yl)(methyl)amino)-2-oxoacetate **S1g** (170 mg, 676 µmol) and KOH (56.9 mg, 1.01 mmol) to give the title compound as a white solid (115 mg, 485 µmol, 72%, m.p. 186.0 – 186.4°C): $\nu_{max}$ (neat): 2974w, 2909m, 2847w, 1740m, 1589s, 1498w, 1453w, 1390w, 1244m, 194m, 1093m, 1052s, 981m, 901w cm$^{-1}$; $^{1}$H NMR (500 MHz, DMSO) δ = 2.82 (3H, s, NC*H*$_3$), 2.14 (6H, s, C2*H*$_2$, C8*H*$_2$, C9*H*$_2$), 2.06 (3H, s, C3*H*, C5*H*, C7*H*), 1.73 – 1.57 (6H, m, C4`*H*$_2$, C6`*H*$_2$, C10`*H*$_2$); $^{13}$C NMR (126 MHz, DMSO) δ = 165.4 (*C*O$_2$H), 164.5 (N(Me)*C*=O), 57.8 (*C1*), 38.8 (*C2*, *C8*, *C9*), 36.3 (*C4*, *C6*, *C10*), 31.7 (N–*C*H$_3$), 29.7 (*C3*, *C5*, *C7*); HMRS (ESI) m/z: [M+Na$^+$] Calcd. for $C_{13}H_{19}NNaO_3$ 260.1257; Found 260.1256 [M+Na$^+$].

**Methyl 2-(benzyl(phenyl)amino)-2-oxoacetate S1h:**

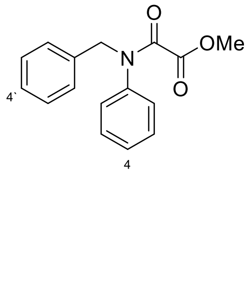


Prepared according to the general procedure **A** using *N*-benzylaniline (1.37 g, 7.50 mmol), methyl 2-chloro-2-oxoacetate (1.19 g, 9.75 mmol), and NaHCO$_3$ (945 mg, 11.3 mmol) to give the title compound as a white solid (1.70 g, 6.31 mmol, 84%). NMR corresponds to literature reports:[4] $^{1}$H NMR (500 MHz, CDCl$_3$) δ = 7.32 – 7.26 (6H, m, C3`*H*, C5`*H*, C3*H*, C5*H*, C4*H*, C4`*H*), 7.24 – 7.16 (2H, m, C2`*H*, C6`*H*), 7.08 – 7.03 (2H, m, C2*H*, C6*H*), 4.95 (2H, s, C*H*$_2$), 3.54 (3H, s, OC*H*$_3$); $^{13}$C NMR (126 MHz, CDCl$_3$) δ = 163.0 (*C*O$_2$Me), 161.8 (N*C*=O), 139.8 (*C1*), 136,0 (*C1`*), 129.5 (*C3*, *C5*), 128.9 (*C3`*, *C5`*), 128.74 (*C2`*, *C6`*), 128.72 (*C4*), 127.9 (*C4`*), 127.8 (*C2*, *C6*), 52.37 (*C*H$_2$), 52.4 (O*C*H$_3$).

**2-(Benzyl(phenyl)amino)-2-oxoacetic acid 1h:**

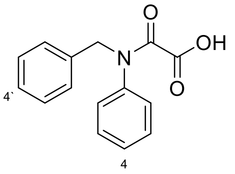

Prepared according to the general procedure **B** using methyl 2-(benzyl(phenyl)amino)-2-oxoacetate **S1h** (1.63 g, 6.05 mmol) and KOH (509 mg, 9.08 mmol) to give the title compound as a white solid (1.23 g, 4.82 mmol, 80%). NMR corresponds to literature reports:[4] $^{1}$H NMR (500 MHz, DMSO) δ = 13.99 (1H, s, $CO_2$*H*), 7.39 – 7.34 (2H, m, C3*H*, C5*H*), 7.34 – 7.29 (3H, m, C3\`*H*, C5\`*H*, C4*H*), 7.28 – 7.23 (1H, m, C4\`*H*), 7.21 – 7.17 (4H, m, C2*H*, C6*H*, C2\`*H*, C6\`*H*), 4.95 (2H, s, C*H*$_2$); $^{13}$C NMR (126 MHz, DMSO) δ = 164.6 (*C*$O_2$H), 163.5 (N*C*=O), 140.2 (*C1*), 136.8 (*C1\`*), 129.8 (*C3*, *C5*), 128.9 (*C3\`*, *C5\`*), 128.6 (*C4*), 128.4 (*C2\`*, *C6\`*), 127.9 (*C4\`*), 127.7 (*C2*, *C6*), 51.1 (*C*$H_2$).

**Methyl 2-(3,4-dihydroquinolin-1(2*H*)-yl)-2-oxoacetate S1i:**

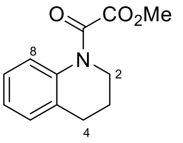

Prepared according to the general procedure **A** using 1,2,3,4-tetrahydroquinoline (1.99 g, 15.0 mmol), methyl 2-chloro-2-oxoacetate (2.39 g, 19.5 mmol), and $NaHCO_3$ (1.89 g, 22.5 mmol) to give the title compound as a white solid (2.97 g, 13.5 mmol, 90%, m.p. 97.9 – 98.5°C): $R_f$ 0.39 (cyclohexane/EtOAc 7:3) $\nu_{max}$ (neat): 2922w, 2962w, 2877w, 2855w, 1731m, 1650s, 1581w, 1494m, 1457m, 1405m, 1224s, 1200s, 1161s, 1072w, 1038w, 980w, 918w, 870w, 853w, 763s, 710m, 674w cm$^{-1}$; $^{1}$H NMR (500 MHz, $CDCl_3$) δ = 7.23 – 7.17 (1H, m, C5*H*), 7.16 – 7.10 (2H, m, C6*H*, C7*H*), 7.00 (1H, d, $^{3}J$ 7.2 Hz, C8*H*), 3.85 (2H, t, $^{3}J$ 6.4 Hz, C2*H*$_2$), 3.71 (3H, s, OC*H*$_3$), 2.80 (2H, t, $^{3}J$ 6.3 Hz, C4*H*$_2$), 2.16 – 1.92 (2H, m, C3*H*$_2$); $^{13}$C NMR (126 MHz, $CDCl_3$) δ = 163.5 (*C*$O_2$Me), 161.1 (N*C*=O), 136.9 (*C8a*), 132.4 (*C4a*), 128.9 (*C5*), 126.7 (*C7*), 126.3 (*C6*), 121.2 (*C8*), 52.5 (O*C*$H_3$), 42.5 (*C2*), 26.7 (*C4*), 23.2 (*C3*); HMRS (ESI) m/z: [M+H$^+$] Calcd. for $C_{12}H_{14}NO_3$ 220.0968; Found 220.0965 [M+H$^+$].

**2-(3,4-Dihydroquinolin-1(2*H*)-yl)-2-oxoacetic acid 1i:**

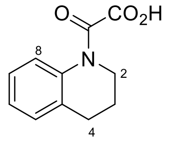

Prepared according to the general procedure **B** using methyl 2-(3,4-dihydroquinolin-1(2*H*)-yl)-2-oxoacetate **S1i** (1.54 g, 7.00 mmol) and KOH (589 mg, 10.5 mmol) to give the title compound as a white solid (1.18 g, 5.76 mmol, 82%). NMR corresponds to literature reports:[5] $^{1}$H NMR (500 MHz, DMSO) δ = 14.26 (1H, s, $CO_2$*H*), 7.43 – 6.80 (4H, m, C5*H*, C6*H*, C7*H*, C8*H*), 3.83 – 3.60 (2H, m, C2*H*$_2$), 2.95 – 2.68 (2H, C4*H*$_2$), 2.08 – 1.74 (2H, m, C3*H*$_2$); $^{13}$C NMR (126 MHz, DMSO) δ = 165.2 (*C*$O_2$H), 162.8 (N*C*=O), 137.3 (*C8a*), 132.4 (*C4a*), 129.4 (*C5*), 127.0 (*C7*), 126.3 (*C6*), 121.5 (*C8*), 42.2 (*C2*), 26.6 (*C4*), 23.2 (*C3*).

**Methyl 2-((4-methoxyphenyl)(methyl)amino)-2-oxoacetate S1j:**

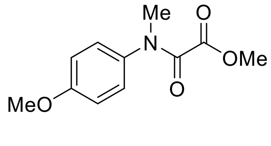

Prepared according to the general procedure **A** using 4-methoxy-*N*-methylaniline (2.47 g, 18.0 mmol), methyl 2-chloro-2-oxoacetate (2.87 g, 23.4 mmol), and $NaHCO_3$ (2.27 g, 27.0 mmol) to give the title compound as a white solid (3.77 g, 16.9 mmol, 94%). NMR corresponds to literature reports:[2] $^{1}$H NMR (500 MHz, $CDCl_3$) δ = 7.16 (2H, d, $^{3}J$ 7.2 Hz, C3*H*, C5*H*), 6.88 (2H, d, $^{3}J$ 7.3 Hz, C2*H*, C6*H*), 3.82 (3H, s, C4–OC*H*$_3$), 3.57 (3H, s, C(=O)OC*H*$_3$), 3.31 (3H, s, N–C*H*$_3$);

$^{13}$C NMR (126 MHz, $CDCl_3$) δ = 163.2 (*C*$O_2$Me), 161.8 (N(Me)*C*=O), 159 (*C4*)., 134.0 (*C1*), 127.9 (*C2*, *C6*), 114.7 (*C3*, *C5*), 55.5 (C4–O*C*$H_3$), 52.2 (C(=O)O*C*$H_3$), 36.3 (N–*C*$H_3$).

## 2-((4-Methoxyphenyl)(methyl)amino)-2-oxoacetic acid 1j:

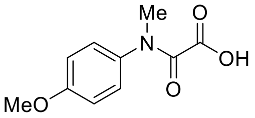

Prepared according to the general procedure **B** using methyl 2-((4-methoxyphenyl)(methyl)amino)-2-oxoacetate (3.57 g, 16.0 mmol) and KOH (1.35 g, 24.0 mmol) to give the title compound as a white solid (2.92 g, 13.9 mmol, 87%): $^{1}$H NMR (500 MHz, DMSO) δ = 7.26 (2H, d, $^{3}J$ 8.9 Hz, C2*H*, C6*H*), 6.97 (2H, d, $^{3}J$ 8.9 Hz, C3*H*, C5*H*), 3.77 (3H, s, OC*H*$_3$), 3.18 (3H, s, N–C*H*$_3$); $^{13}$C NMR (126 MHz, DMSO) δ = 164.9 (*C*$O_2$H), 163.4 (N(Me)*C*=O), 159.2 (*C4*), 134.4 (*C1*), 128.5 (*C2*, *C6*), 114.9 (*C3*, *C5*), 55.9 (O*C*$H_3$), 35.8 (N–*C*$H_3$).

## Methyl 2-(isopropyl(phenyl)amino)-2-oxoacetate S1k:

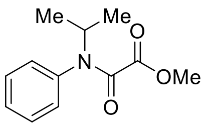

Prepared according to the general procedure **A** using *N*-isopropylaniline (2.03 g, 15.0 mmol), methyl 2-chloro-2-oxoacetate (2.39 g, 19.5 mmol), and $NaHCO_3$ (1.89 g, 22.5 mmol) to give the title compound as a white solid (2.80 g,12.7 mmol, 84%, m.p. 63.6 – 64.0°C): $\nu_{max}$ (neat): 2910w, 2850w, 1739m, 1588s, 1491w, 1452w, 1357w, 1221w, 1115w, 1052w, 980w, 802w, 663m cm$^{-1}$; $^{1}$H NMR (500 MHz, $CDCl_3$) δ = 7.42-7.36 (3H, m, C3*H*, C4*H*, C5*H*), 7.22 – 7.16 (2H, m, C2*H*, C6*H*), 4.89 (1H, hept, $^{3}J$ 6.6 Hz, C*H*($CH_3$)$_2$), 3.46 (3H, s, OC*H*$_3$), 1.16 (6H, d, $^{3}J$ 6.6 Hz, CH(C*H*$_3$)$_2$); $^{13}$C NMR (126 MHz, $CDCl_3$) δ = 163.0 (*C*$O_2$Me), 161.5 (N(*i*Pr)*C*=O), 136.0 (*C1*), 130.5 (*C2*, *C6*), 129.04 (*C4*), 128.97 (*C3*, *C5*), 51.9 (O*C*$H_3$), 46.9 (*C*H($CH_3$)$_2$), 20.6 (CH(*C*$H_3$)$_2$); HMRS (ESI) m/z: [M+H$^+$] Calcd. for $C_{12}H_{16}NO_3$ 222.1125; Found 222.1125.

## 2-(Isopropyl(phenyl)amino)-2-oxoacetic acid 1k:

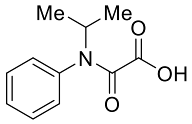

Prepared according to the general procedure **B** using methyl 2-(isopropyl(phenyl)amino)-2-oxoacetate **S1k** (2.66 g, 12.0 mmol) and KOH (1.01 g, 18.0 mmol) to give the title compound as a white solid (2.00 g, 9.65 mmol, 80%, m.p. 120.1 – 120.9°C): $\nu_{max}$ (neat): 2977w, 2929w, 2877w, 1747m, 1732m, 1611m, 1587m, 1496m, 1455m, 1390w, 1204s, 1111m, 940w, 860w, 766w, 696m cm$^{-1}$; $^{1}$H NMR (500 MHz, DMSO) δ = 7.50 – 7.38 (3H, m, C3*H*, C4*H*, C5*H*), 7.29 – 7.19 (2H, m, C2*H*, C6*H*), 4.69 (1H, hept, $^{3}J$ 6.8 Hz, C*H*($CH_3$)$_2$), 1.07 (6H, d, $^{3}J$ 6.8 Hz, CH(C*H*$_3$)$_2$); $^{13}$C NMR (126 MHz, DMSO) δ = 164.6 (*C*$O_2$H), 162.9 (N(*i*Pr)*C*=O), 136.40 (*C1*) 131.1 (*C2*, *C6*), 129.4 (*C3*, *C5*), 46.2 (*C*H($CH_3$)$_2$), 20.8 (CH(*C*$H_3$)$_2$); HMRS (ESI) m/z: [M+H$^+$] Calcd. for $C_{11}H_{14}NO_3$ 208.0968; Found 208.0966.

## Methyl 2-(methyl(*o*-tolyl)amino)-2-oxoacetate S1l:

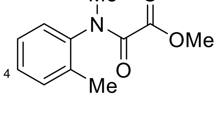

Prepared according to the general procedure **A** using *N*,2-dimethylaniline (606 mg, 5.00 mmol), methyl 2-chloro-2-oxoacetate (796 mg, 6.50 mmol), and $NaHCO_3$ (630 mg, 7.50 mmol) to give the title compound as a white solid (994 mg, 4.80 mmol, 96%): $^{1}$H NMR (500 MHz, $CDCl_3$) δ = 7.31 – 7.26 (2H, m, C3*H*, C4*H*), 7.23 – 7.17 (1H, m, C5*H*), 7.13 (1H, d, $^{3}J$ 7.6 Hz, C6*H*), 3.51 (3H, s, OC*H*$_3$),

3.25 (3H, s, N–C$H_3$), 2.31 (3H, s, C2–C$H_3$); $^{13}$C NMR (126 MHz, $CDCl_3$) δ = 162.7 (*C*$O_2$Me), 161.8 (N(Me)*C*=O), 139.7 (*C1*), 136.5 (*C2*), 131.4 (*C3*), 129.3 (*C4*), 128.3 (*C6*), 127.0 (*C5*), 52.1 (O*C*$H_3$), 35.2 (N–*C*$H_3$), 17.4 (C2–*C*$H_3$).

**2-(Methyl(*o*-tolyl)amino)-2-oxoacetic acid 1l:**

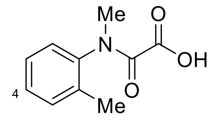

Prepared according to the general procedure **B** using methyl 2-(methyl(*o*-tolyl)amino)-2-oxoacetate **S1l** (995 mg, 4.80 mmol) and KOH (404 mg, 7.20 mmol) to give the title compound as a white solid (862 mg, 4.46 mmol, 93%): $^1$H NMR (500 MHz, DMSO) δ = 13.7 (1H, s, $CO_2$*H*), 7.38 – 7.28 (2H, m, C3*H*, C4*H*), 7.28 – 7.19 (2H, m, C5*H*, C6*H*), 3.12 (3H, s, N–C$H_3$), 2.25 (3H, s, C2–C$H_3$); $^{13}$C NMR (126 MHz, DMSO) δ = 164.7 (*C*$O_2$H), 163.3 (N(Me)*C*=O), 140.2 (*C1*), 136.8 (*C2*), 131.6 (*C3*), 129.5 (*C4*), 129.3 (*C6*), 127.4 (*C5*), 34.8 (N–*C*$H_3$), 17.5 (C2–*C*$H_3$).

**Methyl 2-oxo-2-(phenylamino)acetate S1m:**

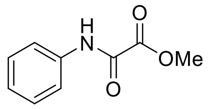

Prepared according to the general procedure **A** using aniline (1.39 g, 15.0 mmol), methyl 2-chloro-2-oxoacetate (2.39 g, 19.5 mmol), and $NaHCO_3$ (1.89 g, 22.5 mmol) to give the title compound as a beige solid (2.58 g 14.4 mmol, 96%). NMR corresponds to literature reports:[3] $^1$H NMR (500 MHz, $CDCl_3$) δ = 8.83 (1H, s, N*H*), 7.64 (2H, d, $^3J$ 8.1 Hz, C2*H*, C6*H*), 7.39 (2H, t, $^3J$ 7.9 Hz, C3*H*, C5*H*), 7.20 (1H, t, $^3J$ 7.4 Hz, C4*H*), 3.98 (3H, s, OC$H_3$); $^{13}$C NMR (126 MHz, $CDCl_3$) δ = 161.5 (*C*$O_2$Me), 153.5 (N*C*=O), 136.2 (*C1*), 129.3 (*C3*, *C5*), 125.6 (*C4*), 119.9 (*C2*, *C6*), 54.1 (O*C*$H_3$).

**2-Oxo-2-(phenylamino)acetic acid 1m:**

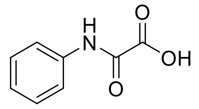

Prepared according to the general procedure **B** using methyl 2-oxo-2-(phenylamino)acetate **S1m** (717 mg, 4.00 mmol) and KOH (337 mg, 6.00 mmol) to give the title compound as a beige solid (590 mg, 3.57 mmol, 89%). NMR corresponds to literature reports:[6] $^1$H NMR (500 MHz, DMSO) δ = 14.19 (1H, s, $CO_2$*H*), 10.70 (1H, s, N*H*), 7.76 (2H, dt, $^3J$ 8.8, $^4J$ 1.6 Hz, C2*H*, C6*H*), 7.45 – 7.23 (2H, m, C3*H*, C5*H*), 7.23 – 6.99 (1H, m, C4*H*).

**2-((2-Bromophenethyl)(methyl)amino)-2-oxoacetic acid 1n:**

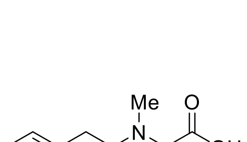

Prepared according to the general procedure **B** using methyl 2-((2-bromophenethyl)amino)-2-oxoacetate (210 mg, 700 µmol) and KOH (58.9 mg, 1.05 mmol) to give the title compound as a white solid (150 mg, 524 µmol, 75%, m.p. 63.8 – 64.5°C): $\nu_{max}$ (neat): 3433w, 2935w, 1952w, 1718m, 1615s, 1469m, 1414w, 1293w, 1243w, 1185w, 1087w, 1022w, 755m, 655w cm$^{-1}$; $^1$H NMR (500 MHz, DMSO) δ = 14.1 (1H, s, $CO_2$*H*), 7.60 (1H, dd, $^3J$ 8.0, $^4J$ 3.9 Hz, C3*H*), 7.42 – 7.28 (2H, m, C5*H*, C6*H*), 7.26 – 7.09 (1H, m, C4*H*), 3.57 – 3.44 (2H, m, NC$H_2$), 3.06 – 2.93 (2H, m, NCH$_2$C$H_2$), 2.93 (s), 2.90 (3H, s, N–C$H_3$); $^{13}$C NMR (126 MHz, DMSO) δ = 165.3 (*C*$O_2$H), 163.3, 163.2 (N*C*=O) 138.2, 137.7 (*C1*), 133.1, 133.0 (*C3*), 131.73, 131.65 (*C6*), 129.4, 129.2 (*C4*), 128.6, 128.4 (*C5*), 124.3, 124.2 (*C2*), 49.5, 45.8 (N*C*$H_2$),

35.4, 34.9 ($NCH_2CH_2$), 33.0, 31.8 ($NCH_3$); HMRS (ESI) m/z: $[M+Na^+]$ Calcd. for. $C_{11}H_{12}BrNNaO_3$ 307.9893; Found 307.9890.

**Methyl 2-(benzylamino)-2-oxoacetate S1o:**

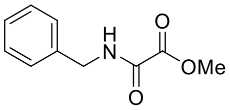

Prepared according to the general procedure **A** using phenylmethanamine (1.61 g, 15.0 mmol), methyl 2-chloro-2-oxoacetate (2.39 g, 19.5 mmol), and $NaHCO_3$ (1.89 g, 22.5 mmol) to give the title compound as a white solid (2.00 g 10.4 mmol, 69%). NMR corresponds to literature reports:[7] $^{1}$H NMR (500 MHz, $CDCl_3$) δ = 7.43 – 7.28 (5H, m, C2*H*, C3*H*, C4*H*, C5*H*, C6*H*), 4.53 (2H, d, $^{3}$J 6.1 Hz, $CH_2$), 3.91 (3H, s, $OCH_3$).

**2-(Benzylamino)-2-oxoacetic acid 1o:**

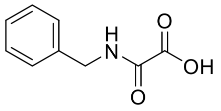

Prepared according to the general procedure **B** using methyl 2-oxo-2-(phenylamino)acetate **S1m** (2.00 g, 10.4 mmol) and KOH (875 mg, 15.6 mmol) to give the title compound as a beige solid (1.59 g, 8.87 mmol, 85%). NMR corresponds to literature reports:[5] $^{1}$H NMR (500 MHz, DMSO) δ = 13.89 (1H, s, COO*H*), 9.37 (1H, t, $^{3}J$ 6.2 Hz, N*H*), 7.48 – 6.92 (5H, m, C2*H*, C3*H*, C4*H*, C5*H*, C6*H*), 4.32 (2H, d, $^{3}J$ 6.2 Hz, $CH_2$).

**Methyl 2-(isopropylamino)-2-oxoacetate S1p:**

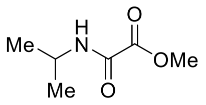

Prepared according to the general procedure **A** using propan-2-amine (887 mg, 15.0 mmol), methyl 2-chloro-2-oxoacetate (2.39 g, 19.5 mmol), and $NaHCO_3$ (1.89 g, 22.5 mmol) to give the title compound as a white solid (841 mg 5.79 mmol, 39%). $^{1}$H NMR (500 MHz, $CDCl_3$) δ = 4.18–4.06 (1H, m, $(CH_3)_2CH$), 3.90 (3H, $OCH_3$), 1.23 (6H, d, $^{3}J$ 6.6 Hz, $(CH_3)_2CH$).

**2-(Isopropylamino)-2-oxoacetic acid 1p:**

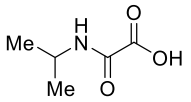

Prepared according to the general procedure **B** using Methyl 2-(isopropylamino)-2-oxoacetate **S1p** (842 mg, 5.8 mmol) and KOH (488 mg, 8.70 mmol) to give the title compound as a beige solid (500 mg, 3.81 mmol, 66%). NMR corresponds to literature reports:[8] $^{1}$H NMR (500 MHz, DMSO) δ = 13.72 (1H, COO*H*), 8.63 (1H, d, $^{3}J$ 7.8 Hz, N*H*), 4.00 – 3.76 (1H, m, $(CH_3)_2CH$), 1.10 (6H, d, $^{3}J$ 6.6 Hz, $(CH_3)_2CH$).

# 3. Optimization Studies

**General optimization procedure for the decarboxylative aminocarbonylation of (hetero)aryl bromides:** Oxamic acid **1a** (35.8 mg, 200 µmol, 2.00 eq.), 4-bromobenzonitrile (18.2 mg, 100 µmol, 1.00 eq.), $Li_2CO_3$ (14.8 mg, 200 µmol, 2.00 eq.) or another base (see conditions below), Ni salt, ligand, photocatalyst, additive (if required) were transferred to a 7 mL vial. The mixture was degassed via vacuum/argon cycles (3 times) and degassed $H_2O$ (unless noted otherwise) and degassed solvent were added and through the resulting mixture was bubbled argon for 3 minutes. The reaction mixture was irradiated with the indicated light source for 22 h, then diluted with EtOAc and $H_2O$. The aqueous phase was extracted with EtOAc (3×10 mL). The combined organic phase was washed with brine (3×20 mL), dried over $Na_2SO_4$, and the solvent was removed under reduced pressure. Durene (1,2,4,5-tetramethylbenzene) was added to the crude mixture. The yield and conversions were determined by $^1$H-NMR analysis.

### List of the photocatalysts used for the reaction condition optimization

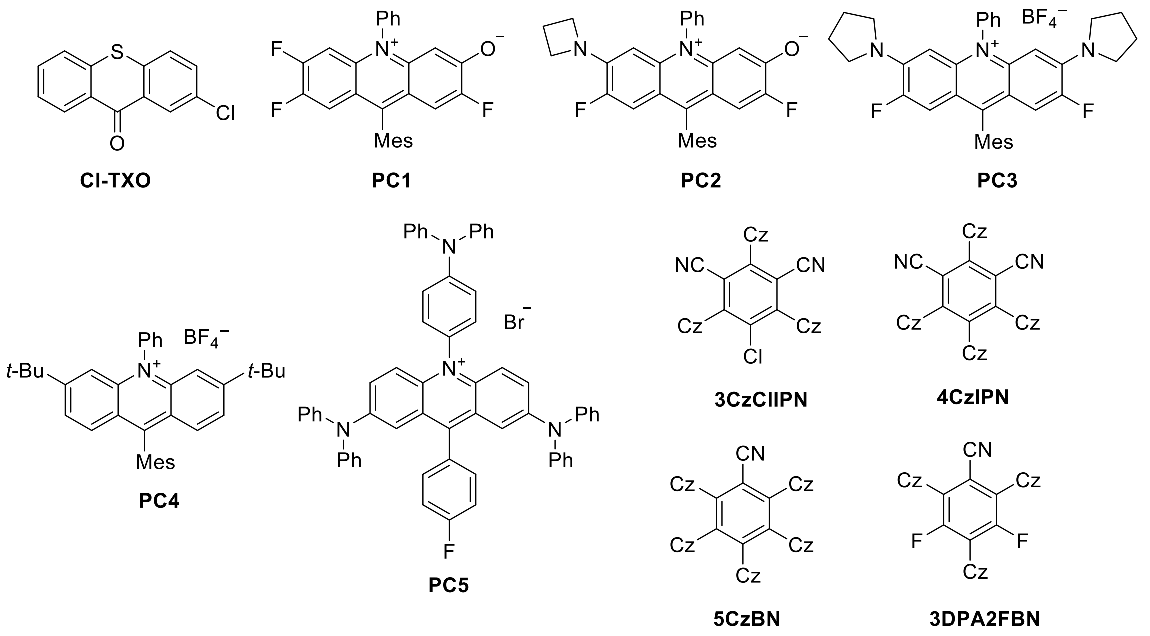


### Selected examples of the reaction condition optimization

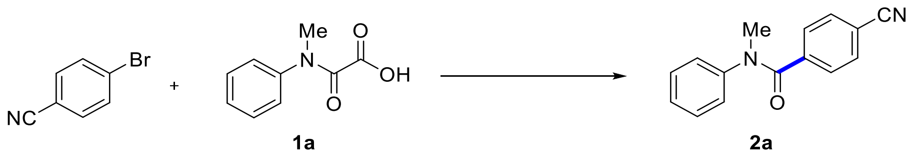


**Table S1.** Examination of the impact of diverse photocatalysts, base and additives

| Entry[a] | PC | PC (mol%) | Light Source | Solvent | Modifications | Conv. (%) | Yield (%) |
|---|---|---|---|---|---|---|---|
| 1[b] | Cl-TXO | 20 | Kessil tuna blue ($\lambda_{max}$ = 464 nm) | DMF | – | 45 | 29 |
| 2[c] | Cl-TXO | 20 | Kessil tuna blue | DMF | – | 39 | 25 |
| 3 | Cl-TXO | 20 | Kessil tuna blue | DMF | Under air | 41 | 20 |
| 4 | Cl-TXO | 20 | Kessil tuna blue | DMF | Salt of oxamic acid as a substrate (2.00 eq.) | 37 | 18 |
| 5 | Cl-TXO | 20 | Kessil tuna blue | DMF | Oxamic acid (3.00 eq.) | 36 | 19 |
| 6 | Cl-TXO | 20 | Kessil tuna blue | DMF | Oxamic acid (4.00 eq.) $Li_2CO_3$ (4.00 eq.) | 32 | 17 |

| Entry[a] | PC | PC (mol%) | Light Source | Solvent | Modifications | Conv. (%) | Yield (%) |
|---|---|---|---|---|---|---|---|
| 7 | Cl-TXO | 20 | Kessil tuna blue | DMF | Oxamic acid (5.00 eq.) $Li_2CO_3$ (5.00 eq.) | 50 | 21 |
| 8 | Cl-TXO | 20 | Kessil tuna blue | DMF | 4-Iodobenzonitrile instead of ArBr | NI | 5 |
| 9 | Cl-TXO | 20 | Kessil tuna blue | DMF | Phthalimide (0.25 eq.) | 56 | 27 |
| 10 | Cl-TXO | 20 | Kessil tuna blue | DMF | Phthalimide (0.50 eq.) | 53 | 28 |
| 11 | Cl-TXO | 20 | Kessil tuna blue | DMF | Phthalimide (1.00 eq.) | 34 | 21 |
| 12 | Cl-TXO | 20 | Kessil 390 nm (44°C) | DMF | – | NI | 30 |
| 13 | Cl-TXO | 20 | Kessil 390 nm (33°C) | DMF | – | NI | 29 |
| 14 | Cl-TXO | 20 | SOLIC-415C (400 nm filter) | DMF | – | 87 | 20 |
| 15 | Cl-TXO | 20 | Kessil 440 nm (48°C) | DMF | – | NI | 27 |
| 16 | Cl-TXO | 20 | SynLED | DMF | – | NI | 9 |
| 17 | Cl-TXO | 20 | Kessil tuna blue | DMF | $Na_2CO_3$ as base (2.00 eq.) | 49 | 22 |
| 18 | Cl-TXO | 20 | Kessil tuna blue | DMF | $K_2CO_3$ as base (2.00 eq.) | 58 | 28 |
| 19 | Cl-TXO | 20 | Kessil tuna blue | DMF | $Cs_2CO_3$ as base (2.00 eq.) | 49 | 23 |
| 20 | Cl-TXO | 20 | Kessil tuna blue | DMF | LiOH as base (2.00 eq.) | 61 | 26 |
| 21 | PC1 | 5 | SynLED | DMF | – | 0 | 0 |
| 22 | PC1 | 5 | SynLED | $CH_3CN$ | | 0 | 0 |
| 23 | PC1 | 5 | SynLED | $CH_3CN$ | No water | 0 | 0 |
| 24 | PC2 | 5 | SynLED | DMF | – | 0 | 0 |
| 25 | PC3 | 5 | SynLED | DMF | – | 0 | 0 |
| 26 | PC3 | 5 | SynLED | $CH_3CN$ | | 0 | 0 |
| 27 | PC3 | 5 | SynLED | $CH_3CN$ | No water | 0 | 0 |
| 28 | PC4 | 5 | SynLED | DMF | – | 0 | 0 |
| 29 | PC5 | 5 | Kessil tuna blue | DMF | | 0 | 0 |
| 30 | $[Ir(dF(CF_3)ppy)_2(dtbpy)]PF_6$ | 8 | SynLED | DMF | – | 85 | 44 |
| 31 | $[Ir(dF(CF_3)ppy)_2(dtbpy)]PF_6$ | 8 | Kessil tuna blue | DMF | – | 76 | 31 |
| 32 | 3CzClIPN | 3 | Kessil tuna blue | DMF | – | 0 | 0 |
| 33 | 5BzBN | 3 | Kessil tuna blue | DMF | – | 100 | 63 |
| 34 | 5BzBN | 3 | SynLED | DMF | – | 100 | 52 |
| 35 | 3DPA2FBN | 3 | Kessil tuna blue | DMF | – | 44 | 32 |
| 36 | 4CzIPN | 1 | Kessil tuna blue | DMF | – | 78 | 53 |
| 37 | 4CzIPN | 3 | Kessil tuna blue | DMF | – | 100 | 63 (51%)[d] |
| 38 | 4CzIPN | 3 | SynLED | DMF | – | 100 | 35 |
| 39 | 4CzIPN | 3 | Kessil 440 nm | DMF | | 100 | 39 |
| 40 | 4CzIPN | 3 | Kessil 440 nm | DMA | | 25 | 7 |
| 41 | 4CzIPN | 5 | Kessil tuna blue | DMF | | 100 | 45 |
| 42 | 4CzIPN | 8 | Kessil tuna blue | DMF | – | 100 | 47 |
| 43 | 4CzIPN | 8 | SynLED | DMF | – | 100 | 42 |
| 44 | 4CzIPN | 15 | Kessil tuna blue | DMF | – | 100 | 40 |
| 45 | 4CzIPN | 3 | Kessil tuna blue | DMF | No $NiCl_2$•glyme, no ligand | 0 | 0 |
| 46 | – | – | Kessil tuna blue | DMF | – | 0 | 0 |

[a]Reaction conditions (unless noted otherwise): 4-Bromobenzonitrile (18.2 mg, 100 µmol, 1.00 eq.), **1a** (35.8 mg, 200 µmol, 1.00 eq.), $Li_2CO_3$ (14.8 mg, 200 µmol, 1.00 eq.), $NiCl_2$•glyme (2.20 mg, 20.0 µmol, 0.10 eq.), dtbppy (4,4′-di-*tert*-butyl-2,2′-dipyridyl) (3.22 mg, 12.0 µmol, 0.12 eq.), PC, $H_2O$ (27.0 mg, 1.50 mmol, 15.0 eq.), solvent (3.0 mL), argon, 22 h, irradiation (see the Table), the yield was determined by the $^1$H-NMR analysis using durene as an internal standard; [b]the vial was placed at a distance of 10 cm of the light source; [c]the vial was placed into the photoreactor; [d]Isolated yield (after silica gel column chromatography (cyclohexane/EtOAc from 1:0 to 3:1).

**Table S2.** Examination of the impact of different nickel salts and ligands

| Entry[a] | PC | PC (mol%) | [Ni] | [Ni] (mol%) | Ligand | [L] (mol%) | Conversion (%) | Yield (%) |
|---|---|---|---|---|---|---|---|---|
| 1 | Cl-TXO | 20 | $NiCl_2$•glyme | 5 | dtbbpy | 6 | 28 | 15 |
| 2 | Cl-TXO | 20 | $NiCl_2$•glyme | 10 | dtbbpy | 12 | 39 | 25 |
| 3 | Cl-TXO | 20 | $NiCl_2$•glyme | 20 | dtbbpy | 24 | 52 | 24 |
| 4 | Cl-TXO | 20 | $NiCl_2$•glyme | 30 | dtbbpy | 36 | NI | 23 |

| Entry[a] | PC | PC (mol%) | [Ni] | [Ni] (mol%) | Ligand | [L] (mol%) | Conversion (%) | Yield (%) |
|---|---|---|---|---|---|---|---|---|
| 5 | Cl-TXO | 20 | $NiCl_2$•glyme | 40 | dtbbpy | 48 | NI | 19 |
| 6 | Cl-TXO | 20 | $NiBr_2$•glyme | 10 | dtbbpy | 12 | 28 | 16 |
| 7 | Cl-TXO | 20 | $Ni(OAc)_2$ | 10 | dtbbpy | 12 | 21 | 9 |
| 8 | Cl-TXO | 20 | $NiBr_2$•glyme | 10 | tpy | 12 | 0 | 0 |
| 9 | 4CzIPN | 3 | $Ni(dtbppy)_2Cl_2$ | 10 | – | – | 100 | 52 |
| 10[b] | 4CzIPN | 3 | $Ni(dtbppy)_2Cl_2$ | 10 | – | – | 33 | 7 |
| 11 | 4CzIPN | 3 | $NiCl_2$•glyme | 10 | 4,4'-Dimethyl-2,2'-bipyridine | 12 | 100 | 58 |
| 12 | 4CzIPN | 3 | $NiCl_2$•glyme | 10 | 5,5′-Dimethyl-2,2′-bipyridine | 12 | 100 | 47 |
| 13 | 4CzIPN | 3 | $NiCl_2$•glyme | 10 | BPhen | 12 | 100 | 48 |
| 14 | 4CzIPN | 3 | $NiCl_2$•glyme | 10 | 6,6'-Dimethyl-2,2'-bipyridine | 12 | 0 | 0 |
| 15 | 4CzIPN | 3 | $NiCl_2$•glyme | 10 | 4-4′-Dimethoxy-2-2′-bipyridine | 12 | 100 | 49 |
| 16 | 4CzIPN | 3 | $Ni(PCy_3)_2Cl_2$ | 10 | dtbbpy | 12 | 0 | 0 |
| 17[c] | 4CzIPN | 3 | $Ni(PCy_3)_2Cl_2$ | 10 | dtbbpy | 12 | 0 | 0 |

[a]Reaction conditions (unless noted otherwise): 4-Bromobenzonitrile (18.2 mg, 100 µmol, 1.00 eq.), **1a** (35.8 mg, 200 µmol, 1.00 eq.), $Li_2CO_3$ (14.8 mg, 200 µmol, 1.00 eq.), [Ni] (see the table), ligand (see the table), PC (see the table), $H_2O$ (27.0 mg, 1.50 mmol, 15.0 eq.), DMF (3.0 mL), argon, 22 h, Kessil tuna blue ($\lambda_{max}$ = 464 nm), the yield was determined by the $^1$H-NMR analysis using durene as an internal standard; [b]DMA as a solvent; [c]THF as a solvent.

**Table S3.** Impact of different water content on the reaction

| Entry | PC | PC (mol%) | $H_2O$ (eq.) | Conversion (%) | Yield (%) |
|---|---|---|---|---|---|
| 1 | Cl-TXO | 20 | 0 | 54 | 28 |
| 2 | 4CzIPN | 3 | 0 | 100 | 45 |
| 3 | Cl-TXO | 20 | 15 | 39 | 25 |
| 4 | Cl-TXO | 20 | 30 | 40 | 20 |
| 5 | Cl-TXO | 20 | 100 | 23 | 16 |

[a]Reaction conditions (unless noted otherwise): 4-Bromobenzonitrile (18.2 mg, 100 µmol, 1.00 eq.), **1a** (35.8 mg, 200 µmol, 1.00 eq.), $Li_2CO_3$ (14.8 mg, 200 µmol, 1.00 eq.), $NiCl_2$•glyme (2.20 mg, 20.0 µmol, 0.10 eq.), dtbppy (4,4′-di-*tert*-butyl-2,2′-dipyridyl) (3.22 mg, 12.0 µmol, 0.12 eq.), PC (see the table), $H_2O$ (see the table), DMF (3.0 mL), argon, 22 h, Kessil tuna blue ($\lambda_{max}$ = 464 nm), the yield was determined by the $^1$H-NMR analysis using durene as an internal standard.

**Table S4.** Screening of solvents, concentrations

| Entry[a] | PC | PC (mol%) | Solvent | Concentration (mM) | Conversion (%) | Yield (%) |
|---|---|---|---|---|---|---|
| 1 | Cl-TXO | 20 | DMF | 33 | 39 | 25 |
| 2 | Cl-TXO | 20 | DMSO | 33 | >90 | 33 |
| 3 | Cl-TXO | 20 | DME | 33 | 0 | 0 |
| 4 | Cl-TXO | 20 | DMA | 33 | 61 | 34 |
| 5 | 4CzIPN | 3 | $CH_3CN$ | 33 | 0 | 0 |
| 6 | 4CzIPN | 3 | DMC | 33 | 0 | 0 |
| 7 | 4CzIPN | 3 | THF | 33 | 0 | 0 |
| 8 | 4CzIPN | 3 | $CH_3CN$ | 33 | 0 | 0 |
| 9 | 4CzIPN | 3 | DMF | 20 | 100 | 36 |
| 10 | 4CzIPN | 3 | DMF | 33 | 100 | 63 |
| 11 | 4CzIPN | 3 | DMF | 50 | 100 | 61 |
| 12 | 4CzIPN | 3 | DMF | 100 | 100 | 64 |

[a]Reaction conditions (unless noted otherwise): 4-Bromobenzonitrile (18.2 mg, 100 µmol, 1.00 eq.), **1a** (35.8 mg, 200 µmol, 1.00 eq.), $Li_2CO_3$ (14.8 mg, 200 µmol, 1.00 eq.), $NiCl_2$•glyme (2.20 mg, 20.0 µmol, 0.10 eq.), dtbppy (4,4′-di-*tert*-butyl-2,2′-dipyridyl) (3.22 mg, 12.0 µmol, 0.12 eq.), PC (see the table), $H_2O$ (27.0 mg, 1.50 mmol, 15.0 eq.), solvent (see the table), argon, 22 h, Kessil tuna blue ($\lambda_{max}$ = 464 nm), the yield was determined by the $^1$H-NMR analysis using durene as an internal standard.

# 4. Substrate Scope

**General procedure C for the decarboxylative aminocarbonylation of (hetero)aryl bromides 2a-2l, 3a-3h:** The corresponding bromide (200 µmol, 1.00 eq.), oxamic acid **1** (400 µmol, 2.00 eq.), $Li_2CO_3$ (29.6 mg, 400 µmol, 2.00 eq.), $NiCl_2$•glyme (4.39 mg, 20.0 µmol, 0.10 eq.), dtbbpy (6.44 mg, 24.0 µmol, 0.12 eq.), 4CzIPN (4.73 mg, 6.00 µmol, 3 mol%) were transferred to a 7 mL vial. The mixture was degassed via vacuum/argon cycles (3 times) and degassed $H_2O$ (54.0 mg, 3.00 mmol, 15.0 eq.) and 4.0 mL of degassed DMF were added and through the resulting mixture was bubbled argon for 3 minutes. The reaction mixture was irradiated with a Kessil tuna blue lamp ($\lambda_{max}$ = 464 nm) with fan cooling for 22 h, then diluted with EtOAc and $H_2O$. The aqueous phase was extracted with EtOAc (3×15 mL). The combined organic phase was washed with brine (3×20 mL), dried over $Na_2SO_4$ and the solvent was removed under reduced pressure. The residue was purified by silica gel column chromatography (cyclohexane/EtOAc from 1:0 to 3:1) to yield the desired product.

### ¹H NMR of crude reaction mixtures

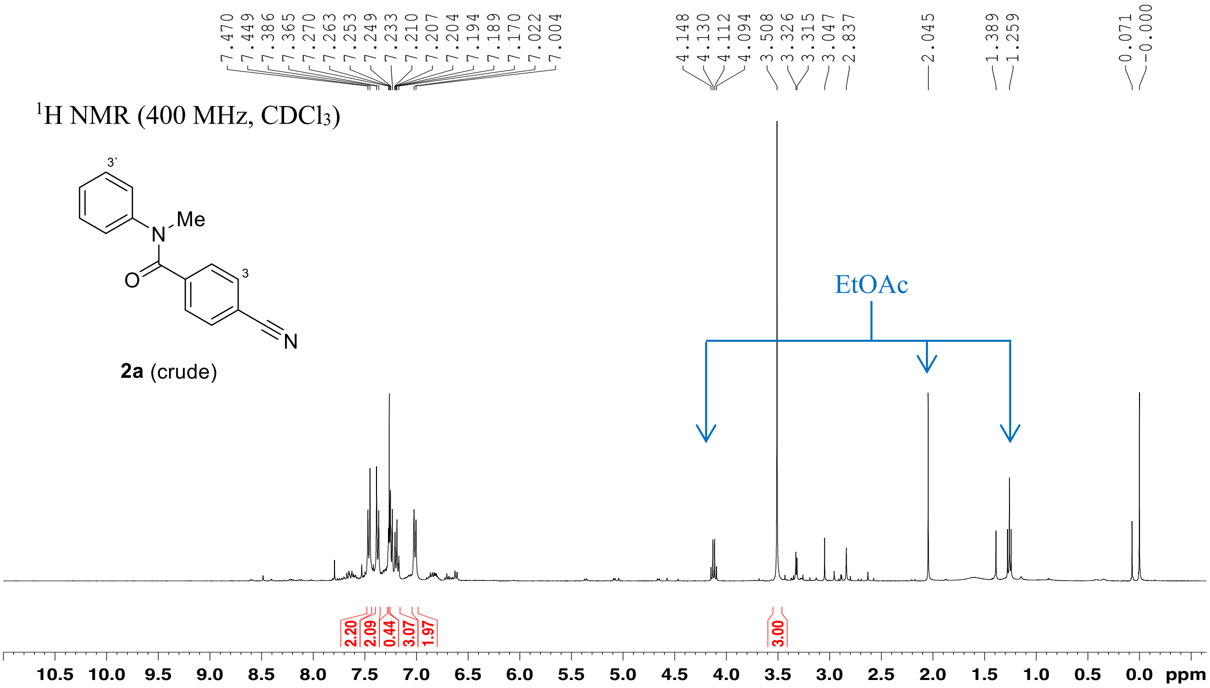


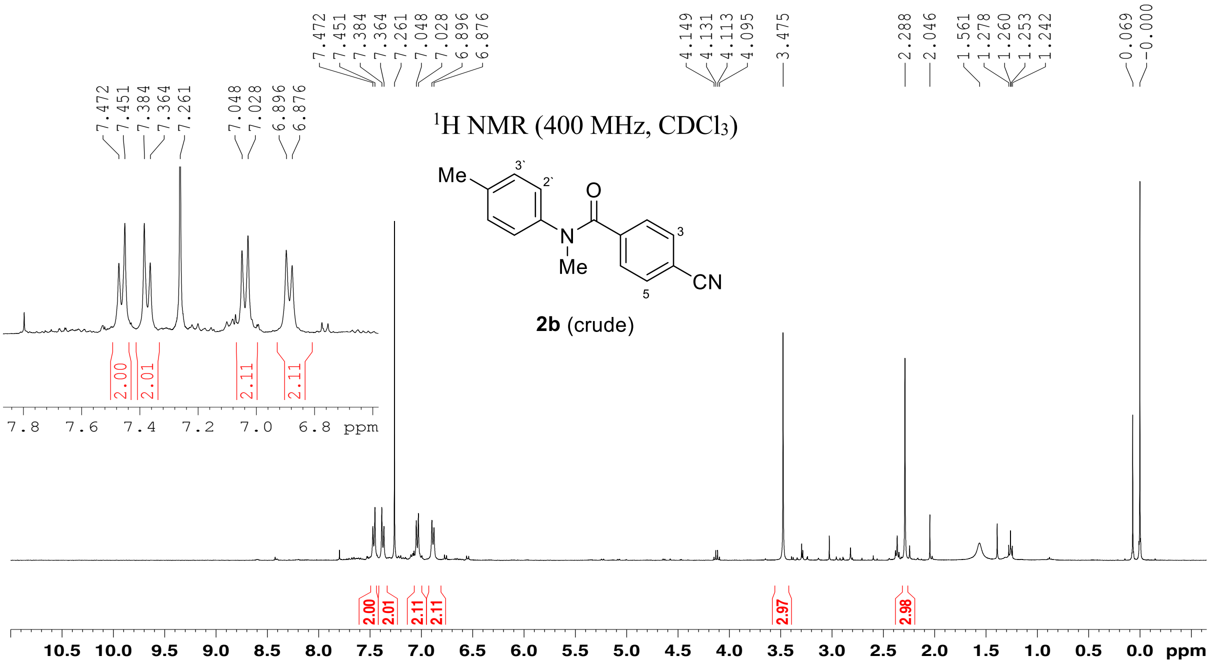

**4-Cyano-*N*-methyl-*N*-phenylbenzamide 2a:**

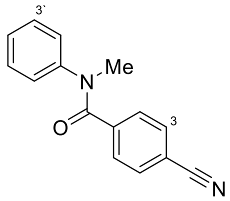

Prepared according to the general procedure **C** using 2-(methyl(phenyl)amino)-2-oxoacetic acid **1a** (71.7 mg, 400 µmol, 2.00 eq.) and 4-bromobenzonitrile (36.4 mg, 200 µmol, 1.00 eq.) to give the title compound as a white solid (31.1 mg, 132 µmol, 66%, m.p. 100.5 – 101.0°C): R*f* 0.58 (cyclohexane/EtOAc 1:1); $\nu_{max}$ (neat): 3067w, 2960w, 2913w, 2838w, 2231m, 1642m, 1595m, 1492m, 1417w, 1375s, 1302m, 1173w, 1102m, 1025m, 850w, 755s, 701s; $^1$H NMR (500 MHz, $CDCl_3$) δ = 7.46 (2H, d, $^3J$ 8.4 Hz, C3*H*, C5*H*), 7.38 (2H, d, $^3J$ 8.4 Hz, C2*H*, C6*H*), 7.27 – 7.23 (2H, m, C3`*H*, C5`*H*), 7.21 – 7.16 (1H, m, C4`*H*), 7.01 (2H, d, $^3J$ 7.5 Hz, C2`*H*, C6`*H*), 3.51 (3H, s, N–C$H_3$); $^{13}$C NMR (126 MHz, $CDCl_3$) δ = 168.8 (*C*=O), 144.1 (*C4`*), 140.5 (*C1*), 131.8 (*C3*, *C5*), 129.7 (*C3`*, *C5`*), 129.4 (*C2*, *C6*), 127.4 (*C4`*), 127.1 (*C2`*, *C6`*), 118.3 (*C*≡*N*), 113.3 (*C4*), 38.5 (N-*C*$H_3$); HMRS (ESI) m/z: [M+H$^+$] Calcd. for $C_{15}H_{13}N_2O$ 237.1022; Found 237.1021.

**4-Cyano-*N*-methyl-*N*-(*p*-tolyl)benzamide 2b:**

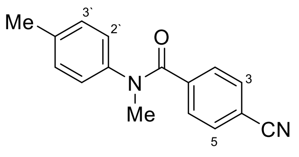

Prepared according to the general procedure **C** using 2-(methyl(*p*-tolyl)amino)-2-oxoacetic acid **1b** (77.3 mg, 400 µmol, 2.00 eq.) and 4-bromobenzonitrile (36.4 mg, 200 µmol, 1.00 eq.) to give the title compound as a white solid (37.3 mg, 149 µmol 75%, m.p. 113.0 – 113.9°C): R*f* 0.39 (cyclohexane/EtOAc 2:1); $\nu_{max}$ (neat): 3036w, 2923w, 2854w, 2229m, 1641s, 1512s, 1424m, 1370s, 1301m, 1176w, 1105w, 1020w, 916w, 850m, 720w, 631s cm$^{-1}$; $^1$H NMR (500 MHz, $CDCl_3$) δ = 7.46 (2H, d, $^3J$ 8.1 Hz, C3*H*, C5*H*), 7.37 (2H, d, $^3J$ 8.1 Hz, C2*H*, C6*H*), 7.04 (2H, d, $^3J$ 8.0 Hz, C3`*H*, C5`*H*), 6.89 (2H, d, $J^3$ 8.0 Hz, C2`*H*, C6`*H*), 3.48 (3H, s, N–C$H_3$), 2.29 (3H, s, C4`–C$H_3$); $^{13}$C NMR (126 MHz, $CDCl_3$) δ = 168.7 (*C*=O), 141.3 (*C1`*), 140.5 (*C1*), 137.3 (*C4`*), 131.6 (*C3*, *C5*), 130.1 (*C3`*, *C5`*), 129.2 (*C2*, *C6*), 126.7 (*C2`*, *C6`*), 118.2 (*C*≡N), 113.1 (*C4*), 38.4 (N–C*$H_3$*), 20.9 (C4`-*C*$H_3$); HMRS (ESI) m/z: [M+Na$^+$] Calcd. for $C_{16}H_{14}N_2NaO$ 273.0998; Found 273.0997.

***N*-(4-Chlorophenyl)-4-cyano-*N*-methylbenzamide 2c:**

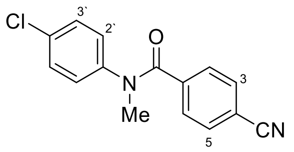

Prepared according to the general procedure **C** using 2-((4-chlorophenyl)(methyl)amino)-2-oxoacetic acid **1c** (85.4 mg, 400 µmol, 2.00 eq.) and 4-bromobenzonitrile (36.4 mg, 200 µmol, 1.00 eq.) to give the title compound as yellow oil (41.5 mg, 153 µmol 77%): R*f* 0.55 (cyclohexane/EtOAc 1:1); $\nu_{max}$ (neat): 3085w, 3046w, 2931w, 2229m, 1641s, 1595m, 1491s, 1423m, 1368w, 1308w, 1176w, 1097s, 1014m, 845s, 776w, 717w cm$^{-1}$; $^1$H NMR (500 MHz, $CDCl_3$) δ = 7.51 (2H, d, $^3J$ 8.3 Hz, C3*H*, C5*H*), 7.38 (2H, d, $^3J$ 8.3 Hz, C2*H*, C6*H*), 7.26 – 7.18 (2H, m, C3`*H*, C5`*H*), 6.96 (2H, d, $^3J$ 8.5 Hz, C2`*H*, C6`*H*), 3.48 (3H, s, N–C*$H_3$*); $^{13}$C NMR (126 MHz, $CDCl_3$) δ = 168.7 (*C*=O), 142.6 (*C1`*), 140.0 (*C1*), 133.15 (*C4`*), 131.9 (*C3*, *C5*), 129.9 (*C3`*, *C5`*), 129.3 (*C2*, *C6*), 128.2 (*C2`*, *C6`*), 118.1 (*C*≡N), 113.7 (*C4*), 38.5 (*C*$H_3$); HMRS (ESI) m/z: [M+H$^+$] Calcd. for $C_{15}H_{12}ClN_2O$ 271.0633; Found 271.0627.

**4-Cyano-*N*-(4-fluorophenyl)-*N*-methylbenzamide 2d:**

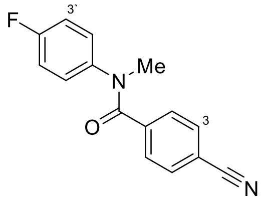


Prepared according to the general procedure **C** using 2-((4-fluorophenyl)(methyl)amino)-2-oxoacetic acid **1d** (78.9 mg, 400 µmol, 2.00 eq.) and 4- bromobenzonitrile (36.4 g, 200 µmol, 1.00 eq.) to give the title compound as yellow oil (44.0 mg, 173 µmol, 87%): $R_f$ 0.39 (cyclohexane/EtOAc 1:1); $\nu_{max}$ (neat): 3121w, 3071w, 2936w, 2230m, 1641s, 1506s, 1429m, 1369s, 1307m, 1222s, 1156w, 1103s, 1013m, 844s, 760m, 629m cm$^{-1}$; $^1$H NMR (500 MHz, $CDCl_3$) δ = 7.49 (2H, d, $^3J$ 8.0 Hz, C3*H*, C5*H*), 7.37 (2H, d, $^3J$ 8.0 Hz, C2*H*, C6*H*), 7.07 – 6.98 (2H, m, C2`*H*, C6`*H*), 6.95 (2H, t, $^3J$ 8.4 Hz, C3`*H*, C5`*H*), 3.48 (3H, s, N–C$H_3$); $^{13}$C NMR (126 MHz, $CDCl_3$) δ = 168.8 (*C*=O), 161.2 (d, $^1J_{CF}$ 248.7 Hz, *C4`*), 140.2 (*C1`*/*C1*), 131.9 (*C3*, *C5*), 129.2 (*C2*, *C6*), 128.7 (d, $^3J_{CF}$ 8.6 Hz,*C2`*, *C6`*), 118.1 (*C*≡N), 116.6 (d, $^2J_{CF}$ 22.8 Hz, *C3`*, *C5`*), 113.5 (*C4*), 38.6 (N–*C*$H_3$); $^{19}$F NMR (471 MHz, $CDCl_3$) δ = –113.34. HMRS (ESI) m/z: [M+H$^+$] Calcd. for $C_{15}H_{12}FN_2O$ 255.0928; Found 255.0924.

**4-Cyano-*N*,*N*-diphenylbenzamide 2e:**

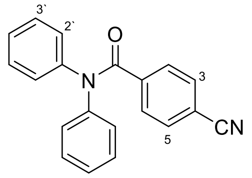


Prepared according to the general procedure **C** using 2-(diphenylamino)-2-oxoacetic acid **1e** (96.5 mg, 400 µmol, 2.00 eq.) and 4-bromobenzonitrile (36.4 mg, 200 µmol, 1.00 eq.) to give the title compound as a white solid (44.0 mg, 147 µmol 74%, m.p. 142.0 – 142.8°C): $R_f$ 0.72 (cyclohexane/EtOAc 1:1); $\nu_{max}$ (neat): 3061w, 2922w, 2838w, 2229m, 1654s, 1590m, 1489s, 1451w, 1402w, 1348s, 1306m, 1169w, 1109w, 1073w, 1018w, 960w, 912w, 842m, 756m, 694s cm$^{-1}$; $^1$H NMR (500 MHz, $CDCl_3$) δ 7.56 – 7.52 (2H, m, C2*H*, C6*H*), 7.52 – 7.49 (2H, C3*H*, C5*H*), 7.31 (4H, t, $^3J$ 7.3 Hz, 2 × C3`*H*, 2 × C5`*H*), 7.23 (2H, t, $^3J$ 7.4 Hz, 2H, 2 × C4`*H*), 7.19 – 7.11 (4H, m, 2 × C2`*H*, 2 × C6`*H*); $^{13}$C NMR (126 MHz, $CDCl_3$) δ = 168.7 (*C*=O), 143.1 (*C1`*), 140.7 (*C1*), 131.9 (*C3*, *C5*), 129.6 (*C2*, *C6*), 129.5 (*C3`*, *C5`*), 127.5 (*C2`*, *C6`*), 127.1 (*C4`*), 118.2 (*C*≡N), 113.7 (*C4*); HMRS (ESI) m/z: [M+H$^+$] Calcd. for $C_{20}H_{15}N_2O$ 299.1179; Found 299.1179.

**4-(Morpholine-4-carbonyl)benzonitrile methane 2f:**

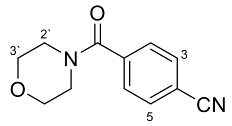


Prepared according to the general procedure **C** using 2-morpholino-2-oxoacetic acid **1e** (63.7 mg, 400 µmol, 2.00 eq.) and 4-bromobenzonitrile (36.4 mg, 200 µmol, 1.00 eq.) to give the title compound as a white solid (12.3 mg, 56.9 µmol 28%, m.p. 123.0 – 123.8°C): $R_f$ 0.15 (cyclohexane/EtOAc 1:1); $\nu_{max}$ (neat): 2971m, 2907m, 2861m, 2230m, 1631s, 1434s, 1277s, 1258s, 1154w, 1113s, 1067m, 1015m, 894w, 841w, 631s cm$^{-1}$; $^1$H NMR (500 MHz, $CDCl_3$) δ = 7.82 – 7.66 (2H, m, C3*H*, C5*H*), 7.56 – 7.40 (2H, m, C2*H*, C6*H*), 3.92 – 3.23 (8H, m, C2`$H_2$, C3`$H_2$, C5`$H_2$, C6`$H_2$,); $^{13}$C NMR (126 MHz, $CDCl_3$) δ = 168.4 (*C*=O), 139.8 (*C1*), 132.6 (*C3*, *C5*), 127.9 (*C2*, *C6*), 118.1 (*C*≡N), 113.9 (*C4*), 66.9 (*C3`*, *C5`*), 48.2 (*C2`*, *C6`*), 42.7 (*C2`*, *C6`*); HMRS (ESI) m/z: [M+H$^+$] Calcd. for $C_{12}H_{13}N_2O_2$ 217.0972; Found 217.0979.

***N*-((3s,5s,7s)-Adamantan-1-yl)-4-cyano-*N*-methylbenzamide 2g:**

Prepared according to the general procedure **C** using 2 2-(((3s,5s,7s)-adamantan-1-yl)(methyl)amino)-2-oxoacetic acid **1g** (94.9 mg,

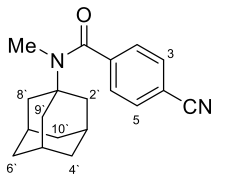

400 µmol, 2.00 eq.) and 4- bromobenzonitrile (36.4 g, 200 µmol, 1.00 eq.) to give the title compound as a white solid (42.3 mg, 144 µmol 72%, m.p. 186.1 – 186.8°C): $R_f$ 0.78 (cyclohexane/EtOAc 1:1); $\nu_{max}$ (neat): 2920s, 2907s, 2854m, 2231m, 1627s, 1454wm 1384m, 1359w, 1280w, 1207w, 1165w, 1103w, 1065s, 909w, 854w, 762w cm$^{-1}$;$^{1}$H NMR (500 MHz, $CDCl_3$) δ = 7.68 (2H, d, $^{3}J$ 8.4 Hz, C3*H*, C5*H*), 7.56 – 7.42 (2H, m, C2*H*, C6*H*), 2.80 (3H, s, N–C$H_3$), 2.26 (6H, d, $^{3}J$ 2.8 Hz, C2\`$H_2$, C9\`$H_2$, C8\`$H_2$), 2.21 – 2.08 (3H, m, C3\`*H*, C5\`*H*, C7\`*H*), 1.80 – 1.63 (6H, m, C4\`$H_2$, C6\`$H_2$, C10\`$H_2$); $^{13}$C NMR (126 MHz, $CDCl_3$) δ = 171.0 (*C*=O), 143.8 (*C1*), 132.5 (*C3*, *C5*), 127.8 (*C2*, *C6*), 118.5 (*C*≡N), 113.0 (*C4*), 58.5 (*C1\`*), 39.2 (*C2\`*, *C8\`*, *C9\`*), 36.5 (*C4\`*, *C6\`*, *C10\`*), 34.2 (N–*C*$H_3$), 30.0 (*C3\`*, *C5\`*, *C7\`*); HMRS (ESI) m/z: [M+H$^+$] Calcd. for $C_{19}H_{23}N_2O$ 295.1805; Found 295.1809 [M+H$^+$].

### *N*-Benzyl-4-cyano-*N*-phenylbenzamide 2h:

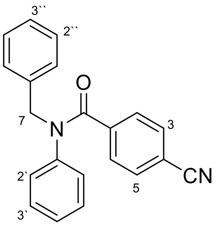

Prepared according to the general procedure **C** using 2-(benzyl(phenyl)amino)-2-oxoacetic acid **1h** (102 mg, 400 µmol, 2.00 eq.) and 4-bromobenzonitrile (36.4 mg, 200 µmol, 1.00 eq.) to give the title compound as a yellow oil (45.0 mg, 144 µmol, 72%): $R_f$ 0.65 (cyclohexane/EtOAc 1:1); $\nu_{max}$ (neat): 3085w, 3063w, 3031w, 2963w, 2928w, 2842w, 2230m, 1641s, 1594m, 1494m, 1494m, 1454w, 1387s, 1301m, 1281m, 1220w, 1149w, 1078m, 1020m, 975w, 912w, 849m, 812w, 758m, 698s cm$^{-1}$; $^{1}$H NMR (500 MHz, $CDCl_3$) δ = 7.49 – 7.43 (2H, m, C3*H*, C5*H*), 7.42 – 7.37 (2H, m, C2*H*, C6*H*), 7.33 – 7.26 (5H, m, C2\`\`*H*, C3\`\`*H*, C4\`\`*H*, C5\`\`*H*, C6\`\`*H*), 7.17 – 7.11 (3H, m, C3\`*H*, C4\`*H*, C5\`*H*), 6.87 (2H, d, $^{3}J$ 7.7 Hz, C2\`*H*, C6\`*H*), 5.12 (2H, s, C7$H_2$); $^{13}$C NMR (126 MHz, $CDCl_3$) δ = 168.7 (*C*=O), 142.6 (*C1\`*), 140.5 (*C1*), 137.0 (*C1\`\`*), 131.7 (*C3*, *C5*), 129.5 (*C3\`*, *C5\`*), 129.3 (*C2*, *C6*), 128.7 (*C2\`\`*, *C3\`\`*, *C5\`\`*, *C6\`\`*), 128.0 (*C2\`*, *C6\`*), 127.8 (*C4\`\`*), 127.6 (*C4\`*), 118.2 (*C*≡N), 113.3 (*C4*), 54.0 (*C7*); HMRS (ESI) m/z: [M+H$^+$] Calcd. for $C_{21}H_{17}N_2O$ 313.1335; Found 313.1332.

### 4-(1,2,3,4-Tetrahydroquinoline-1-carbonyl)benzonitrile 2i:

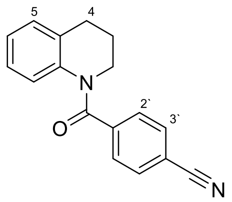

Prepared according to the general procedure **C** using 2-(3,4-dihydroquinolin-1(2*H*)-yl)-2-oxoacetic acid **1i** (82.1 mg, 400 µmol, 2.00 eq.) and 4-bromobenzonitrile (36.4 mg, 200 µmol, 1.00 eq.) to give the title compound as a beige solid (36.0 mg, 137 µmol, 69%, m.p. 148.0 – 148.9°C): $R_f$ 0.83 (cyclohexane/EtOAc 1:1); $\nu_{max}$ (neat): 3071w, 2950w, 2881w, 2845w, 2229m, 1639s, 1580m, 1492s, 1458m, 1381s, 1302w, 1264m, 1213w, 1149w, 1063w, 1018w, 941w, 850m, 756s, 707w cm$^{-1}$; $^{1}$H NMR (500 MHz, $CDCl_3$) δ = 7.56 (2H, d, $^{3}J$ 8.2 Hz, C3\`*H*, C5\`*H*), 7.43 (2H, d, $^{3}J$ 8.2 Hz, C2\`*H*, C6\`*H*), 7.18 (1H, d, $^{3}J$ 7.5 Hz, C5*H*), 7.04 (1H, t, $^{3}J$ 7.5 Hz, C6*H*), 6.87 (1H, t, $^{3}J$ 7.5 Hz, C7*H*), 6.56 (1H, br. s, C8*H*), 3.92 (2H, t, $^{3}J$ 6.6 Hz, C2$H_2$), 2.85 (2H, t, $^{3}J$ 6.6 Hz, C4$H_2$), 2.08 (2H, p, $^{3}J$ 6.6 Hz, C3$H_2$); $^{13}$C NMR (126 MHz, $CDCl_3$) δ = 168.2 (*C*=O), 140.7 (*C1\`*), 138.5 (*C8a*), 132.3 (*C4a*), 132.0 (*C3\`*, *C5\`*), 129.4 (*C2\`*, *C6\`*), 128.7 (*C5*), 126.1 (*C7*), 125.5 (*C6*), 118.3 (*C*≡N), 113.8 (*C1\`*), 44.4 (*C2*), 27.0 (*C4*), 24.1 (*C3*); HMRS (ESI) m/z: [M+H$^+$] Calcd. for $C_{17}H_{15}N_2O$ 263.1179; Found 263.1174.

**4-Cyano-*N*-(4-methoxyphenyl)-*N*-methylbenzamide 2j:**

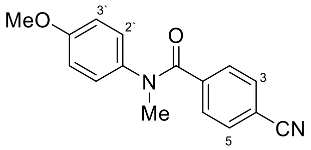


Prepared according to the general procedure **C** using 2-((4-methoxyphenyl)(methyl)amino)-2-oxoacetic acid **1j** (83.7 mg, 400 µmol, 2.00 eq.) and 4-bromobenzonitrile (36.4 mg, 200 µmol, 1.00 eq.) to give the title compound as a white solid (41.9 mg, 157 µmol 79%, m.p. 107.5 – 108.1°C).

**Procedure for gram-scale synthesis:** 4-Bromobenzonitrile (910 mg, 5.00 mmol, 1.00 eq.), 2-((4-methoxyphenyl)(methyl)amino)-2-oxoacetic acid **1j** (2.09 g, 10.0 mmol, 2.00 eq.), $Li_2CO_3$ (739 mg, 10.0 mmol, 2.00 eq.), $NiCl_2$•glyme (110 mg, 500 µmol, 0.10 eq.), dtbbpy (161 mg, 600 µmol, 0.12 eq.), 4CzIPN (118 mg, 150 µmol, 3 mol%) were transferred to a 250 mL round-bottom flask. The mixture was degassed via vacuum/argon cycles (3 times) and degassed $H_2O$ (1.35 g, 75.0 mmol, 15.0 eq.), 100 mL of degassed DMF were added and the resulting mixture was bubbled with argon for 3 minutes. The flask was placed at a 10 cm distance of the Kessil tuna blue ($\lambda_{max}$ = 464 nm) with a fan cooling for 22 h. The reaction mixture was then diluted with EtOAc and $H_2O$. Aqueous phase was extracted with EtOAc (3×60 mL). The combined organic phase was washed with brine (3×100 mL), dried over $Na_2SO_4$ and the solvent was removed under reduced pressure. The residue was purified by silica gel column chromatography (cyclohexane/EtOAc from 1:0 to 3:1) to yield the desired product as a white solid (1.12 g, 4.21 mmol, 84%): $R_f$ 0.43 (cyclohexane/EtOAc 1:1); $\nu_{max}$ (neat): 3093wm 3017w, 2955w, 2927w, 2897w, 2874w, 2832w, 2229m, 1647s, 1509s, 1461m, 1355m, 1287m, 1243s, 1173m, 1108s, 1029s, 860m, 762w, 702w cm$^{-1}$; $^1$H NMR (500 MHz, $CDCl_3$) δ = 7.47 (2H, d, $^3J$ 7.7 Hz, C3*H*, C5*H*), 7.37 (2H, d, $^3J$ 7.7 Hz, C2*H*, C6*H*), 6.93 (2H, d, $^3J$ 8.7 Hz, C2`*H*, C6`*H*), 6.75 (2H, d, $^3J$ 8.7 Hz, C3`, C5`*H*), 3.76 (3H, s, OC*H*3), 3.46 (3H, s, N–*C*H3); $^{13}$C NMR (126 MHz, $CDCl_3$) δ = 168.8 (*C*=O), 158.5 (*C4`*), 140.7 (*C1*), 136.8 (*C1`*), 131.7 (*C3*, *C5*), 129.2 (*C2*, *C6*), 128.2 (*C2`*, *C6`*), 118.3 (*C*≡N), 114.8 (*C3`*, *C5`*), 113.1 (*C4*), 55.5 (O*C*H3), 38.56 (N–*C*H3); HMRS (ESI) m/z: [M+H$^+$] Calcd. for $C_{16}H_{15}N_2O_2$ 267.1128; Found 267.1125.

**4-Cyano-*N*-isopropyl-*N*-phenylbenzamide 2k:**

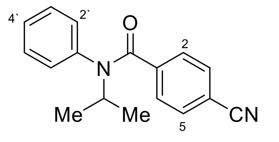


Prepared according to the general procedure **C** using 2-(isopropyl(phenyl)amino)-2-oxoacetic acid **1k** (82.9 mg, 400 µmol) and 4-bromobenzonitrile (36.4 mg, 200 µmol, 1.00 eq.) to give the title compound as a white solid (36.5 mg, 138 µmol, 69%, m.p. 133.9 – 134.5°C): $R_f$ 0.30 (cyclohexane/EtOAc 3:1); $\nu_{max}$ (neat): 3062w, 2976m, 2936w, 2869w, 2229m, 1639s, 1591m, 1494m, 1394s, 1346s, 1253m, 1116m, 1021w, 914w, 855m, 758m, 703s cm$^{-1}$; $^1$H NMR (500 MHz, $CDCl_3$) δ = 7.41 (2H, d, $^3J$ 7.9 Hz, C3*H*, C5*H*), 7.31 (2H, d, $^3J$ 7.9 Hz, C2*H*, C6*H*), 7.26 – 7.18 (3H, m, C3`*H*, C4`*H*, C5`*H*), 7.00 (2H, d, $^3J$ 7.4 Hz, C2`*H*, C6`*H*), 5.17 – 4.95 (1H, m, C*H*($CH_3)_2$), 1.22 (6H, d, $^3J$ 6.8 Hz, CH(C*H*3)2); $^{13}$C NMR (126 MHz, $CDCl_3$) δ = 168.8 (*C*=O), 141.8 (*C1*), 138.8 (*C1`*), 131.6 (*C3*, *C5*), 130.6 (*C2`*, *C6`*), 129.1 (*C3`*, *C5`*), 128.7 (*C2*, *C6*), 128.1 (*C4`*), 118.3 (*C*≡N), 112.6 (*C4*), 48.0 (*C*H($CH_3)_2$), 21.1 (CH(*C*H3)2); HMRS (ESI) m/z: [M+H$^-$] Calcd. for $C_{17}H_{17}N_2O$ 265.1335; Found 265.1327.

## 4-Cyano-*N*-methyl-*N*-(*o*-tolyl)benzamide 2l:

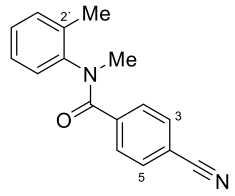


Prepared according to the general procedure **C** using 2-(methyl(*o*-tolyl)amino)-2-oxoacetic acid **1l** (77.3 mg, 400 µmol, 2.00 eq.) and 4-bromobenzonitrile (36.4 g, 200 µmol, 1.00 eq.) to give the title compound as a white solid (33.8 mg, 135 µmol, 68%, m.p.126.0 – 126.5 °C): $R_f$ 0.56 (cyclohexane/EtOAc 1:1); $\nu_{max}$ (neat): 2973s, 2902s, 2223m, 1642s, 1493m, 1376s, 1301m, 1252m, 1052s, 851m, 760m, 727m, 631s cm$^{-1}$; $^1$H NMR (500 MHz, $CDCl_3$) δ 7.47 – 7.40 (2H, m, C3*H*, C5*H*), 7.38 – 7.32 (2H, m, C2*H*, C6*H*), 7.20 – 7.05 (3H, m, C3`*H*, C4`*H*, C5`*H*), 7.01 (1H, d, $^3J$ 8.0 Hz, C6`*H*), 3.39 (3H, s, NC$H_3$), 2.21 (3H, s, C2`–C$H_3$); $^{13}$C NMR (126 MHz, $CDCl_3$) δ = 168.9 (*C*=O), 142.6 (*C1`*), 140.3 (*C1*), 134.8 (*C2`*), 131.7 (*C3`*), 131.6 (*C3*, *C5*), 128.8 (*C2*, *C6*), 128.6 (*C6`*), 128.5 (*C4`*), 127.4 (*C5`*), 118.2 (*C*≡N), 113.4 (*C4*), 37.6 (N–*C*$H_3$), 17.8 (C2`–*C*$H_3$); HMRS (ESI) m/z: [M+H$^+$] Calcd. for $C_{16}H_{15}N_2O$ 251.1179; Found 251.1178.

## *N*-Methyl-*N*-phenyl-4-(trifluoromethyl)benzamide 3a:

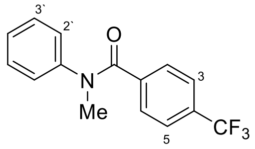


Prepared according to the general procedure **C** using 2-(methyl(phenyl)amino)-2-oxoacetic acid **1a** (71.7 mg, 400 µmol, 2.00 eq.) and *p*-(trifluoromethyl)bromobenzene (45.0 mg, 200 µmol, 1.00 eq.) to give the title compound as a white solid (13.5 mg, 48.3 µmol 24%, m.p. 77.0 – 78.0°C): $R_f$ 0.32 (cyclohexane/EtOAc 3:1); $\nu_{max}$ (neat): 3082w, 2958w, 2920w, 2847w, 1641m, 1594m, 1495m, 1409m, 1378m, 1324s, 1167m, 1105s, 1065s, 1017m, 854s, 805w, 766m, 700s cm$^{-1}$; $^1$H NMR (500 MHz, $CDCl_3$) δ = 7.45 – 7.35 (4H, m, C2*H*, C3*H*, C5*H*, C6*H*), 7.26 – 7.21 (2H, m, C3`*H*, C5`*H*), 7.20 – 7.14 (1H, m, C4`*H*), 7.03 (1H, d, $^3J$ 7.5 Hz, C2`*H*, C6`*H*), 3.51 (3H, s, N–C$H_3$); $^{13}$C NMR (126 MHz, $CDCl_3$) δ = 169.3 (*C*=O), 144.4 (*C1`*), 139.6 (*C1*), 131.4 (q, $^2J_{CF}$ 33.4 Hz, *C4*), 129.6 (*C3`*, *C5`*), 129.1 (*C2*, *C6*), 127.1 (*C4`*), 127.0 (*C2`*, *C6`*), 124.9 (d, $^3J_{CF}$ 3.8 Hz, *C3*, *C5*), 38.5 (*C*$H_3$); $^{19}$F NMR (471 MHz, $CDCl_3$) δ = –62.96; HMRS (ESI) m/z: [M+H$^+$] Calcd. for $C_{15}H_{13}FNO$ 280.0944; Found 280.0944.

## 3-Cyano-*N*-methyl-*N*-phenylbenzamide 3b:

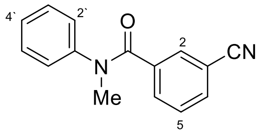


Prepared according to the general procedure **C** using 2-(methyl(phenyl)amino)-2-oxoacetic acid **1a** (71.7 mg, 400 µmol, 2.00 eq.) and 3-bromobenzonitrile (36.4 mg, 200 µmol, 1.00 eq.) to give the title compound as beige oil (7.80 mg, 33.0 µmol 17%,): $R_f$ 0.56 (cyclohexane/EtOAc 1:1); $\nu_{max}$ (neat): 3064w, 3044w, 2934w, 2231m, 1645s, 1593s, 1495s, 1431m, 1370s, 1301m, 1218w, 1150w, 1106w, 1031w, 905w, 814w, 743m, 701s, 631s cm$^{-1}$; $^1$H NMR (500 MHz, $CDCl_3$) δ = 7.57 (1H, s, C2*H*), 7.54 – 7.47 (2H, m, C4*H*, C6*H*), 7.31 – 7.23 (3H, m, C5*H*, C3`*H*, C5`*H*), 7.20 (1H, t, $^3J$ 7.4 Hz, C4`*H*), 7.03 (2H, d, $^3J$ 7.7 Hz, C2`*H*, C6`*H*), 3.51 (3H, s, N–C$H_3$); $^{13}$C NMR (126 MHz, $CDCl_3$) δ = 168.2 (*C*=O), 144.0 (*C1`*), 137.3 (*C1*), 132.9 (*C4*), 132.8 (*C6*), 132.3 (*C2*), 129.6 (*C3`*, *C5`*), 128.7 (*C5*), 127.3 (*C4`*), 126.9 (*C2`*, *C6`*), 118.0 (*C*≡N), 112.2 (*C3*), 38.4 (N–*C*H3); HMRS (ESI) m/z: [M+H$^+$] Calcd. for $C_{15}H_{13}N_2O$ 237.1022; Found 237.1019.

**Methyl 4-(methyl(phenyl)carbamoyl)benzoate 3c:**

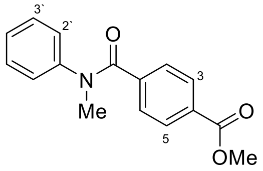


Prepared according to the general procedure **C** using 2-(methyl(phenyl)amino)-2-oxoacetic acid **1a** (71.7 mg, 400 µmol, 2.00 eq.) and methyl 4-bromobenzoate (43.0 mg, 200 µmol, 1.00 eq.) to give the title compound as a white solid (6.50 mg, 24.1 µmol 12%, m.p. 81.6 – 82.0°C): $R_f$ 0.28 (cyclohexane/EtOAc 3:1); $\nu_{max}$ (neat): 3052w, 2991w, 2952w, 1722s, 1644s, 1595m, 1495m, 1434m, 1370m, 1278s, 1178w, 1108m, 1019w, 964w, 865w, 771w, 738w, 700m cm$^{-1}$; $^{1}$H NMR (500 MHz, $CDCl_3$) δ = 7.83 (2H, d, $^{3}J$ 8.4 Hz, C3*H*, C5*H*), 7.35 (2H, d, $^{3}J$ 8.4 Hz, C2*H*, C6*H*), 7.25 – 7.18 (2H, m, C3`*H*, C5`*H*), 7.18 – 7.10 (1H, m, C4`*H*), 7.02 (2H, d, $^{3}J$ 7.5 Hz, C2`*H*, C6`*H*), 3.87 (3H, s, OC*H*3), 3.51 (3H, s, N–C*H*3); $^{13}$C NMR (126 MHz, $CDCl_3$) δ = 169.8 (N–*C*=O), 166.5 (*C*O2Me), 144.4 (*C1`*), 140.4 (*C1*), 130.9 (*C4*), 129.4 (*C3`*, *C5`*), 129.1 (*C3*, *C5*), 128.7 (*C2*, *C6*), 127.05 (*C4`*), 127.03 (*C2`*, *C6`*), 52.3 (O*C*H3), 38.4 (N*C*H3). HMRS (ESI) m/z: [M+H$^{+}$] Calcd. for $C_{16}H_{16}NO_3$ 270.1125; Found 270.1125.

***N*-Methyl-*N*-phenylquinoline-3-carboxamide 3e:**

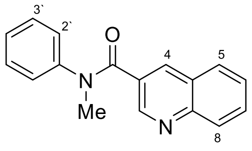


Prepared according to the general procedure **C** using 2-(methyl(phenyl)amino)-2-oxoacetic acid **1a** (71.7 mg, 400 µmol, 2.00 eq.) and 3-bromoquinoline (41.6 mg, 200 µmol, 1.00 eq.) to give the title compound as yellow oil (7.30 mg, 27.8 µmol 14%): $R_f$ 0.28 (cyclohexane/EtOAc 1:1); $\nu_{max}$ (neat): 3060w, 2969w, 2929w, 2843wm 1731w, 1641s, 1593s, 1494s, 1467m, 1422m, 1378s, 1299m, 1260m, 1163w, 1102w, 966w, 918w, 791w, 699m, 633m cm$^{-1}$; $^{1}$H NMR (500 MHz, $CDCl_3$) δ = 8.72 (1H, d, $^{4}J$ 2.1 Hz, C2*H*), 8.20 (1H, d, $^{4}J$ 2.1 Hz, C4*H*), 8.00 (1H, d, $^{3}J$ 8.8 Hz, C8*H*), 7.74 – 7.67 (2H, m, C5*H*, C7*H*), 7.55 – 7.48 (1H, m, C6*H*), 7.27 – 7.21 (2H, m, C3`*H*, C5`*H*), 7.18 – 7.13 (1H, m, C4`*H*), 7.12 – 7.08 (2H, m, C2`*H*, C6`*H*), 3.58 (3H, s, N–C*H*3) ; $^{13}$C NMR (126 MHz, $CDCl_3$) δ = 168.2 (*C*=O), 149.6 (*C2*), 147.9 (*C8a*), 144.4 (*C1`*), 137.4 (*C4*), 130.9 (*C7*), 129.8 (*C3`*, *C5`*), 129.3 (*C8*), 129.0 (*C3*), 128.6 (*C5*), 127.4 (*C4`*), 127.25 (*C6*), 127.23 (*C2`*, *C6`*), 126.9 (*C4a*), 38.7 (N–*C*H3); HMRS (ESI) m/z: [M+H$^{+}$] Calcd. for $C_{17}H_{15}N_2O$ 263.1179; Found 263.1181.

***N*-Methyl-*N*-phenylpyrazine-2-carboxamide 3g:**

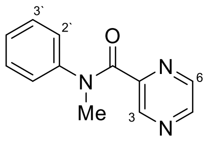


Prepared according to the general procedure **C** using 2-(methyl(phenyl)amino)-2-oxoacetic acid **1a** (71.7 mg, 400 µmol, 2.00 eq.) and 2-bromopyrazine (31.8 mg, 200 µmol, 1.00 eq.) to give the title compound as a white solid (9.70 mg, 45.5 µmol 23%, m.p. 108.0 – 108.5°C): $R_f$ 0.23 (cyclohexane/EtOAc 1:1); $\nu_{max}$ (neat): 3061w, 2956w, 2924w, 2842w, 1660s, 1595m, 1495m, 1423w, 1376m, 1302w, 1209w, 1152w, 1111w, 1018w, 860w, 700w, 631s cm$^{-1}$; $^{1}$H NMR (500 MHz, $CDCl_3$) δ = 8.68 (1H, s, C3*H*), 8.41 (1H, s, C5*H*), 8.31 (1H, s, C6*H*), 7.25 – 6.96 (5H, m, C2`*H*, C3`*H*, C4`*H*, C5`*H*, C6`*H*), 3.54 (3H, s, N–C*H*3); $^{13}$C NMR (126 MHz, $CDCl_3$) δ = 166.7 (*C*=O), 150.0 (*C2*), 144.9 (*C3*), 144.6 (*C5*), 143.7 (*C1`*), 143.2 (*C6*), 129.4 (*C3`*, *C5`*), 127.3 (*C2`*, *C6`*), 127.0 (*C4`*), 38.1 (N–C*H*3); HMRS (ESI) m/z: [M+H$^{+}$] Calcd. for $C_{12}H_{12}N_3O$ 214.0975; Found 214.0974.

## *N*-Methyl-*N*-(*p*-tolyl)-9*H*-carbazole-3-carboxamide 3h:

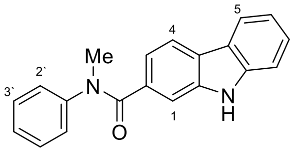


Prepared according to the general procedure **C** using 2-(methyl(phenyl)amino)-2-oxoacetic acid **1a** (71.7 mg, 400 µmol, 2.00 eq.) and 2-bromo-9*H*-carbazole (49.2 mg, 200 µmol, 1.00 eq.) to give the title compound as a white solid (29.0 mg, 96.6 µmol, 48%, m.p. 185.0 – 185.4°C): $R_f$ 0.43 (cyclohexane/EtOAc 1:1); $\nu_{max}$ (neat): 3271w, 3244w, 3180w, 3065w, 2901w, 1618s, 1587s, 1495s, 1443m, 1371m, 1328m, 1243m, 1155w, 1102w, 1030w, 908m, 821w, 731m, 698w; $^1$H NMR (500 MHz, $CDCl_3$) δ = 8.42 (1H, s, N*H*), 8.12 – 7.92 (1H, s, C5*H*), 7.79 (1H, d, $^3J$ 8.1 Hz, C4*H*), 7.57 – 7.52 (1H, m, C1*H*), 7.43 – 7.35 (2H,m, C7*H*, C8*H*), 7.20 – 7.13 (3H, m, C6*H*, C3`*H*, C5`*H*), 7.12 – 6.99 (4H, m, C3*H*, C2`*H*, C4`*H* C6`*H*), 3.55 (3H, N–C*H*3); $^{13}$C NMR (126 MHz, $CDCl_3$) δ = 171.5 (*C*=O) 14543 (*C1`*), 140.5 (*C8a*), 138.8 (*C9a*), 133.3 (*C2*), 129.2 (*C3`*, *C5`*), 126.9 (*C2`*, *C6`*), 126.6 (*C7*), 126.4 (*C4`*), 124.4 (*C4a*), 122.8 (*C4b*), 120.7 (*C5*), 120.3 (*C3*), 119.7 (*C6*), 119.4 (*C4*), 111.9 (*C1*), 110.9 (*C8*), 38.8 (*C*$H_3$).; HMRS (ESI) m/z: [M+H$^+$] Calcd. for $C_{20}H_{17}N_2O$ 301.1335; Found 301.1341.

### Methodology limitations

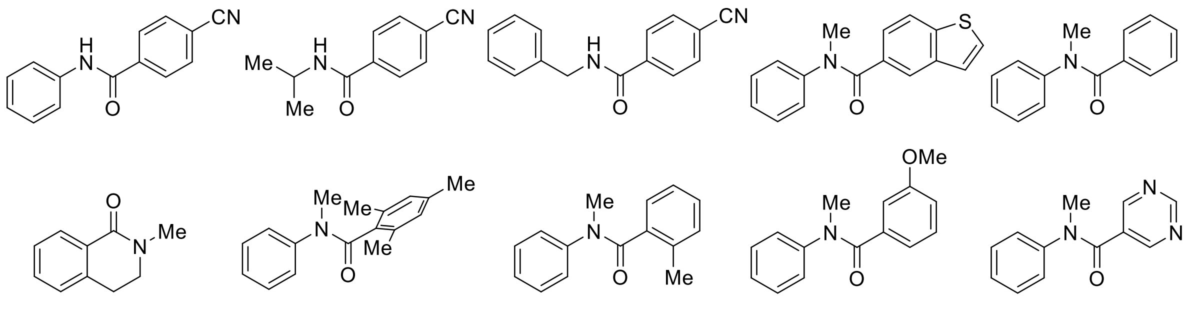

# 5. Synthesis of Deuterated Formamides: Optimization Studies

**General optimization procedure for Table S5:**

2-(Methyl(phenyl)amino)-2-oxoacetic acid **1a** (17.9 mg, 100 µmol, 1.00 eq.), $Li_2CO_3$ (7.39 mg, 100 µmol, 1.00 eq.) and the photocatalyst were transferred to 4 mL vial. The mixture was degassed via vacuum/argon cycles (3 times). Then DMF (2.0 mL), degassed $D_2O$ and thiol were added under argon. The reaction mixture was irradiated with a Kessil tuna blue lamp ($\lambda_{max}$ = 464 nm) with fan cooling for 22 h, then diluted with EtOAc and $H_2O$. The aqueous phase was extracted with EtOAc (3×15 mL). The combined organic phase was washed with brine (3×20 mL), dried over $Na_2SO_4$ and the solvent was removed under reduced pressure.

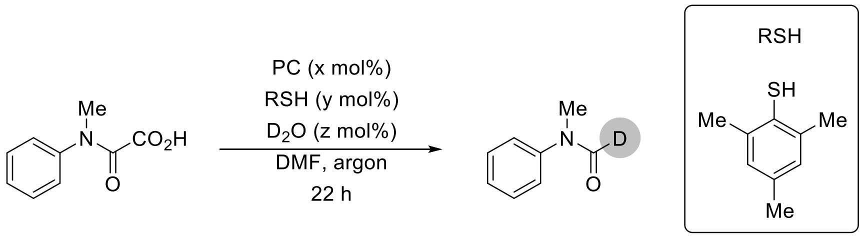


**Table S5.** Reaction condition optimization for the preparation of deuterated formamides

| Entry[a] | Thiol (eq.) | PC | PC (mol%) | $D_2O$ (eq.) | Yield (%)[b] | D incorporation (%) |
|---|---|---|---|---|---|---|
| 1 | 0.1 | $(Ir[dF(CF_3)ppy]_2(dtbpy))PF_6$ | 3% | 28 eq. | 37% | 73% |
| 2 | 0.1 | $(Ir[dF(CF_3)ppy]_2(dtbpy))PF_6$ | 3% | 56 eq. | 40% | 87% |
| 3 | 0.1 | 4CzIPN | 3% | 28 eq. | 22% | 81% |
| 4 | 0.1 | 4CzIPN | 3% | 56 eq. | 43% | 90% |
| 5 | 0.1 | 4CzIPN | 3% | 112 eq. | 11% | 92% |
| 6 | 0.3 | 4CzIPN | 3% | 56 eq. | 35% | 87% |
| 7 | 0.6 | 4CzIPN | 3% | 56 eq. | 32% | 86% |
| 8 | 0.1 | – | – | 56 eq. | 0% | 0% |
| 9 | 0.1 | 4CzIPN | 3% | – | 44% | 0% |
| 10 | – | 4CzIPN | 3% | 56 eq. | 0% | 0% |

[a]**Reaction conditions: 1a** (17.9 mg, 100 µmol, 1.00 eq.), 2,4,6-trimethylthiophenol (see the table), PC (see the table), $D_2O$ (see the table) in DMF (2.0 mL), Ar, 25°C, Kessil tuna blue ($\lambda_{max}$ = 464 nm), 22 h. [b]Yield was estimated by the 1H-NMR analysis using durene as an internal standard.

**General procedure D for the synthesis of deuterated formamides 4a-4d:**

The corresponding oxamic acid **1a**/**1b**/**1d**/**1e** (300 µmol, 1.00 eq.), $Li_2CO_3$ (22.2 mg, 300 µmol, 1.00 eq.), 4CzIPN (7.10 mg, 9.00 µmol, 3 mol%) were transferred to a 7 mL vial. The mixture was degassed via vacuum/argon cycles (3 times). Then DMF (6.0 mL), degassed $D_2O$ (300 µL, 16.8 mmol, 56.0 eq.) and 2,4,6-trimethylthiophenol (4.57 mg, 30.0 µmol, 10 mol%) were added under argon. The reaction mixture was irradiated with a Kessil tuna blue lamp ($\lambda_{max}$ = 464 nm) with fan cooling for 22 h, then diluted with EtOAc and $H_2O$. The aqueous phase was extracted with EtOAc (3×15 mL). The combined organic phase was washed with brine (3×20 mL), dried over $Na_2SO_4$ and the solvent was removed under reduced pressure. The residue was purified by silica gel column chromatography (cyclohexane/EtOAc from 1:0 to 3:1) to yield the desired product.

## *N*-Methyl-*N*-phenylformamide-*d* 4a:

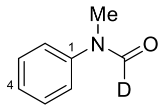


Prepared according to the general procedure **D** using 2-(methyl(phenyl)amino)-2-oxoacetic acid **1a** (53.8 mg, 300 µmol) to give the title compound as yellow oil (10.0 mg, 73.4 µmol, 25%): $R_f$ 0.20 (cyclohexane/EtOAc 3:1); $\nu_{max}$ (neat): 2956w, 2921w, 2845w, 2229w, 1641s, 1491m, 1369w, 1308w, 1261w, 1096s, 1014s, 833m, 799m, 719w cm$^{-1}$; $^1$H NMR (500 MHz, $CDCl_3$) δ = 8.48 (0.1 H, 90% D, s, C(=O)*H*), 7.46 – 7.36 (2H, m, C3*H*, C5*H*), 7.32 – 7.26 (1H, m, C4*H*), 7.21 – 7.14 (2H, m, C2*H*, C6*H*), 3.33 (3H, s, N–C*H*$_3$); $^{13}$C NMR

(126 MHz, $CDCl_3$) δ = 162.5 (*C*(=O)H), 162.1 (d, $^1J_{CD}$ 30.4 Hz, *C*(=O)D), 142.3 (*C1*), 129.8 (*C3*, *C5*), 126.5 (*C4*), 122.5 (*C2*, *C6*), 32.2 (N–*C*$H_3$). HMRS (ESI) m/z: [M+$Na^+$] Calcd. for $C_8H_8DNNaO$ 159.0639; Found 159.0639.

### ***N*-Methyl-*N*-(*p*-tolyl)formamide-*d* 4b:**

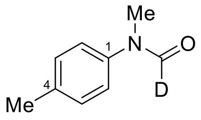


Prepared according to the general procedure **D** using 2-(methyl(*p*-tolyl)amino)-2-oxoacetic acid **1b** (58.0 mg, 300 μmol) to give the title compound as yellow oil (17.5 mg, 117 μmol 39%): $R_f$ 0.25 (cyclohexane/EtOAc 3:1); $\nu_{max}$ (neat): 3035w, 2963w, 2922w, 2174w, 1654s, 1515m, 1452w, 1411w, 1348w, 1264w, 1113m, 1077w, 932w, 818w $cm^{-1}$; $^1$H NMR (500 MHz, $CDCl_3$) δ = 8.42 (0.09 H, 91% D, s, C(=O)*H*), 7.24 – 7.17 (2H, m, C3*H*, C5*H*), 7.12 – 7.03 (2H, m, C2*H*, C6*H*), 3.29 (3H, s, N–C$H_3$), 2.36 (3H, s, C4–C$H_3$); $^{13}$C NMR (126 MHz, $CDCl_3$) δ = 16254 (*C*(=O)H), 162.03 (d, $^1J_{CD}$ 30.1 Hz, *C*(=O)D), 139.8 (*C1*), 136.5 (*C4*), 130.3 (*C3*, *C5*), 122.6 (*C2*, *C6*), 32.3 (N–*C*$H_3$), 21.0 (C4–*C*$H_3$); HMRS (ESI) m/z: [M+$Na^+$] Calcd. for $C_9H_{10}DNNaO$ 173.0796; Found 173.0784.

### ***N*-(4-Fluorophenyl)-*N*-methylformamide-*d* 4c:**

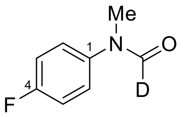


Prepared according to the general procedure **D** using 2-((4-fluorophenyl)(methyl)amino)-2-oxoacetic acid **1d** (59.1 mg, 300 μmol) to give the title compound as yellow oil (12.0 mg, 77.8 μmol 26%): $R_f$ 0.18 (cyclohexane/EtOAc 3:1); $\nu_{max}$ (neat): 3070w, 2970w, 2921w, 2174w, 1649s, 1508s, 1412w, 1347m, 1224s, 1160w, 1112m, 1013w, 933m, 835s $cm^{-1}$; $^1$H NMR (500 MHz, $CDCl_3$) δ = 8.39 (0.08 H, 92% D, s, C(=O)*H*), 7.21 – 6.99 (4H, m, C2*H*, C3*H*, C5*H*, C6*H*), 3.29 (3H, s, C$H_3$); $^{13}$C NMR (126 MHz, $CDCl_3$) δ = 162.3 – 161.7 (m, *C*(=O)H, *C*(=O)D), 161.1 (d, $^1J_{CF}$ 247.0 Hz, *C4*), 138.4 (*C1*), 124.7 (d, $^3J_{CF}$ 8.3 Hz, *C2*, *C6*), 116.6 (d, $^2J_{CF}$ 23.2 Hz, *C3*, *C5*), 32.6 (*C*$H_3$); $^{19}$F NMR (471 MHz, $CDCl_3$) δ = –115.3. HMRS (ESI) m/z: [M+$H^+$] Calcd. for $C_8H_8DFNO$ 155.0725; Found 155.0725 [M+$H^+$].

### ***N*,*N*-Diphenylformamide-*d* 4d:**

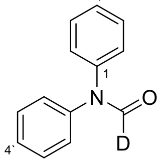


Prepared according to the general procedure **D** using 2-(diphenylamino)-2-oxoacetic acid **1e** (72.4 mg, 300 μmol) to give the title compound as a white solid (13.0 mg, 65.6 μmol 22%, m.p. 63.0 – 63.6°C): $R_f$ 0.48 (cyclohexane/EtOAc 3:1); $\nu_{max}$ (neat): 2969w, 2895w, 2199w, 1698m, 1658s, 1589s, 1334m, 1274s, 1153w, 1076w, 1037m, 848w, 803w, 760m $cm^{-1}$; $^1$H NMR (500 MHz, $CDCl_3$) δ = 8.67 (0.09 H, 91% D, s, C(=O)*H*), 7.44 – 7.37 (4H, m, C3*H*, C5*H*, C3`*H*, C5`*H*), 7.34 – 7.27 (4H, m, C4*H*, C2`*H*, C4`*H*, C6`*H*), 7.17 (2H, d, $^3J$ 7.6 Hz, C2*H*, C6*H*); $^{13}$C NMR (126 MHz, $CDCl_3$) δ = 161.9 (*C*(=O)H), 161.4 (d, $^1J_{CD}$ 30.2 Hz, *C*(=O)D), 141.9 (*C1*), 139.7 (*C1`*), 129.8 (*C3*, *C5*), 129.3 (*C3`*, *C5`*), 127.2 (*C4*), 127.0 (*C4`*), 126.2 (*C2`*, *C6`*), 125.2 (*C2*, *C6*); HMRS (ESI) m/z: [M+$H^+$] Calcd. for $C_{13}H_{11}DNO$ 199.0976; Found 199.0975.

# 6. NMR Data

## Methyl 2-(methyl(phenyl)amino)-2-oxoacetate S1a:

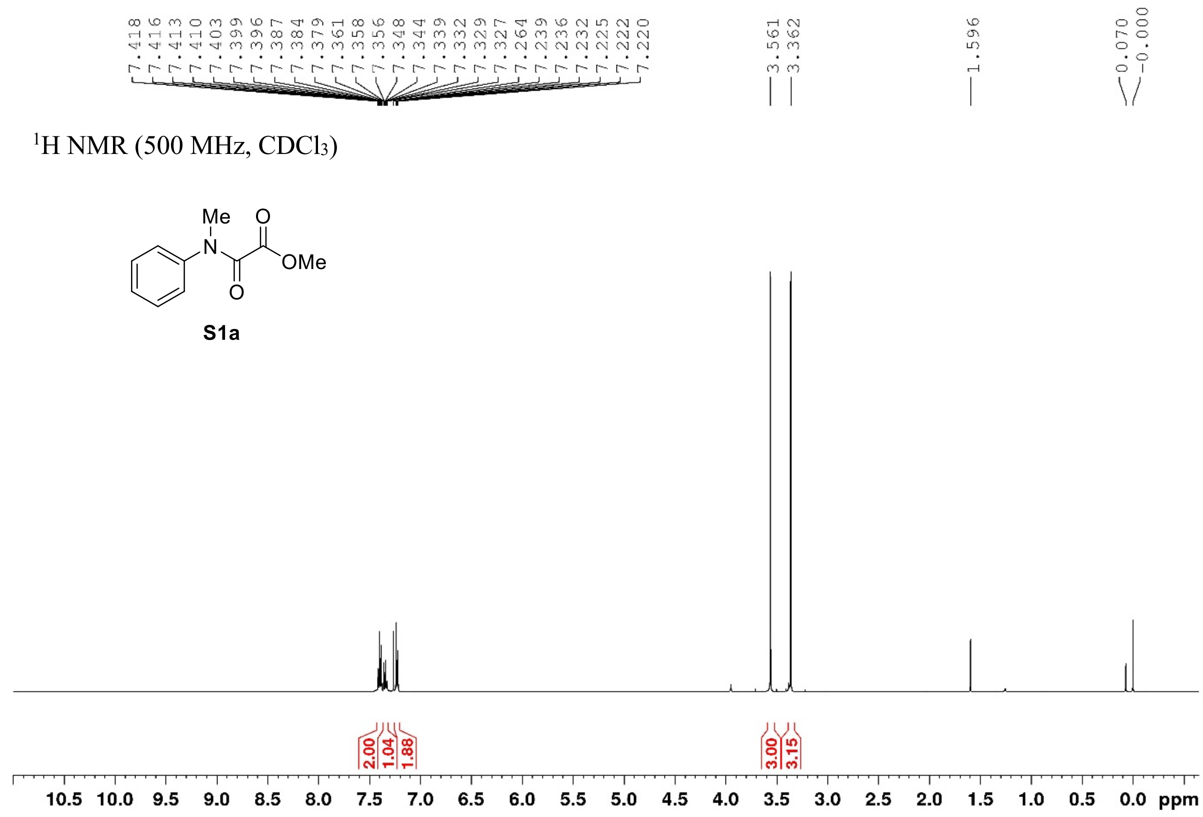


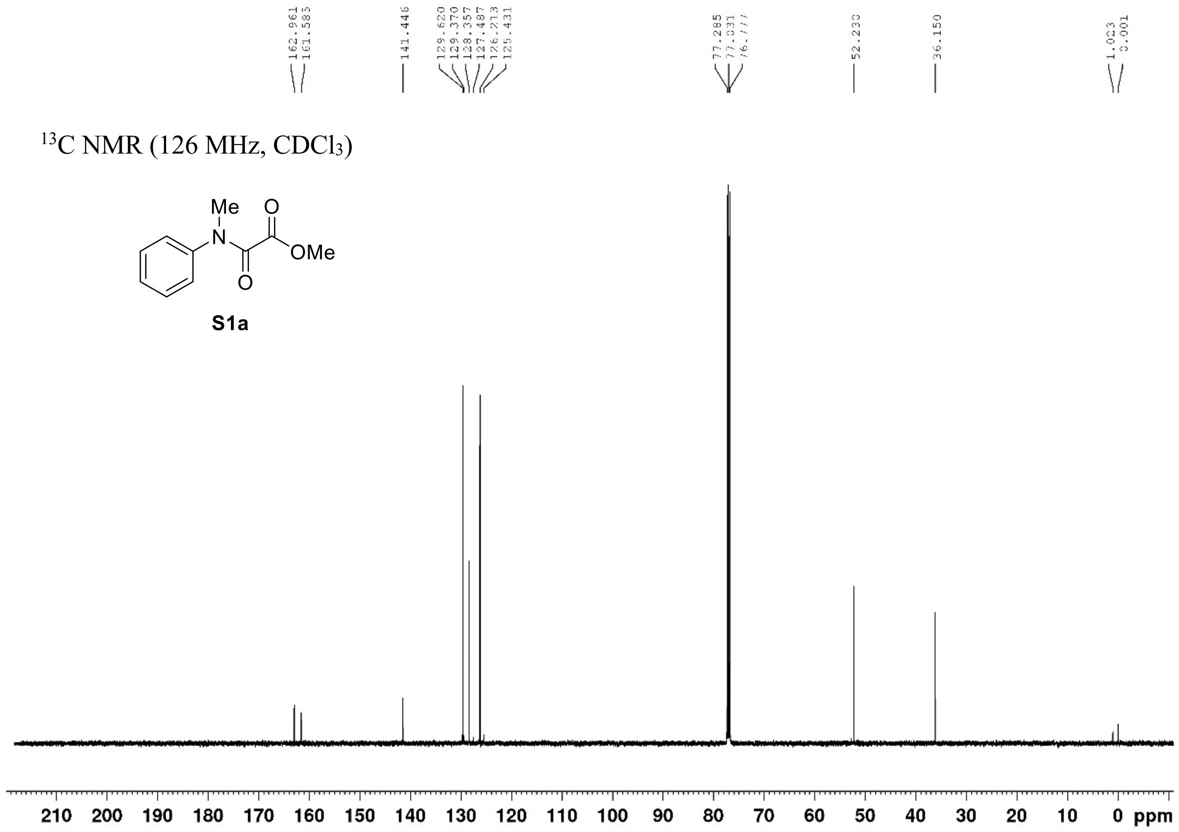

**2-(Methyl(phenyl)amino)-2-oxoacetic acid 1a:**

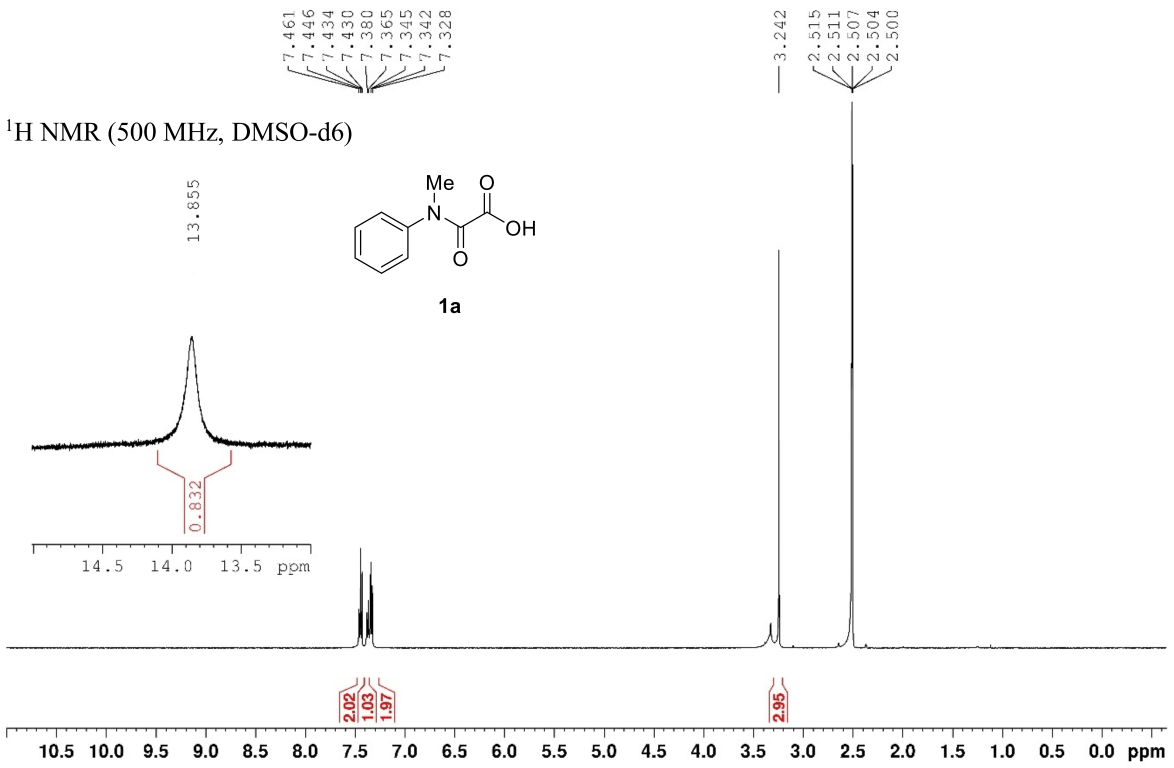


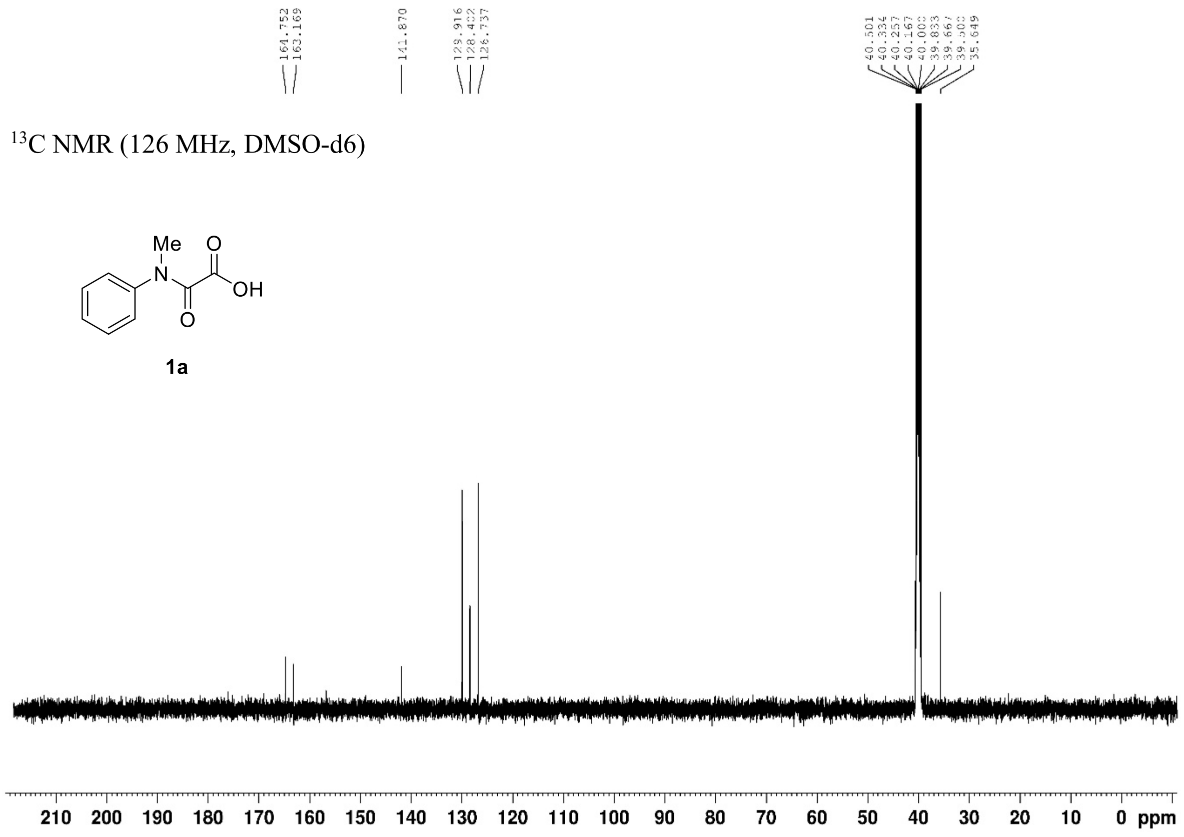

**Methyl 2-(methyl(p-tolyl)amino)-2-oxoacetate S1b:**

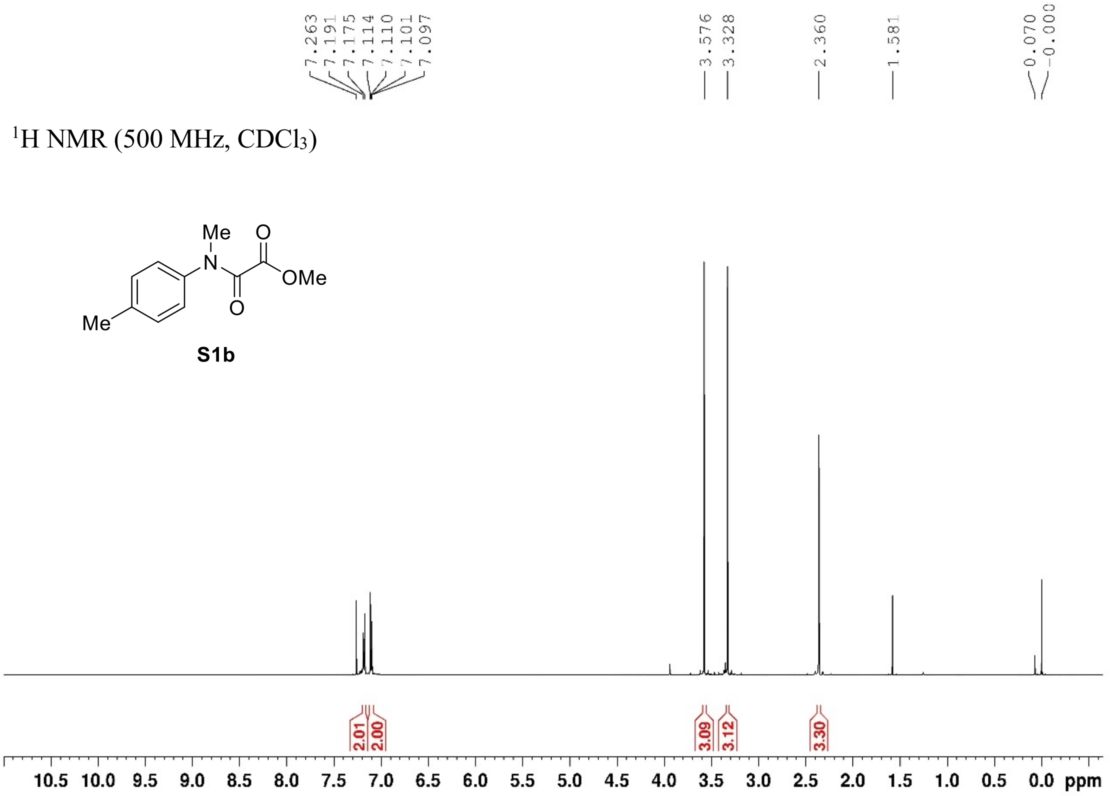


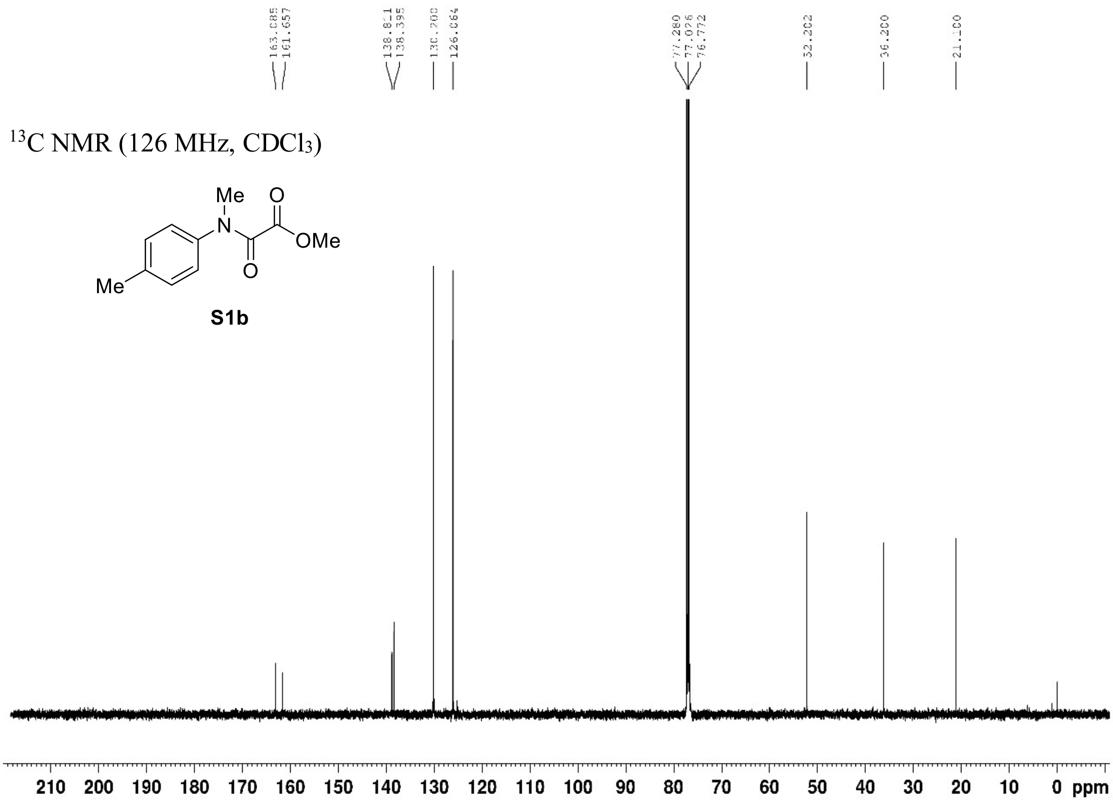

**2-(Methyl(*p*-tolyl)amino)-2-oxoacetic acid 1b:**

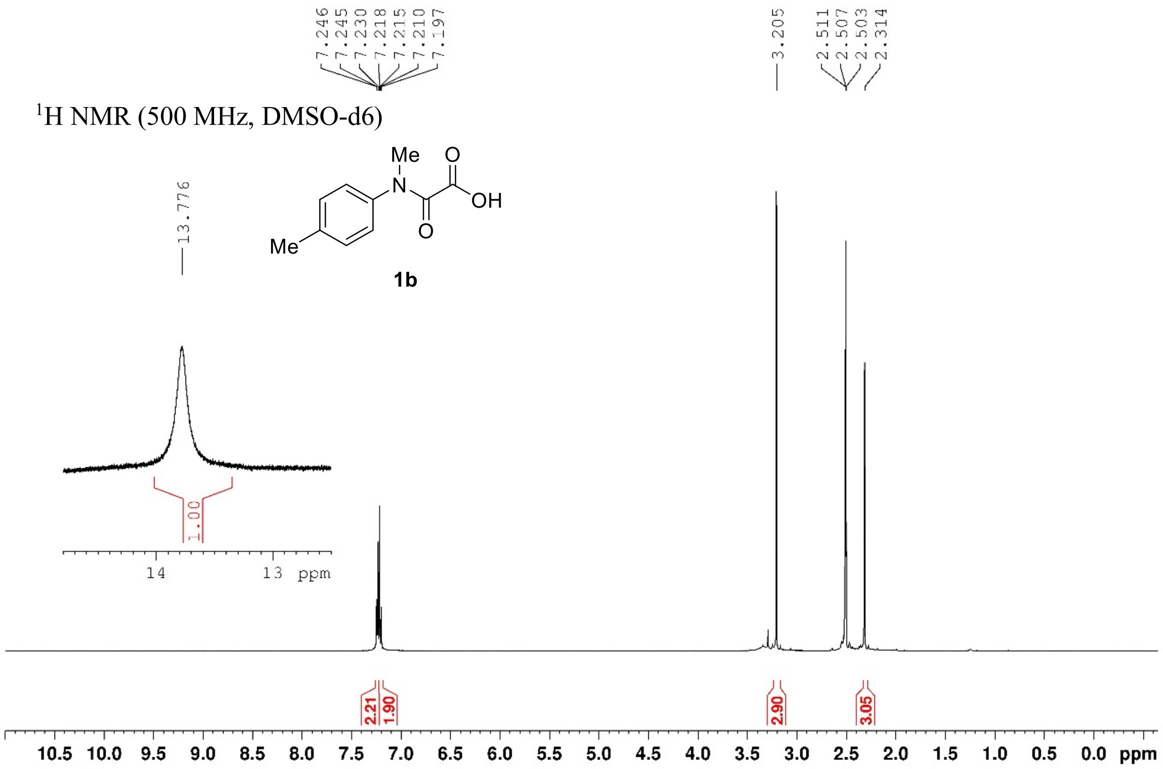


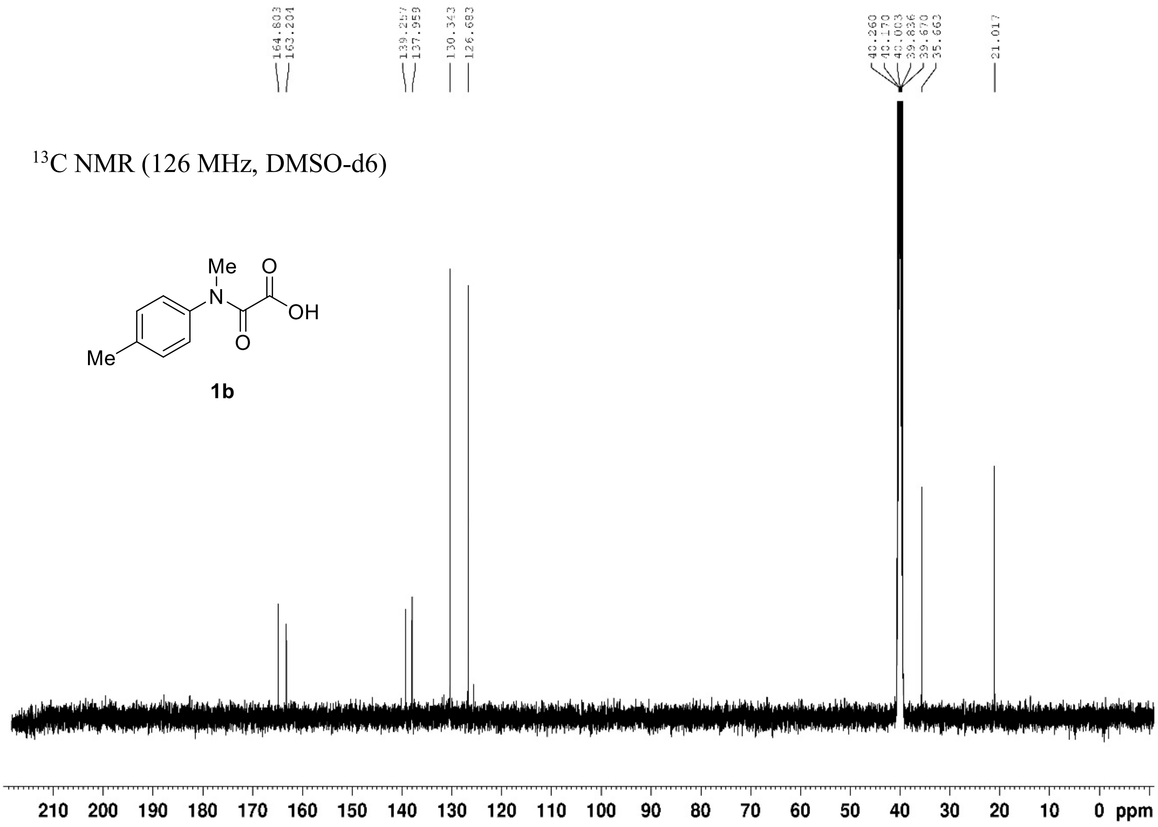

**Methyl 2-((4-chlorophenyl)(methyl)amino)-2-oxoacetate S1c:**

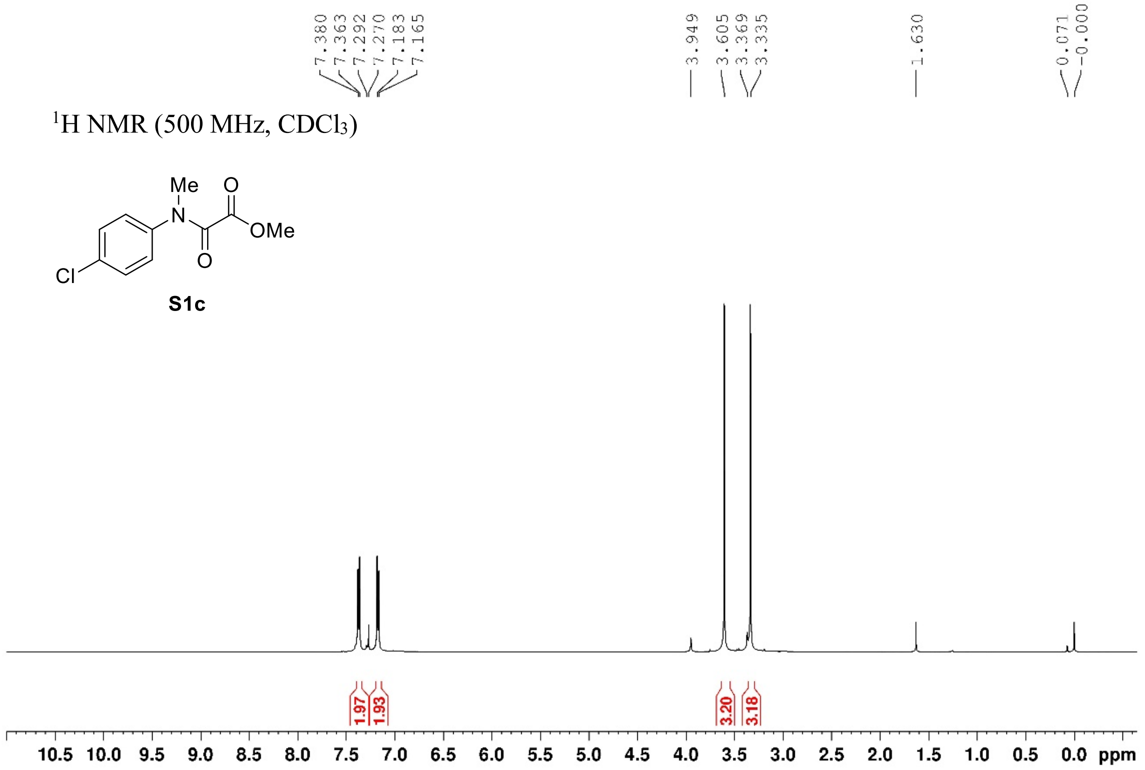


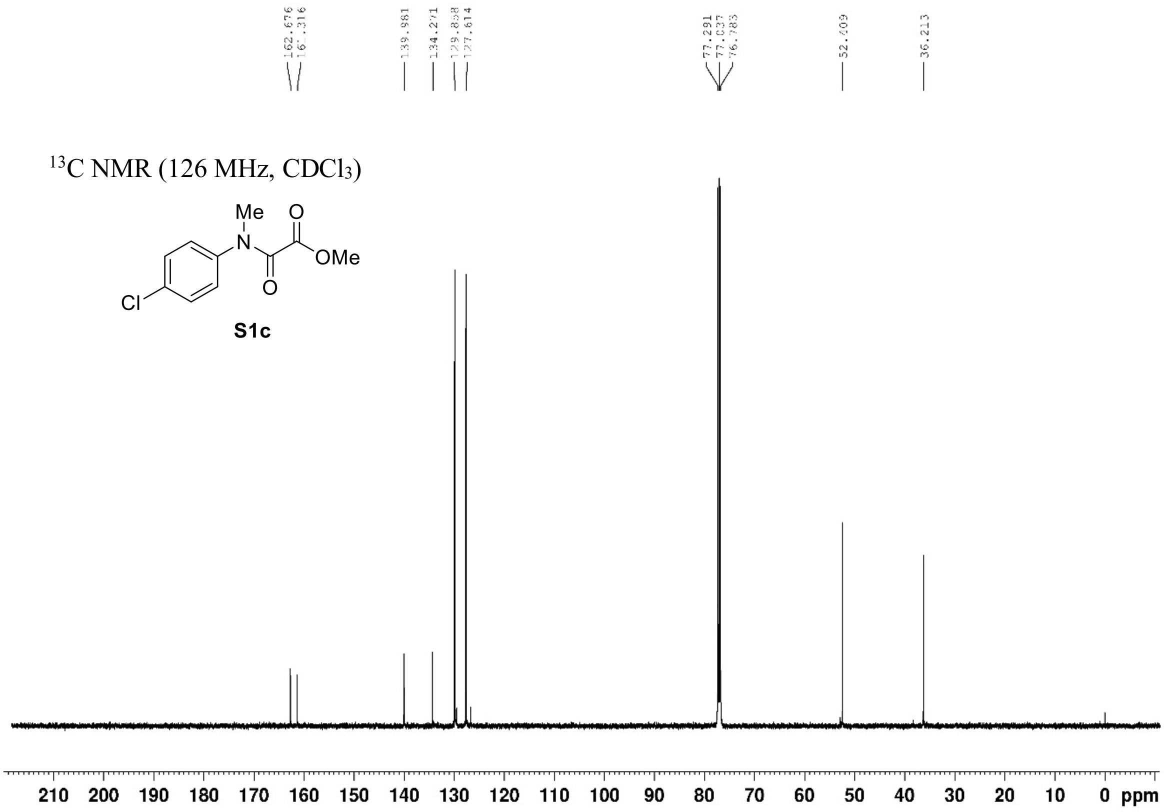

**2-((4-Chlorophenyl)(methyl)amino)-2-oxoacetic acid 1c:**

$^{1}$H NMR (500 MHz, DMSO-d6)

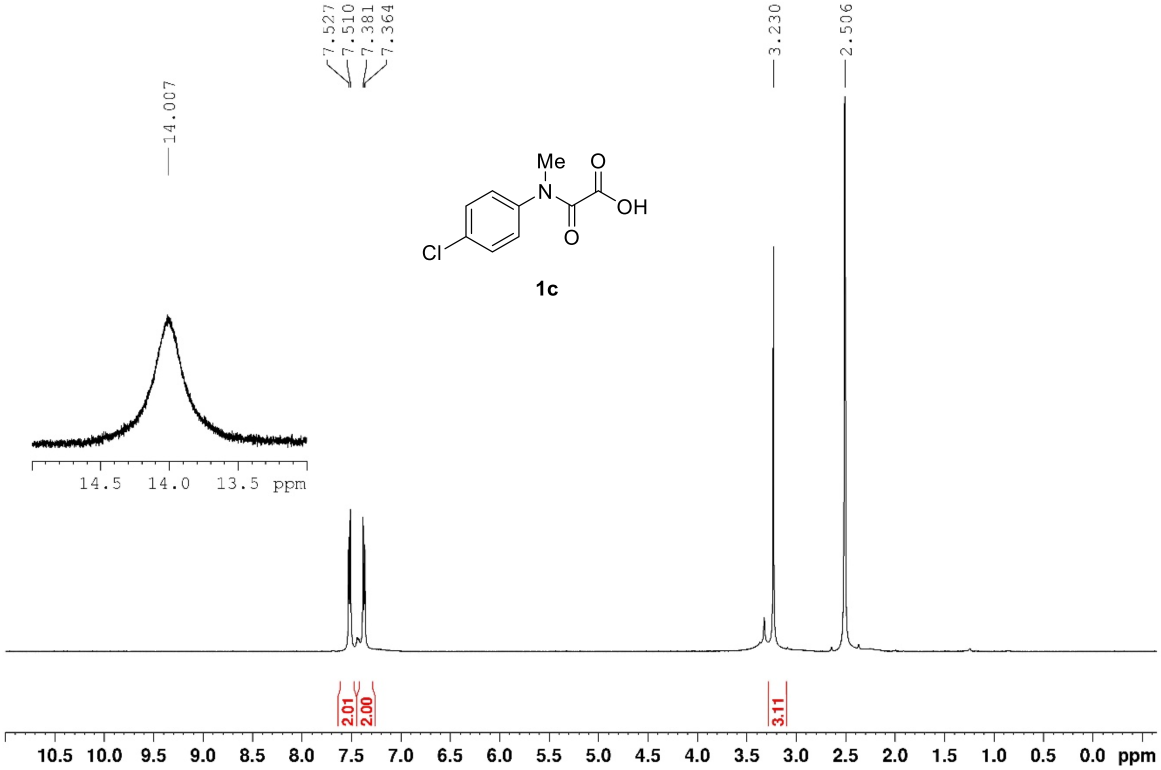


$^{13}$C NMR (126 MHz, DMSO-d6)

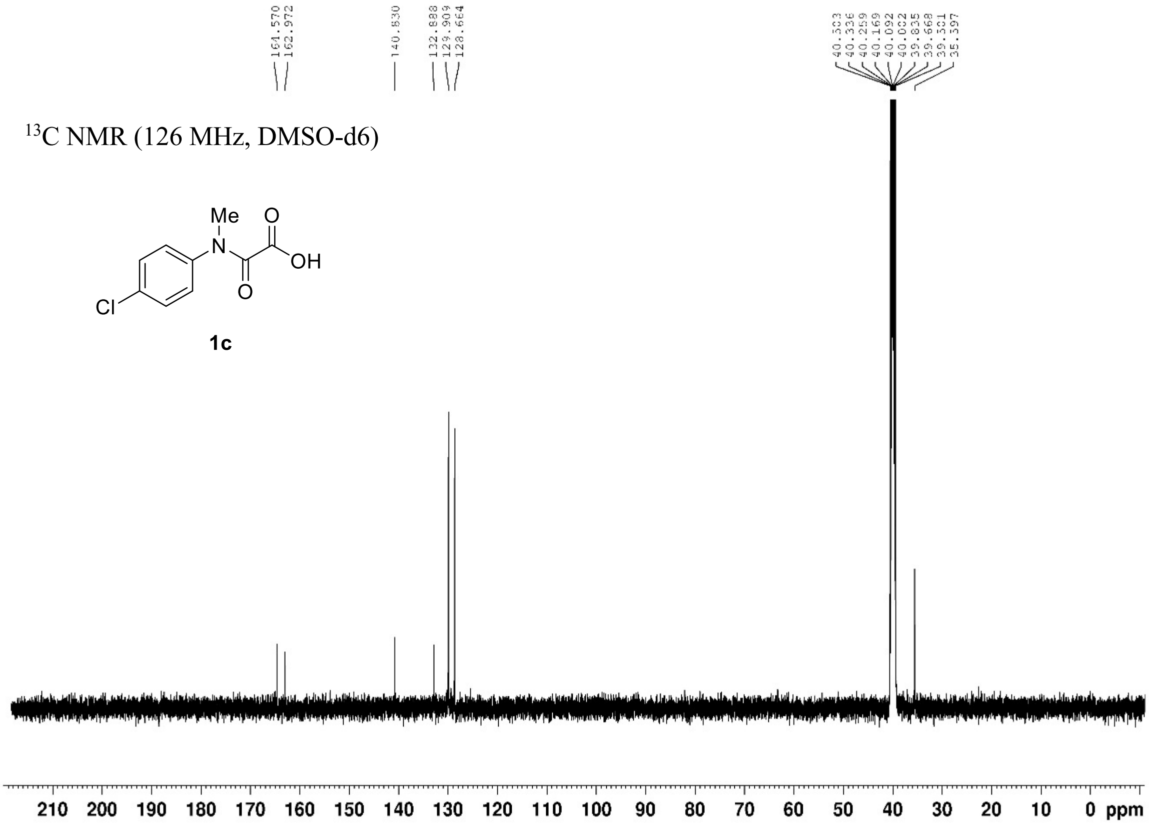

**Methyl 2-((4-fluorophenyl)(methyl)amino)-2-oxoacetate S1d:**

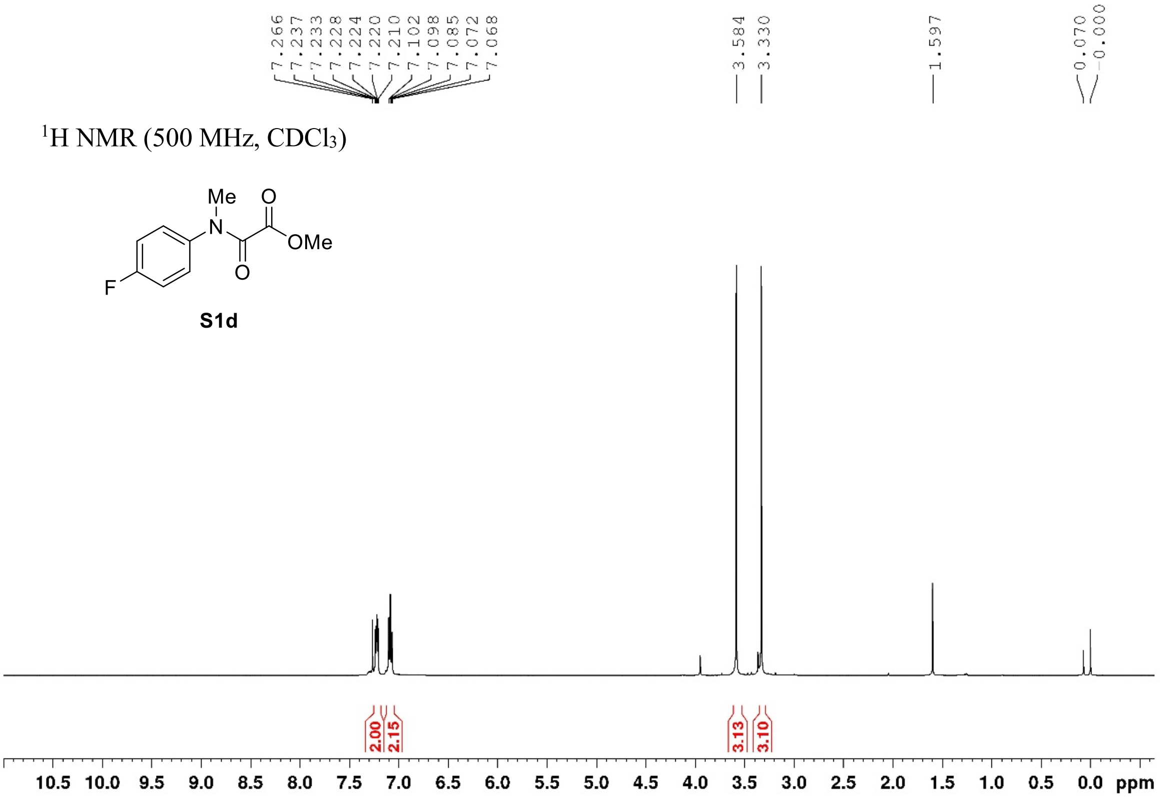


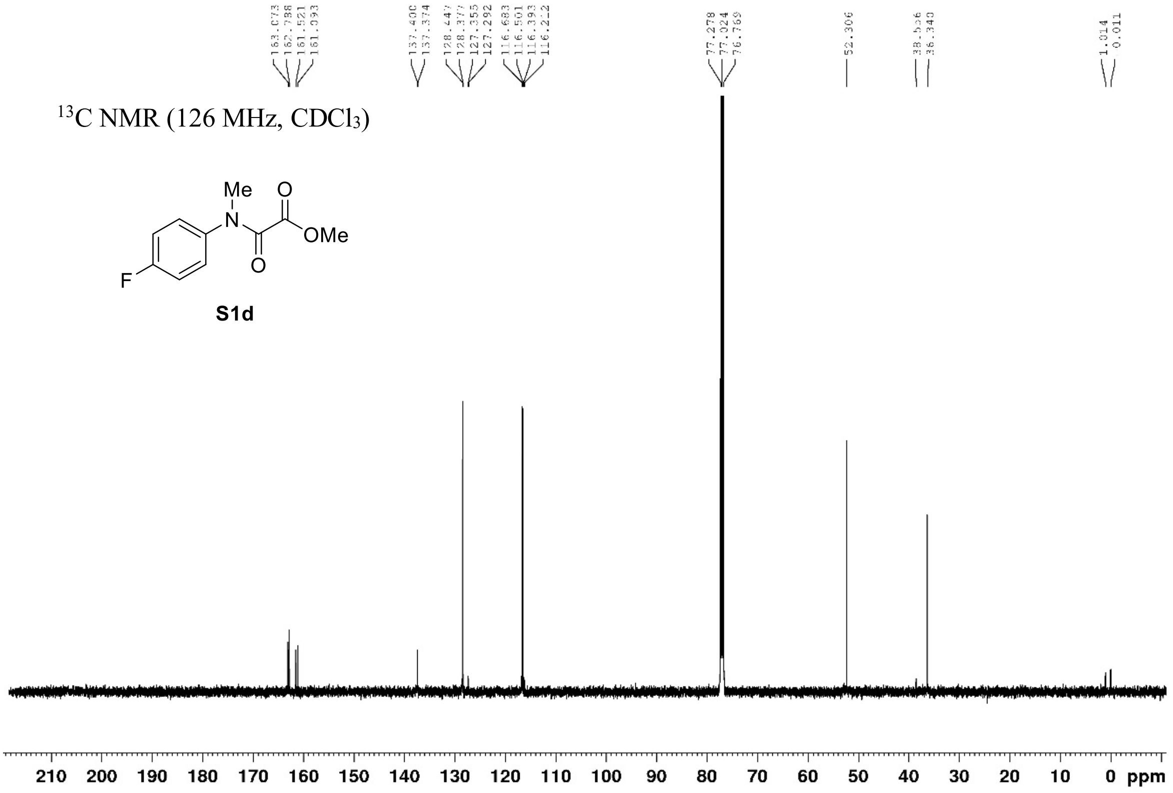

## 2-((4-Fluorophenyl)(methyl)amino)-2-oxoacetic acid 1d:

$^{1}$H NMR (500 MHz, DMSO-d6)

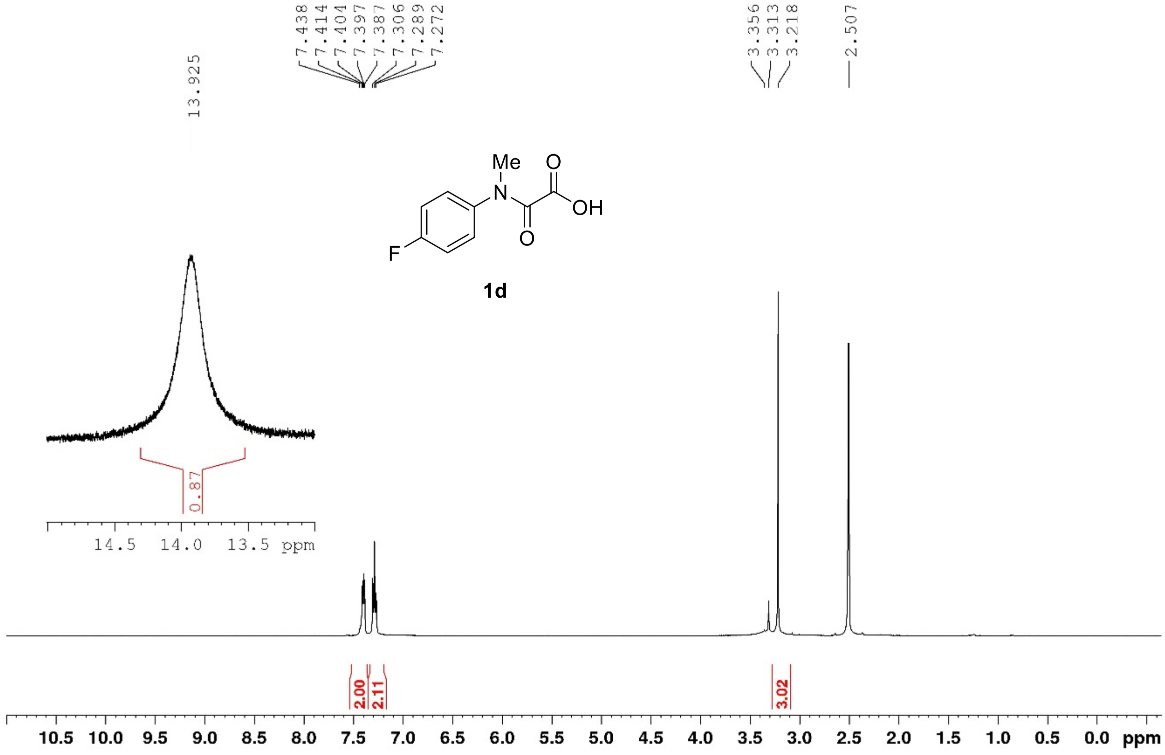


$^{13}$C NMR (126 MHz, DMSO-d6)

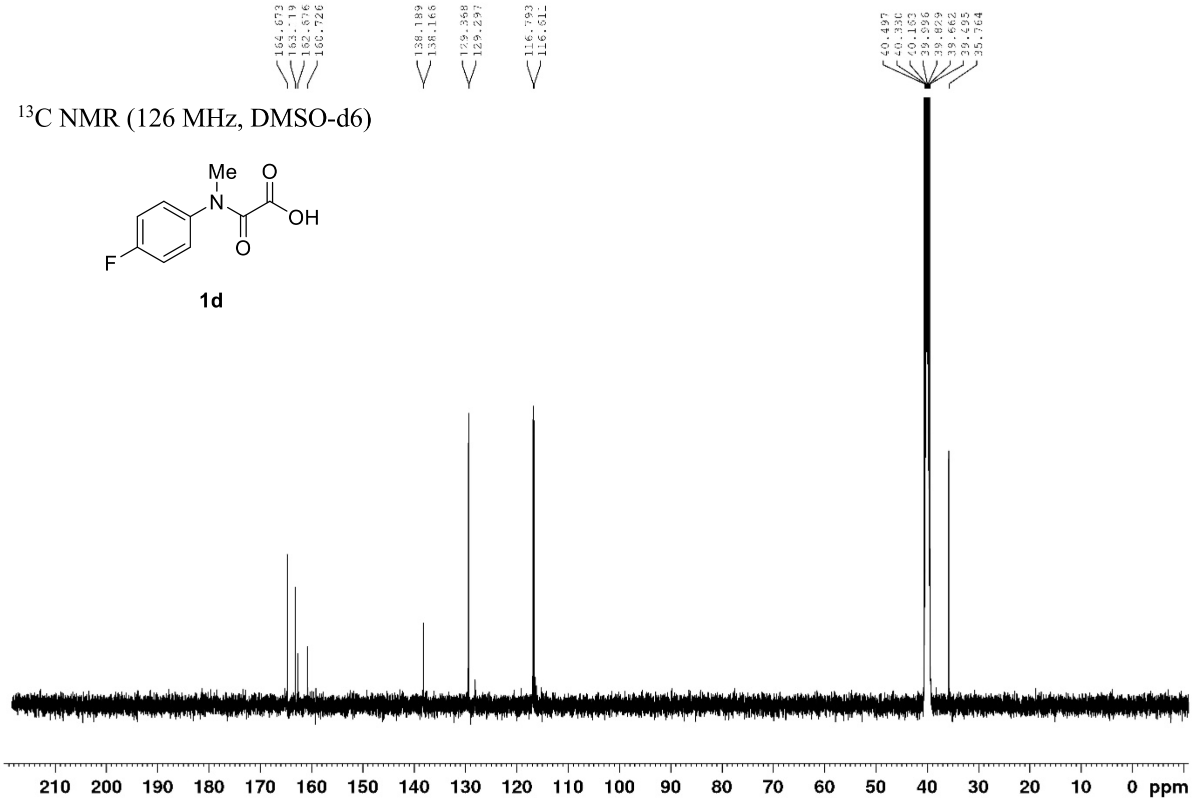

**Methyl 2-(diphenylamino)-2-oxoacetate S1e:**

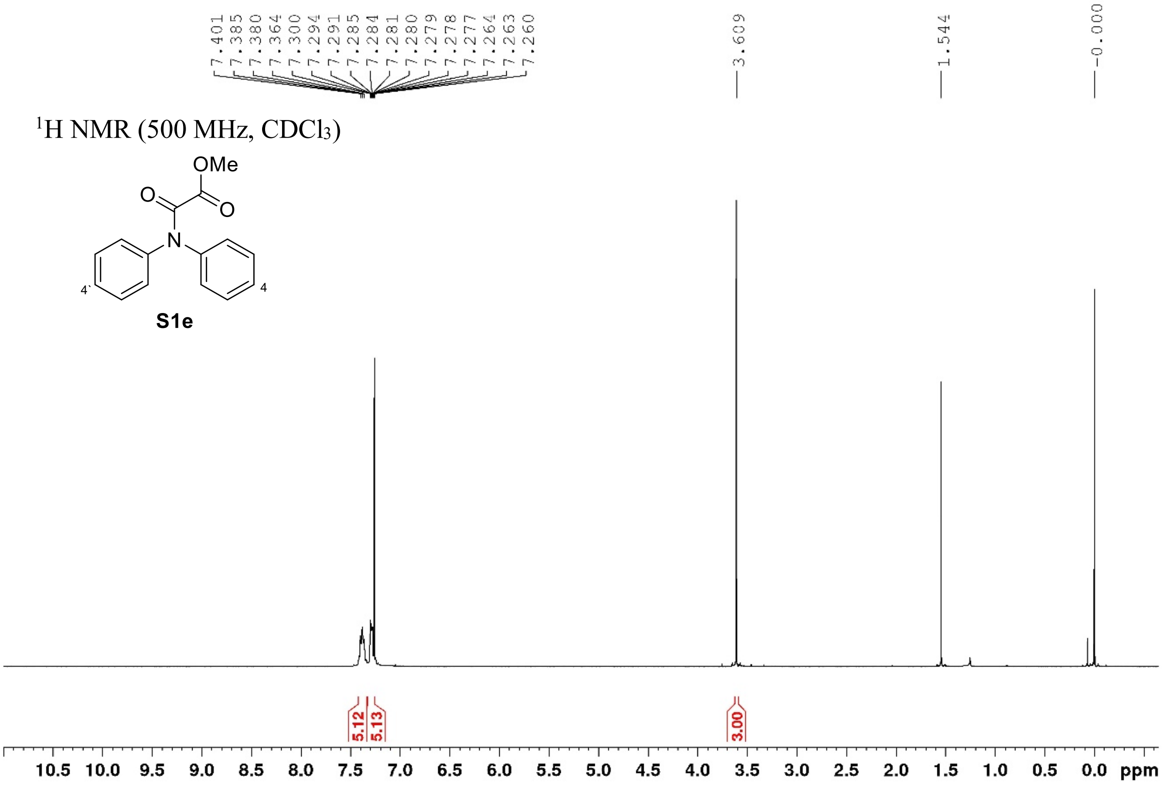


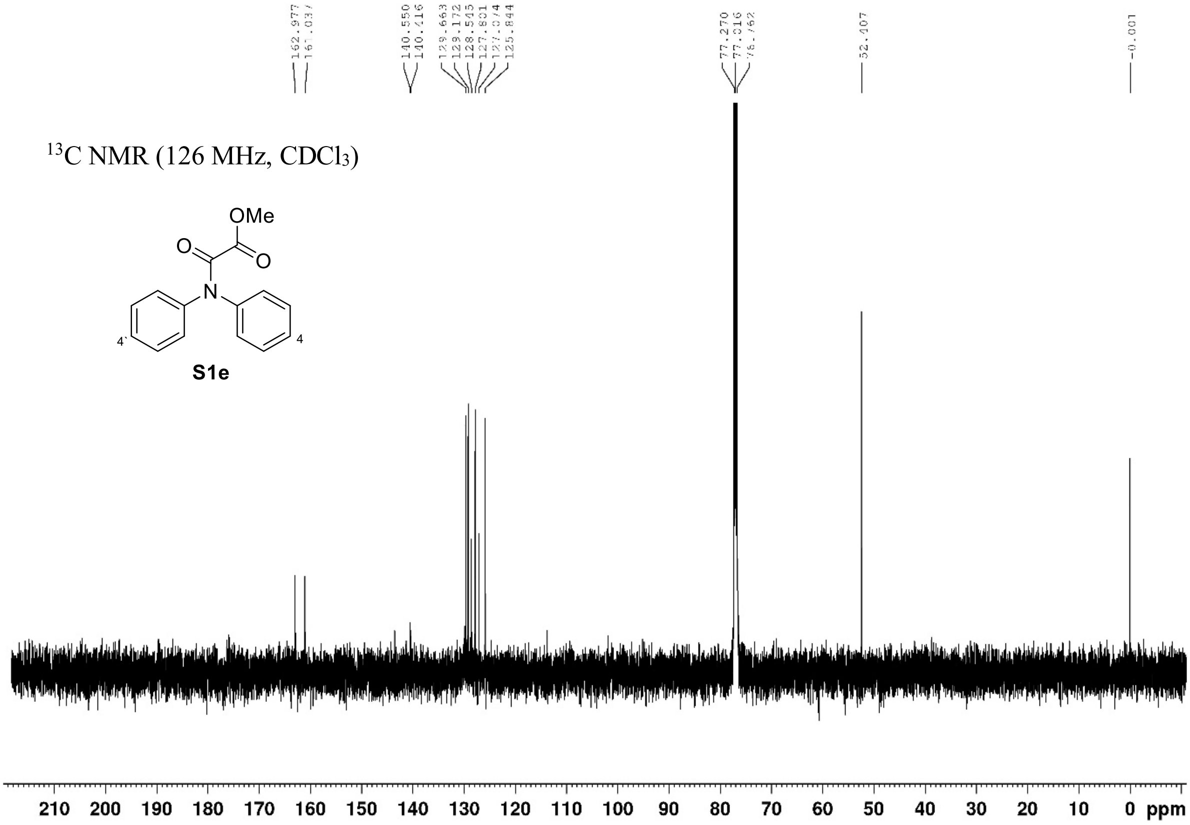

**2-(Diphenylamino)-2-oxoacetic acid 1e:**

$^{1}$H NMR (500 MHz, DMSO-d6)

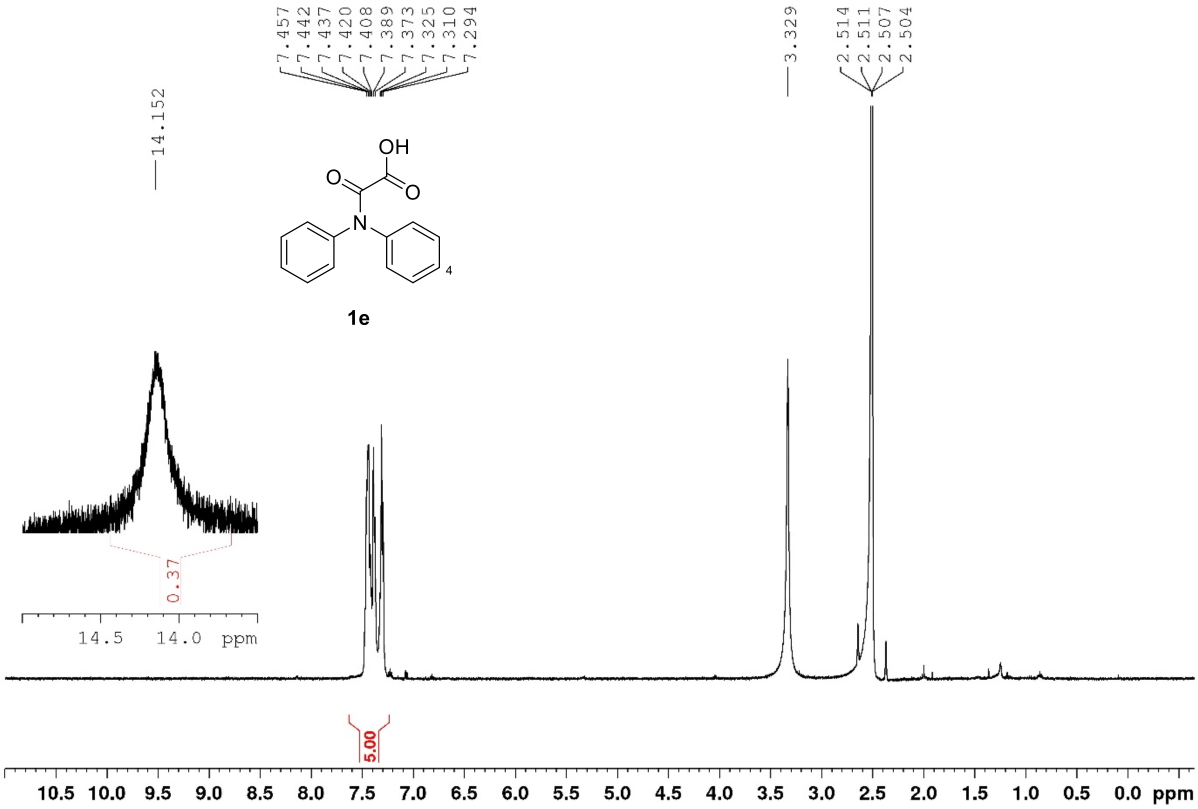


$^{13}$C NMR (126 MHz, DMSO-d6)

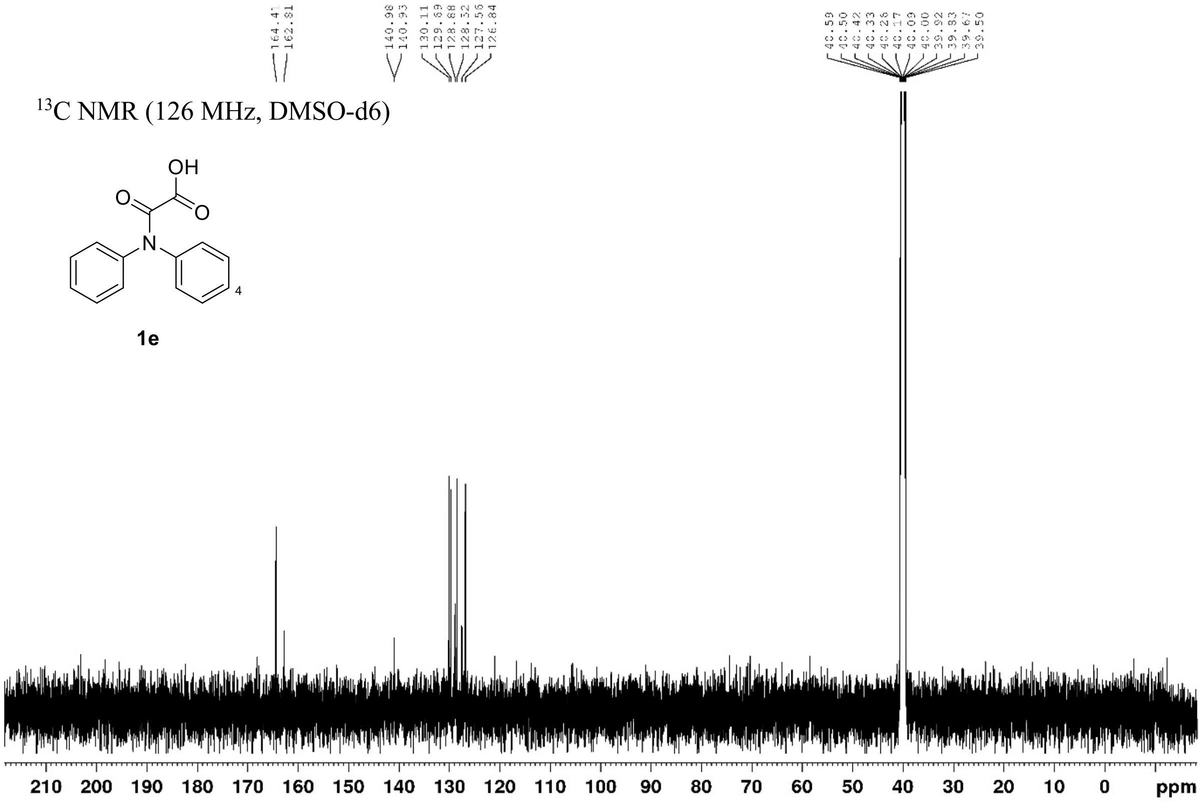

**Methyl 2-morpholino-2-oxoacetate S1f:**

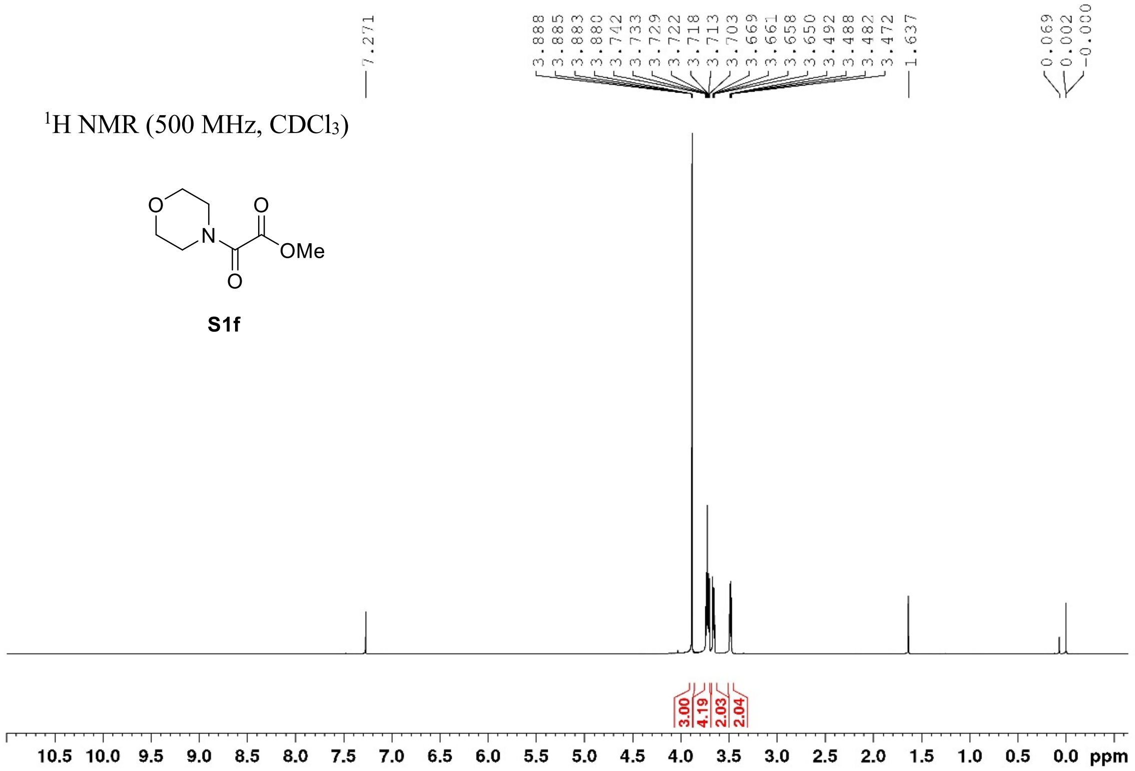


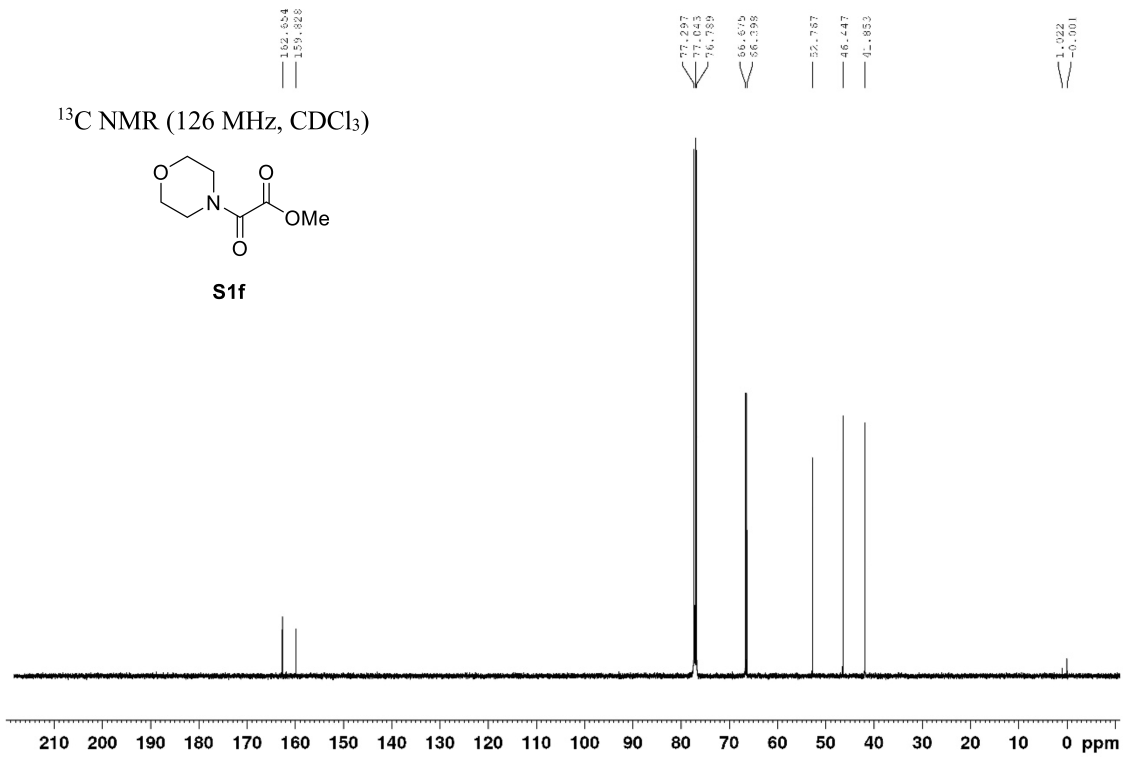

**2-Morpholino-2-oxoacetic acid 1f:**

$^{1}$H NMR (500 MHz, DMSO-d6)

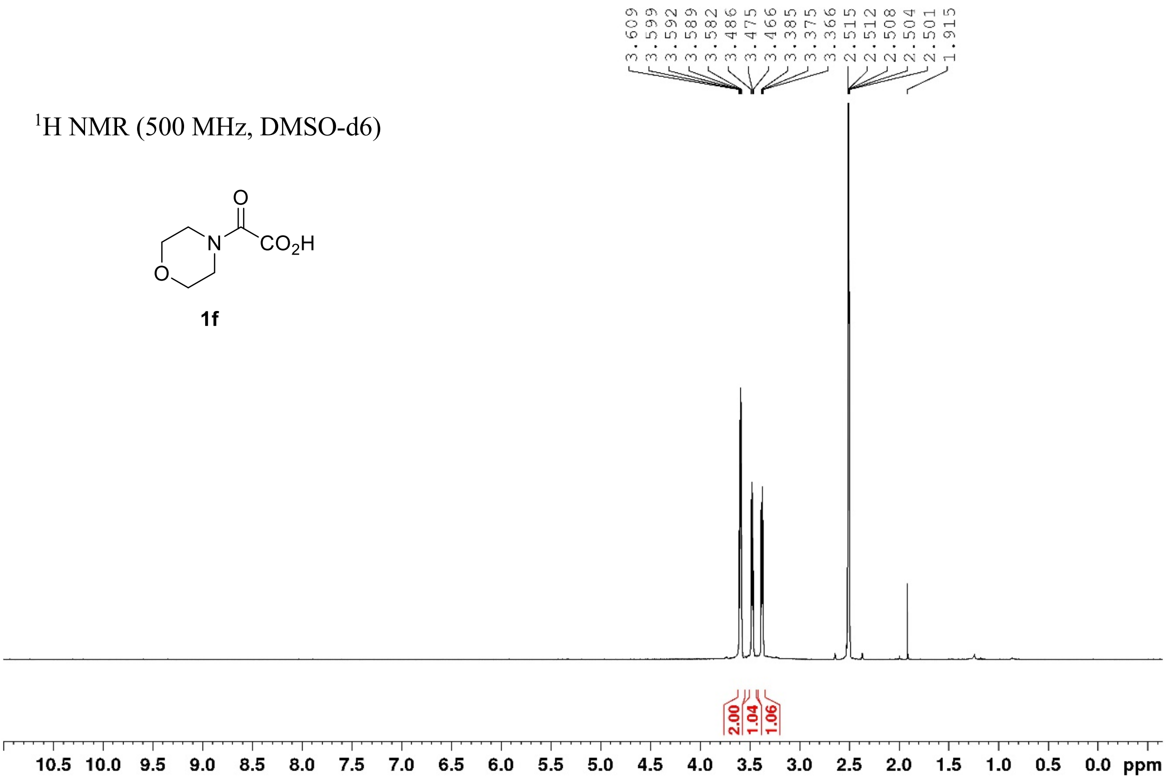


$^{13}$C NMR (126 MHz, DMSO-d6)

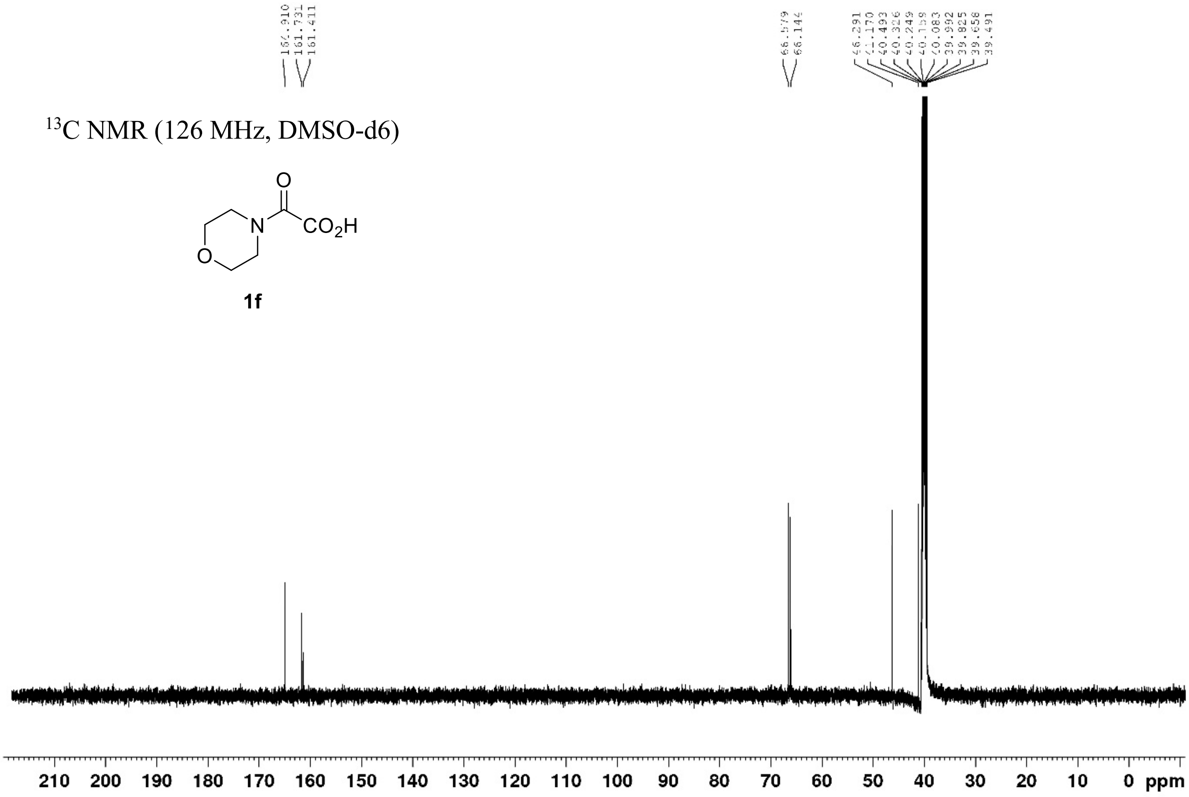

**Methyl 2-(((3s,5s,7s)-adamantan-1-yl)(methyl)amino)-2-oxoacetate S1g:**

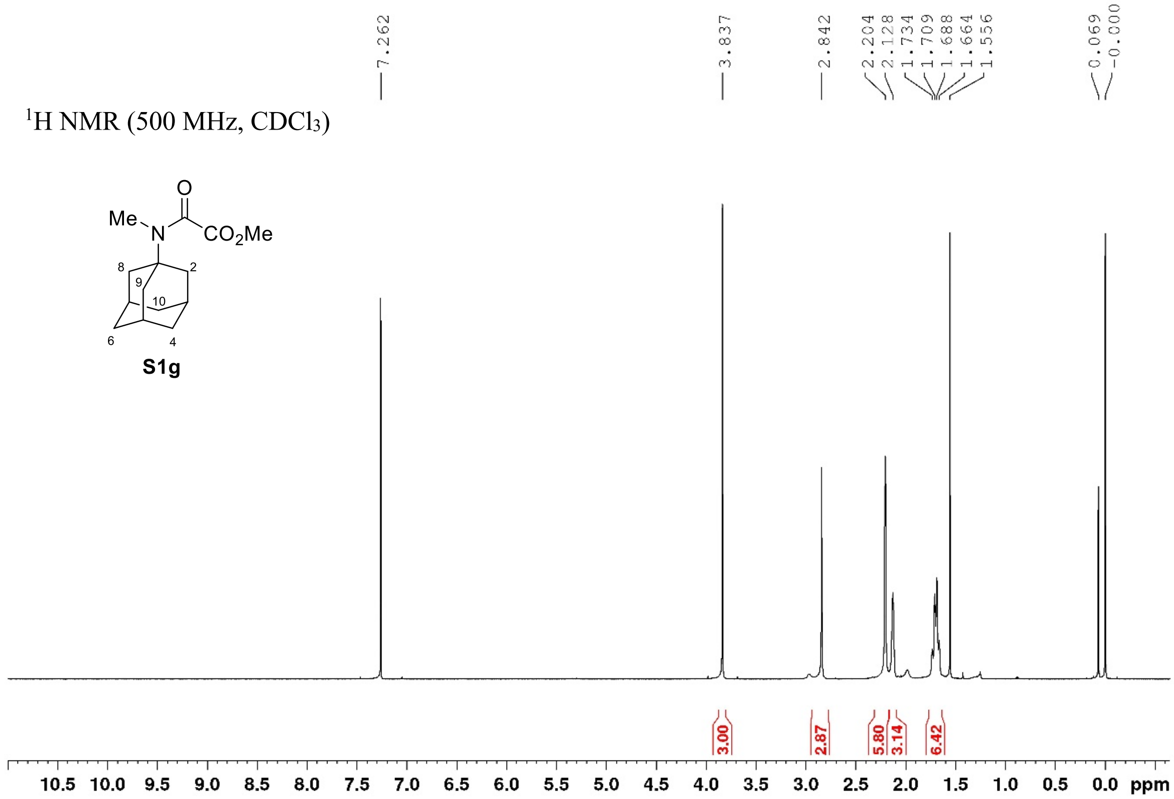


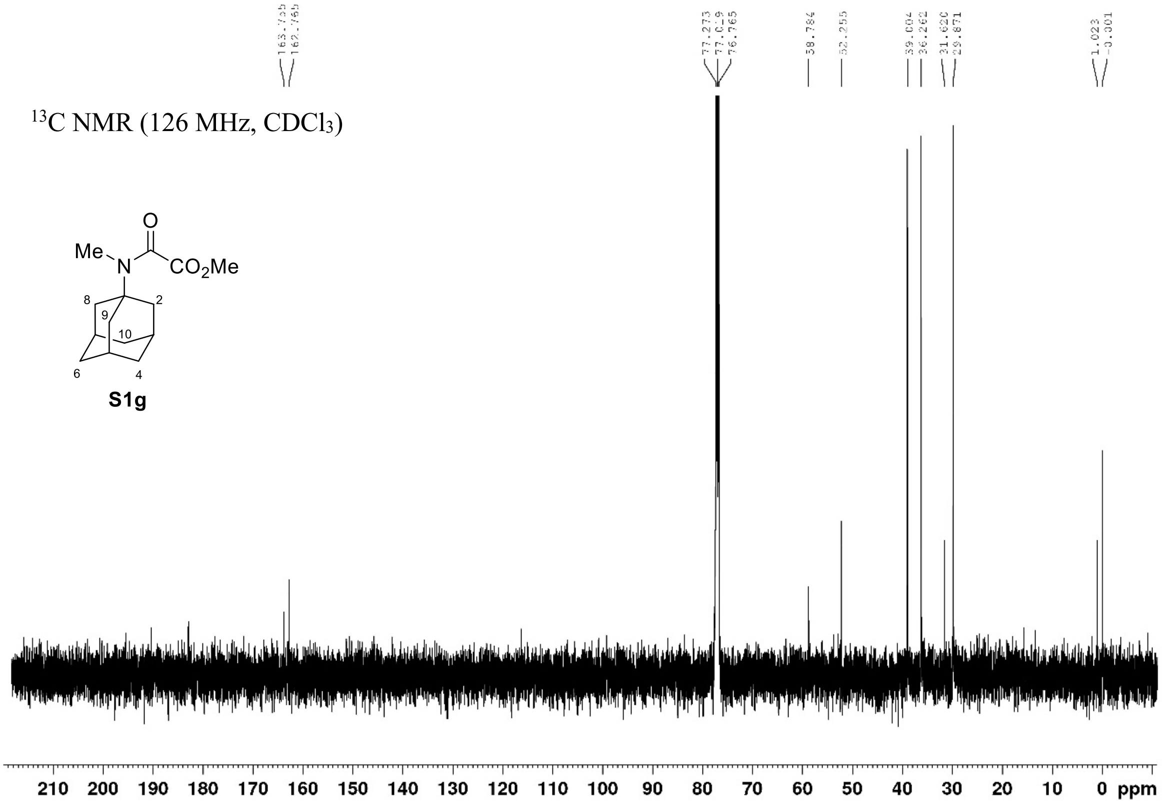

**2-(((3s,5s,7s)-Adamantan-1-yl)(methyl)amino)-2-oxoacetic acid 1g:**

$^{1}$H NMR (500 MHz, DMSO-d6)

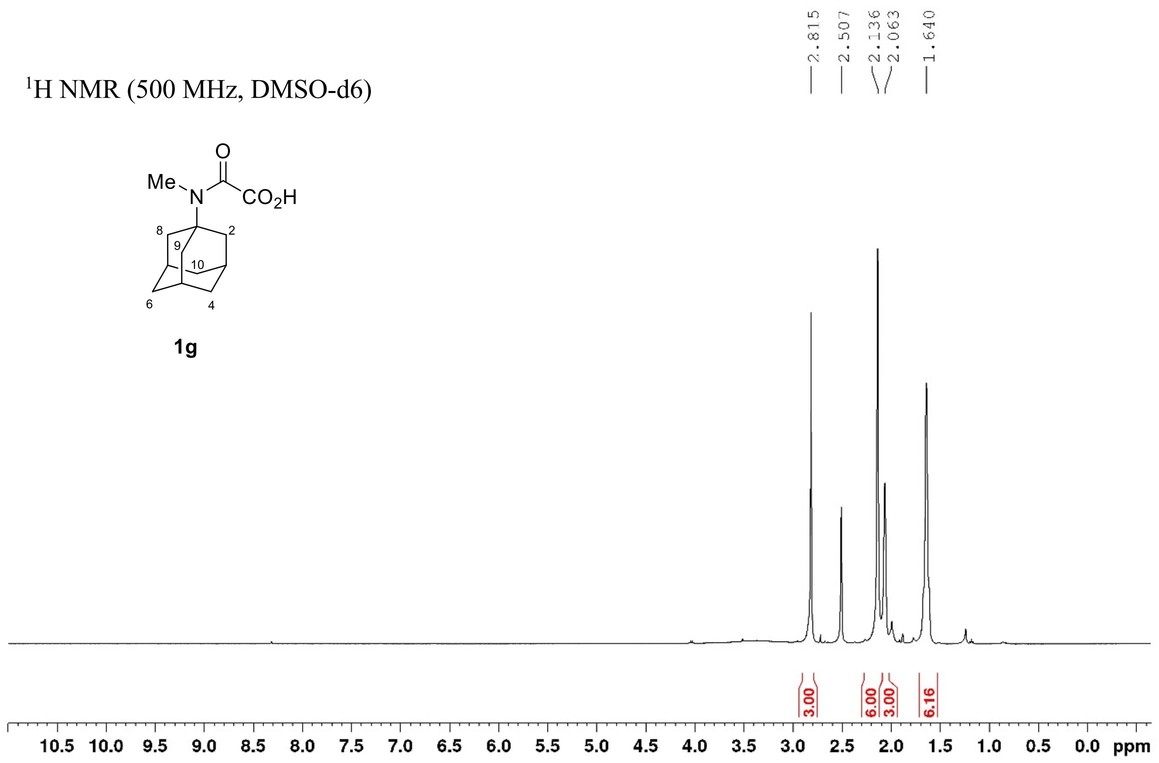


$^{13}$C NMR (126 MHz, DMSO-d6)

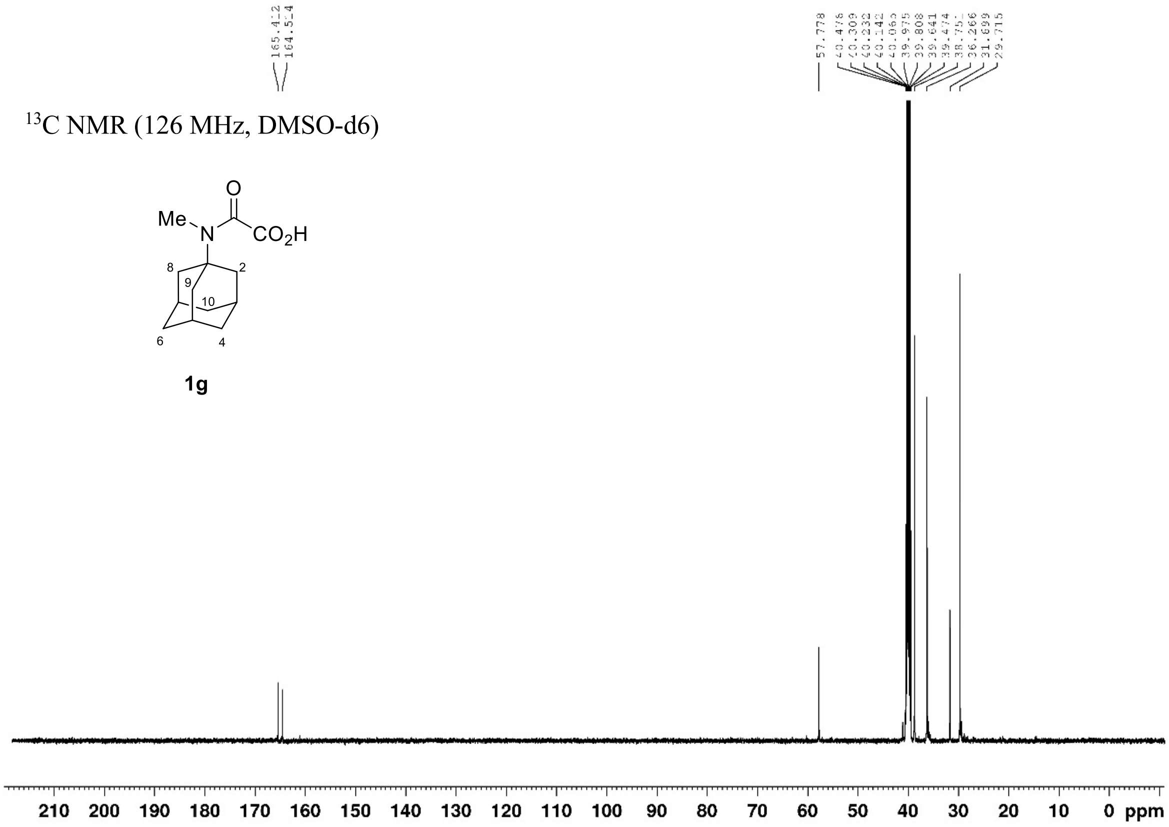

**Methyl 2-(benzyl(phenyl)amino)-2-oxoacetate S1h:**

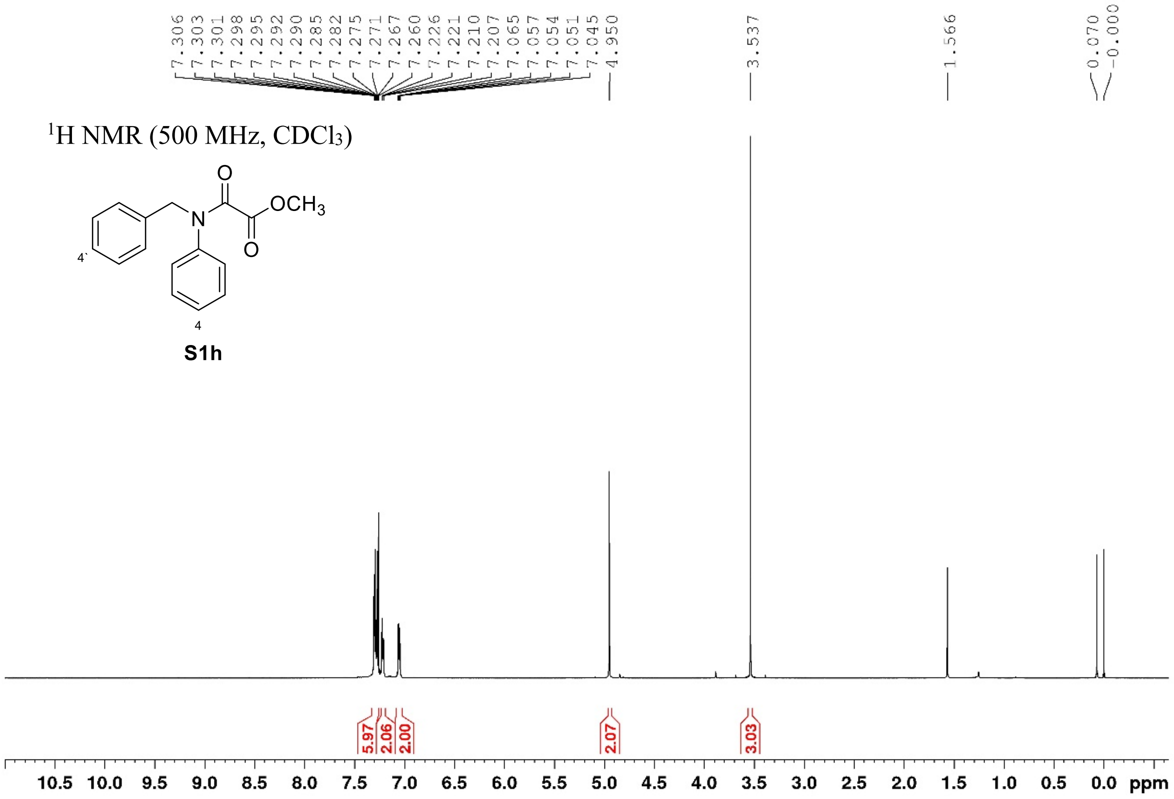


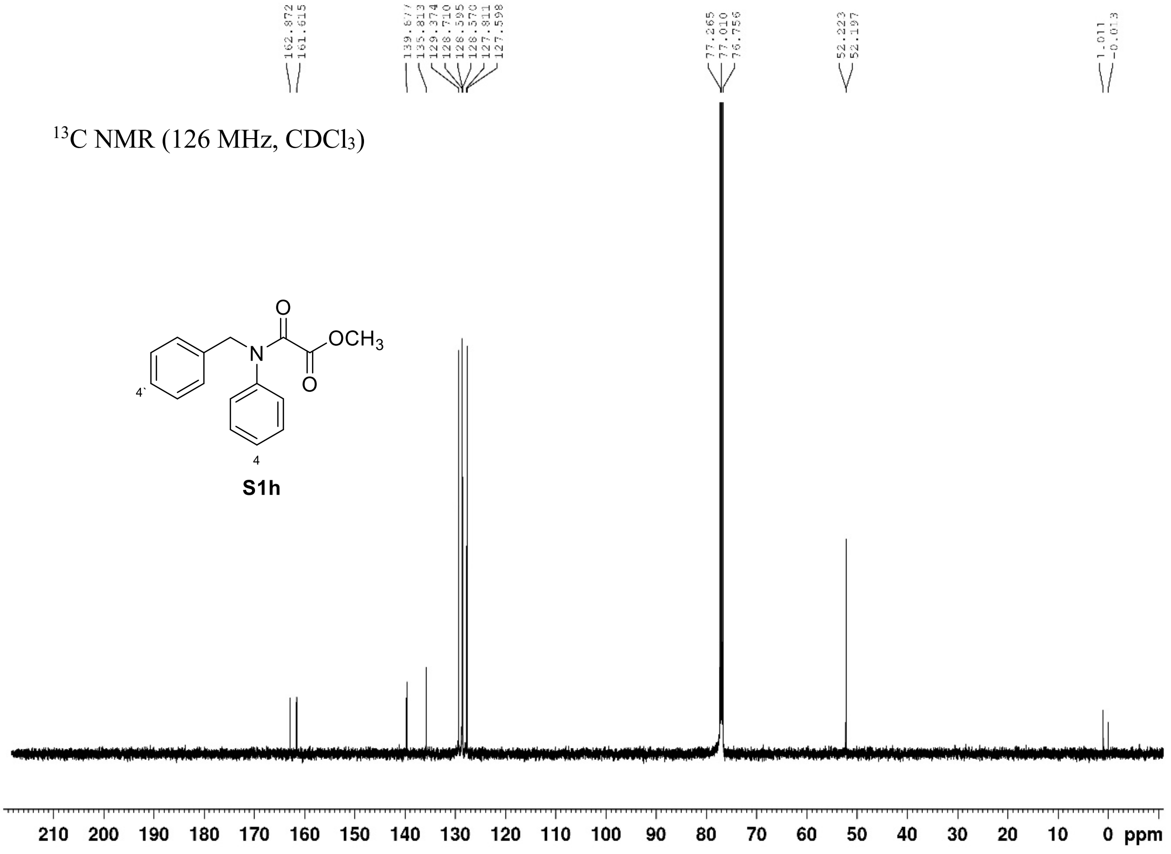

**2-(Benzyl(phenyl)amino)-2-oxoacetic acid 1h:**

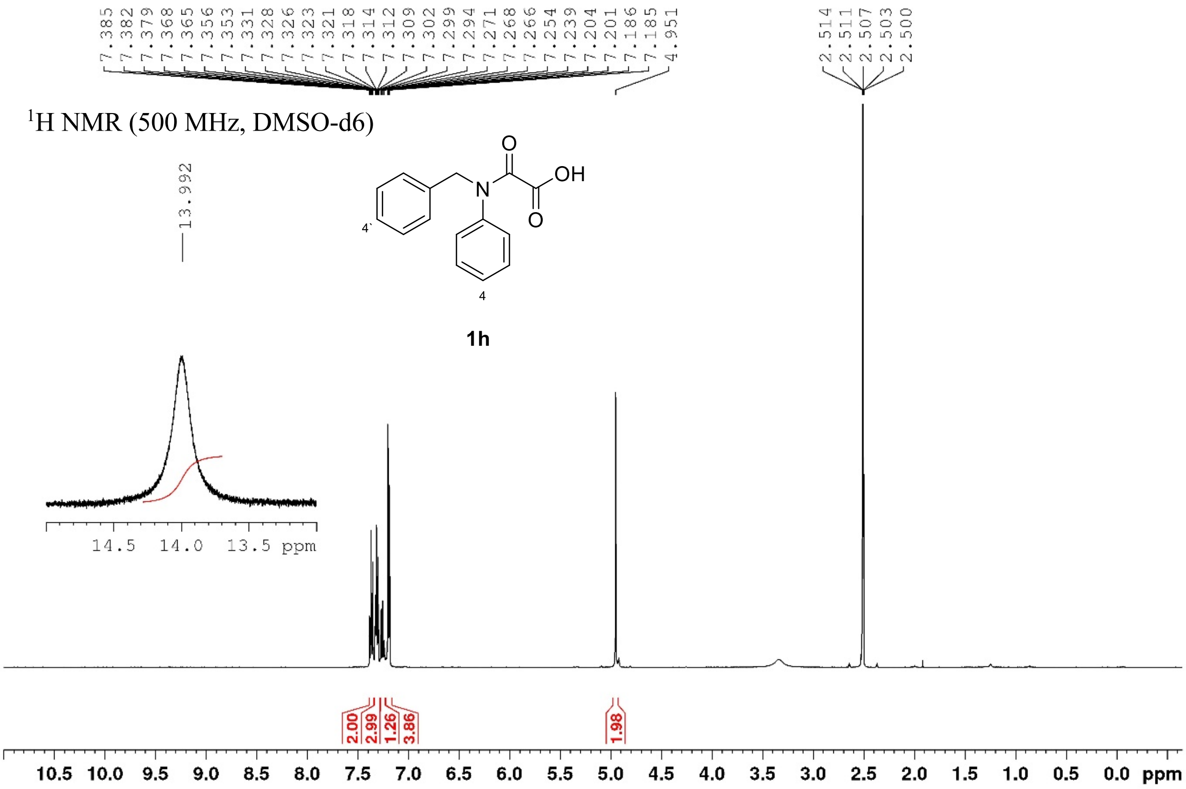


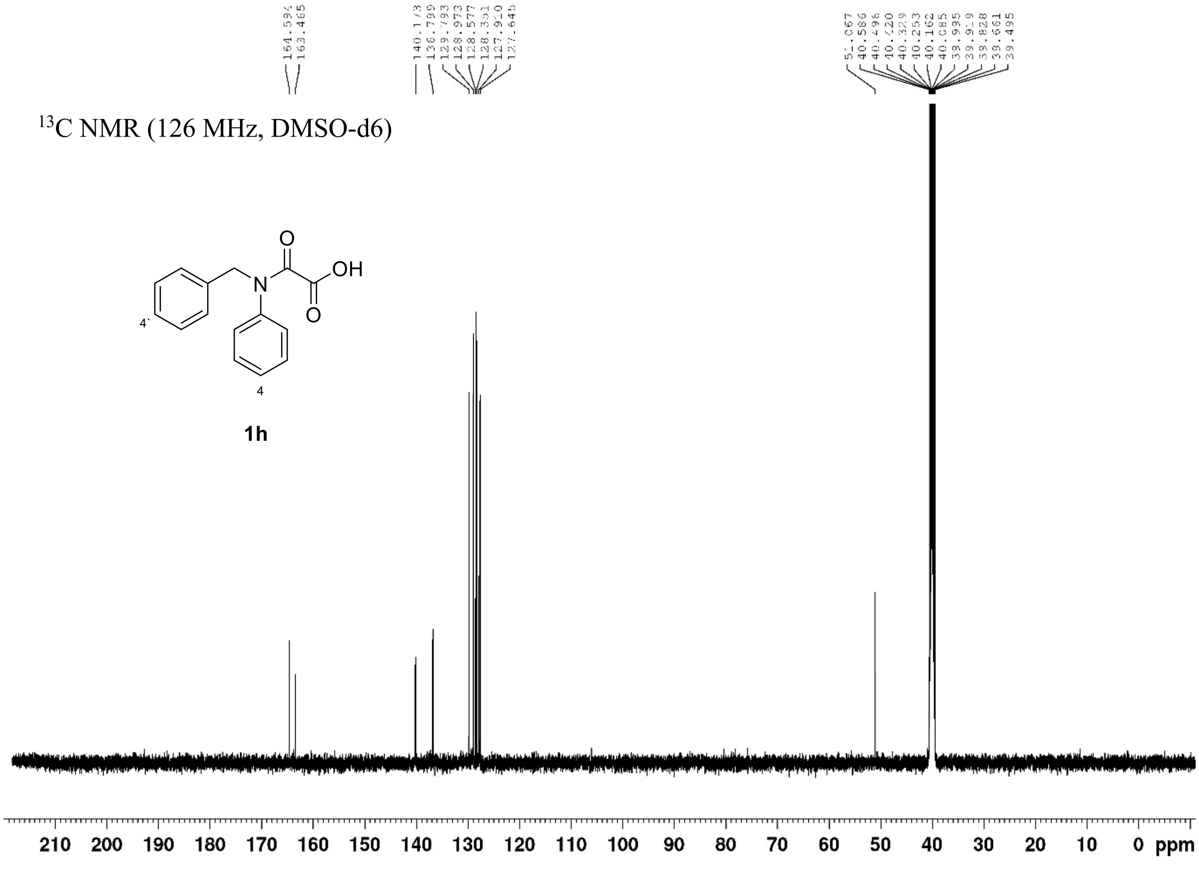

**Methyl 2-(3,4-dihydroquinolin-1(2*H*)-yl)-2-oxoacetate S1i:**

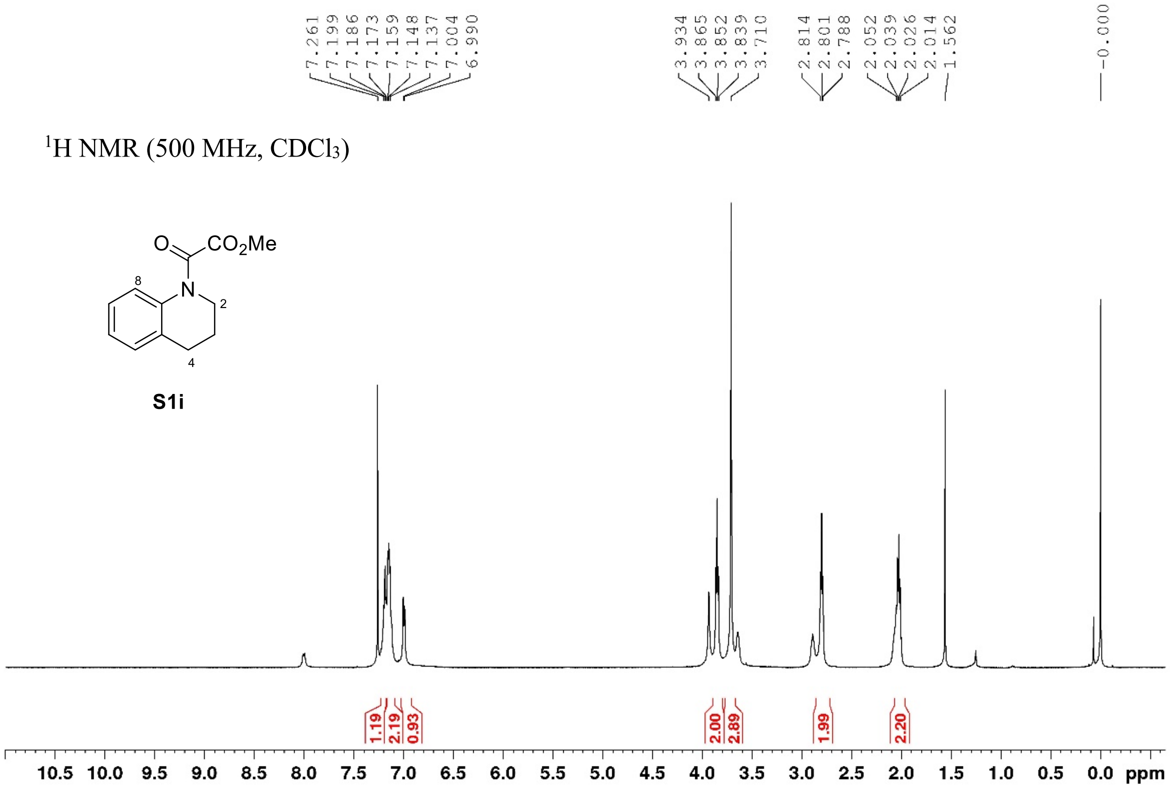


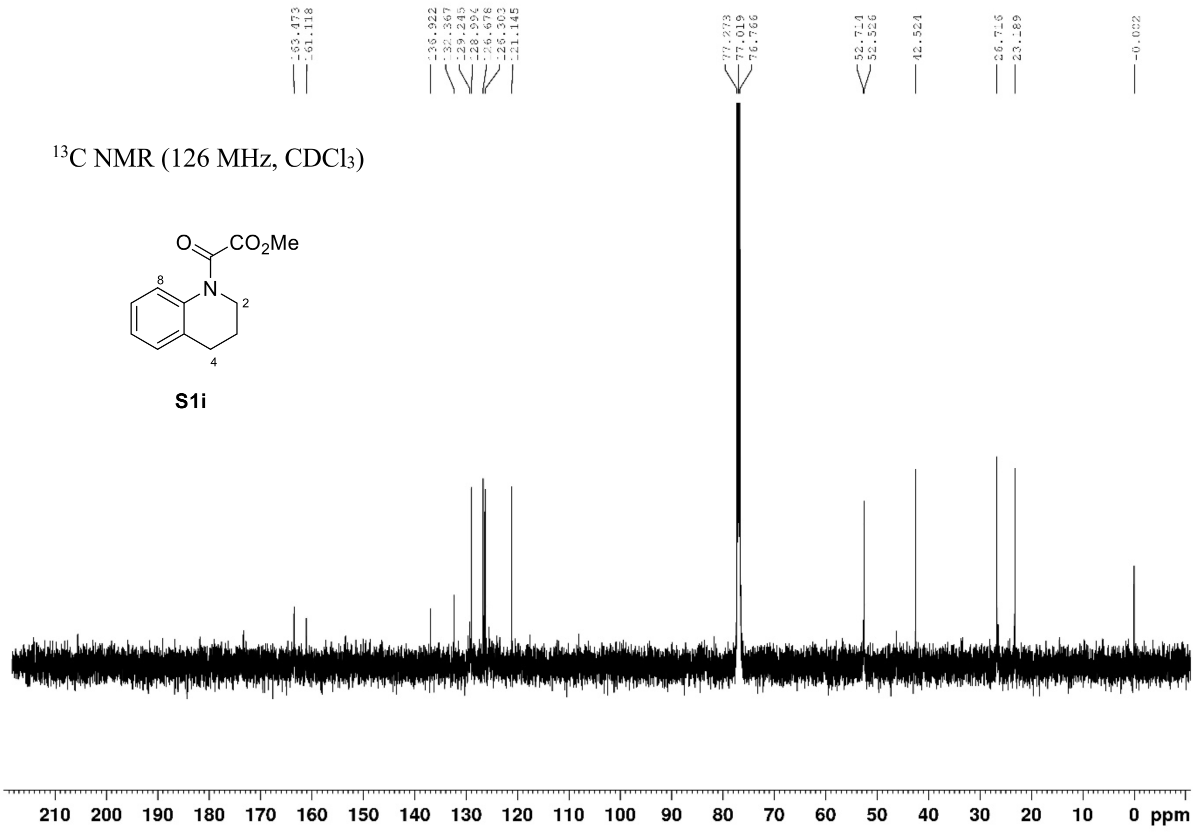

**2-(3,4-Dihydroquinolin-1(2*H*)-yl)-2-oxoacetic acid 1i:**

$^1$H NMR (500 MHz, DMSO-d6)

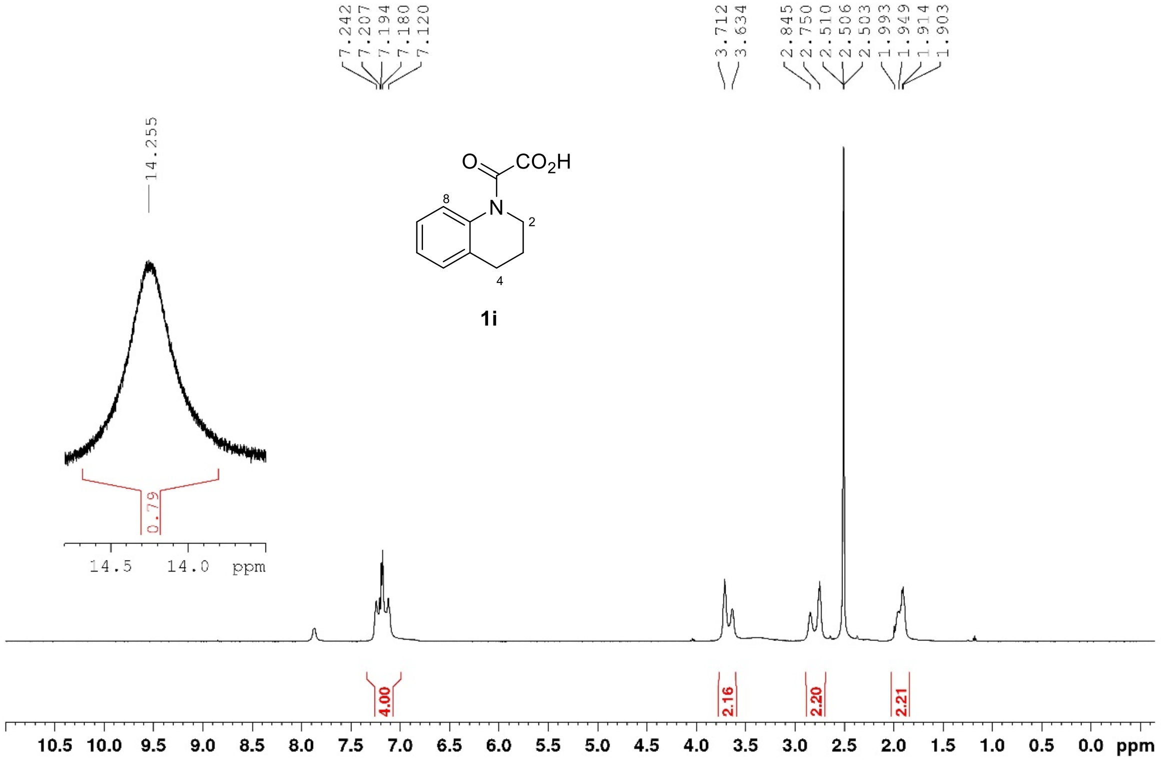

$^{13}$C NMR (126 MHz, DMSO-d6)

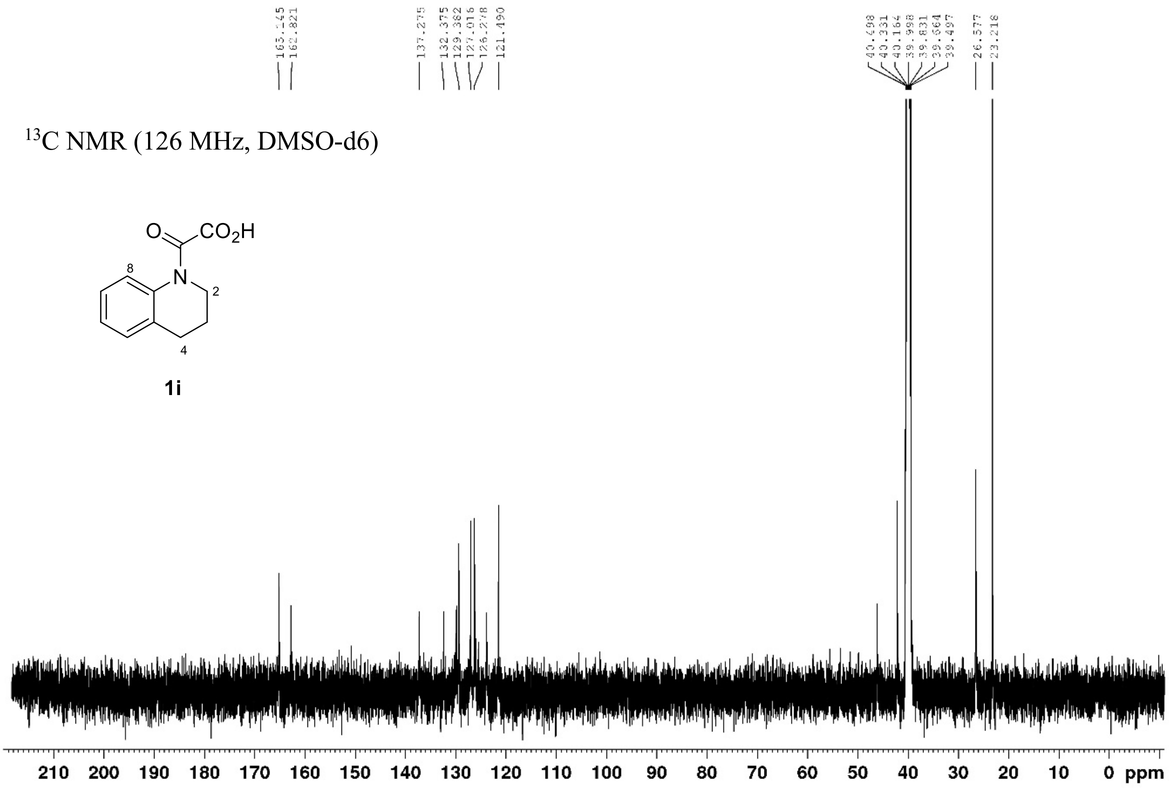

**Methyl 2-((4-methoxyphenyl)(methyl)amino)-2-oxoacetate S1j:**

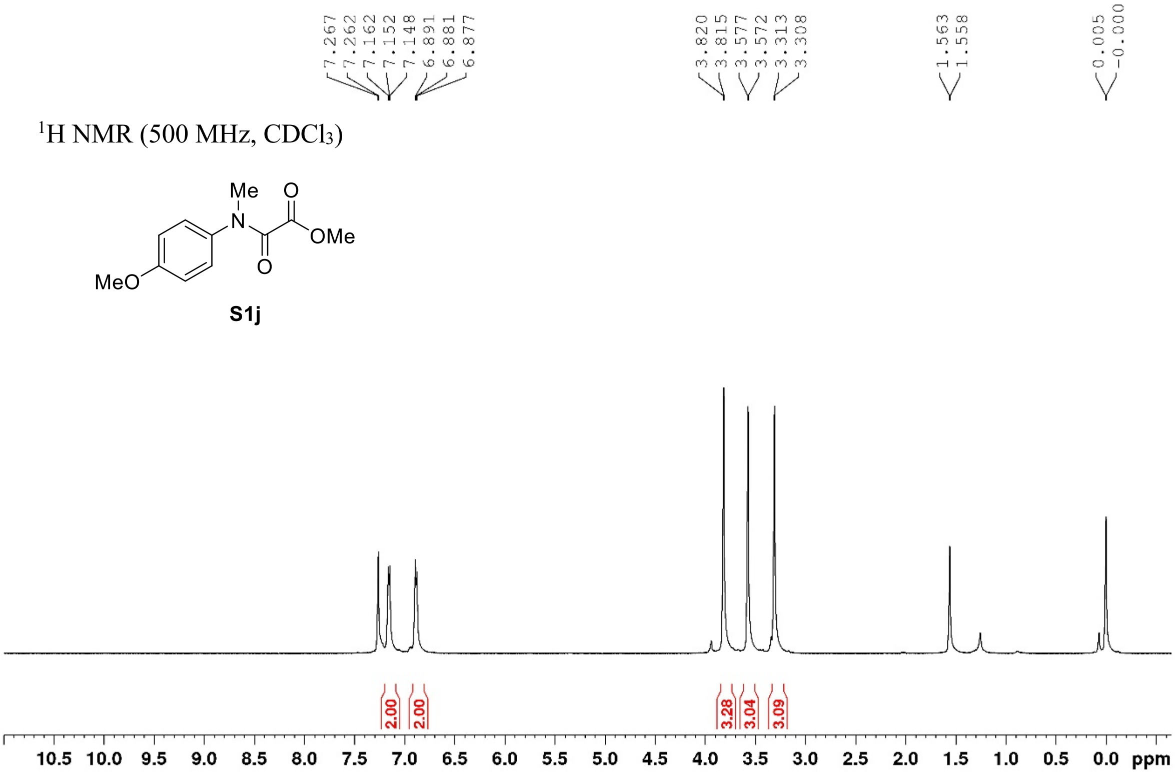


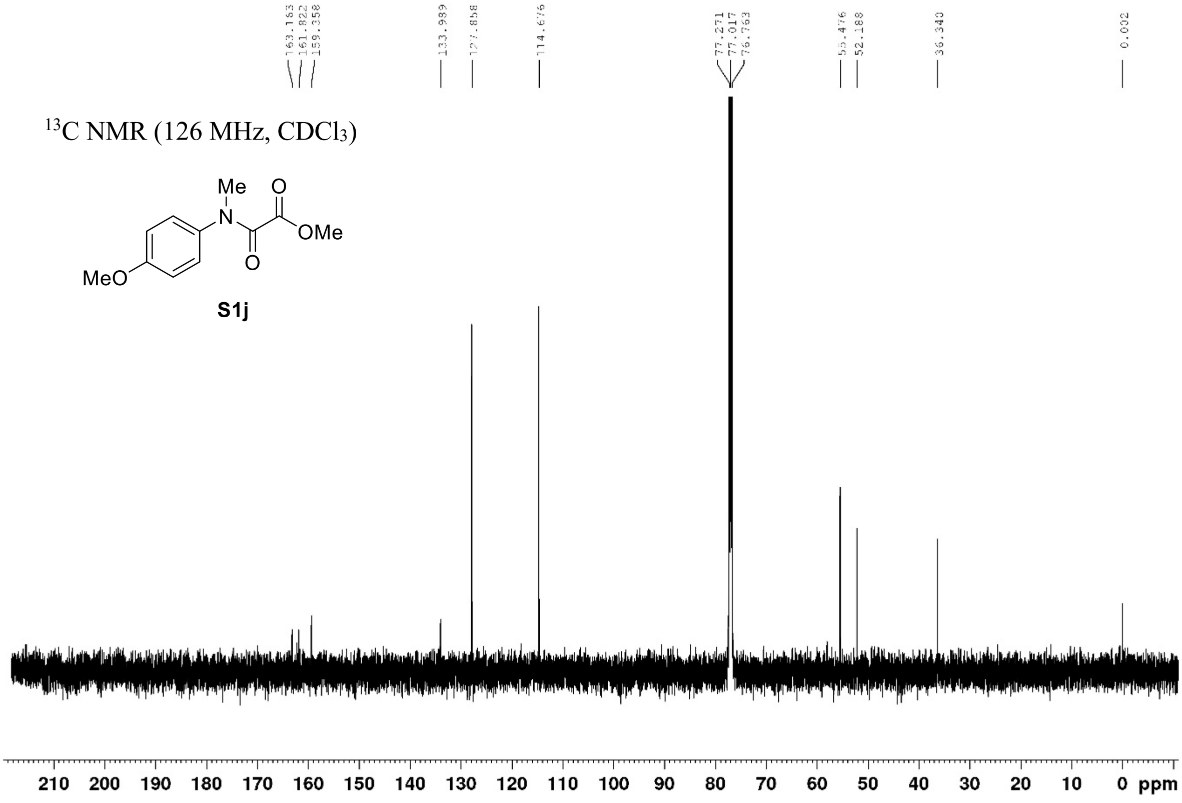

**2-((4-Methoxyphenyl)(methyl)amino)-2-oxoacetic acid 1j:**

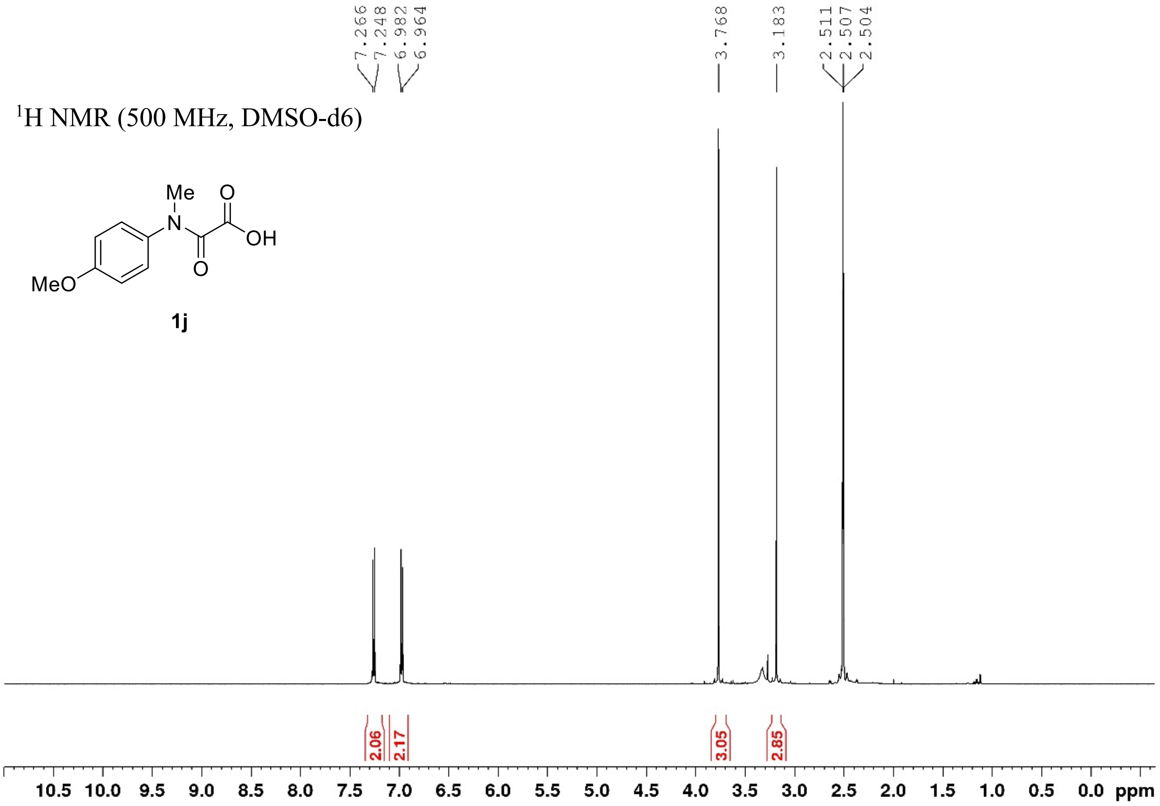


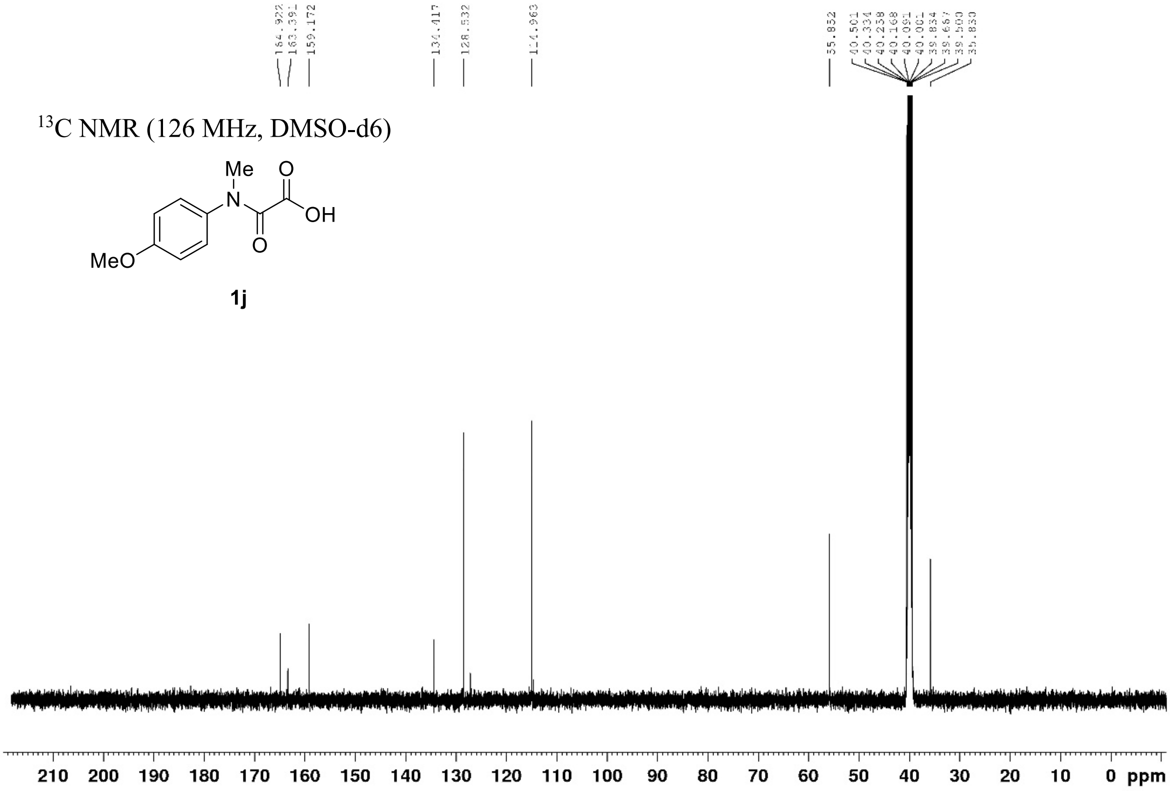

**Methyl 2-(isopropyl(phenyl)amino)-2-oxoacetate S1k:**

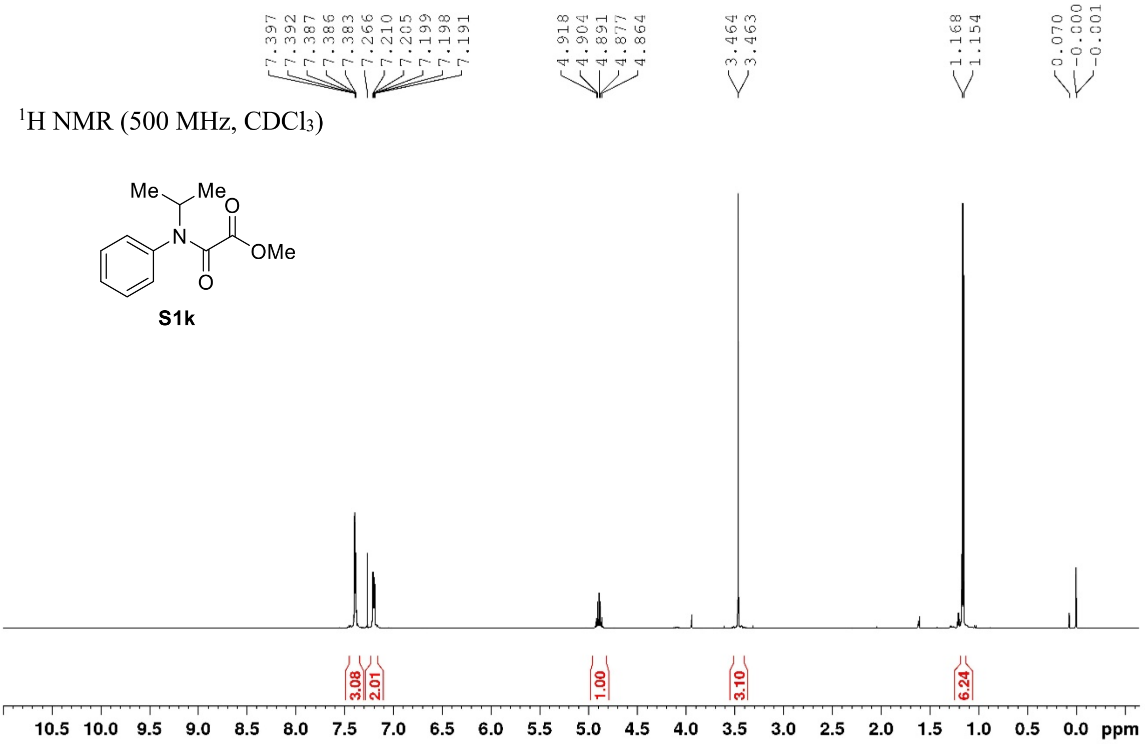


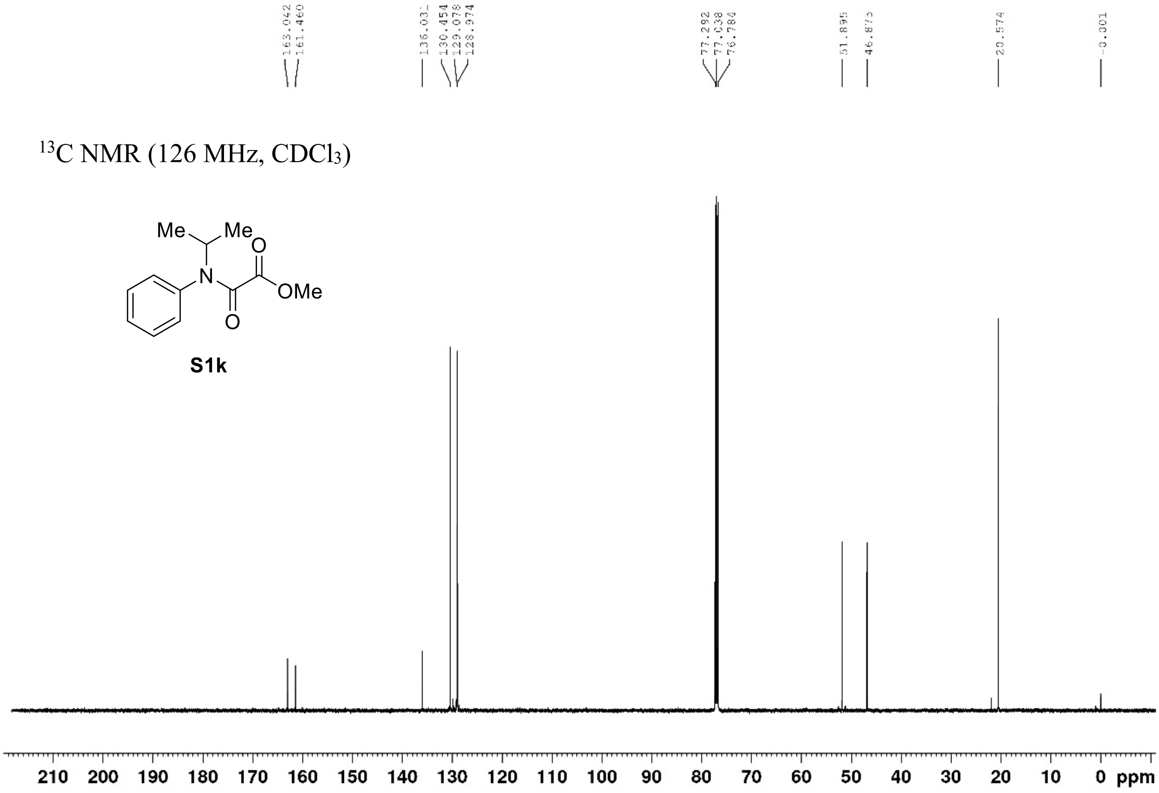

**2-(Isopropyl(phenyl)amino)-2-oxoacetic acid 1k:**

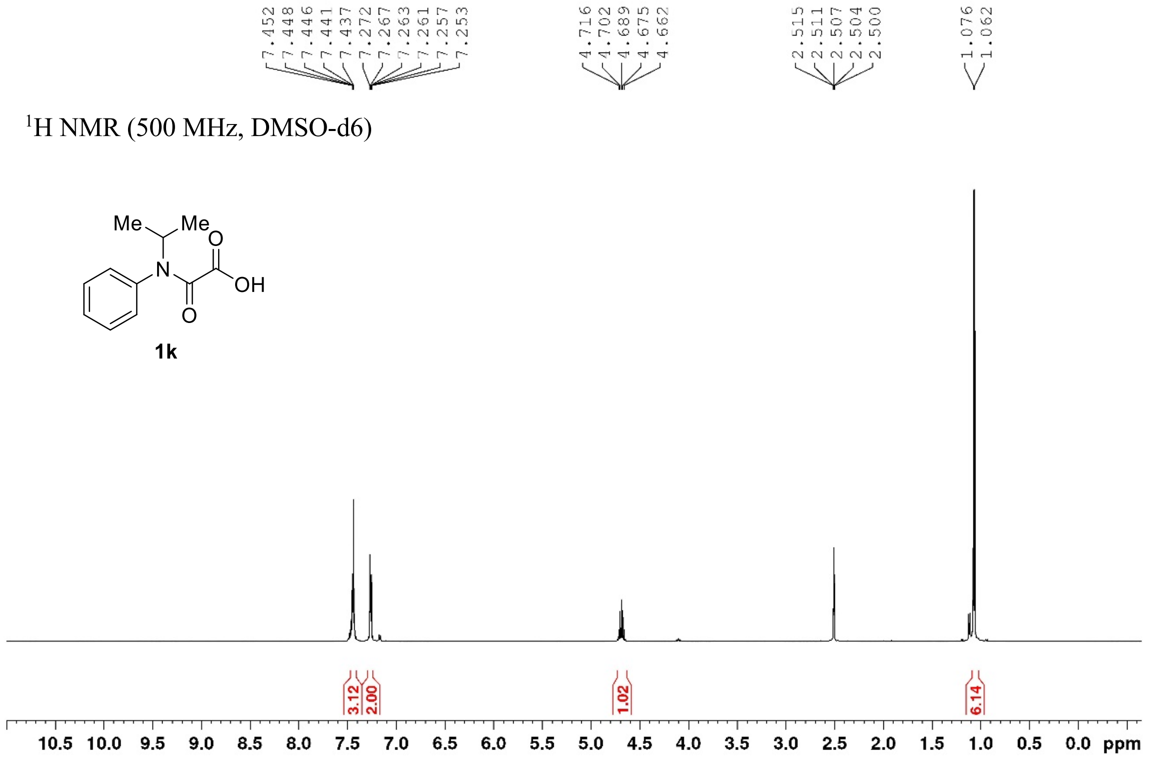


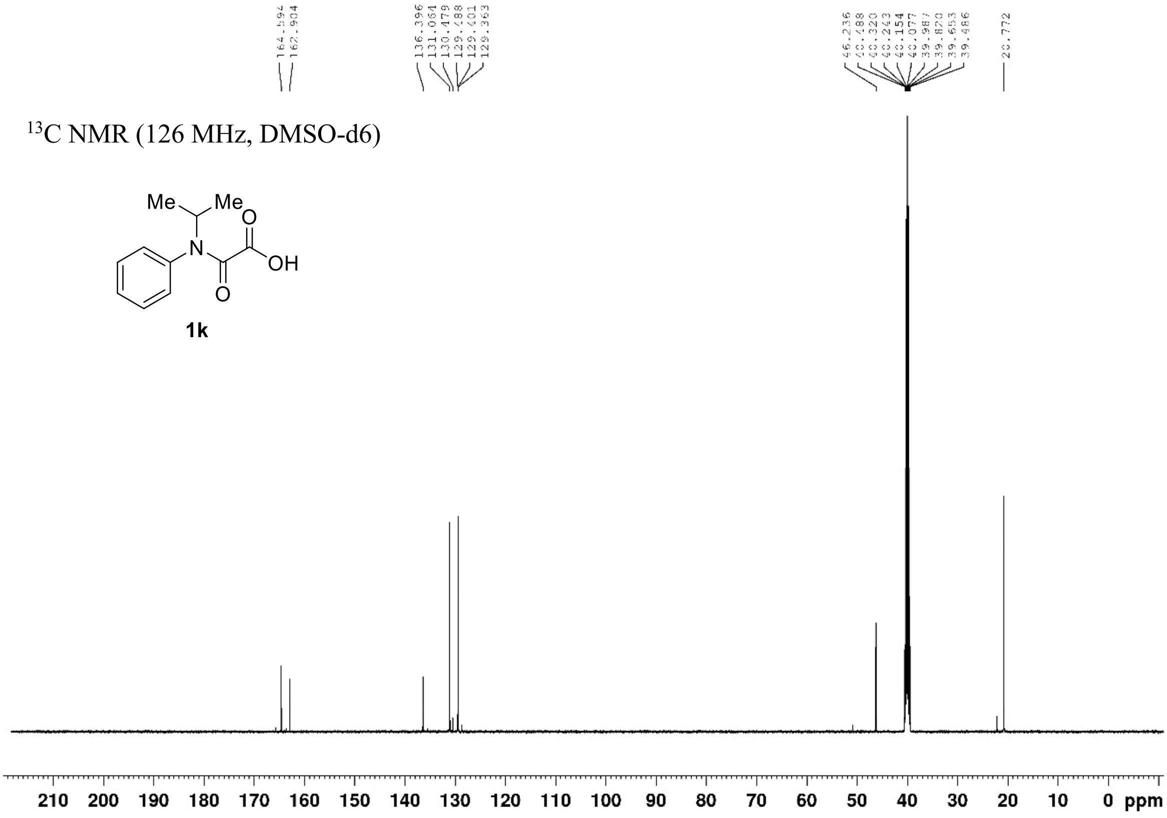

**Methyl 2-(methyl(*o*-tolyl)amino)-2-oxoacetate S1l**:

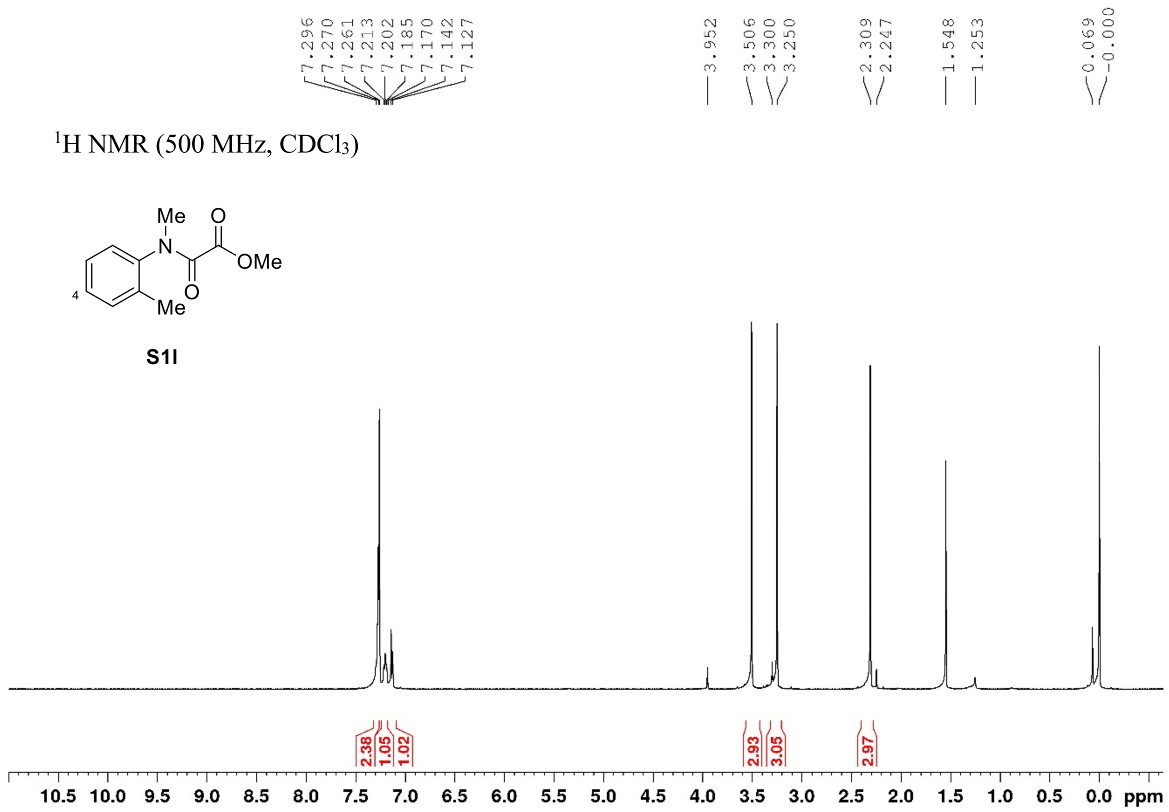


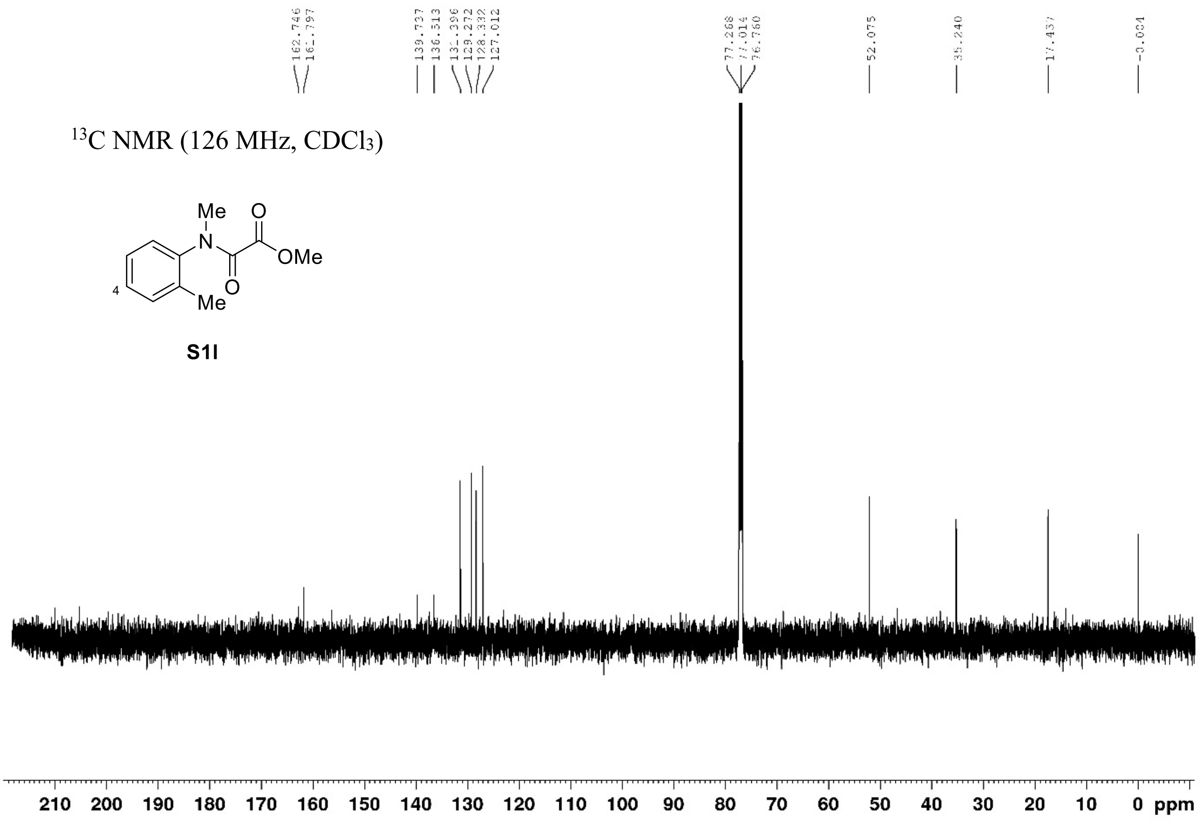

**2-(Methyl(*o*-tolyl)amino)-2-oxoacetic acid 1l:**

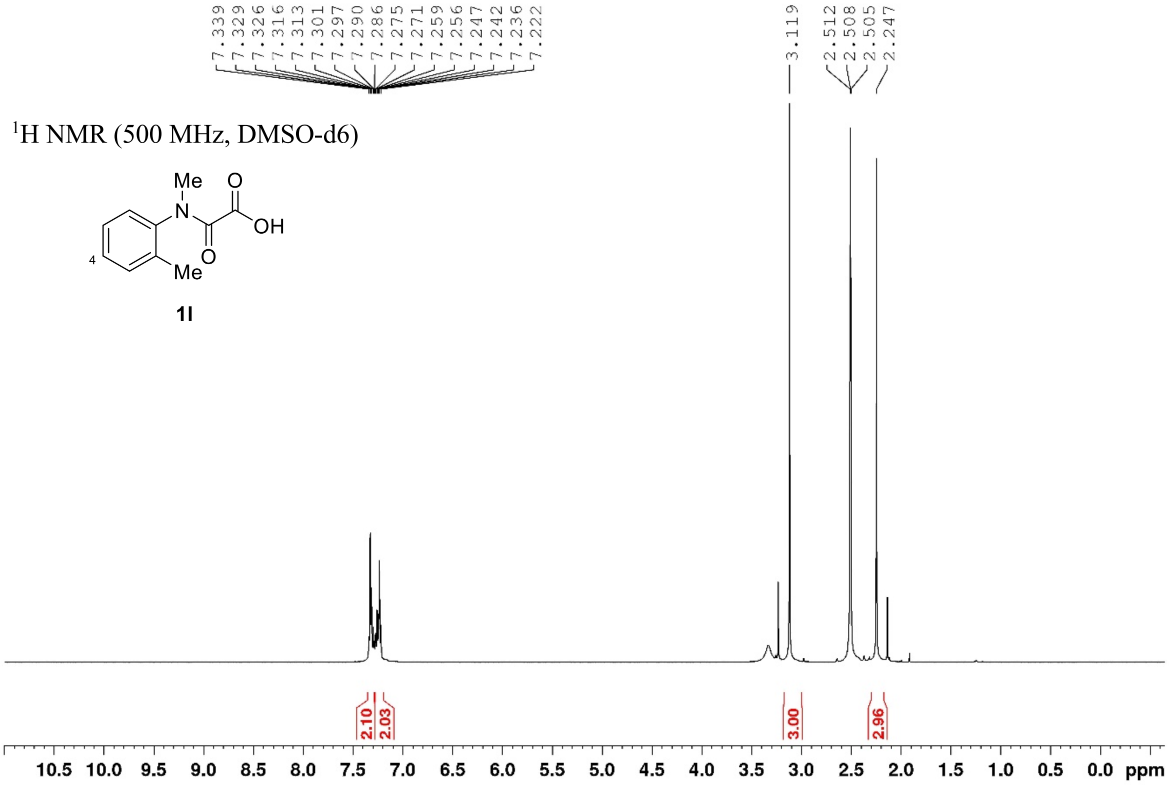


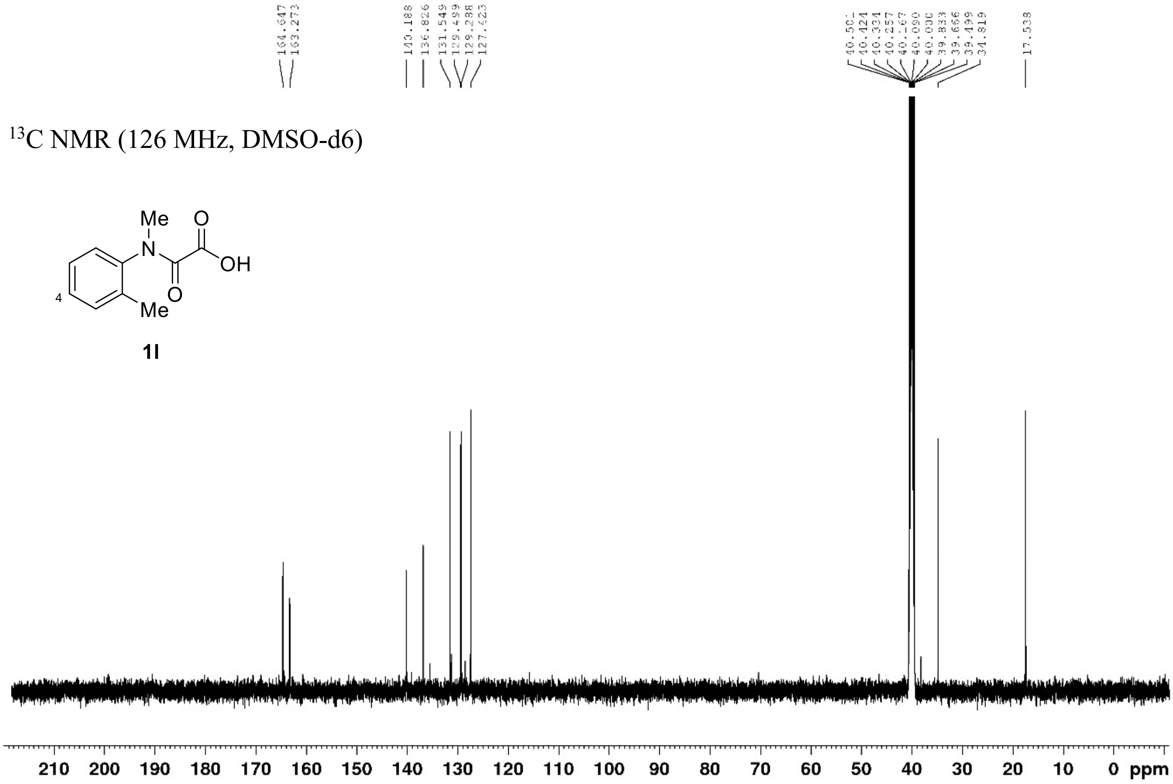

**Methyl 2-oxo-2-(phenylamino)acetate S1m:**

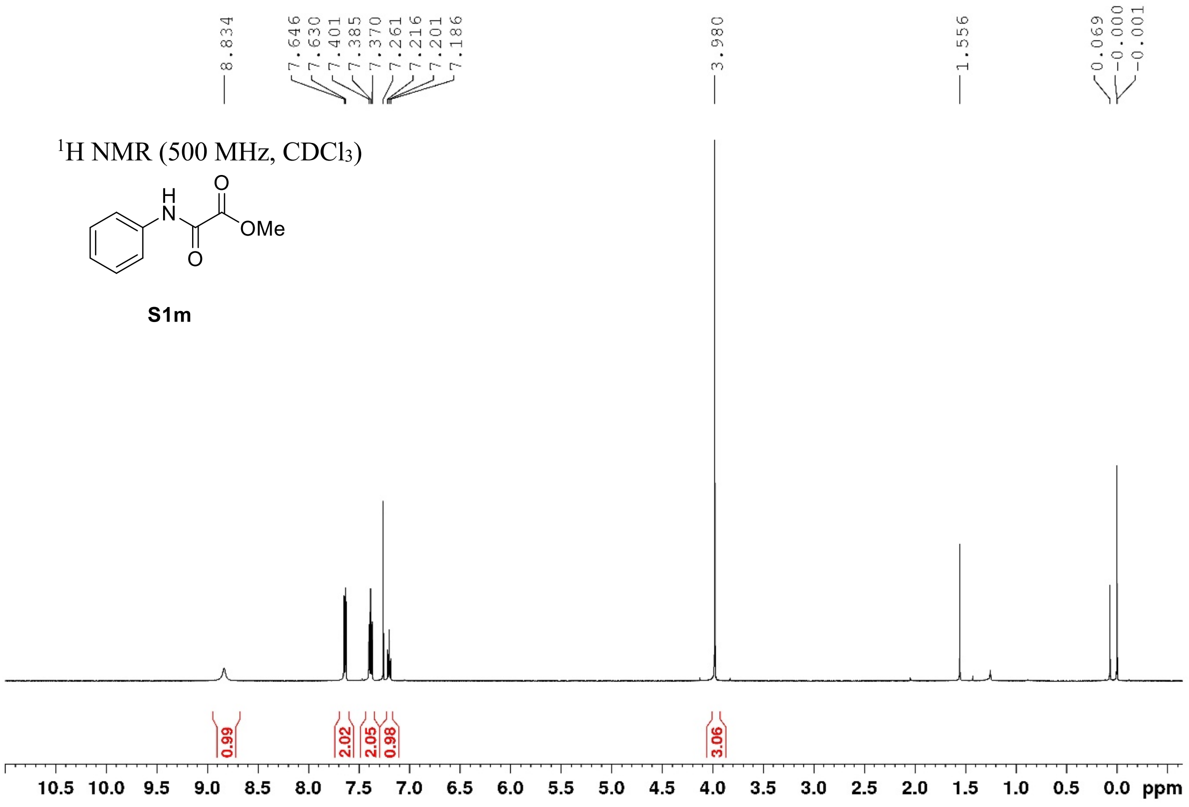


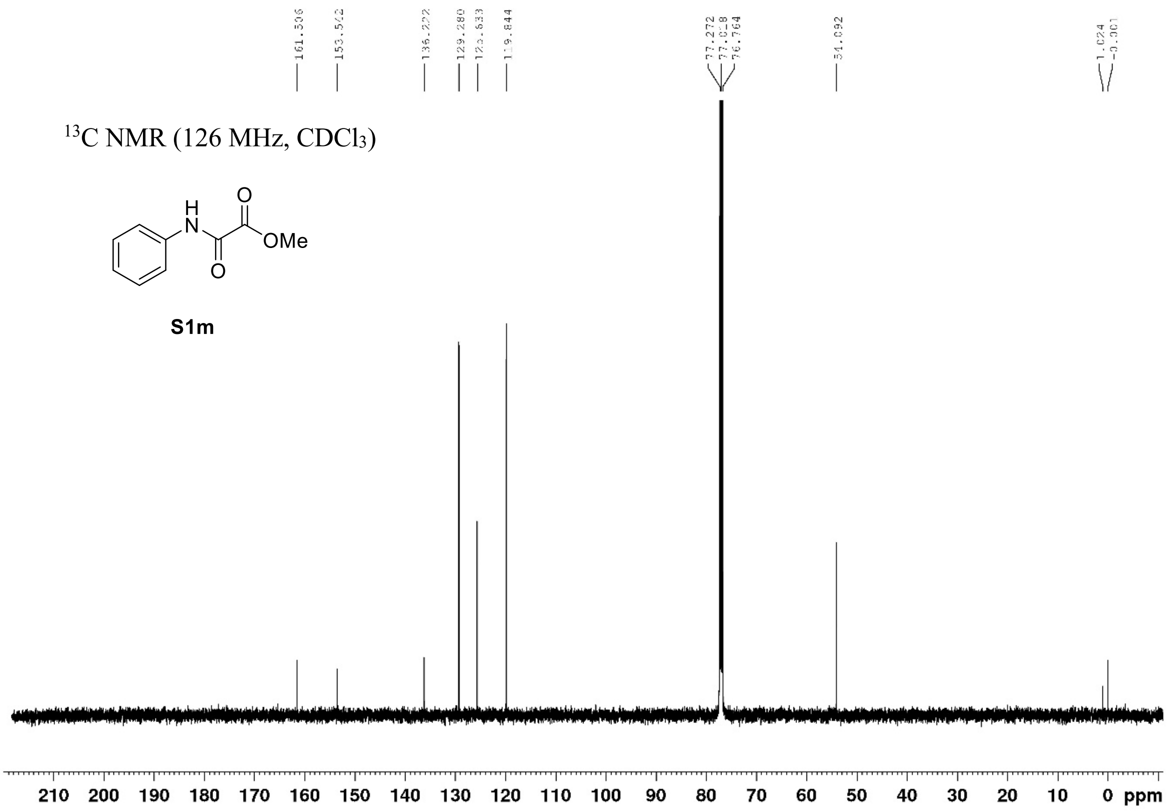

**2-Oxo-2-(phenylamino)acetic acid 1m:**

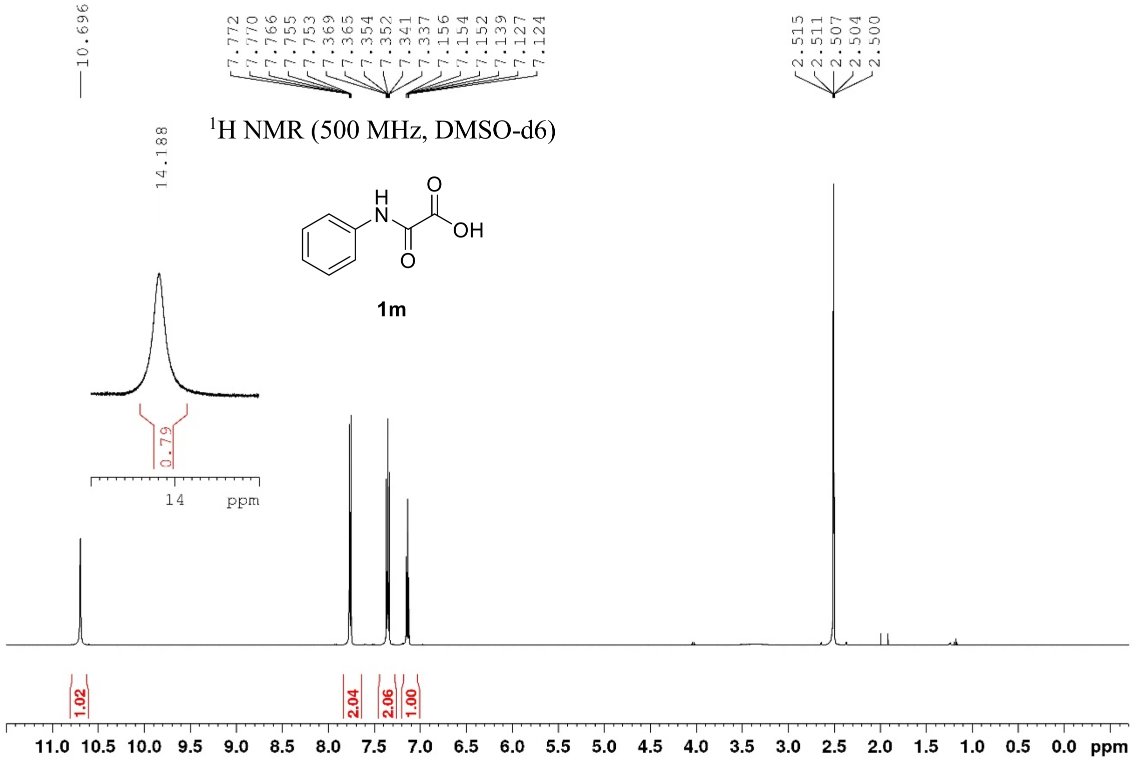


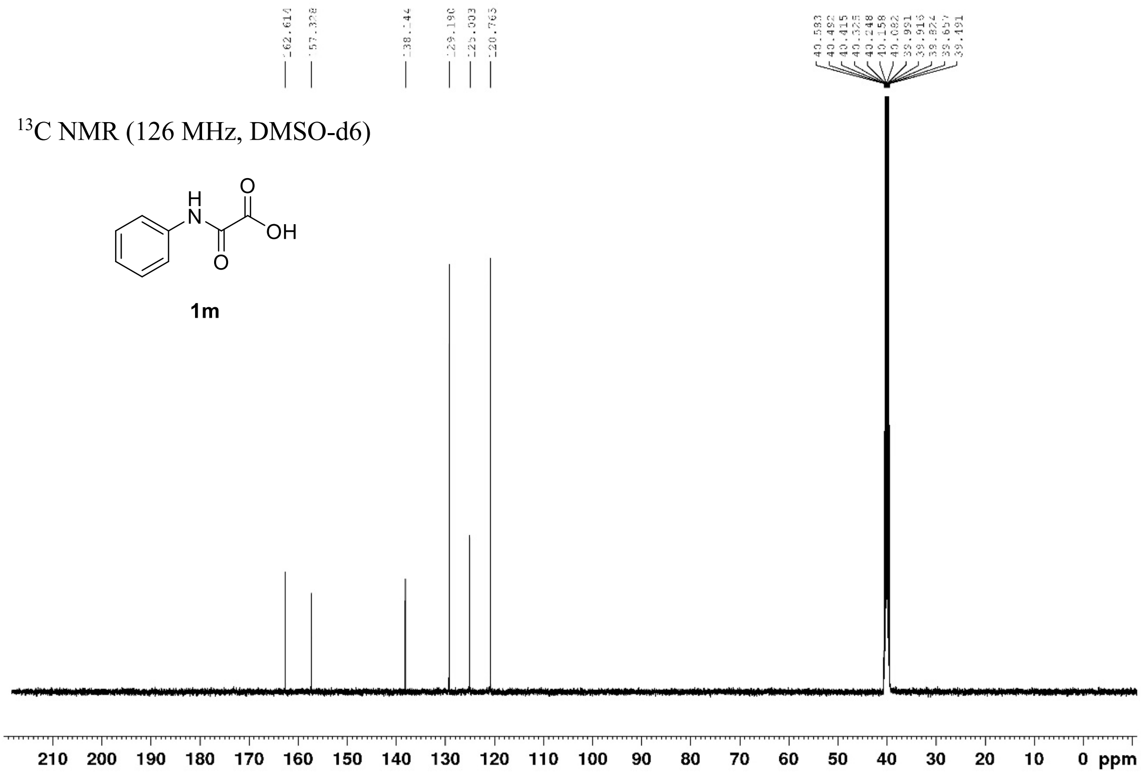

**((2-Bromophenethyl)(methyl)amino)-2-oxoacetic acid 1n:**

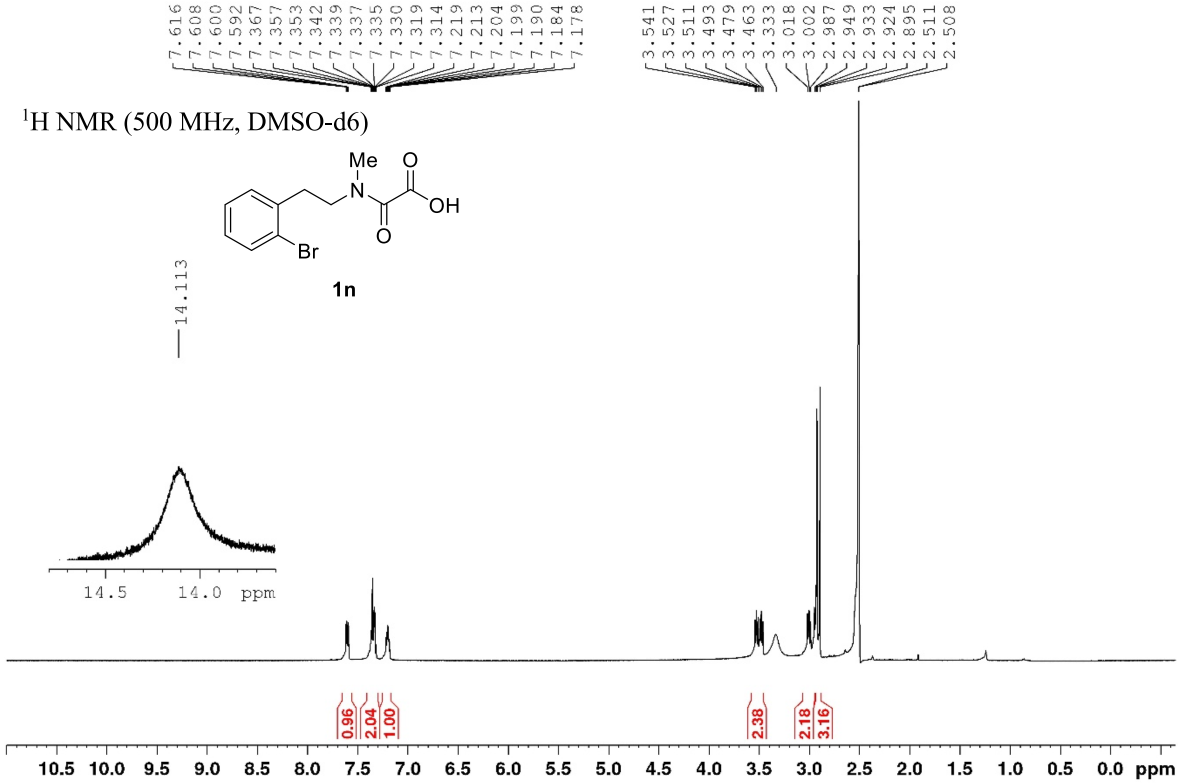


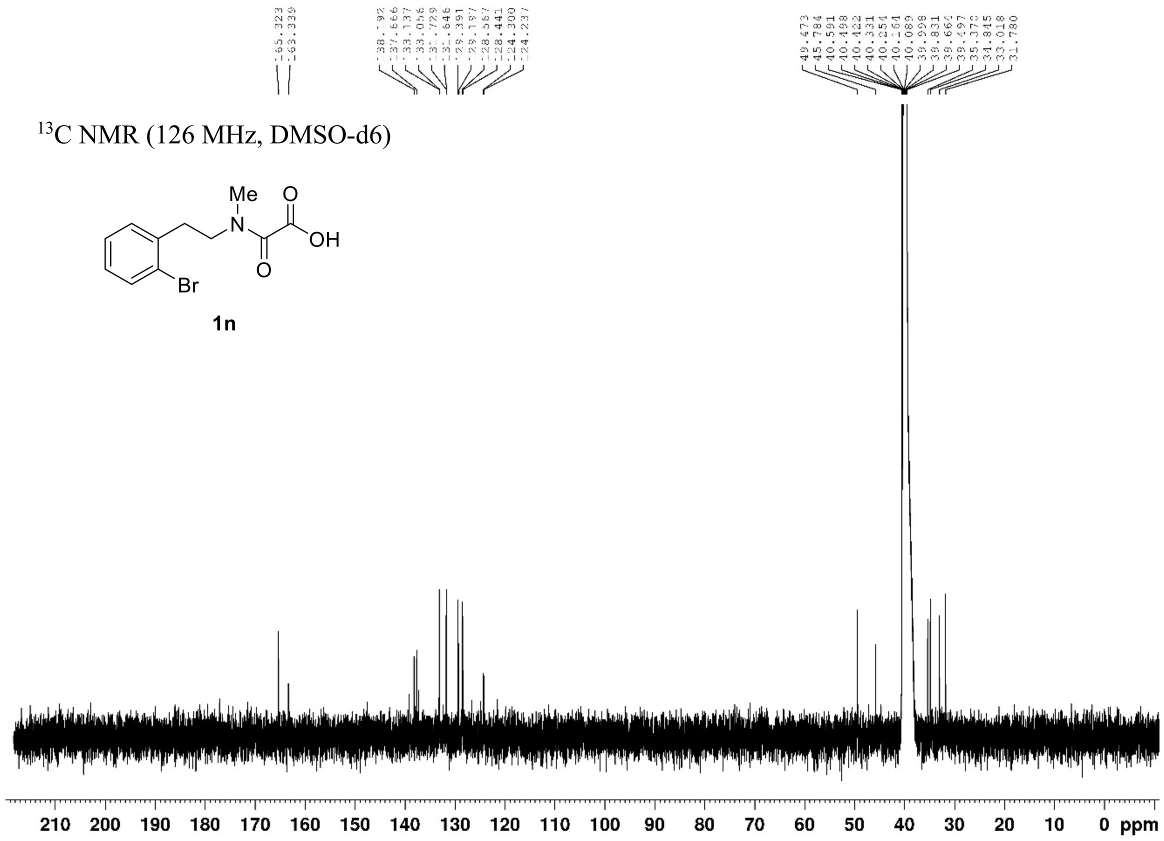

**Methyl 2-(benzylamino)-2-oxoacetate S1o:**

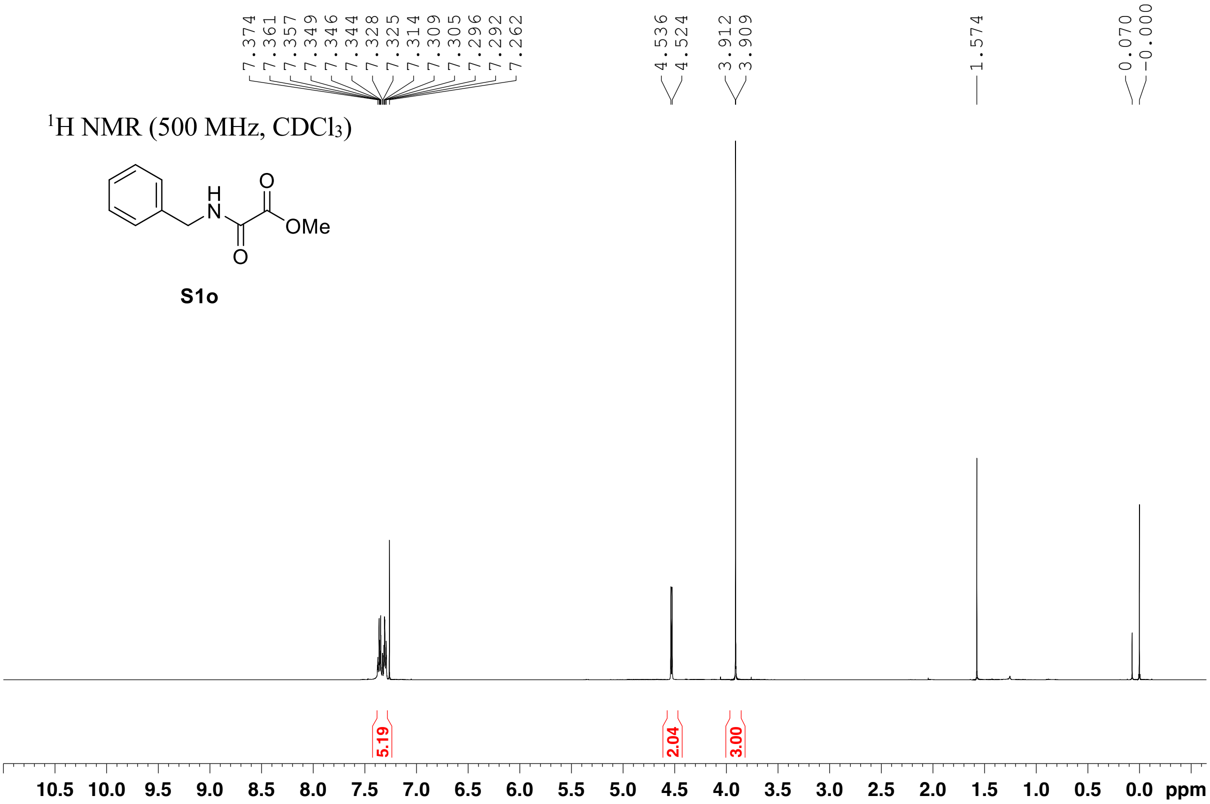


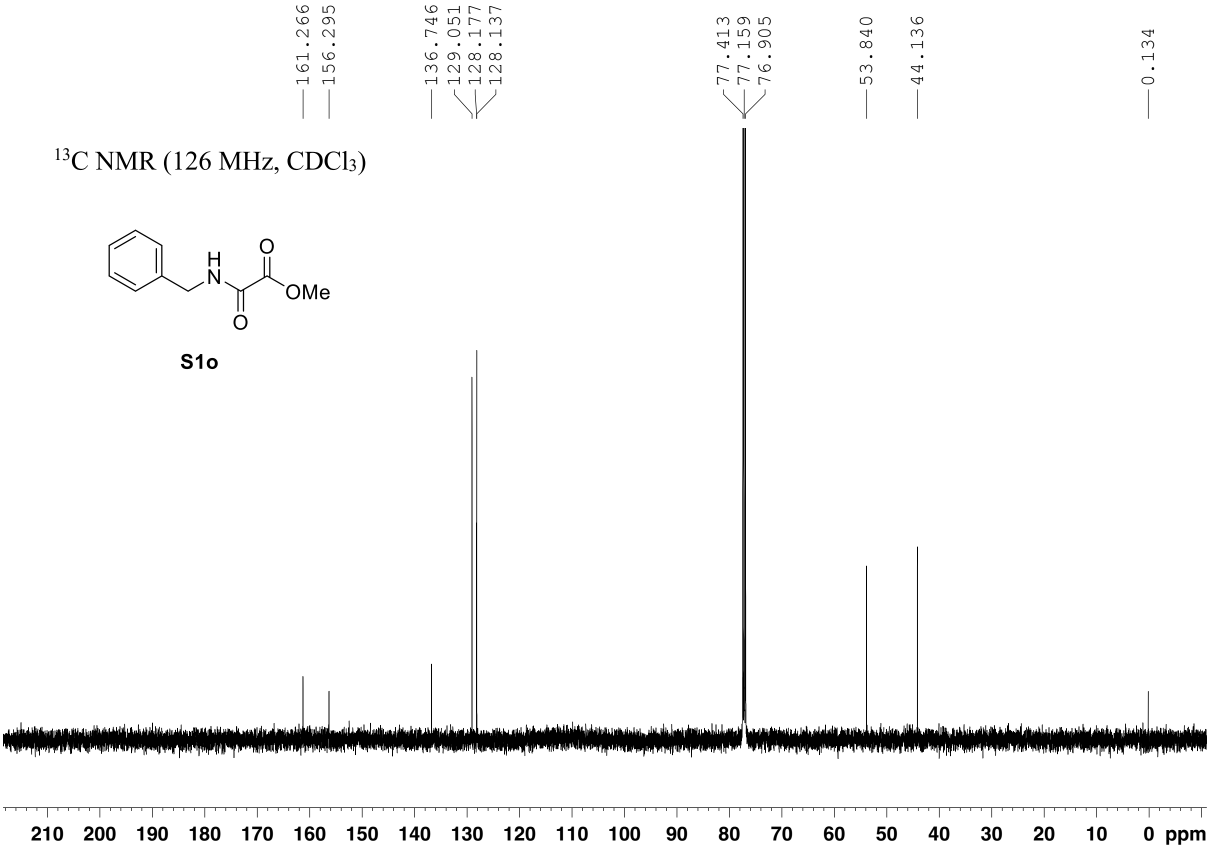

**2-(Benzylamino)-2-oxoacetic acid 1o:**

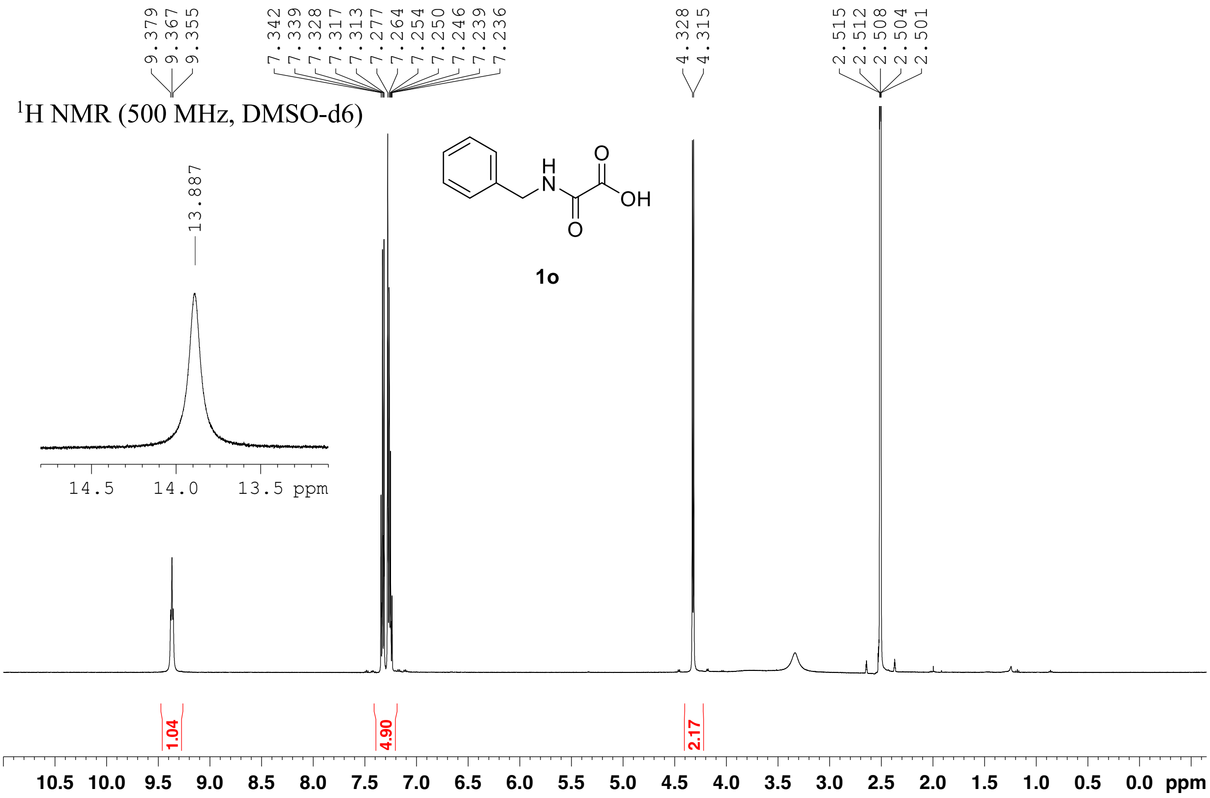


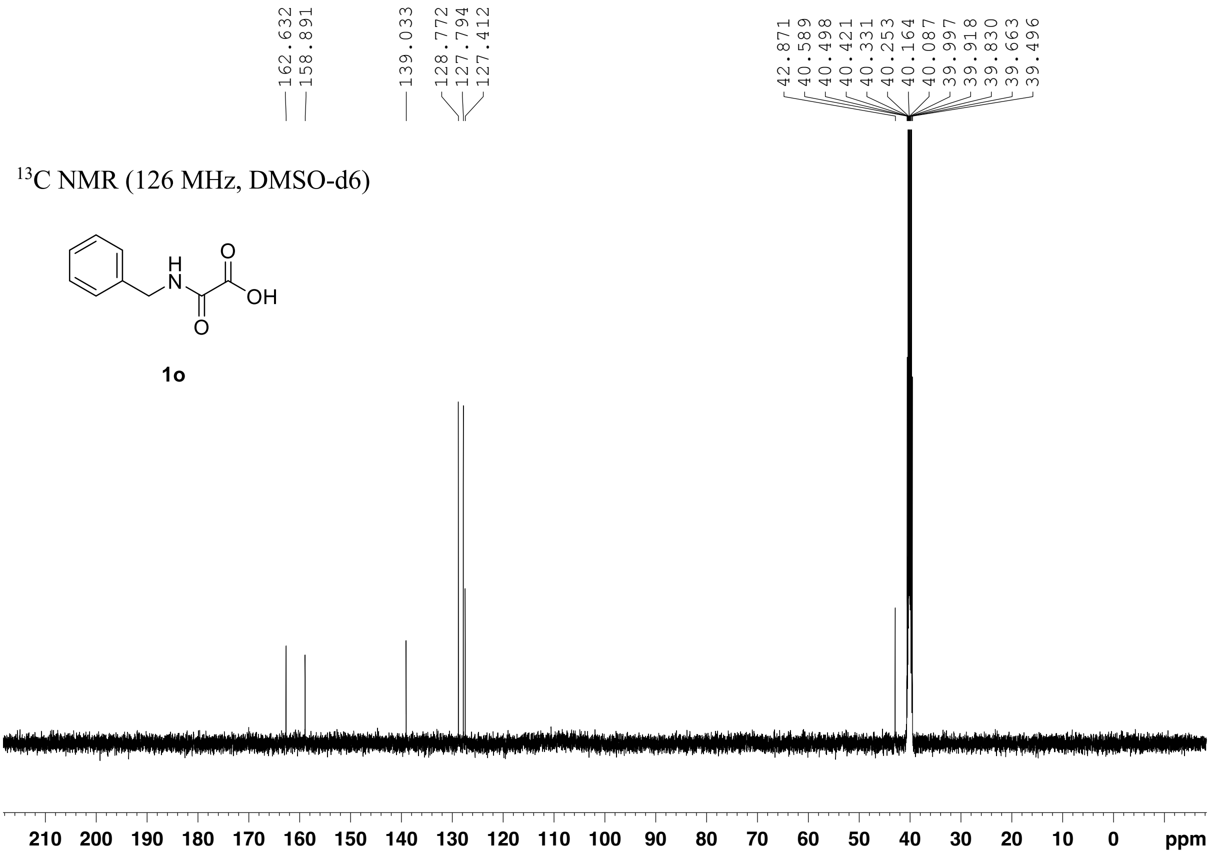

**Methyl 2-(isopropylamino)-2-oxoacetate S1p:**

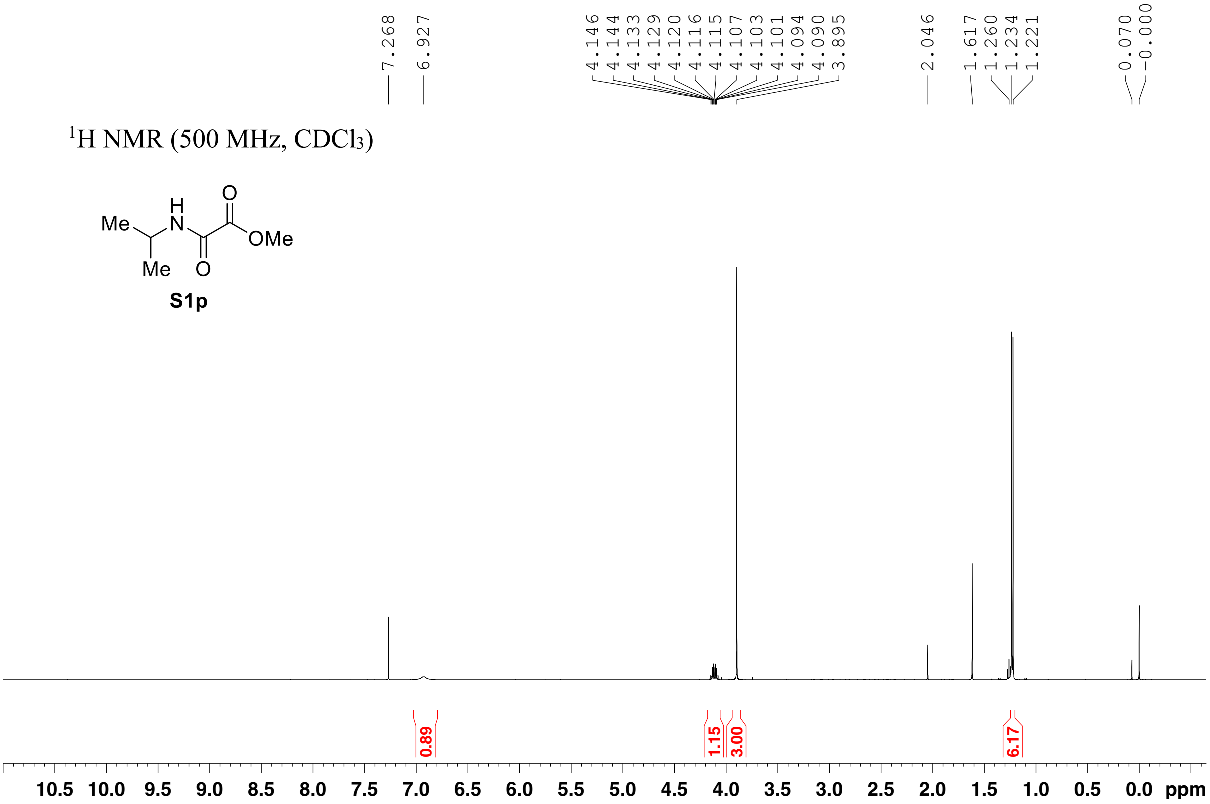


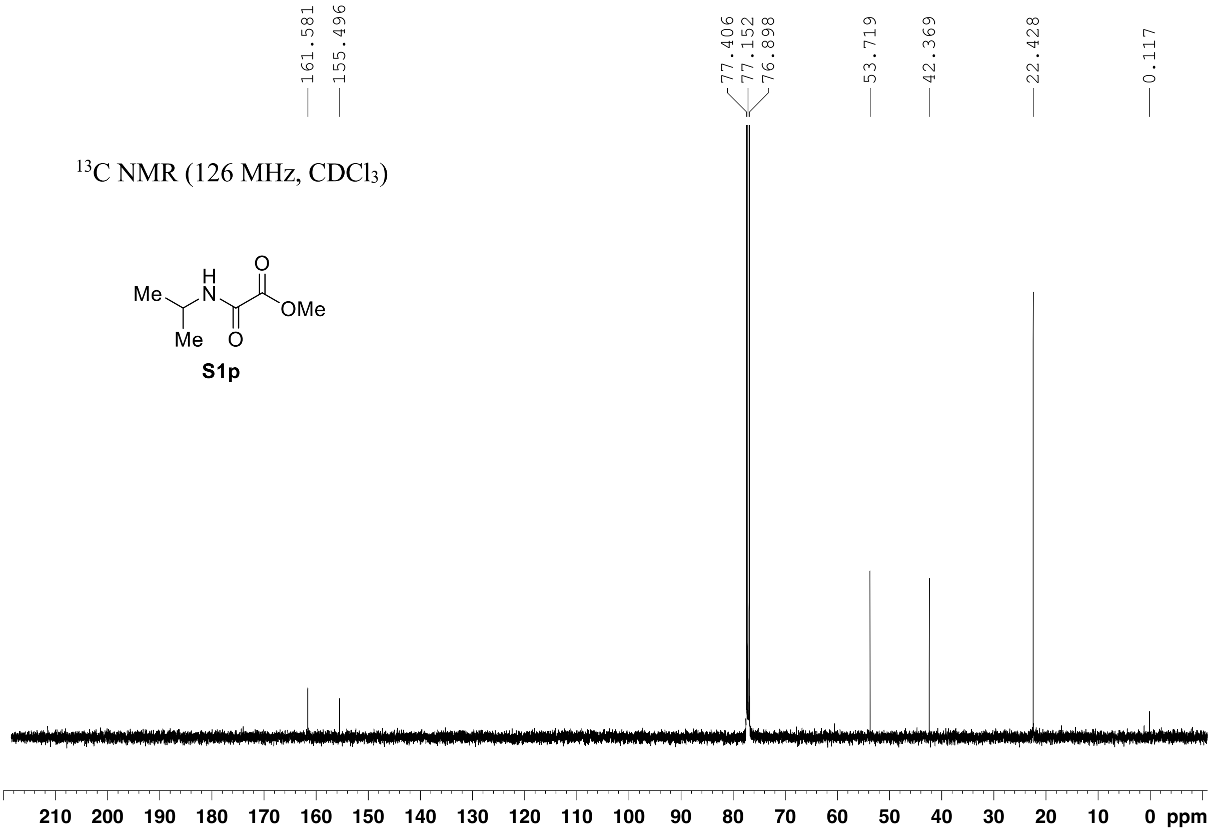

**2-(Isopropylamino)-2-oxoacetic acid 1p:**

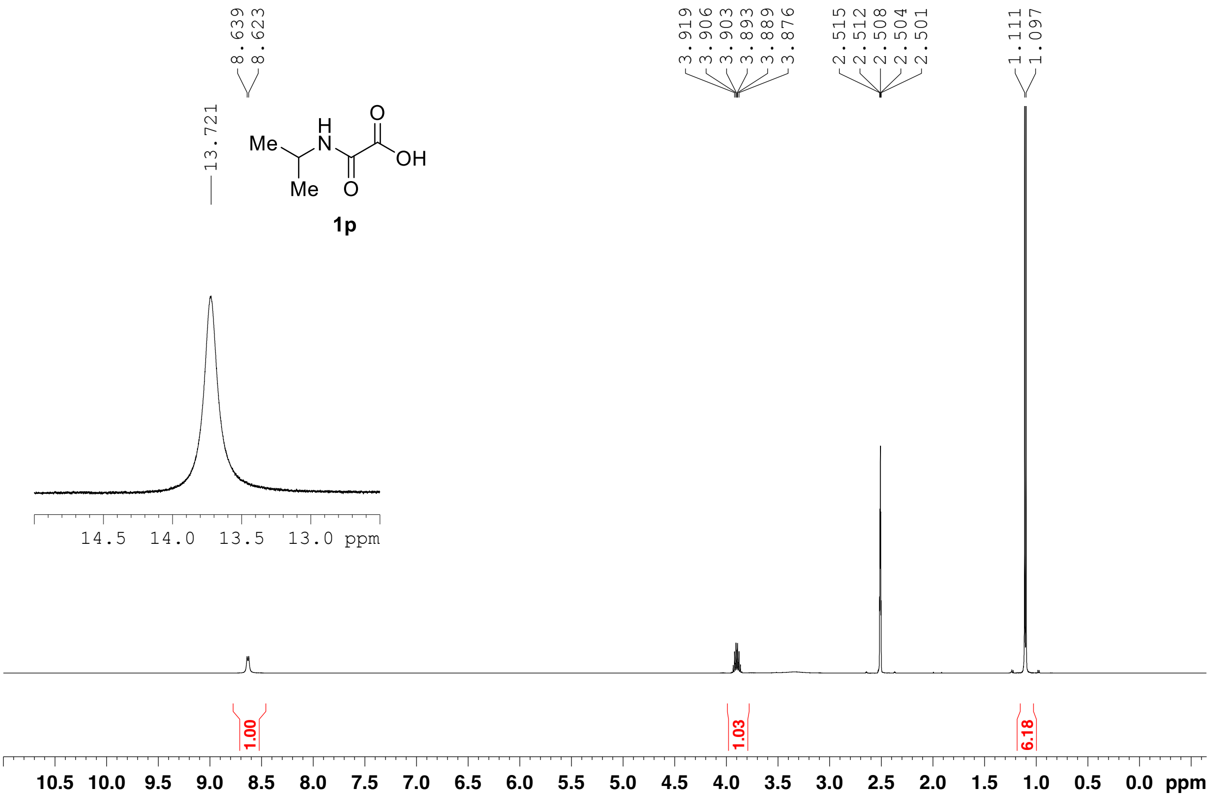


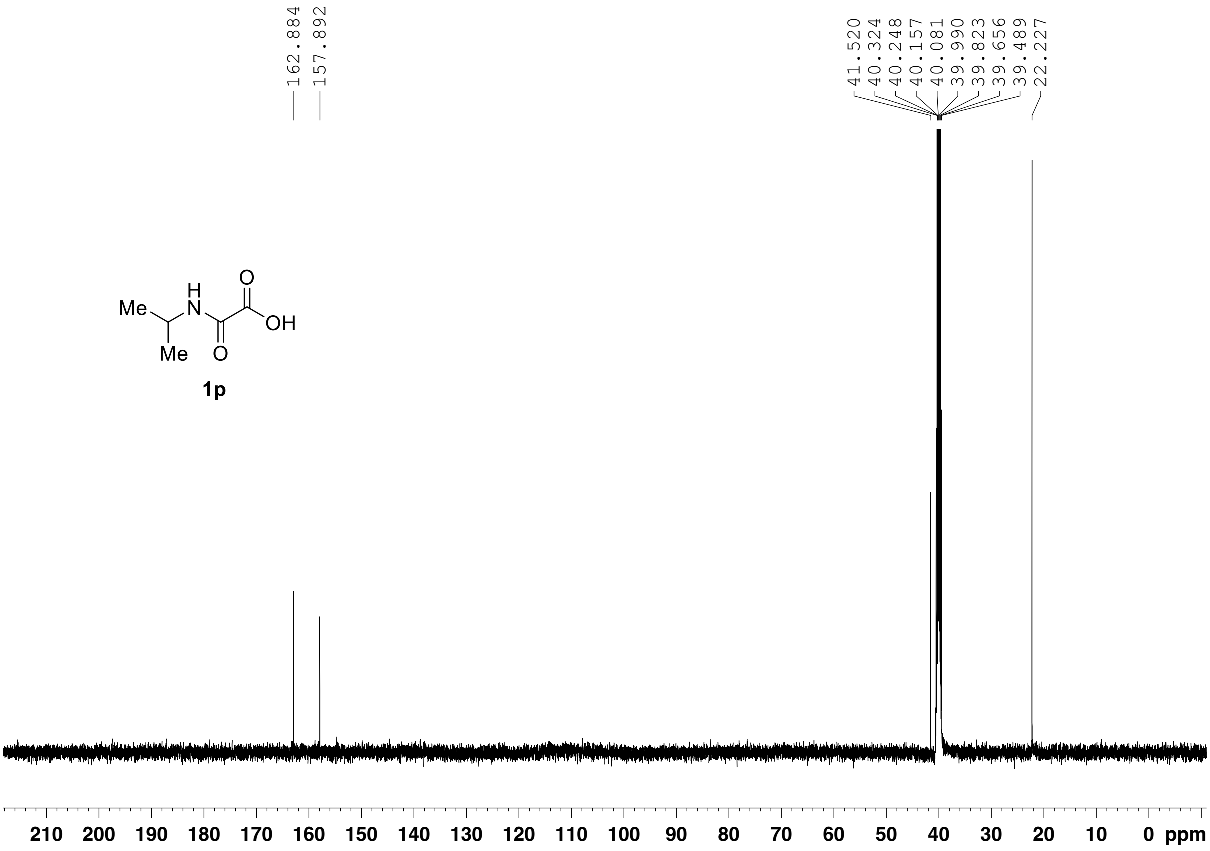

**4-Cyano-*N*-methyl-*N*-phenylbenzamide 2a:**

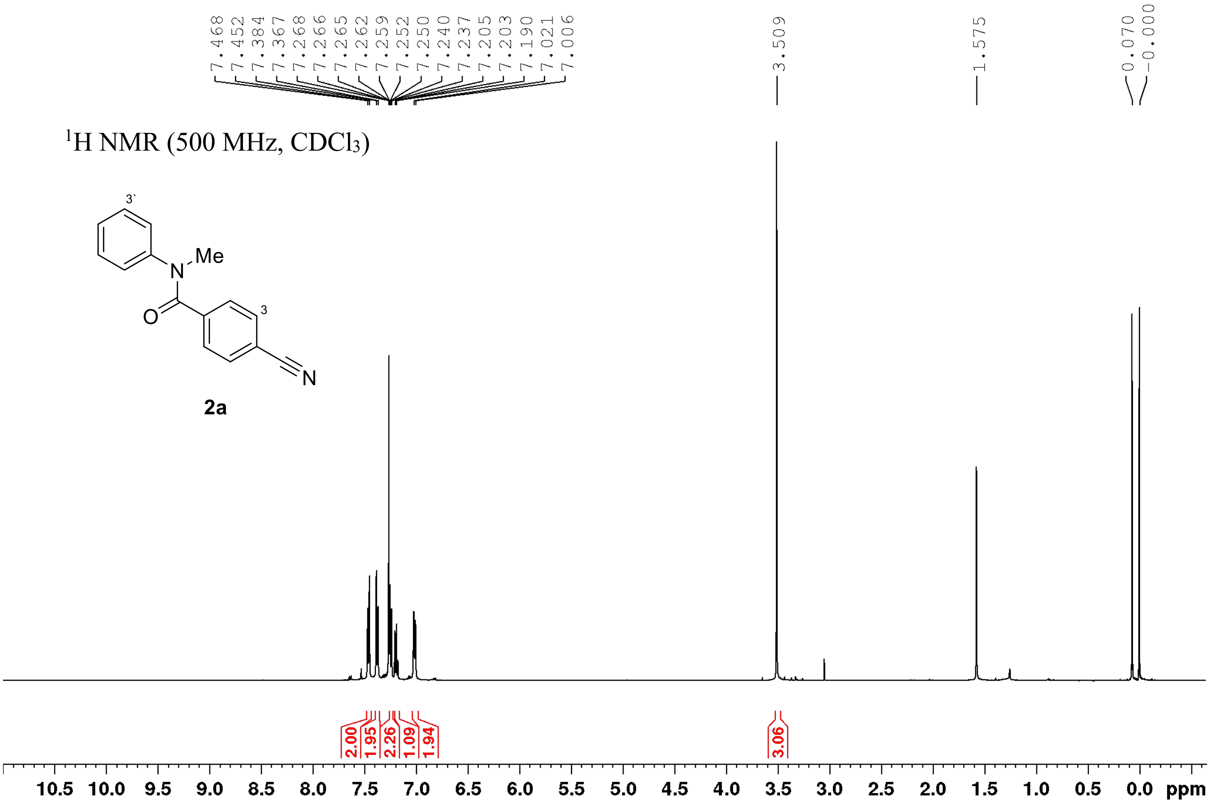


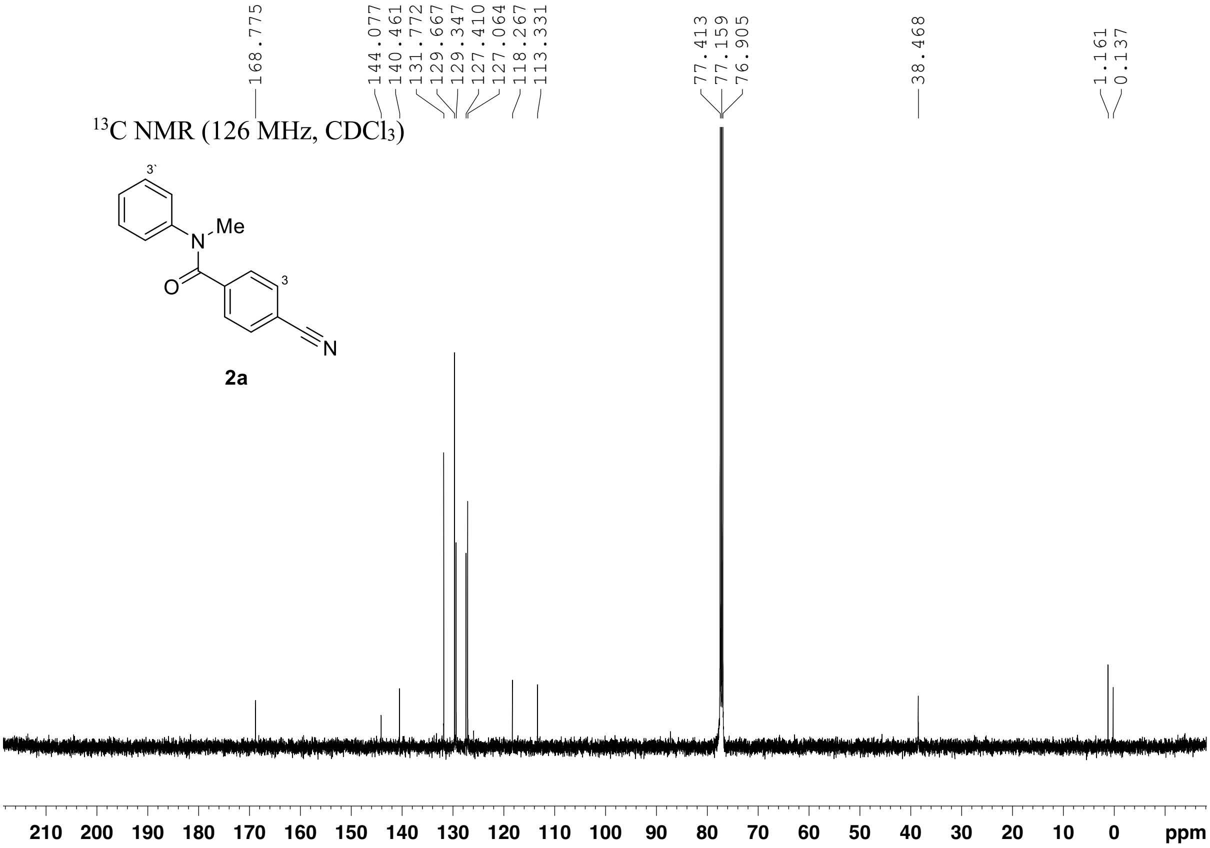

**4-Cyano-*N*-methyl-*N*-(*p*-tolyl)benzamide 2b:**

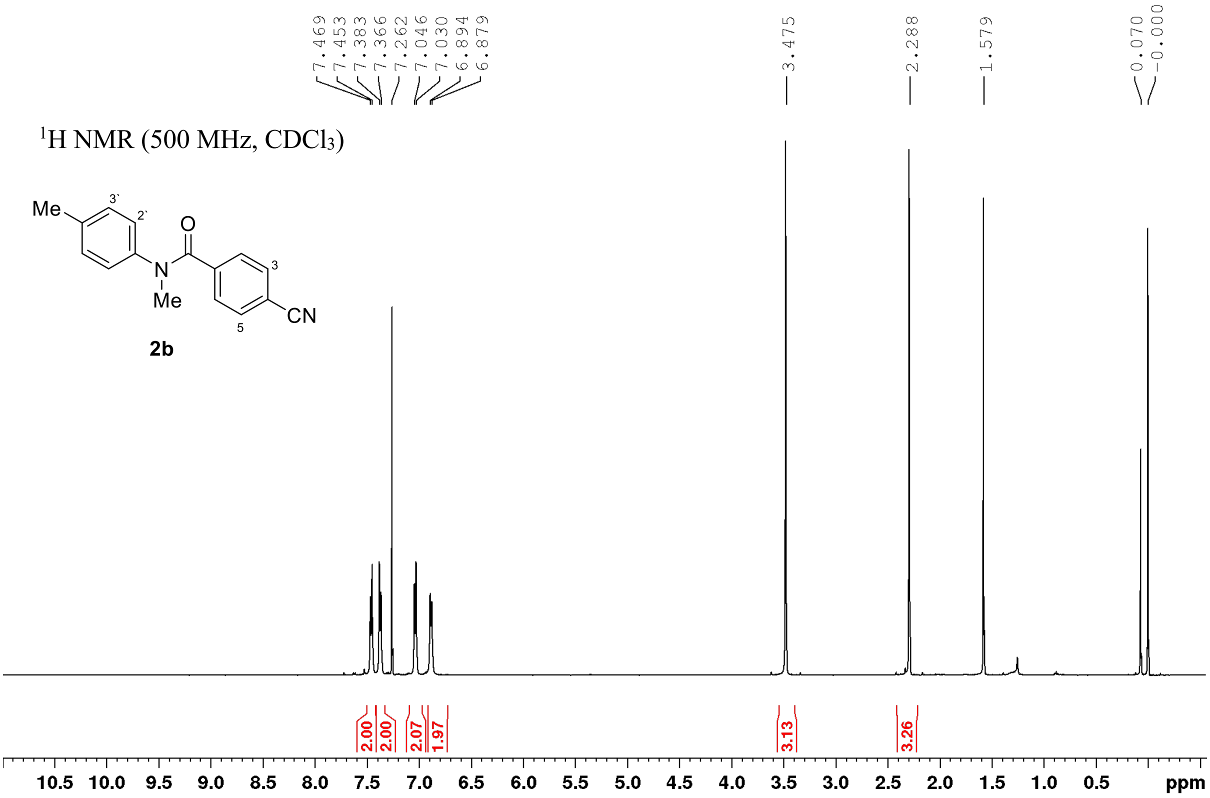


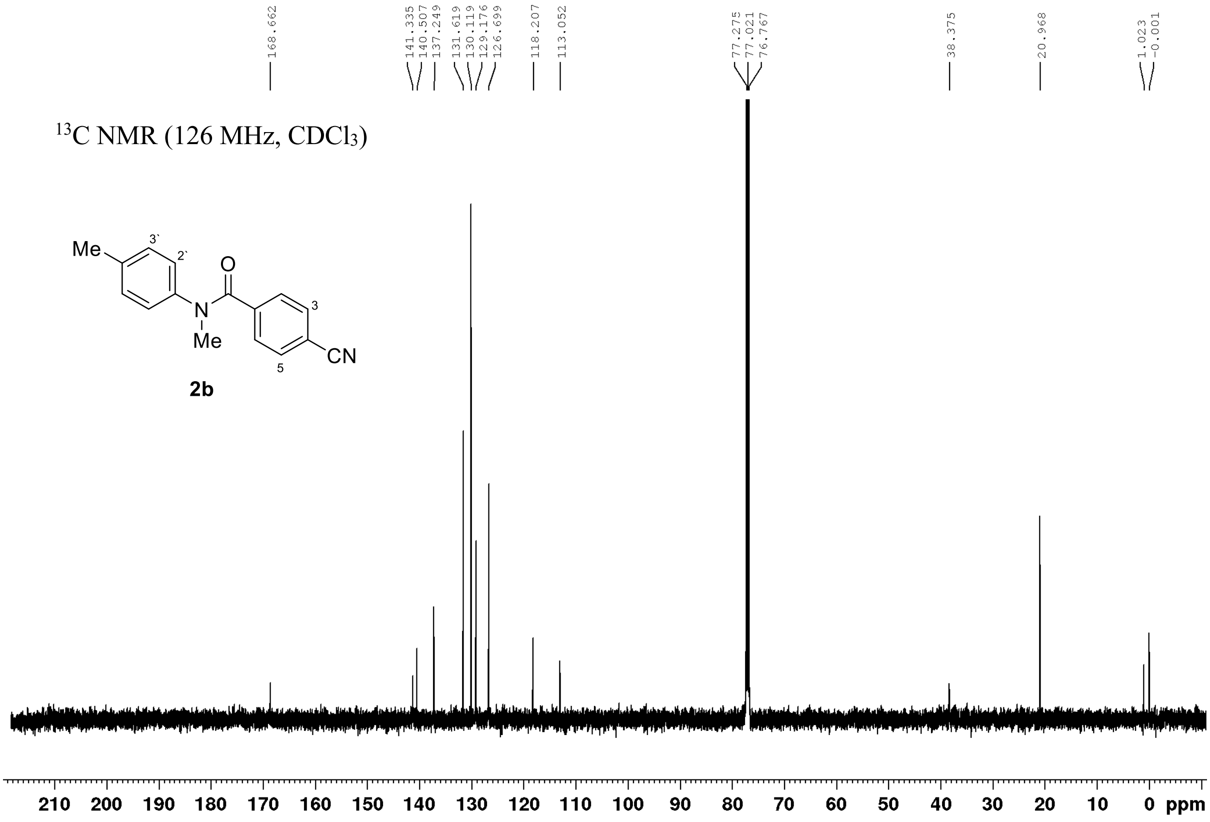

**$N$-(4-chlorophenyl)-4-cyano-$N$-methylbenzamide 2c:**

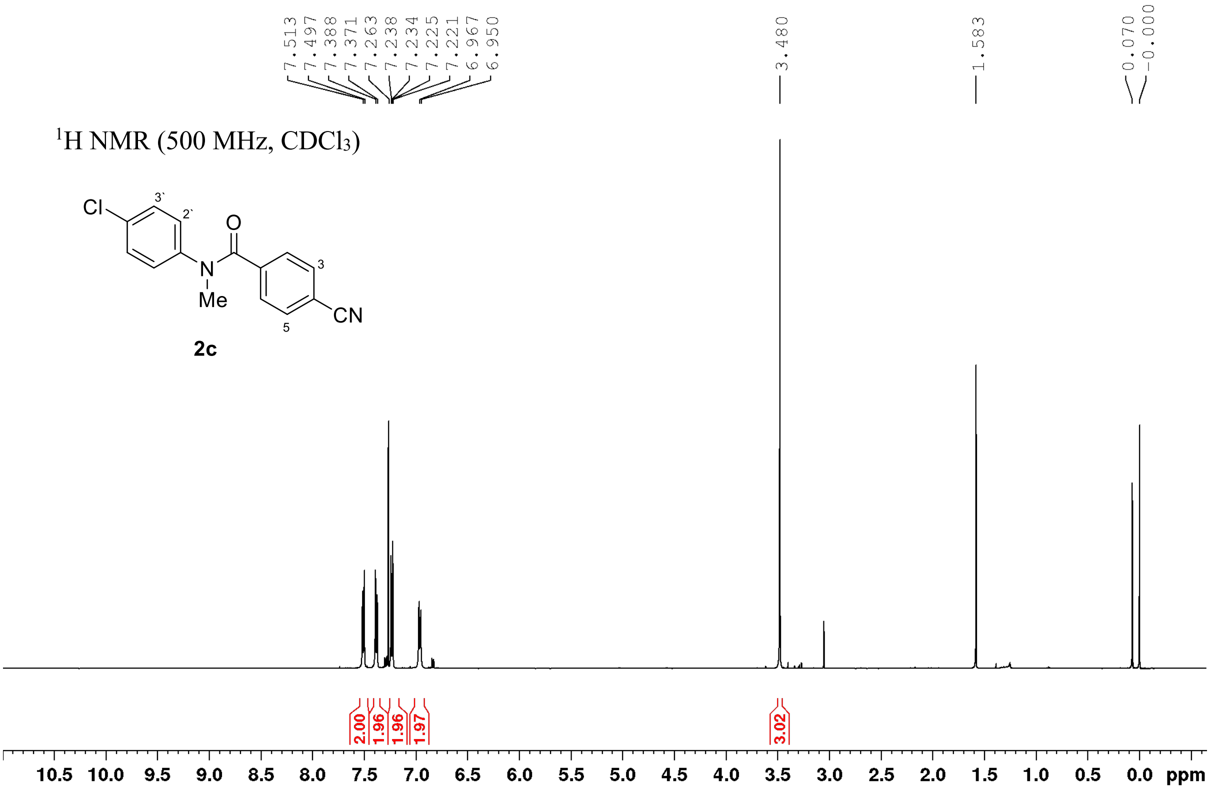


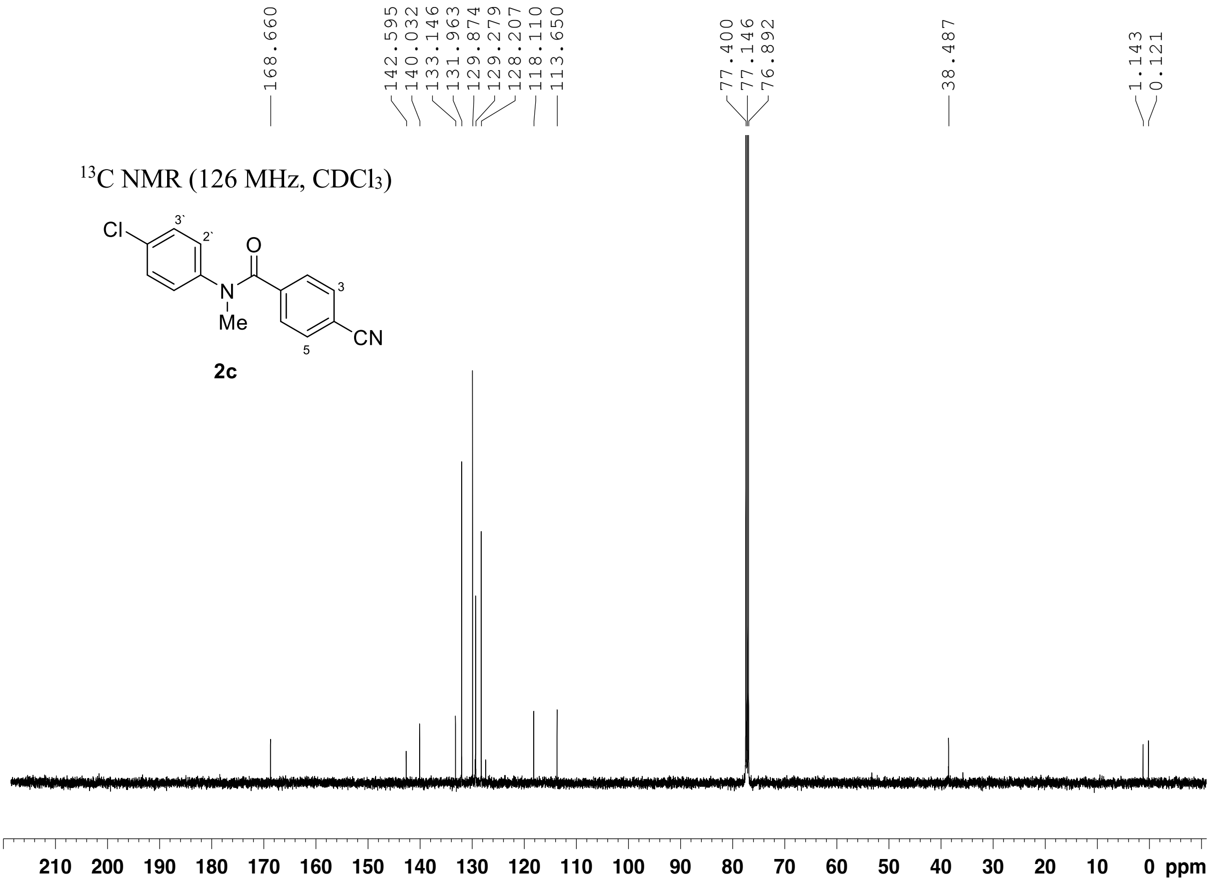

**4-Cyano-*N*-(4-fluorophenyl)-*N*-methylbenzamide 2d:**

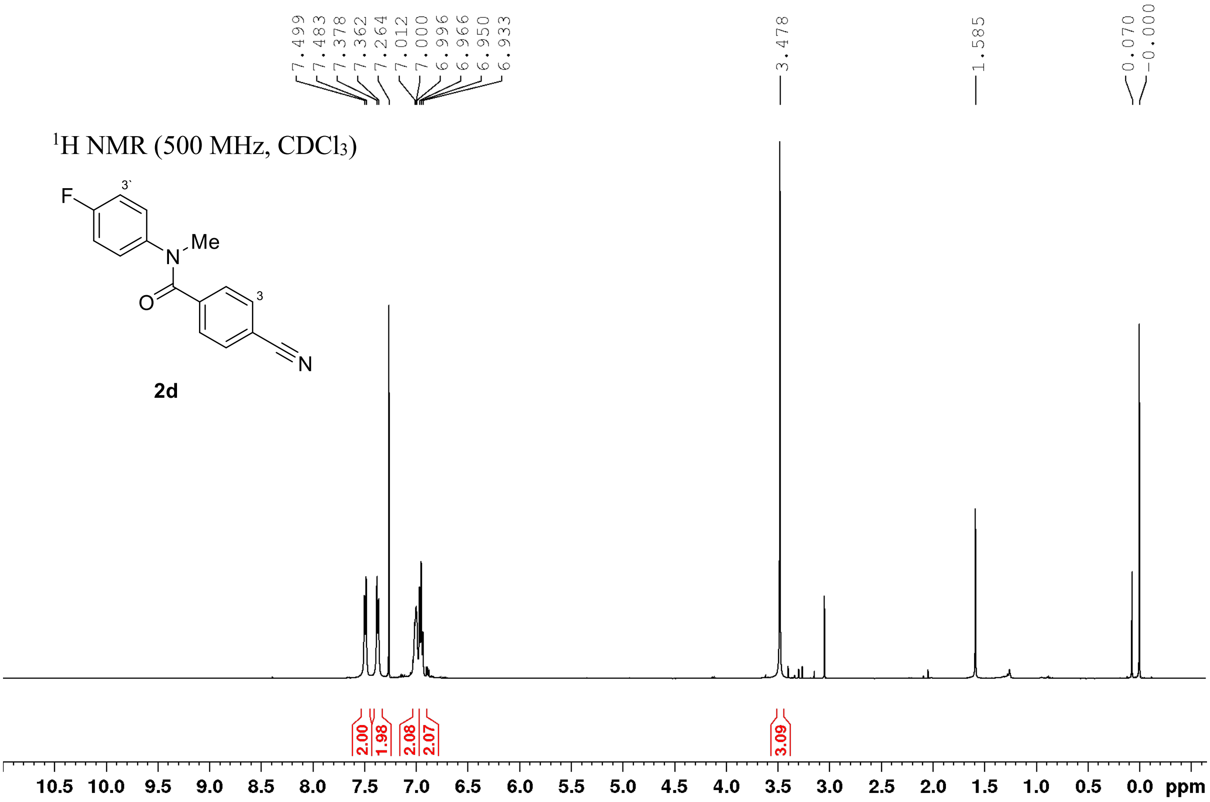


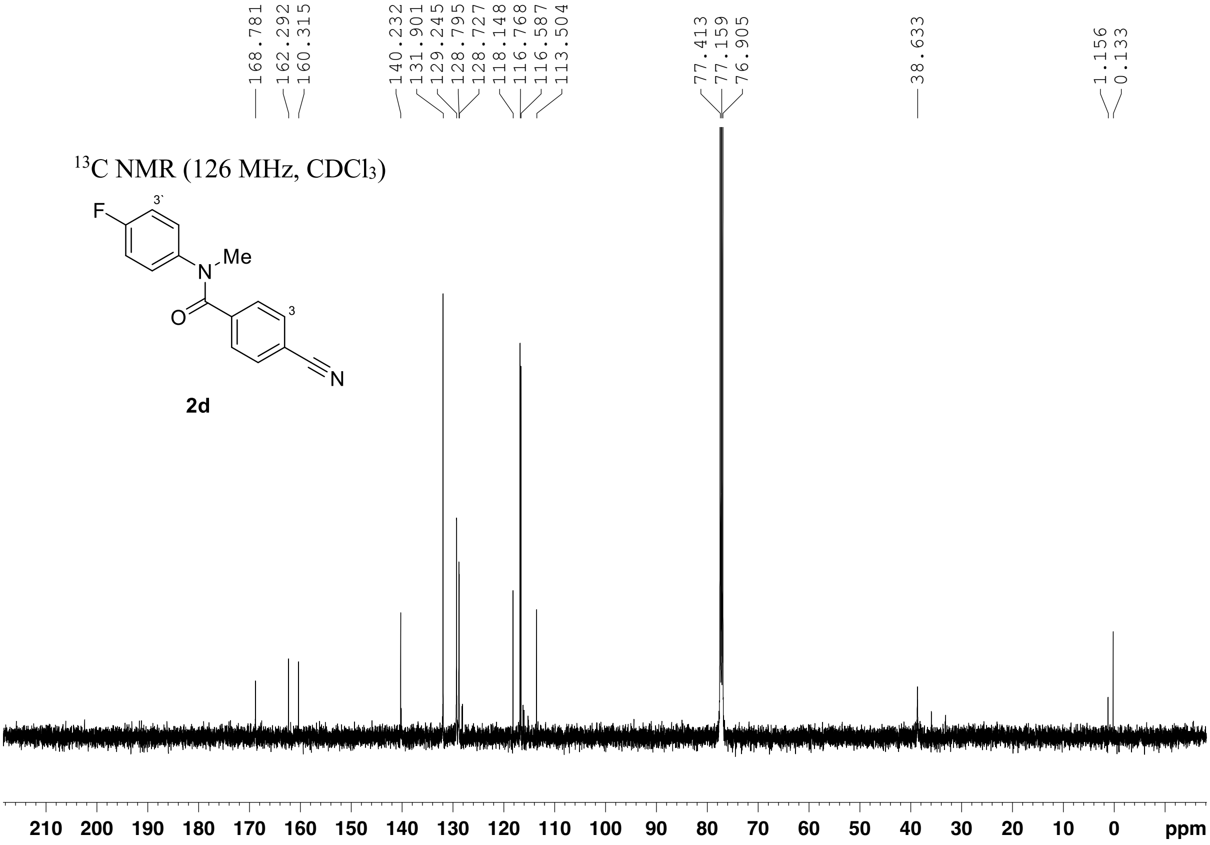

$^{19}$F NMR (471 MHz, $CDCl_3$)

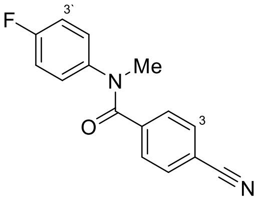

**2d**

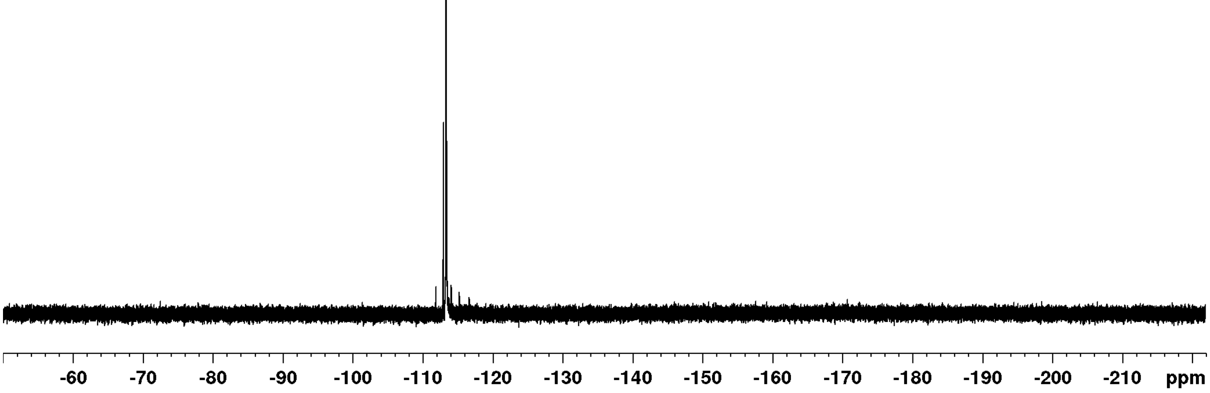

**4-Cyano-*N*,*N*-diphenylbenzamide 2e:**

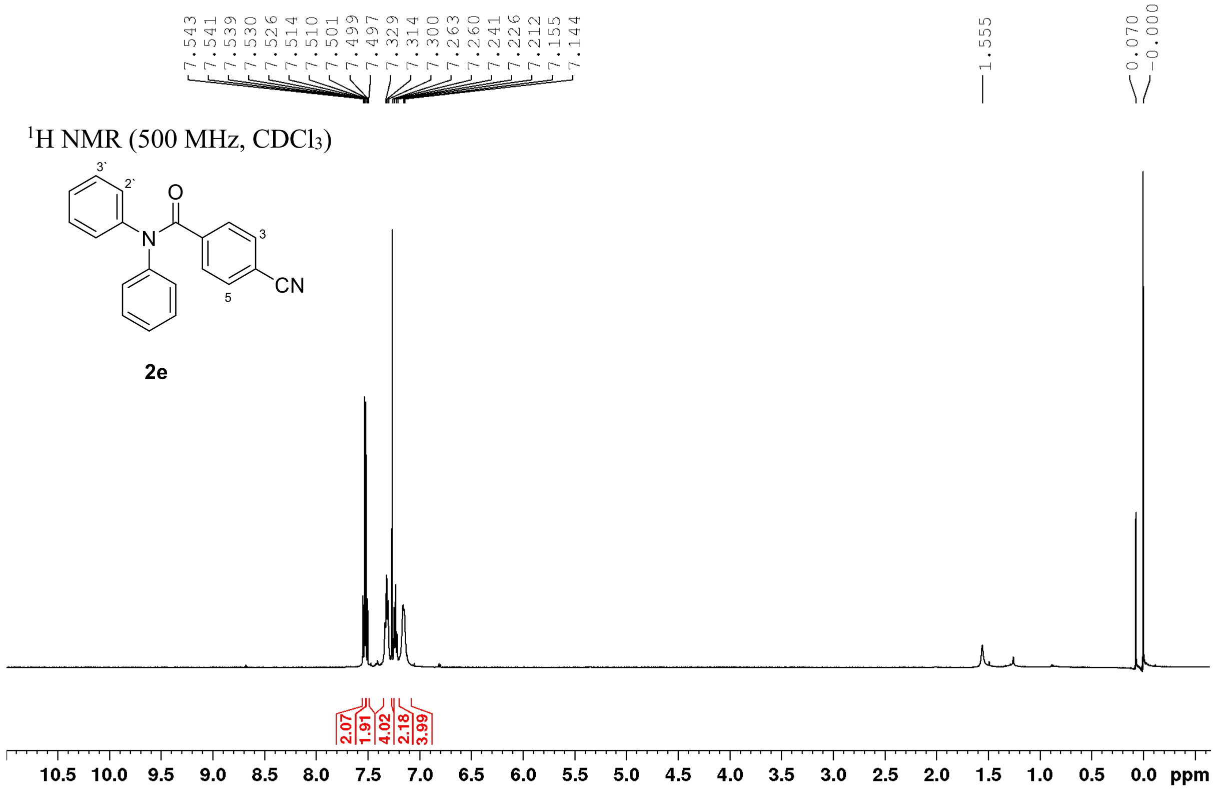


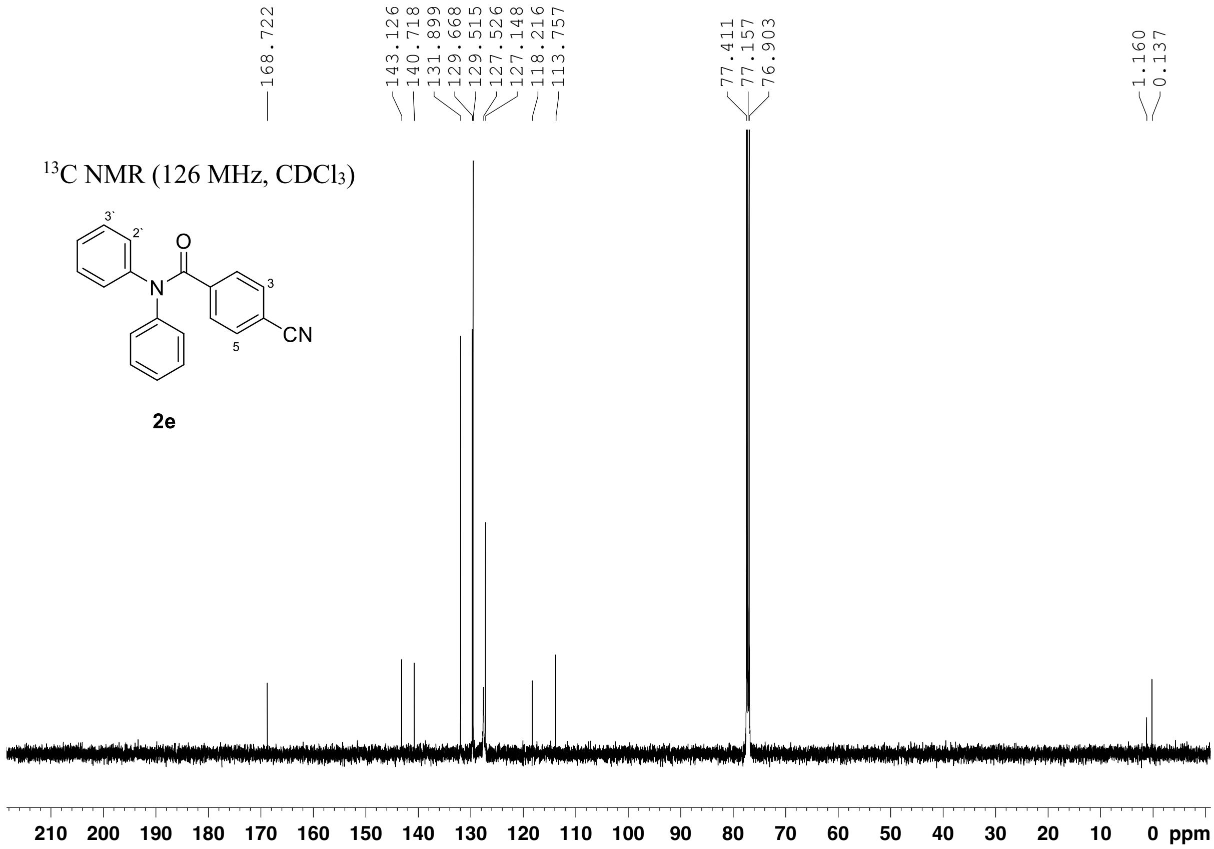

**4-(Morpholine-4-carbonyl)benzonitrile methane 2f:**

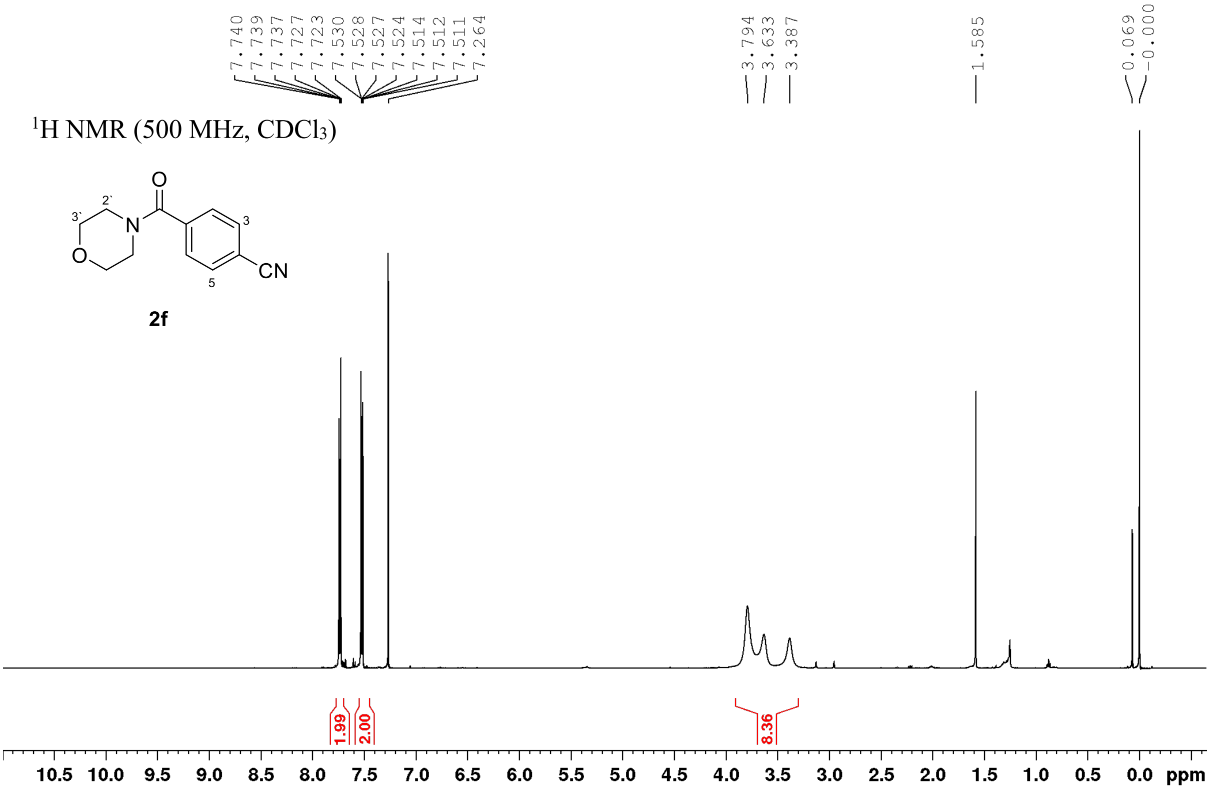


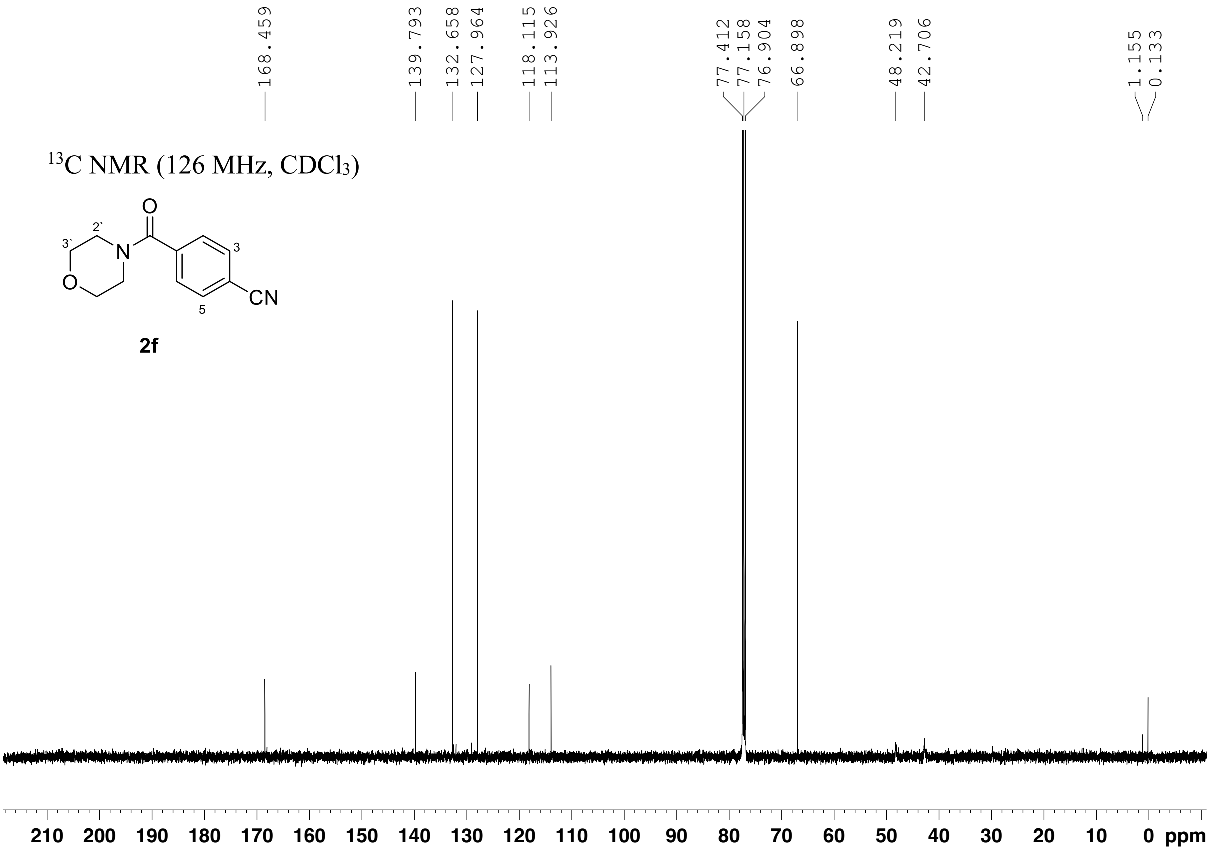

**N-((3s,5s,7s)-Adamantan-1-yl)-4-cyano-N-methylbenzamide 2g:**

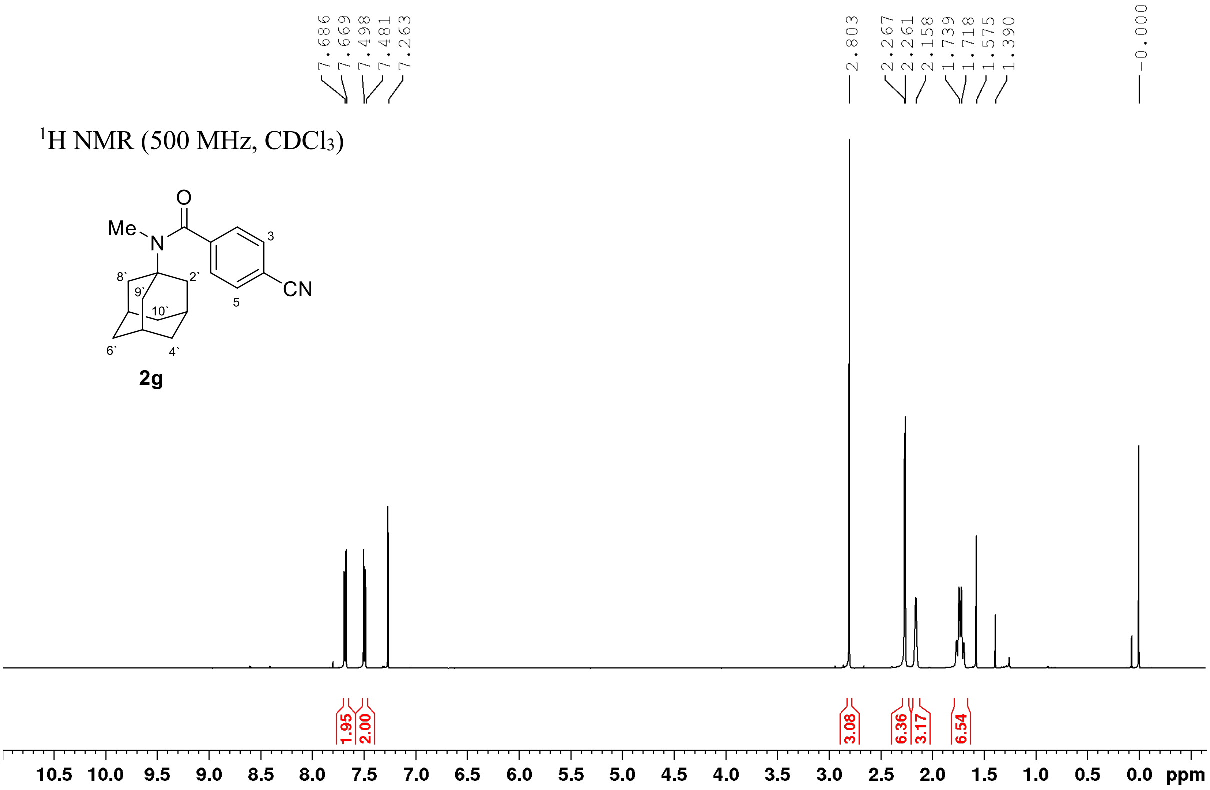


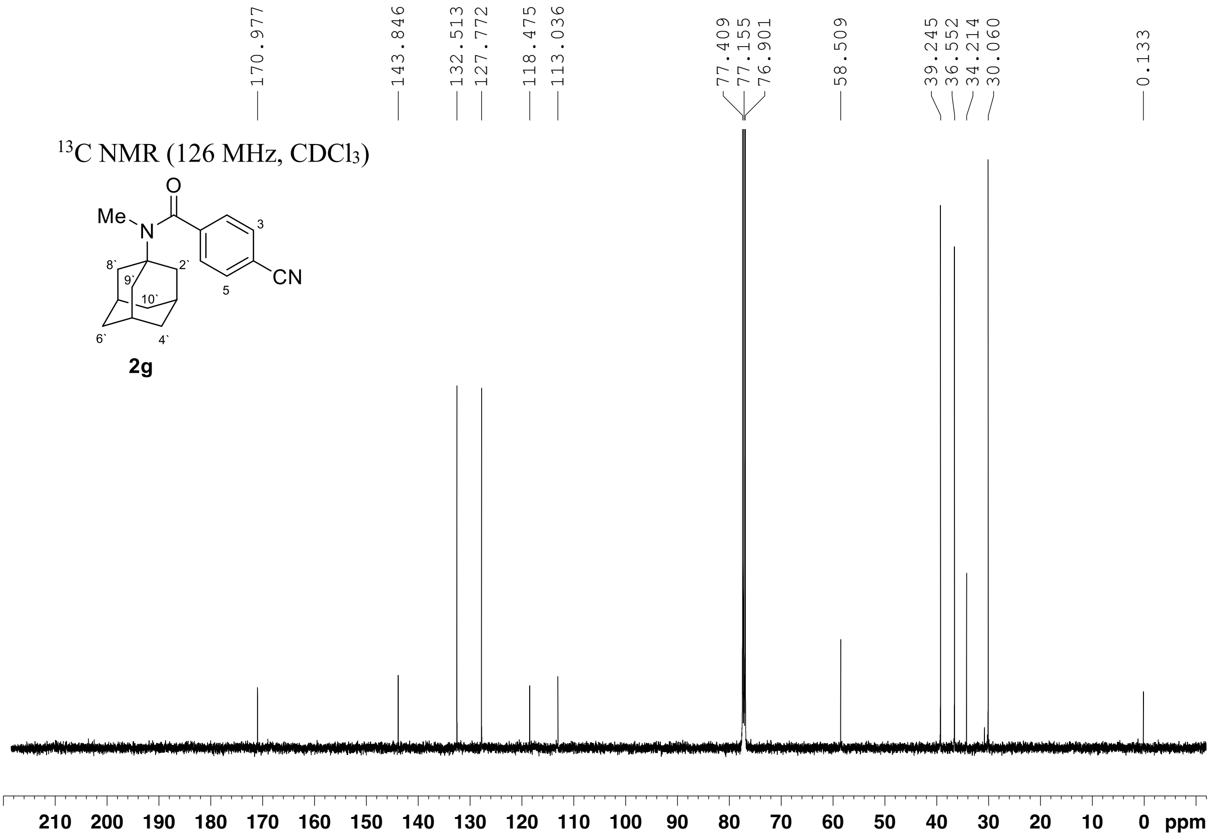

## *N*-Benzyl-4-cyano-*N*-phenylbenzamide 2h:

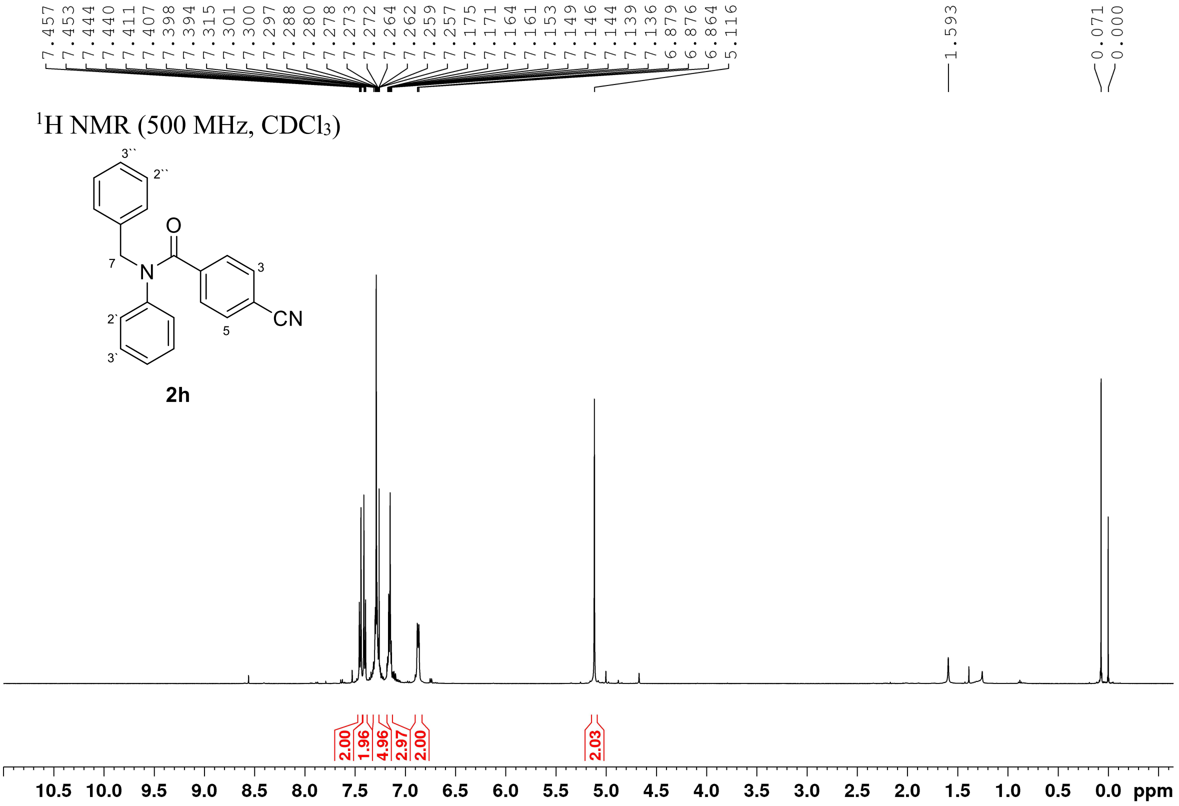


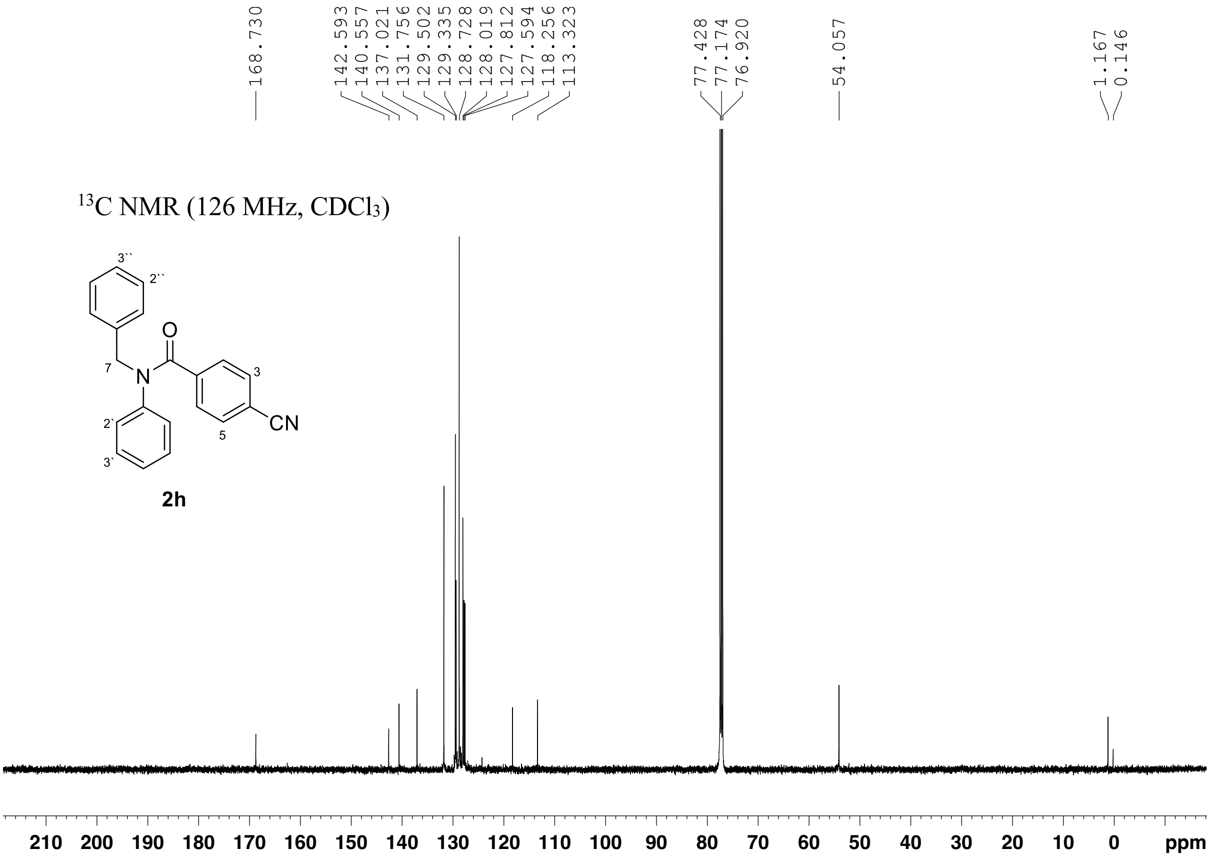

**4-(1,2,3,4-Tetrahydroquinoline-1-carbonyl)benzonitrile 2i:**

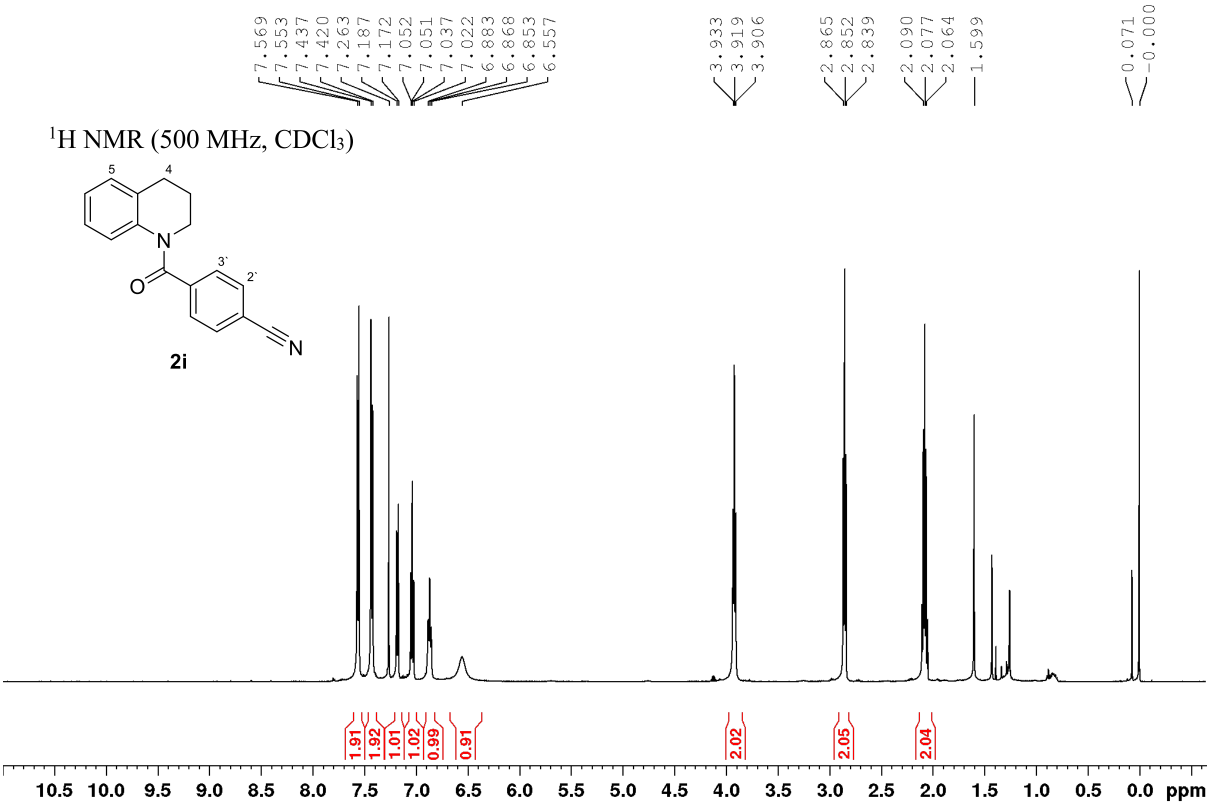


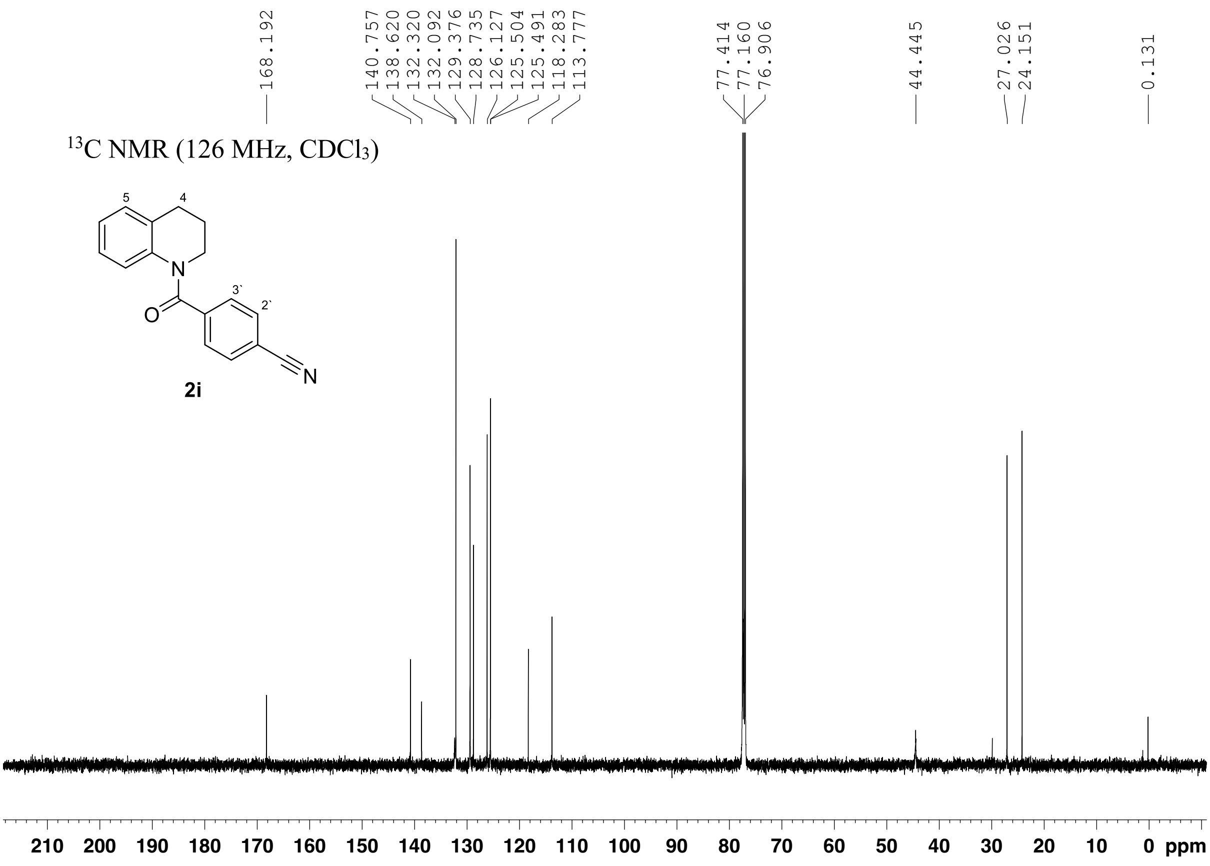

**4-Cyano-*N*-(4-methoxyphenyl)-*N*-methylbenzamide 2j:**

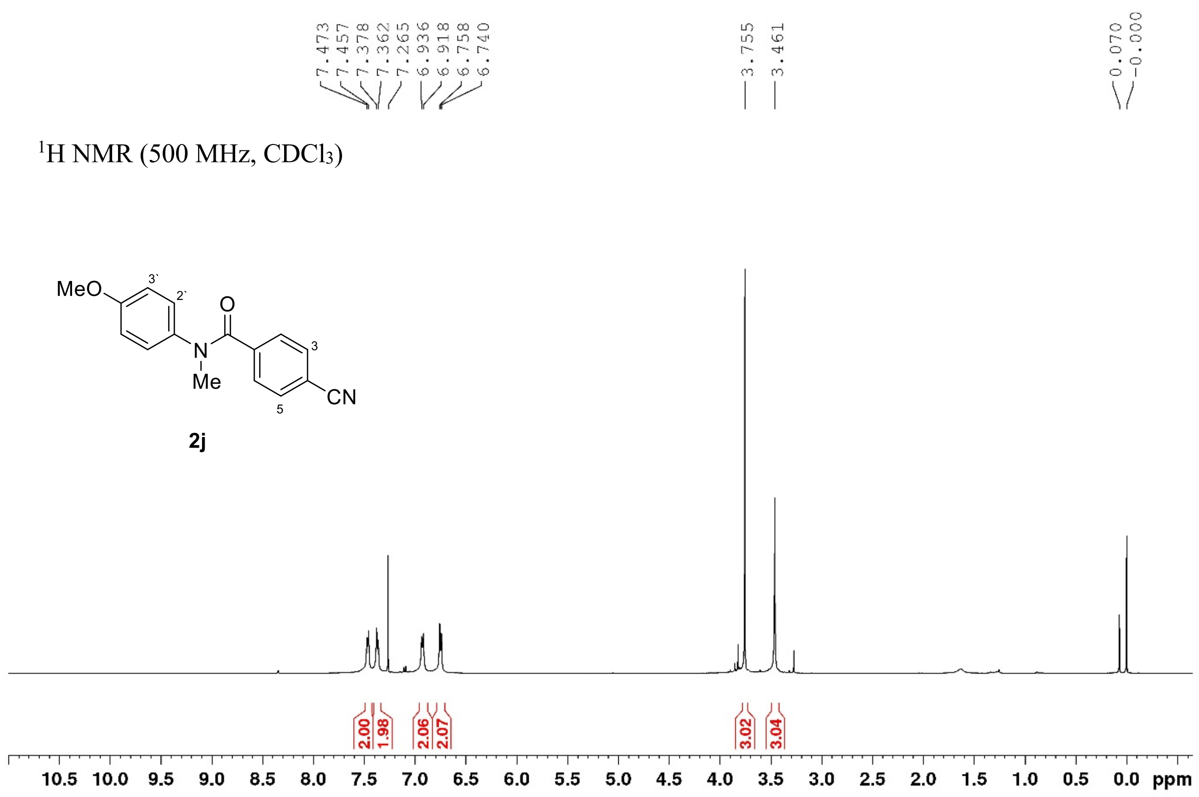


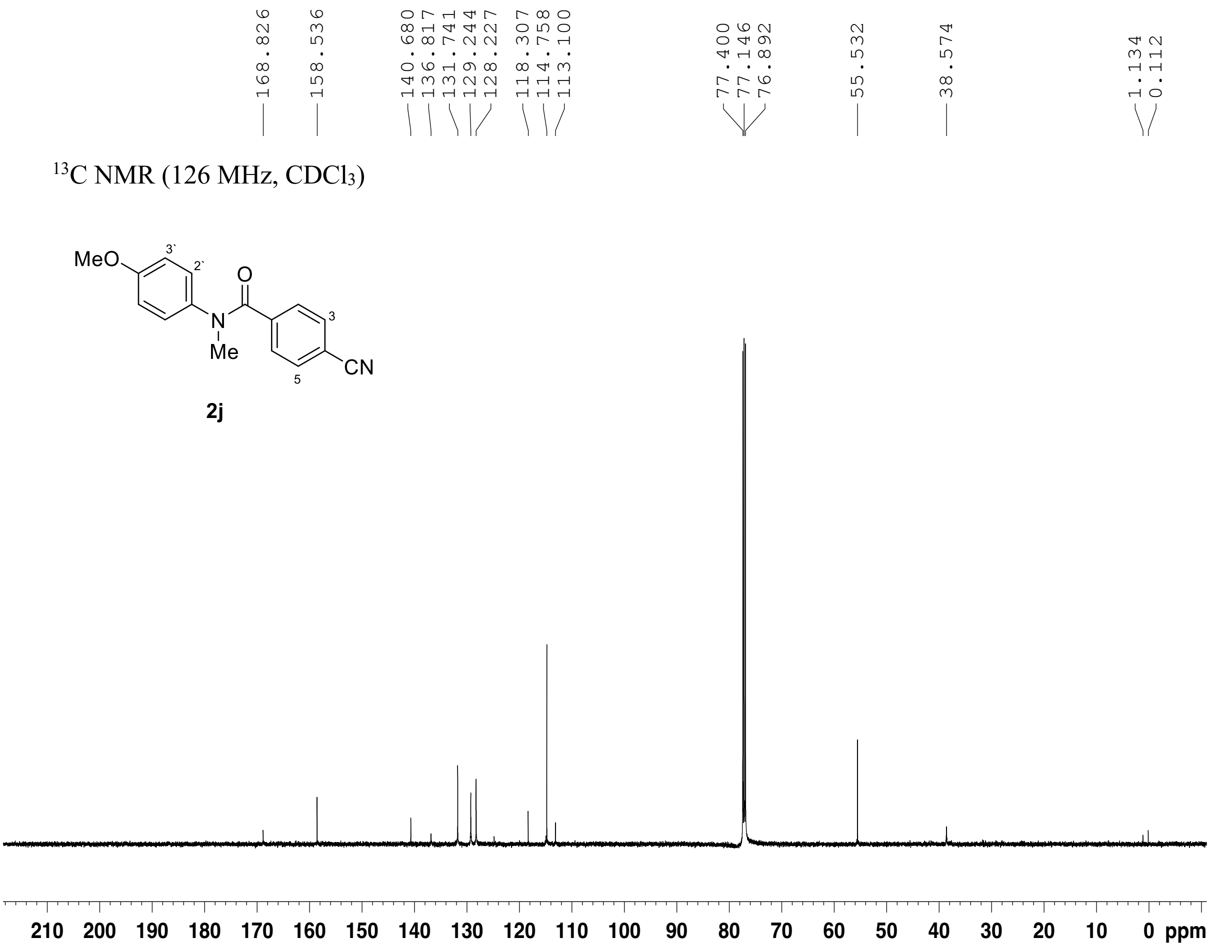

**4-Cyano-*N*-isopropyl-*N*-phenylbenzamide 2k:**

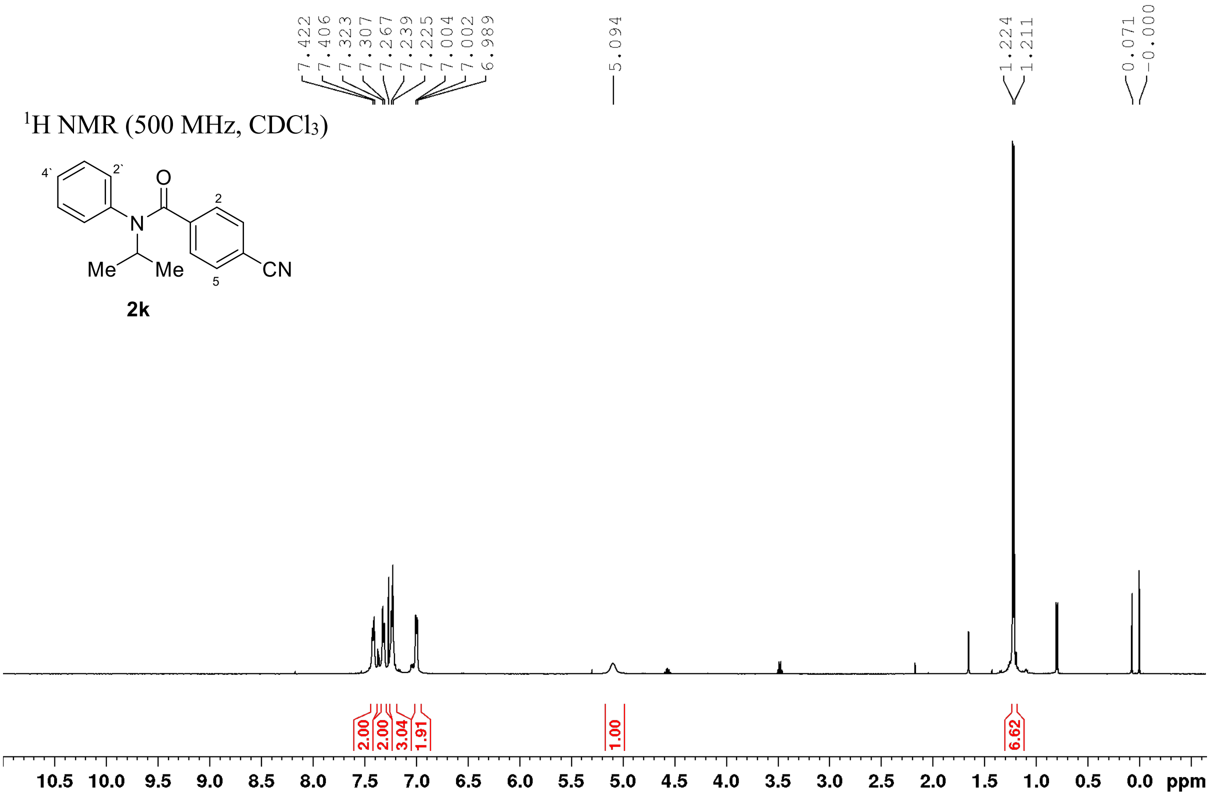


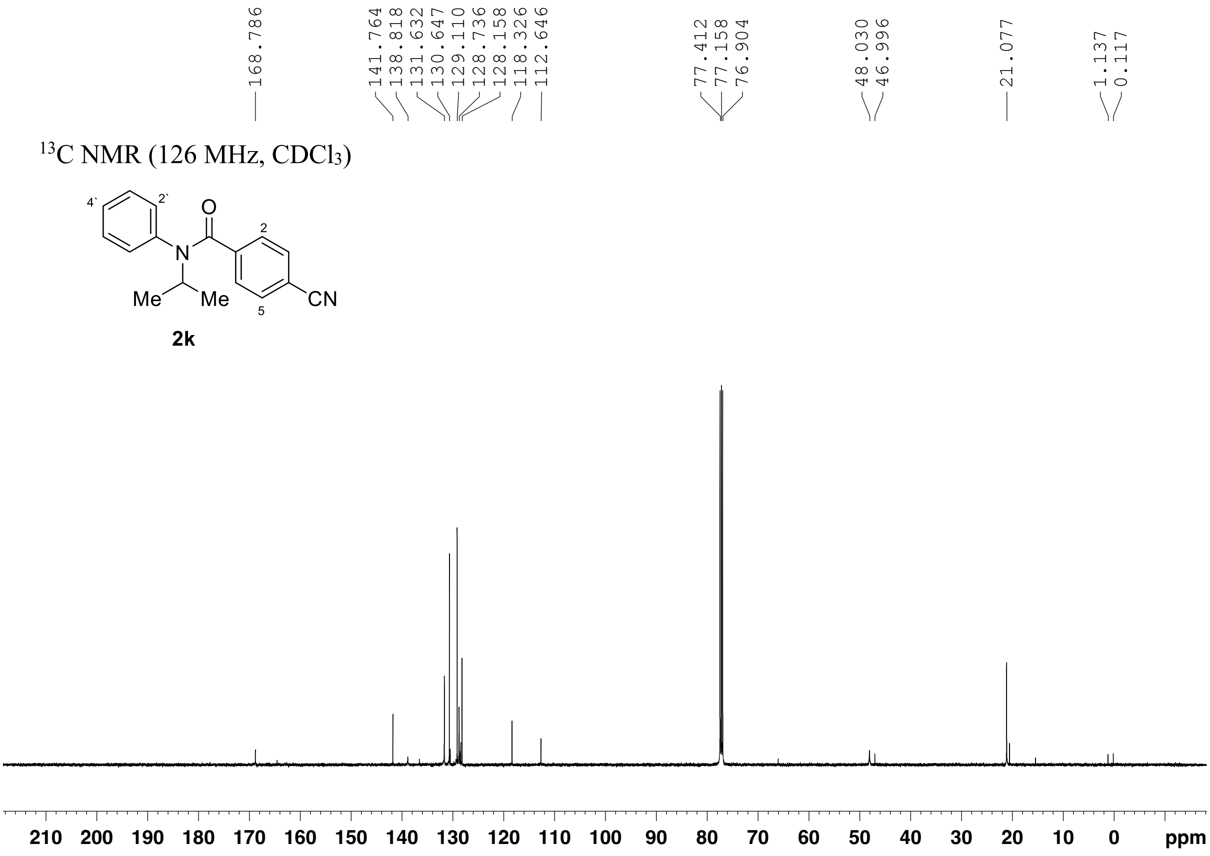

**4-Cyano-*N*-methyl-*N*-(*o*-tolyl)benzamide 2l:**

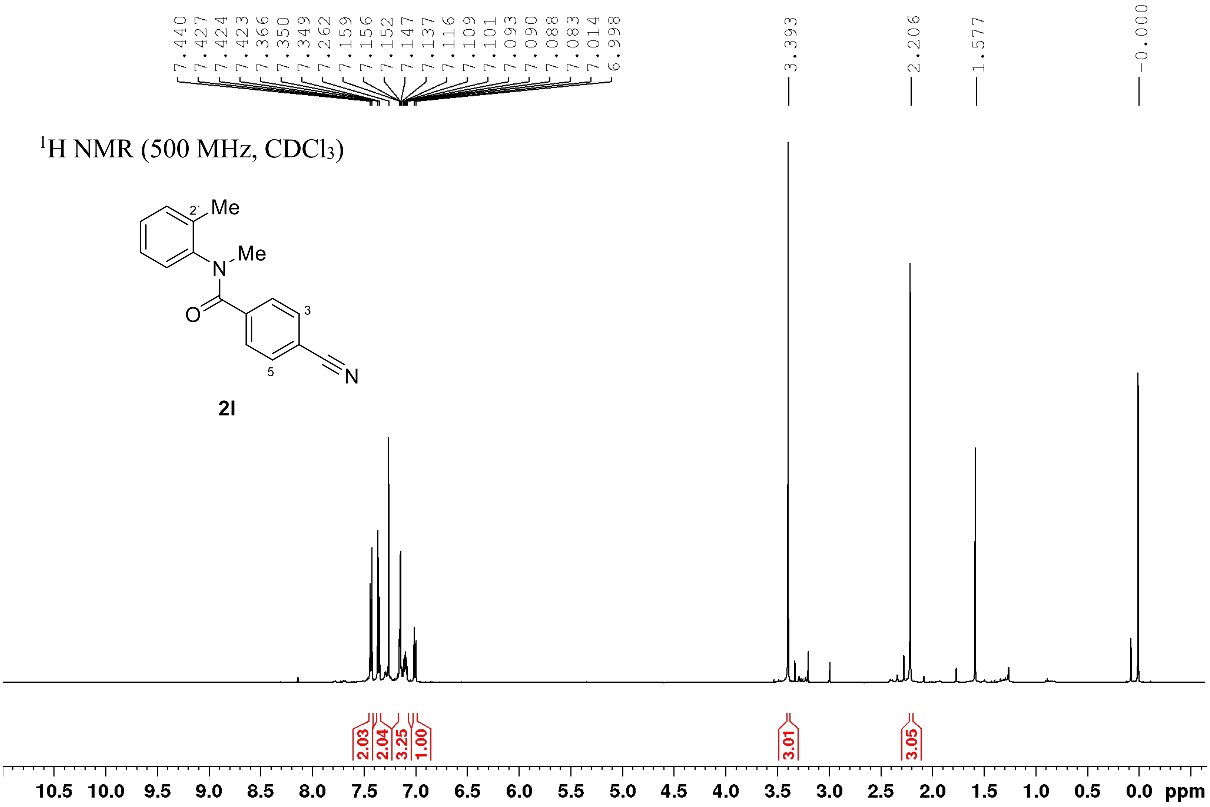


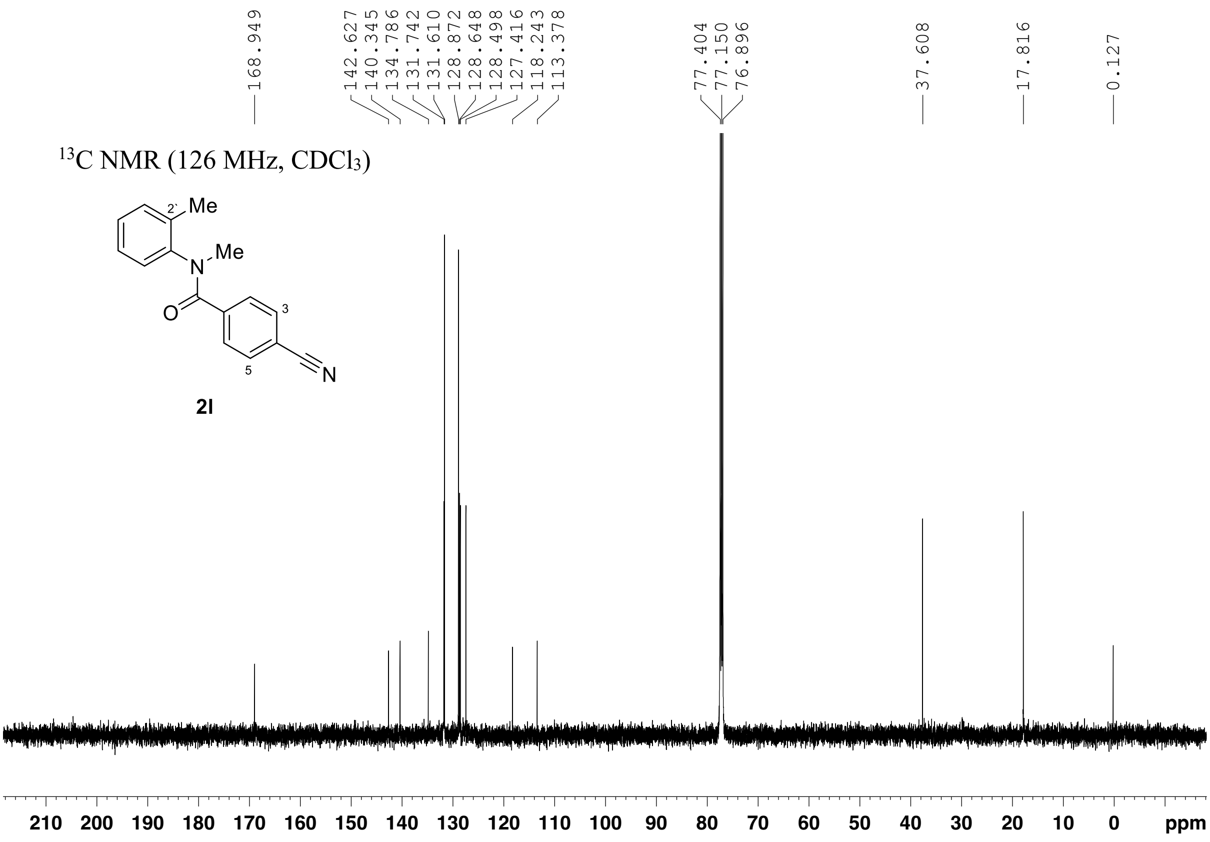

***N*-methyl-*N*-phenyl-4-(trifluoromethyl)benzamide 3a:**

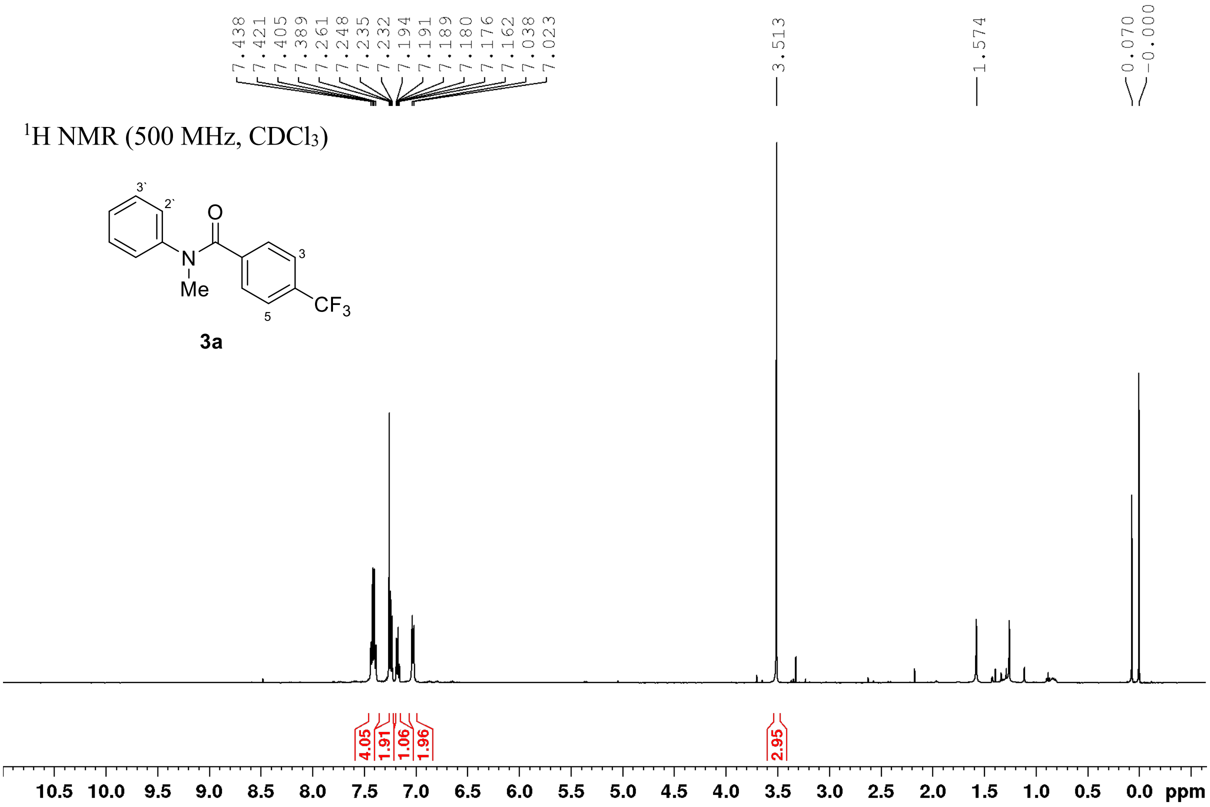


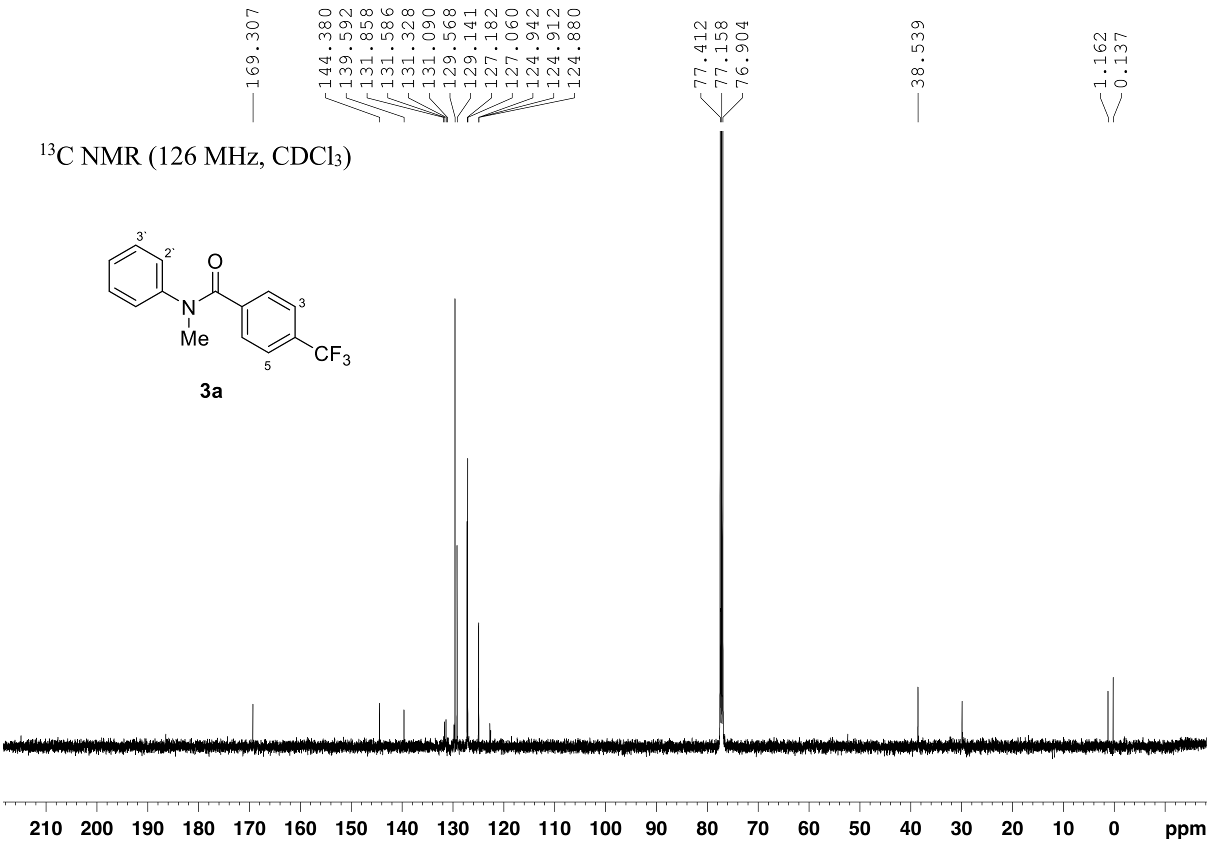

$^{19}F$ NMR (471 MHz, $CDCl_3$)

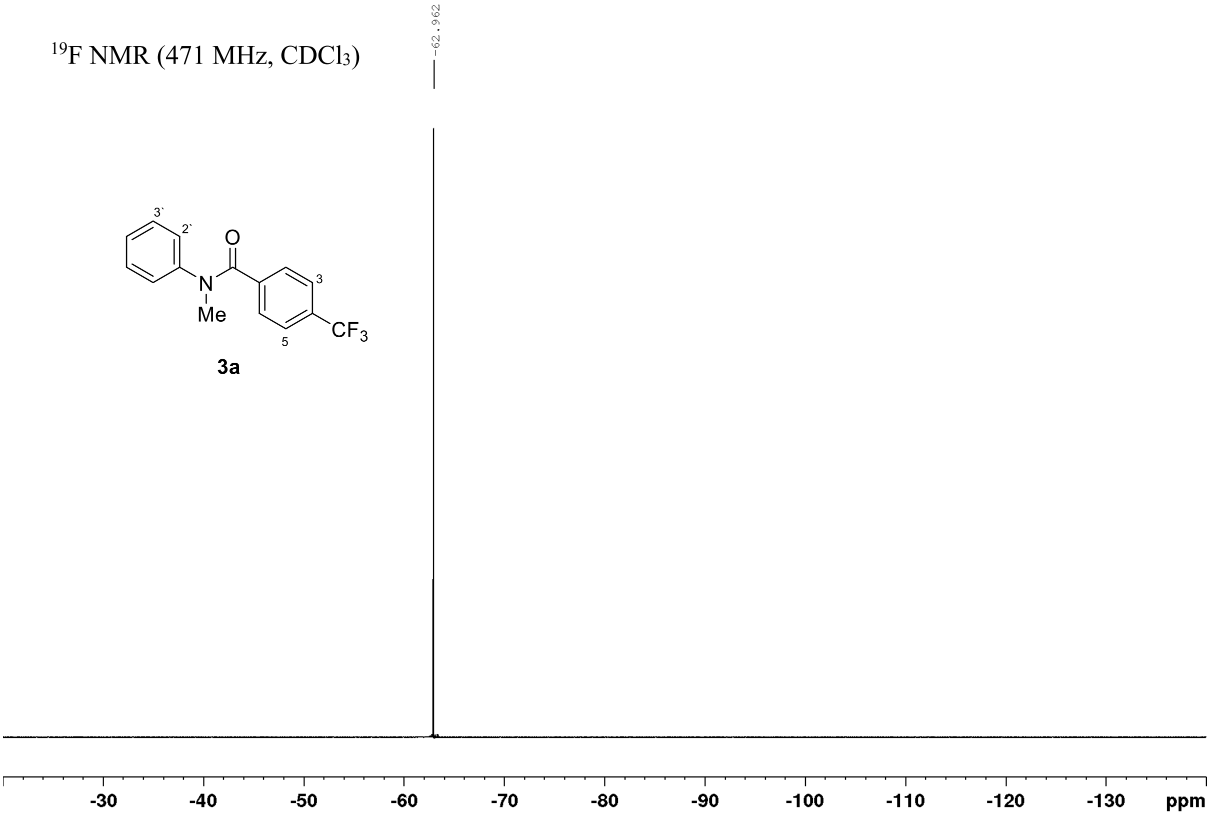

**3-Cyano-*N*-methyl-*N*-phenylbenzamide 3b:**

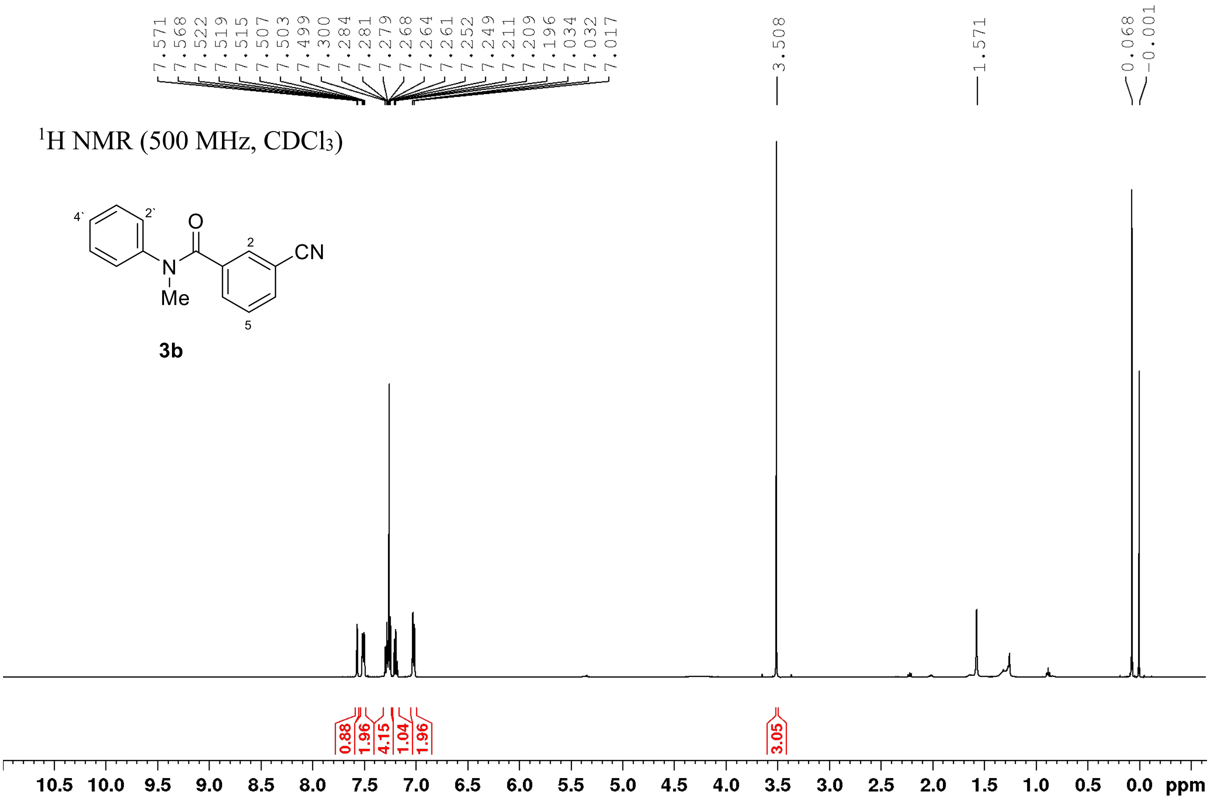


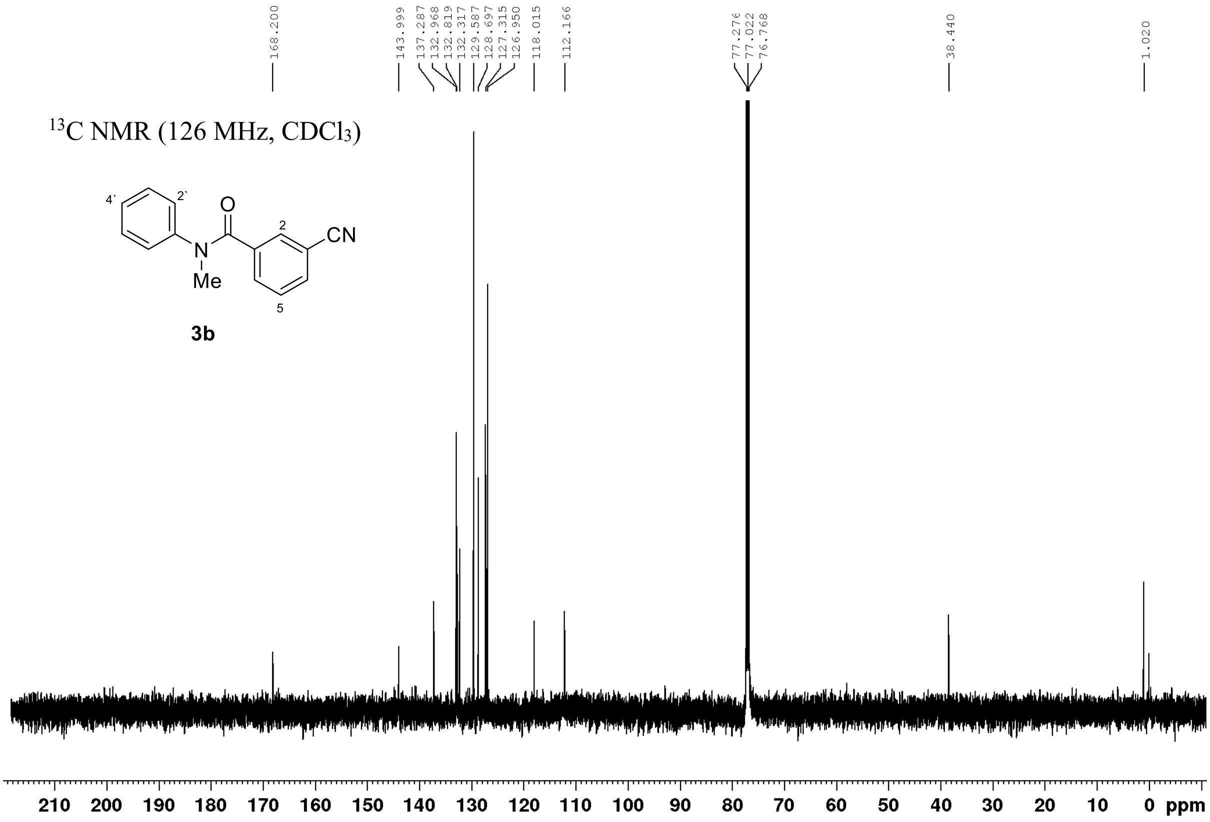

**Methyl 4-(methyl(phenyl)carbamoyl)benzoate 3c:**

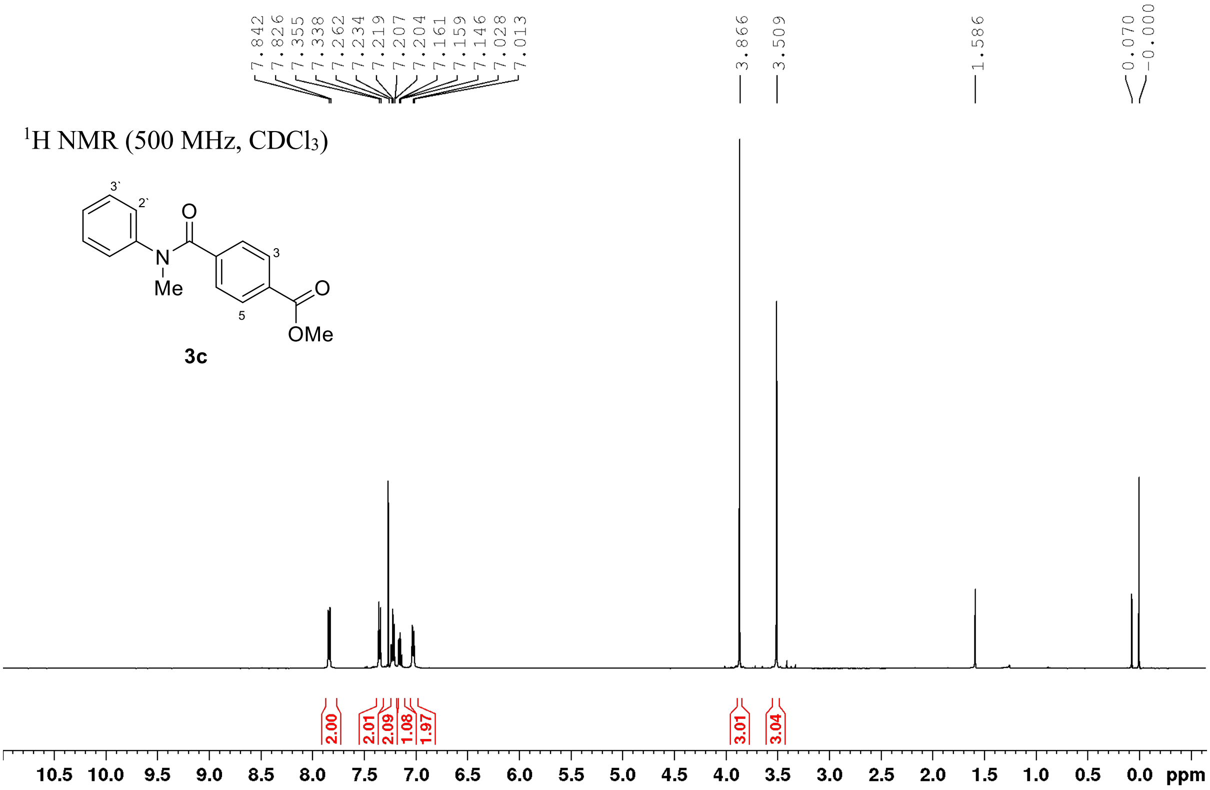


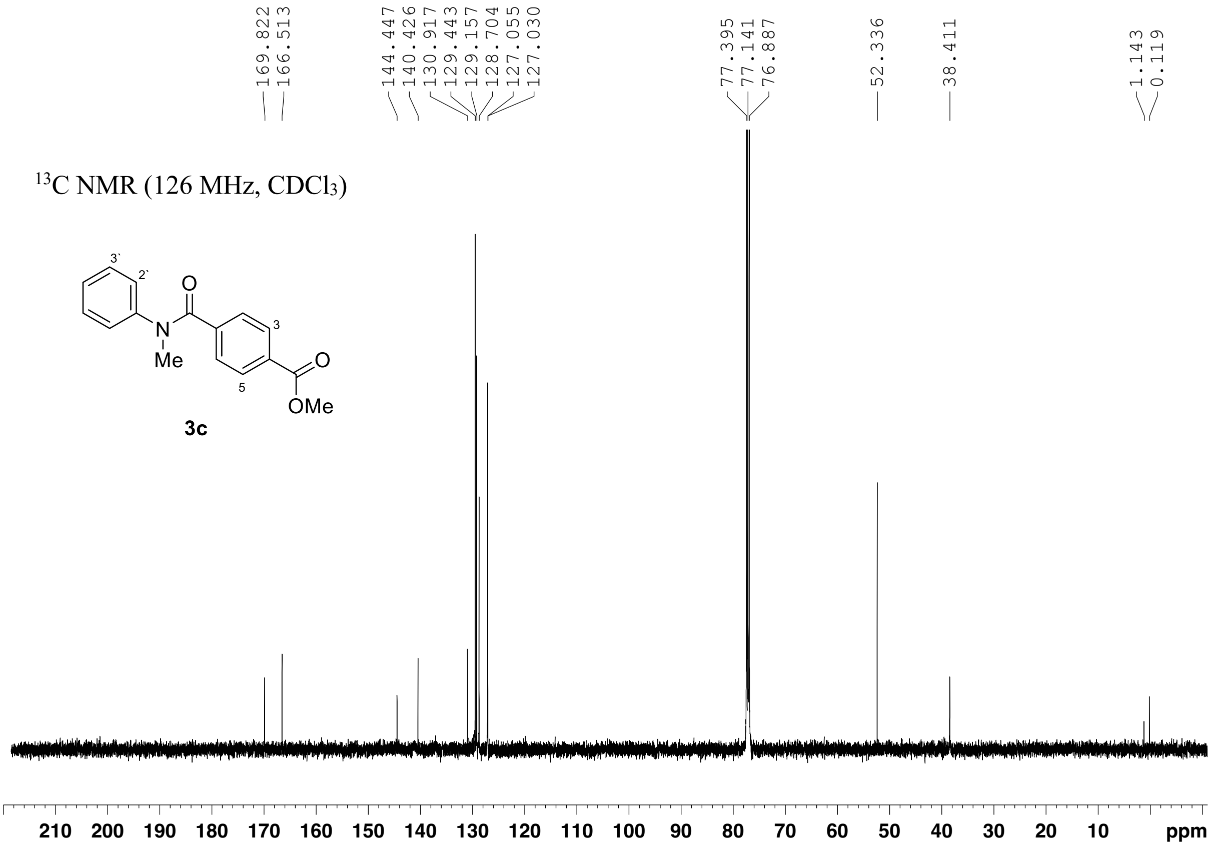

**_N_-Methyl-_N_-phenylquinoline-3-carboxamide 3e:**

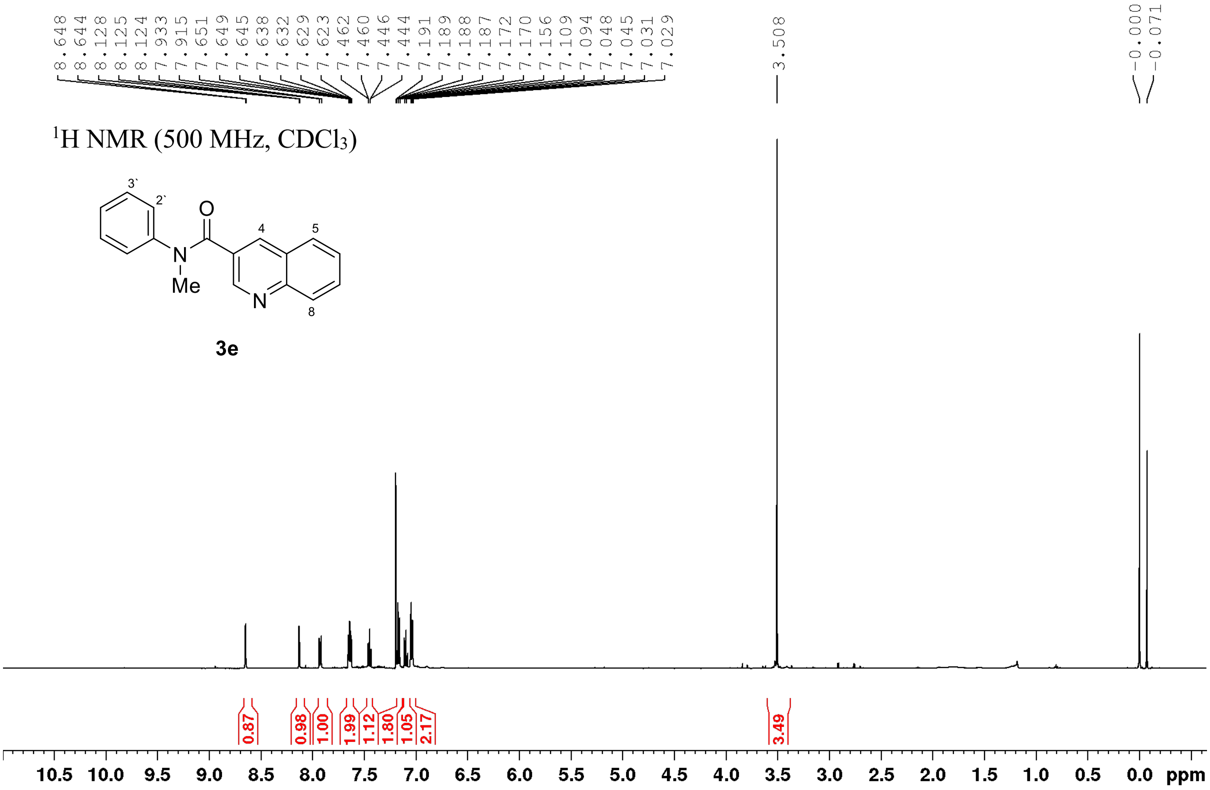


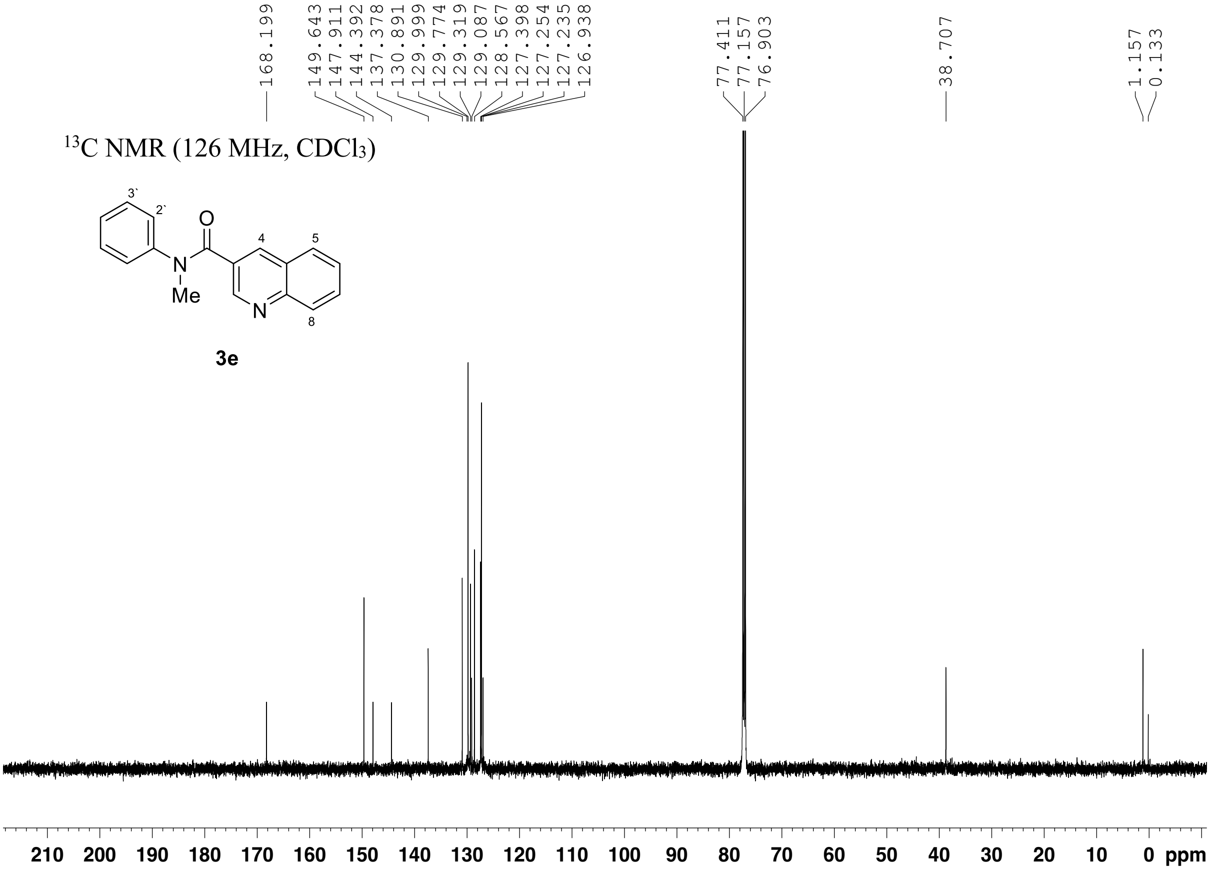

**N-Methyl-N-phenylpyrazine-2-carboxamide 3g:**

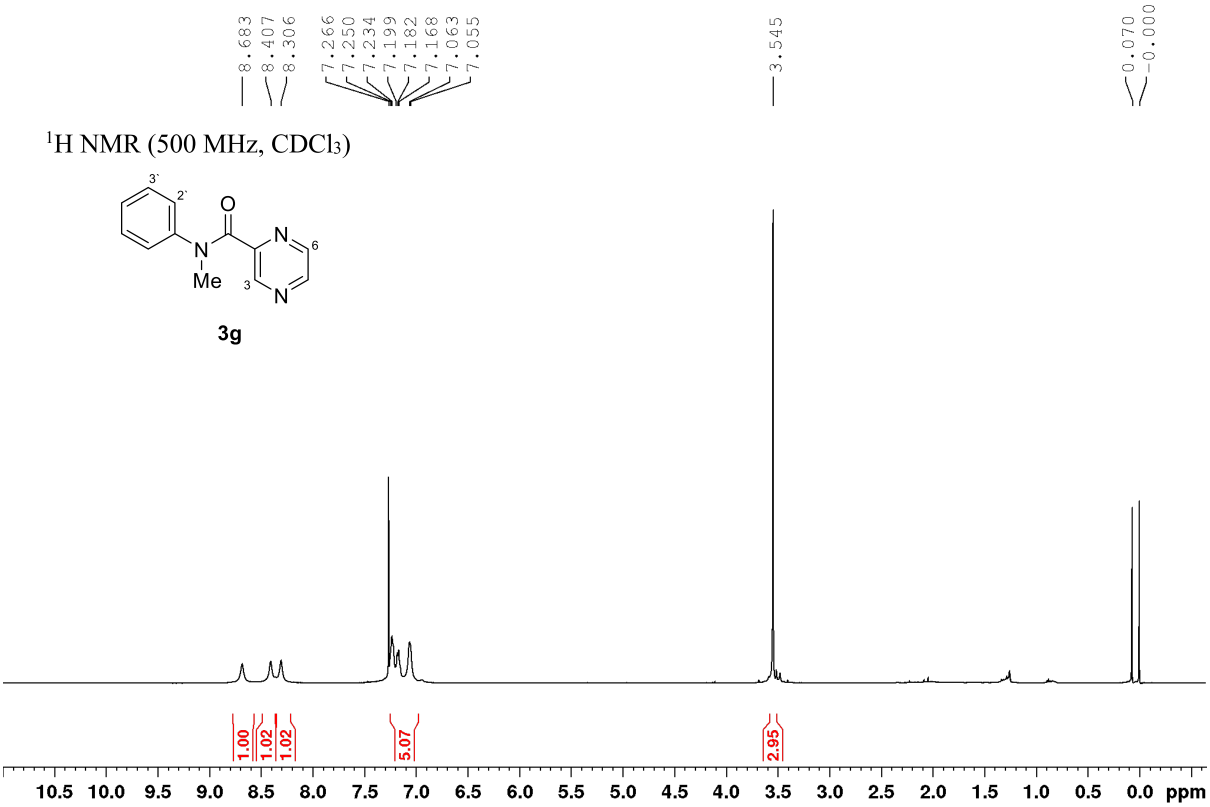


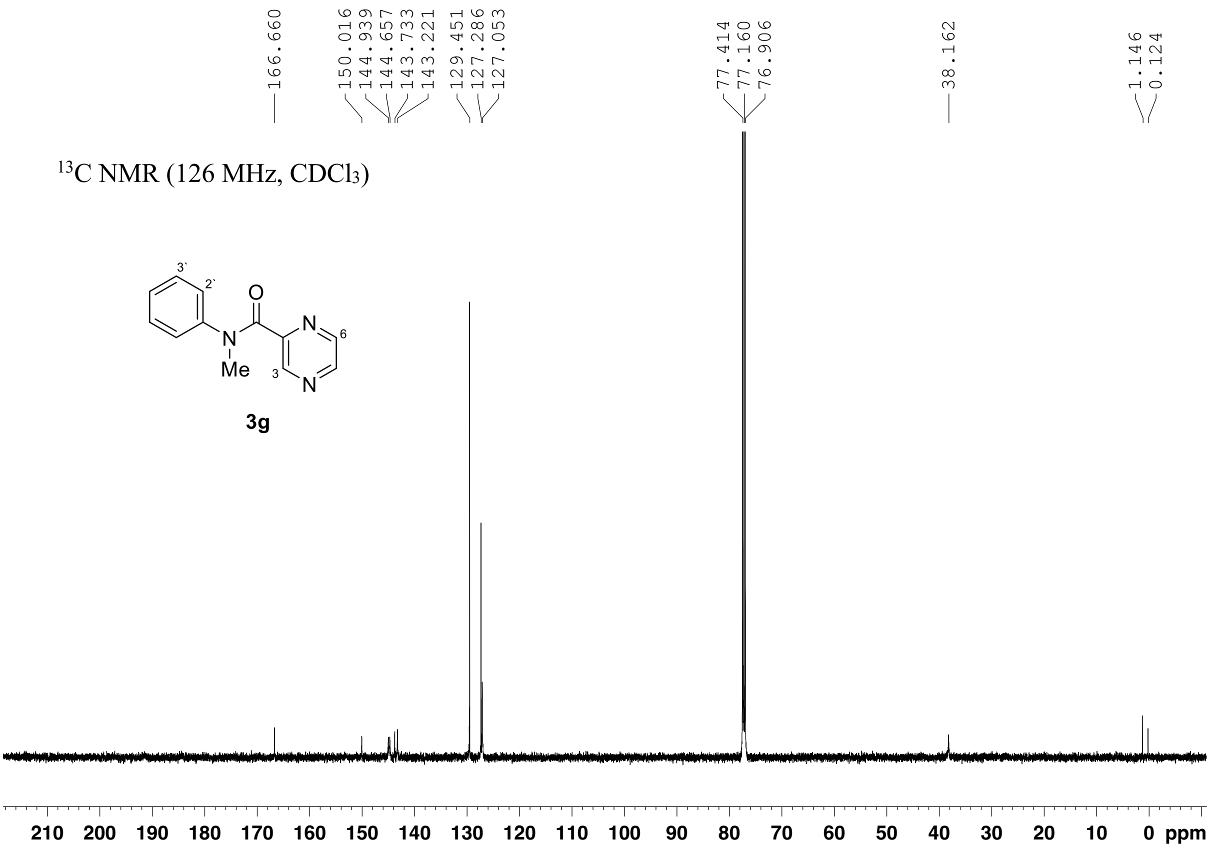

***N*-Methyl-*N*-(*p*-tolyl)-9*H*-carbazole-3-carboxamide 3h:**

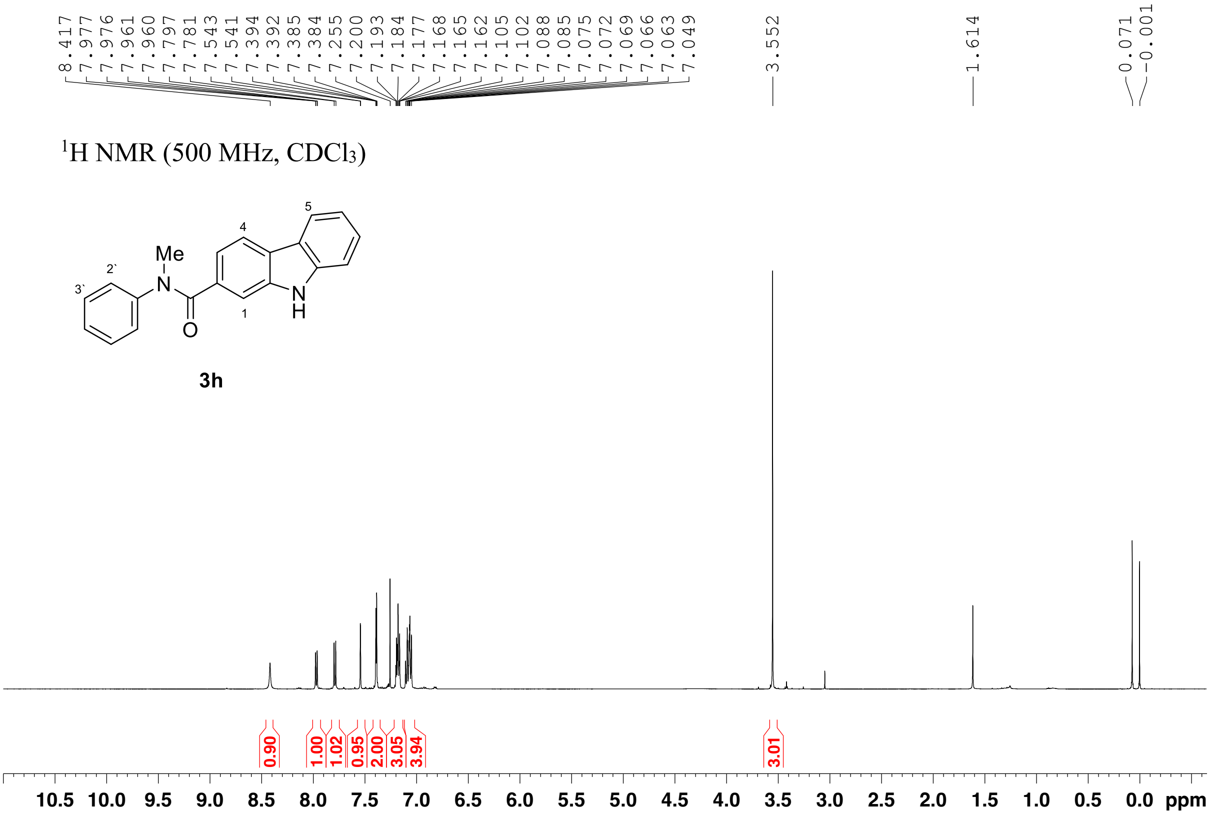


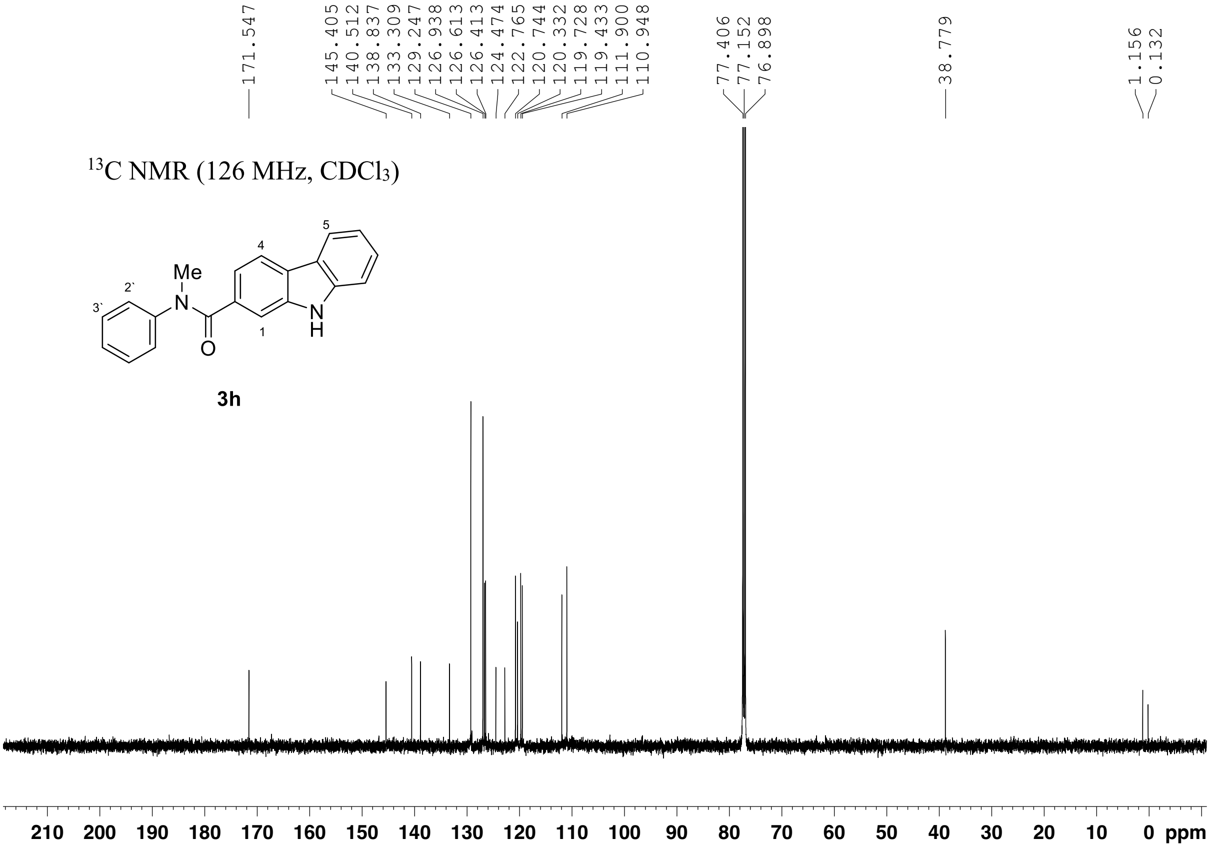

***N*-Methyl-*N*-phenylformamide-*d* 4a:**

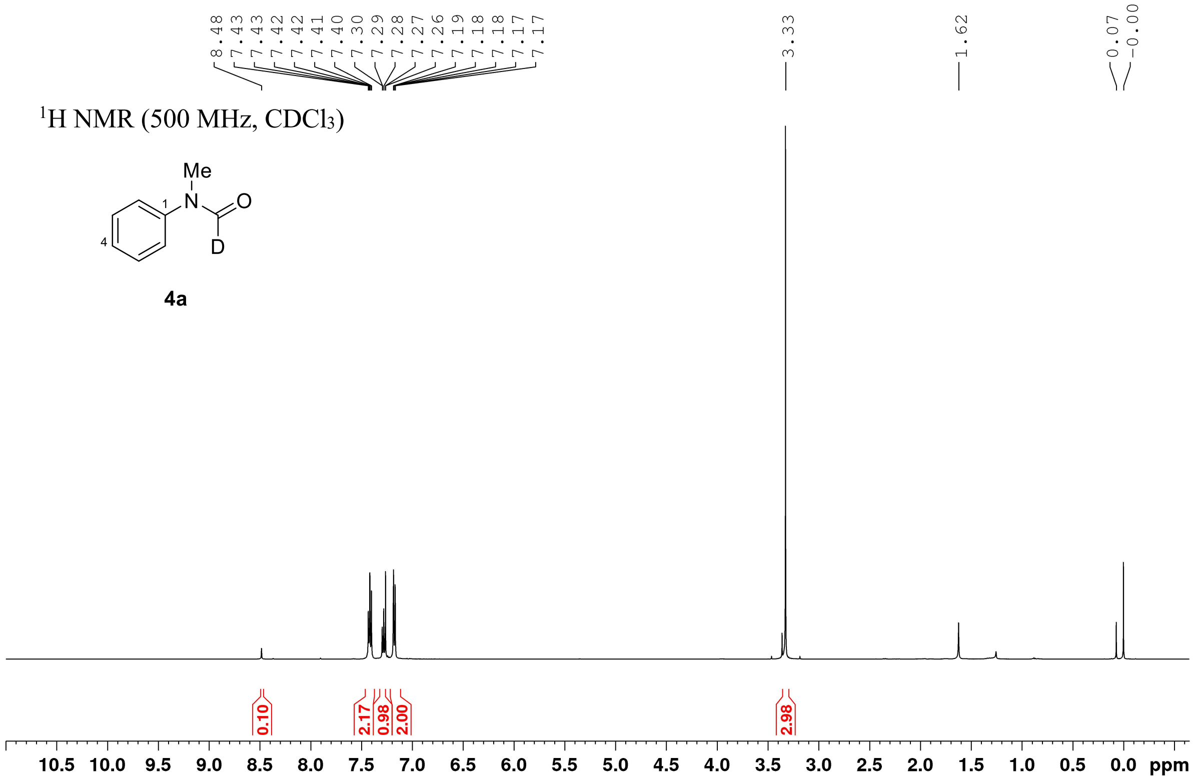


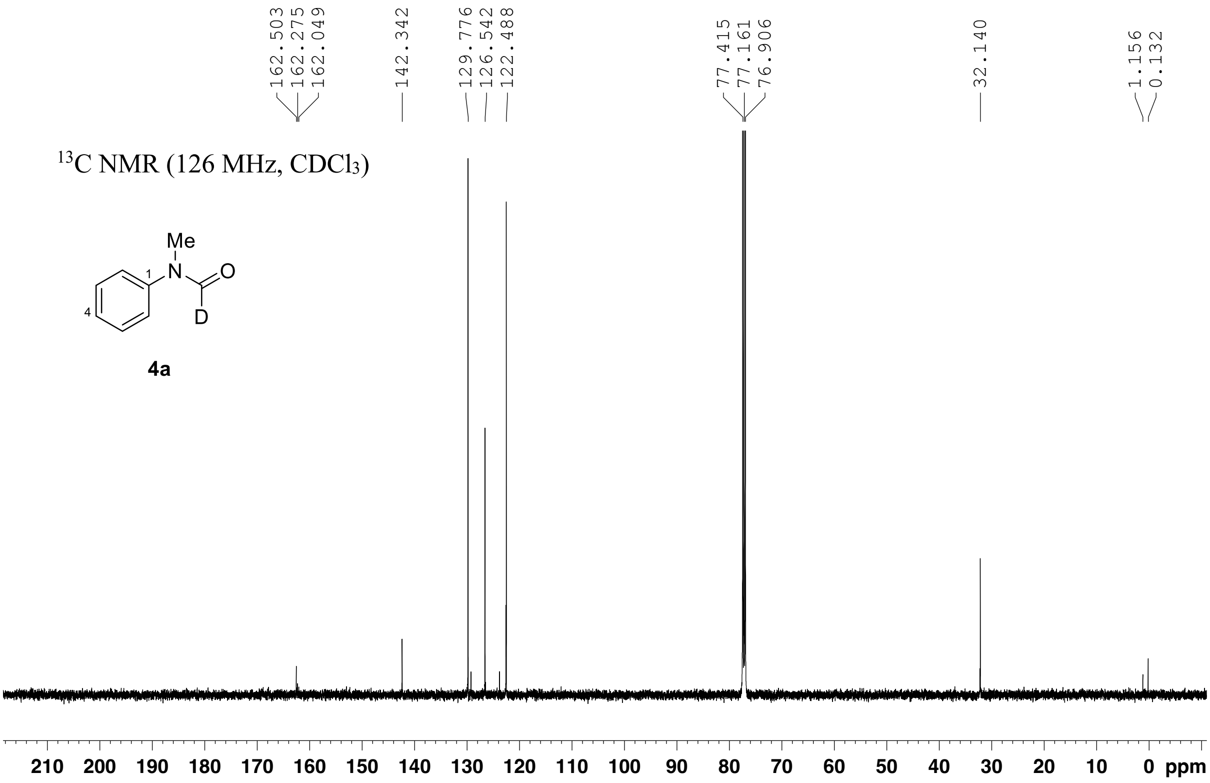

## *N*-Methyl-*N*-(*p*-tolyl)formamide-*d* 4b:

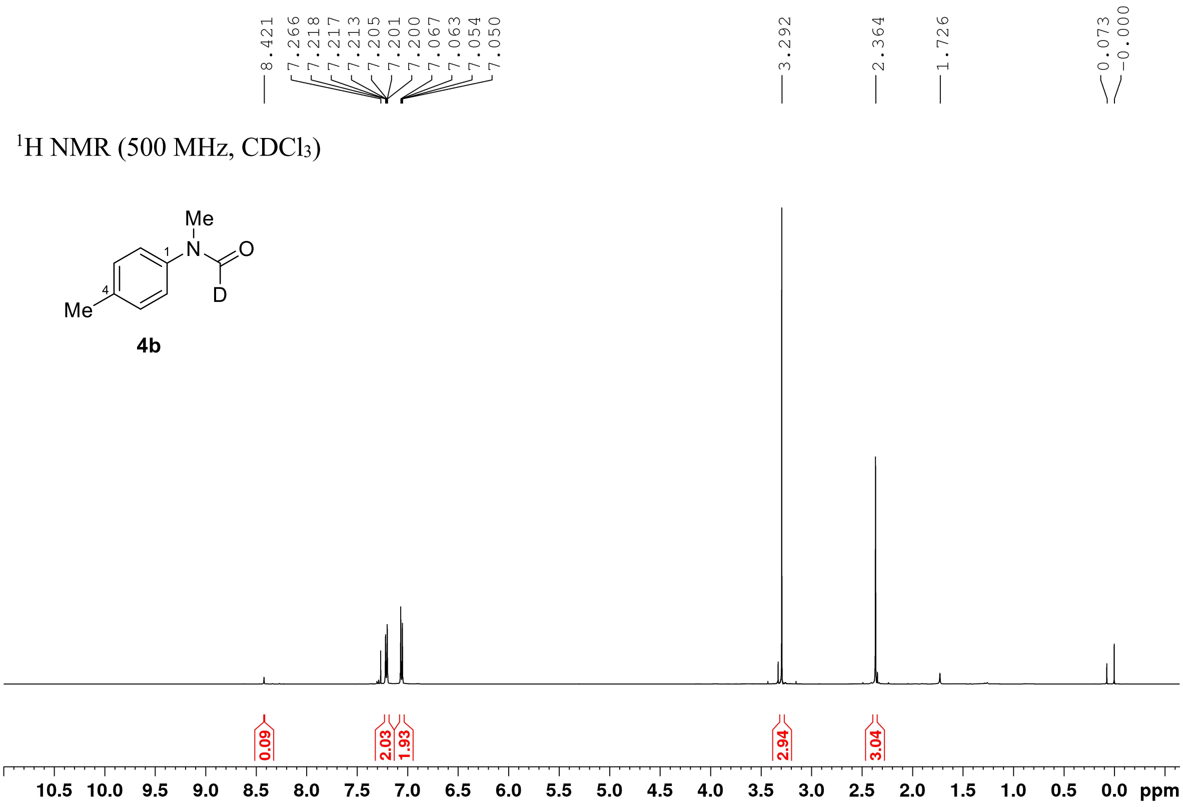


$^{1}H$ NMR (500 MHz, $CDCl_3$)

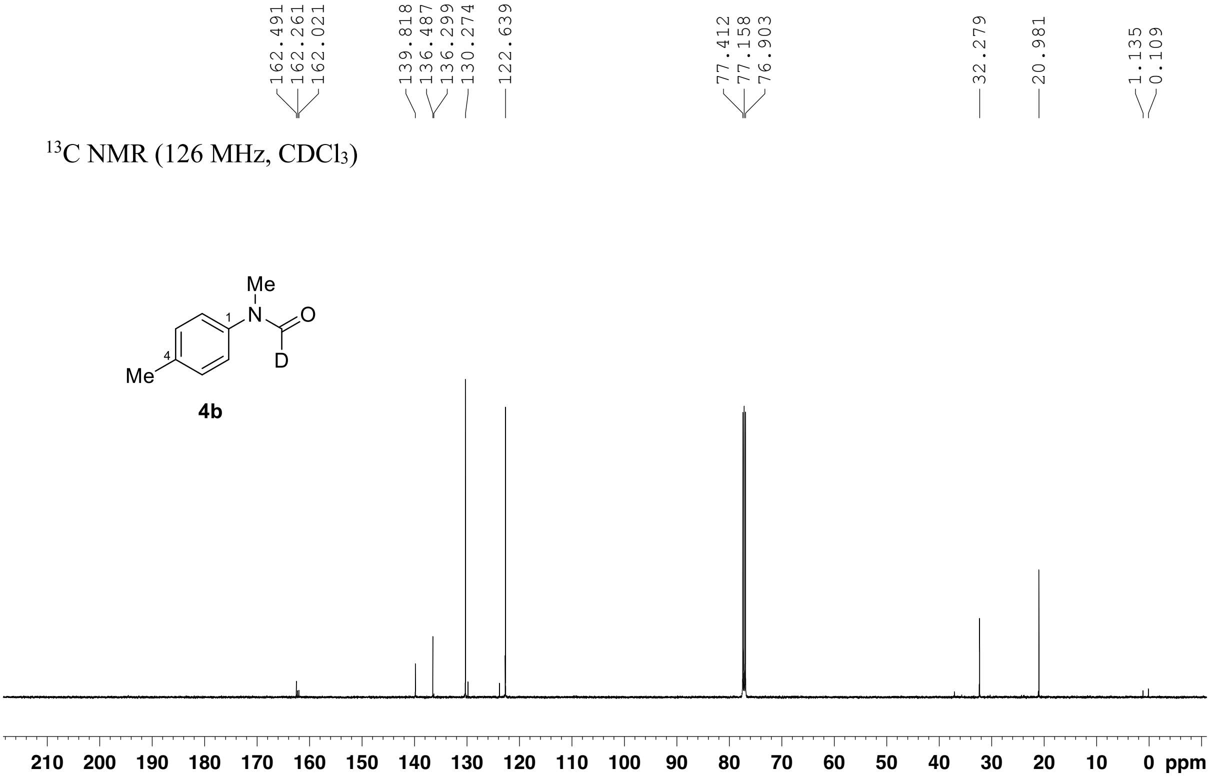


$^{13}C$ NMR (126 MHz, $CDCl_3$)

***N*-(4-Fluorophenyl)-*N*-methylformamide-*d* 4c:**

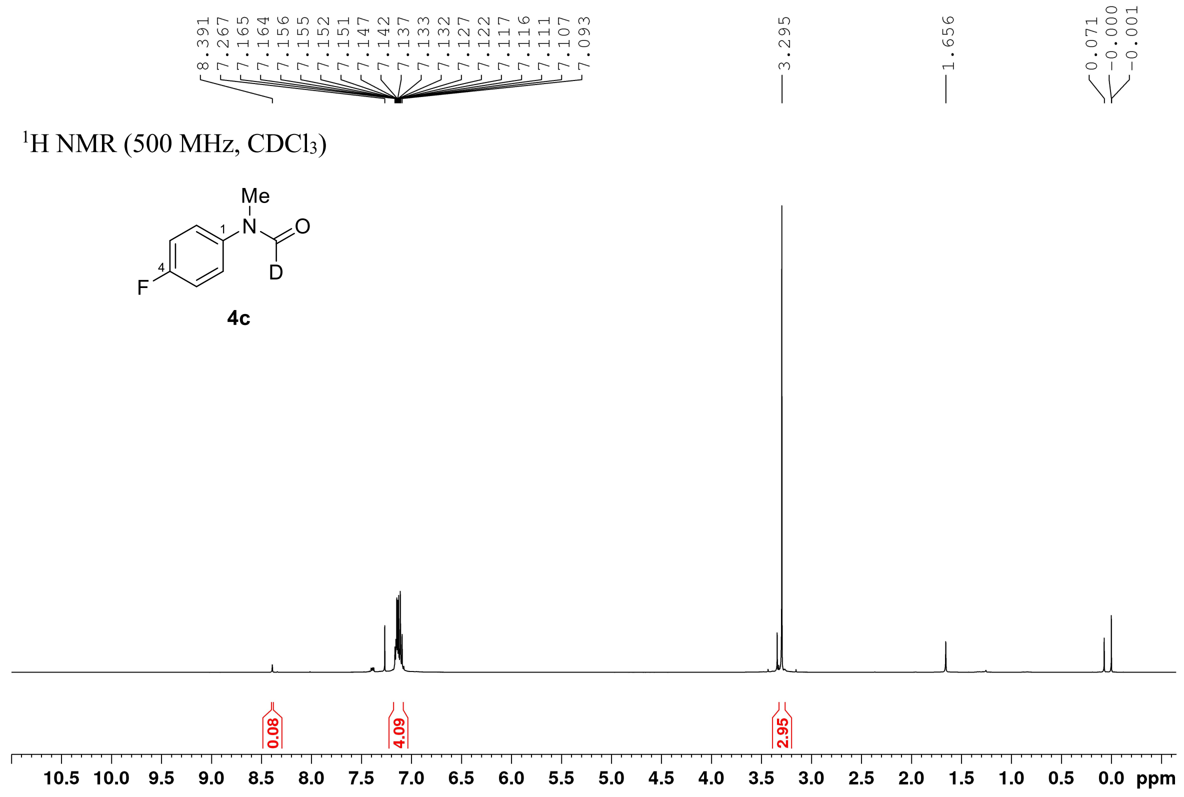


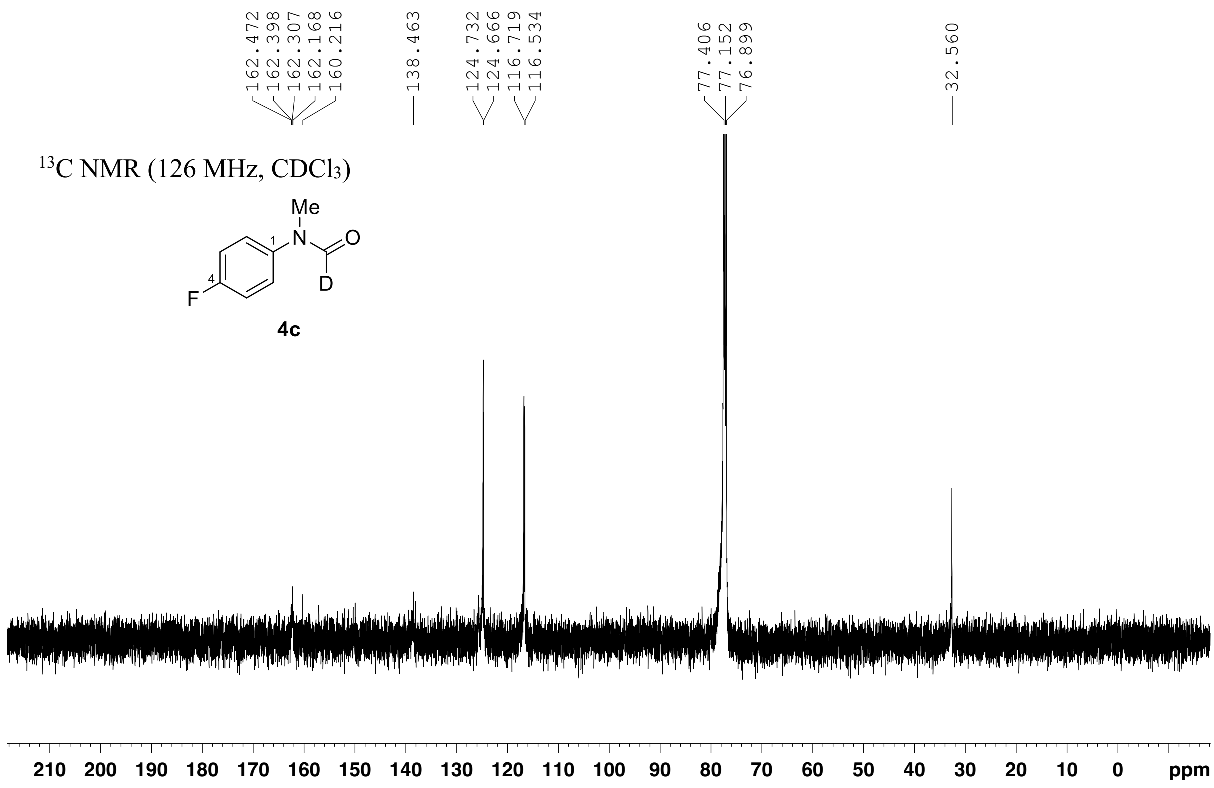

$^{19}F$ NMR (471 MHz, $CDCl_3$)

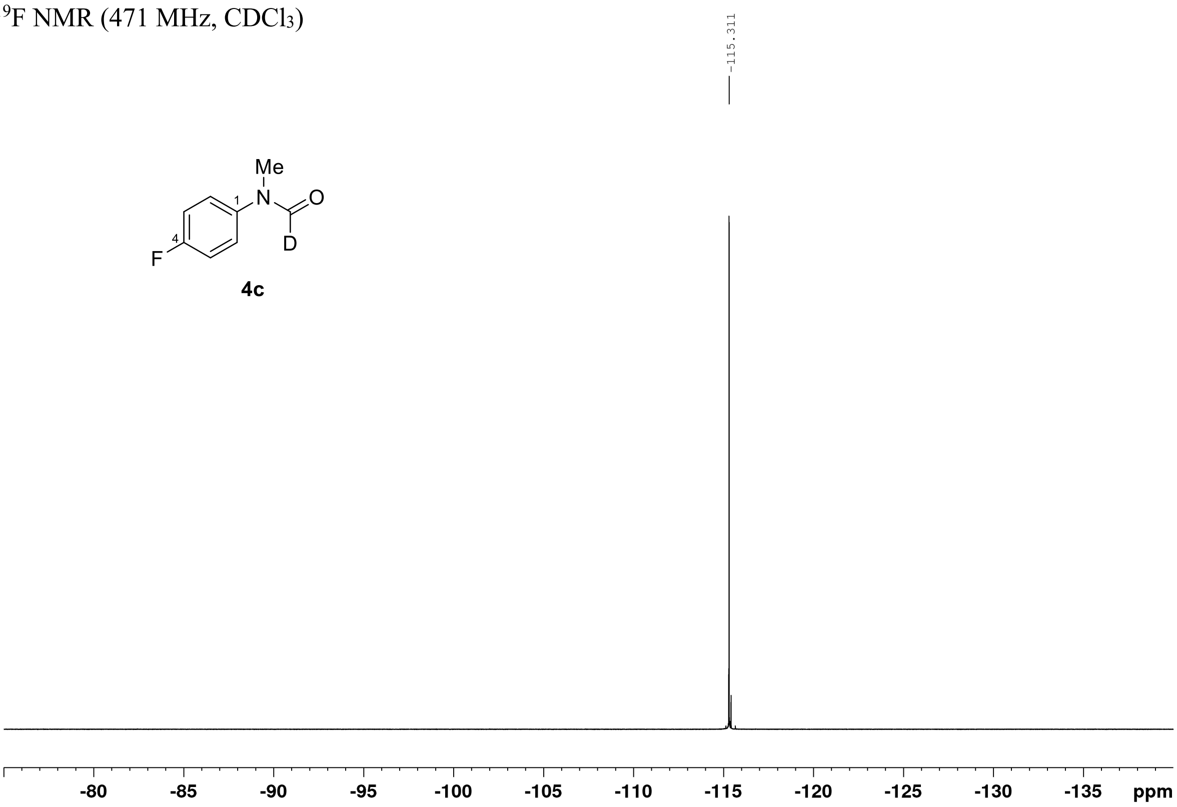

***N*,*N*-Diphenylformamide-*d* 4d:**

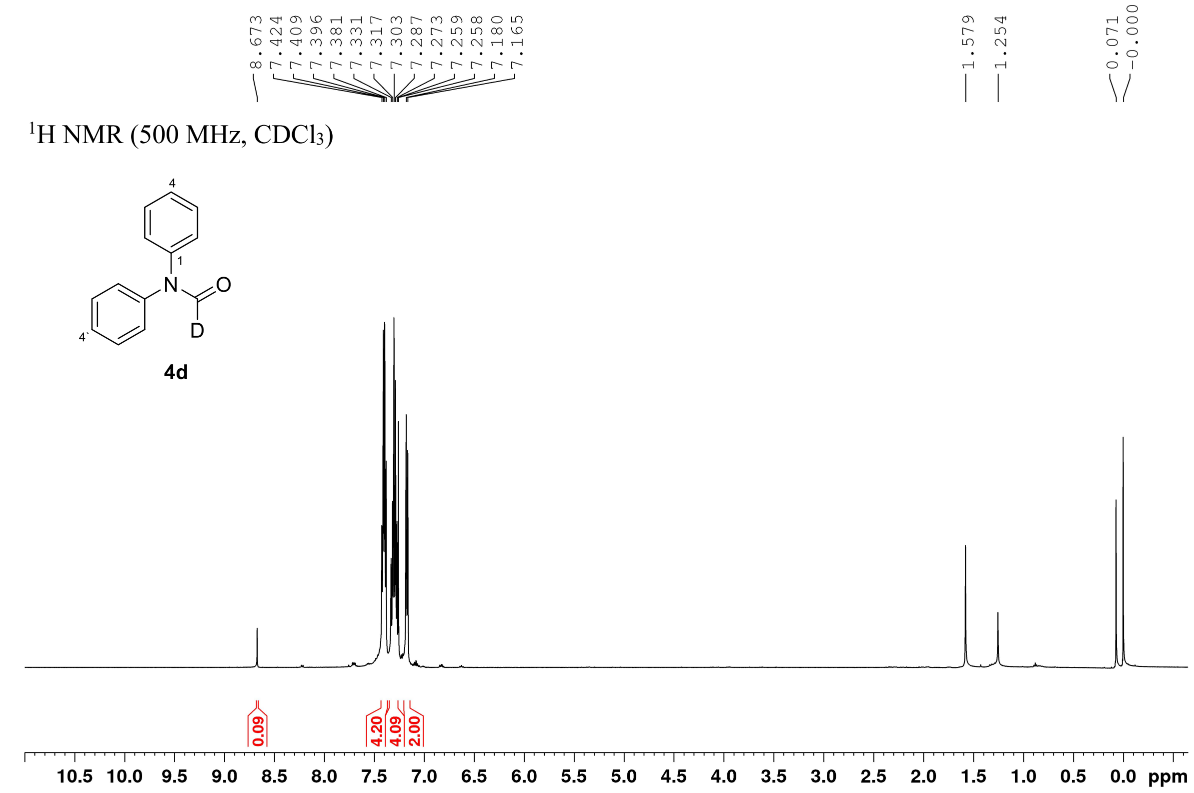


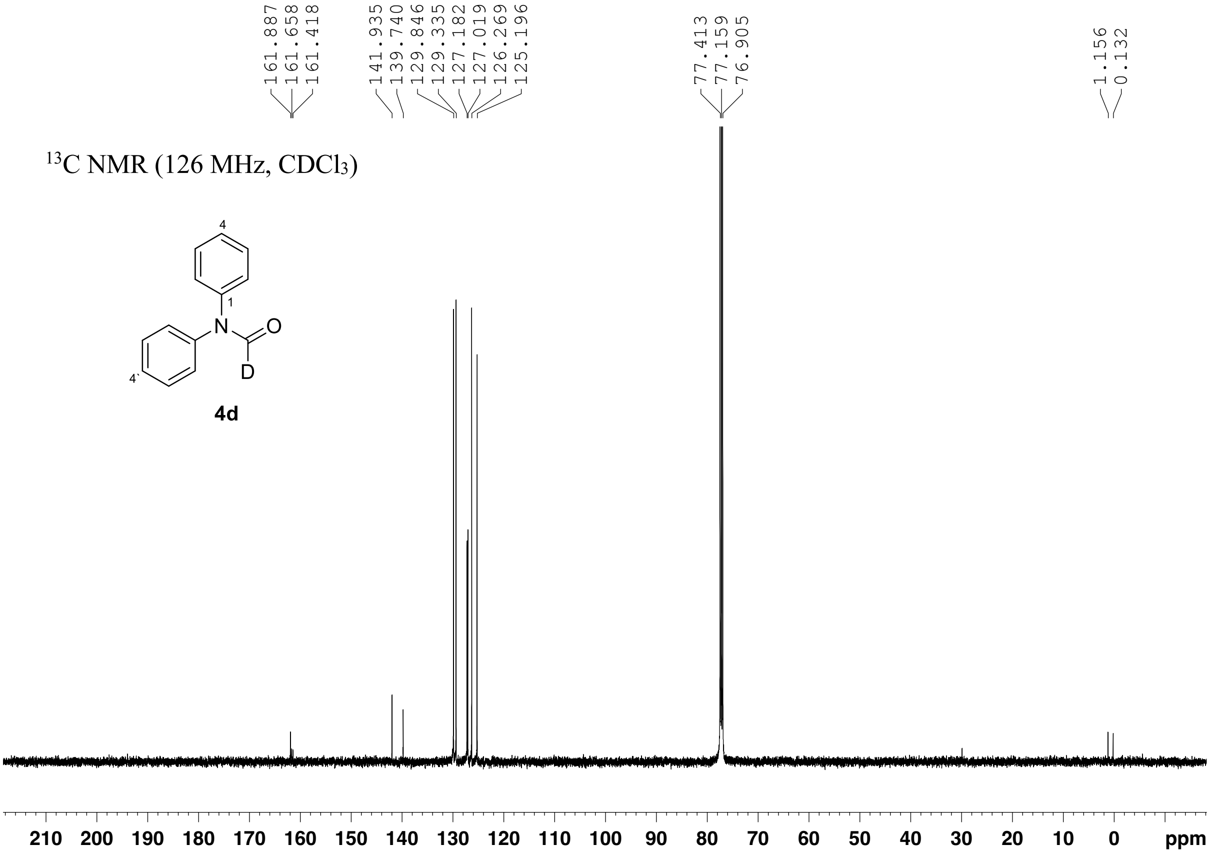

# 7. References


1. Bai, Q. F.; Jin, C.; He, J. Y.; Feng, G. Carbamoyl Radicals via Photoredox Decarboxylation of Oxamic Acids in Aqueous Media: Access to 3,4-Dihydroquinolin-2(1*H*)-ones. *Org. Lett.* **2018**, *20*, 2172–2175.
2. Mazodze, C. M.; Petersen, W. F. Silver-catalysed double decarboxylative addition–cyclisation–elimination cascade sequence for the synthesis of quinolin-2-ones. *Org. Biomol. Chem.* **2022**, *20*, 3469–3474.
3. Gediya, S. K.; Vyas, V. K.; Clarkson, G. J.; Wills, M. Asymmetric Transfer Hydrogenation of α-Keto Amides; Highly Enantioselective Formation of Malic Acid Diamides and α-Hydroxyamides. *Org. Lett.* **2021**, *23*, 7803–7807.
4. Chen, G.; Li, C.; Peng, J.; Yuan, Z.; Liu, P.; Liu, X. Silver-promoted decarboxylative radical addition/annulation of oxamic acids with gem-difluoroolefins: concise access to $CF_2$-containing 3,4-dihydroquinolin-2-ones. *Org. Biomol. Chem.* **2019**, *17*, 8527–8532.
5. Liu, J.; Zhang, B.; Hu, J.; Qiu, Z.; Chen, X.; Tian, X.; Wang, Q.; Zheng, G.; Yuan, M. Radical Arylaminoformylation of Activated Alkenes to Amides Containing All-Carbon Quaternary Stereocenters. *Eur. J. Org. Chem.* **2023***, 26*, e202201378.
6. Jafari, M.; V. Sriram, V.; Premnauth, G.; Merino, E.; Lee, J. Y. Modified peroxamide-based reactive oxygen species (ROS)-responsive doxorubicin prodrugs. *Bioorg. Chem.* **2022**, *127*, 105990–105998.
7. Zhang, Z.; Gao, X.; Yu, H.; Zhang, G.; Liu, J. Copper-Catalysed (Diacetoxyiodo)benzene-Promoted Aerobic Esterification Reaction: Synthesis of Oxamates from Acetoacetamides. *Adv. Synth. Catal.* **2018**, *360*, 3406–3411.
8. Kandale, A.; Patel, K.; Hussein, W. M.; Wun, S. J.; Zheng, S.; Tan, L.; West, N. P.; Schenk, G.; Guddat, L. W.; McGeary, R. P. Analogues of the Herbicide, *N*-Hydroxy-*N*-isopropyloxamate, Inhibit Mycobacterium tuberculosis Ketol-Acid Reductoisomerase and Their Prodrugs Are Promising Anti-TB Drug Leads. *J. Med. Chem.* **2021**, *64*, 1670–1684.
